# The Pseudo‐Natural Product Rhonin Targets RHOGDI

**DOI:** 10.1002/anie.202115193

**Published:** 2022-03-02

**Authors:** Mohammad Akbarzadeh, Jana Flegel, Sumersing Patil, Erchang Shang, Rishikesh Narayan, Marcel Buchholzer, Neda S. Kazemein Jasemi, Michael Grigalunas, Adrian Krzyzanowski, Daniel Abegg, Anton Shuster, Marco Potowski, Hacer Karatas, George Karageorgis, Niloufar Mosaddeghzadeh, Mia‐Lisa Zischinsky, Christian Merten, Christopher Golz, Lucas Brieger, Carsten Strohmann, Andrey P. Antonchick, Petra Janning, Alexander Adibekian, Roger S. Goody, Mohammad Reza Ahmadian, Slava Ziegler, Herbert Waldmann

**Affiliations:** ^1^ Department of Chemical Biology Max Planck Institute of Molecular Physiology Otto-Hahn-Straße 11 44227 Dortmund Germany; ^2^ Max Planck Institute of Molecular Physiology Otto-Hahn-Straße 11 44227 Dortmund Germany; ^3^ Institute of Biochemistry and Molecular Biology II Medical Faculty and University Hospital Düsseldorf Heinrich Heine University Düsseldorf Universitätsstrasse 1, Building 22.03.05 40225 Düsseldorf Germany; ^4^ School of Chemical and Materials Sciences IIT Goa, Farmagudi Ponda Goa-403401 India; ^5^ Department of Chemistry The Scripps Research Institute 130 Scripps Way Jupiter FL 33458 USA; ^6^ Lead discovery center Otto-Hahn-Str. 15 44227 Dortmund Germany; ^7^ Faculty of Chemistry and Biochemistry Organic Chemistry II Ruhr-University Bochum Universitätsstrasse 150 44780 Bochum Germany; ^8^ Faculty of Chemistry and Chemical Biology Technical University Dortmund Otto-Hahn-Straße 6 44221 Dortmund Germany

**Keywords:** Inhibitors, Liposomes, Osteogenesis, Proteins, Pseudo-Natural Products, RHOGDI, Small Molecules

## Abstract

For the discovery of novel chemical matter *generally* endowed with bioactivity, strategies may be particularly efficient that combine previous insight about biological relevance, e.g., natural product (NP) structure, with methods that enable efficient coverage of chemical space, such as fragment‐based design. We describe the de novo combination of different 5‐membered NP‐derived N‐heteroatom fragments to structurally unprecedented *“pseudo‐natural products”* in an efficient complexity‐generating and enantioselective one‐pot synthesis sequence. The pseudo‐NPs inherit characteristic elements of NP structure but occupy areas of chemical space not covered by NP‐derived chemotypes, and may have novel biological targets. Investigation of the pseudo‐NPs in unbiased phenotypic assays and target identification led to the discovery of the first small‐molecule ligand of the RHO GDP‐dissociation inhibitor 1 (RHOGDI1), termed Rhonin. Rhonin inhibits the binding of the RHOGDI1 chaperone to GDP‐bound RHO GTPases and alters the subcellular localization of RHO GTPases.

## Graphical abstract

The identification of the pseudo‐natural product Rhonin as the first small‐molecule modulator of RHO GDP dissociation inhibitor (RHOGDI) is reported. Rhonin binds to RHOGDI and interferes with its function by disrupting the interaction between RHOGDI and RHO GTPases.

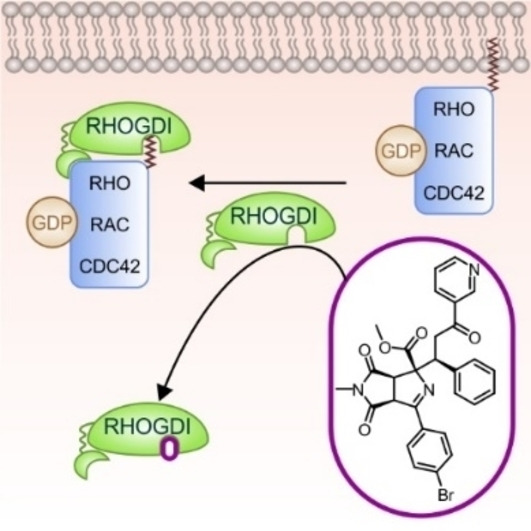

## Introduction

The discovery of novel chemical matter, which *in general* is endowed with bioactivity and biological relevance, is at the heart of chemical biology. Such compound classes may have new biological targets and modes of action, and, therefore, their bioactivities will best be evaluated in unbiased target‐agnostic phenotypic assays followed by target identification and validation.^[1]^

Strategies for the design of such novel compound classes can draw inspiration from previous insights about the biological relevance of compound classes, as for instance gained by Biology Oriented Synthesis (BIOS). In BIOS, complex natural product (NP) scaffolds are reduced to less complex, synthetically better accessible structures retaining the characteristic properties of the guiding NPs.^[2]^ However, BIOS covers only a small fraction of natural product‐like chemical space and arrives at compound classes that may retain similar bioactivities to the guiding NPs. These limitations can be overcome by the design and synthesis of “pseudo‐natural products” (pseudo‐NPs).^[3]^ In the pseudo‐NP concept, NP fragments that represent NP structure and properties^[4]^ are combined through de novo combination to afford unprecedented NP‐inspired compound classes not accessible by known biosynthesis pathways. Pseudo‐NPs inherit characteristic NP structures and properties but go beyond the chemical space explored by nature and, therefore, promise to have unexpected bioactivity and targets.

**Figure 1:**
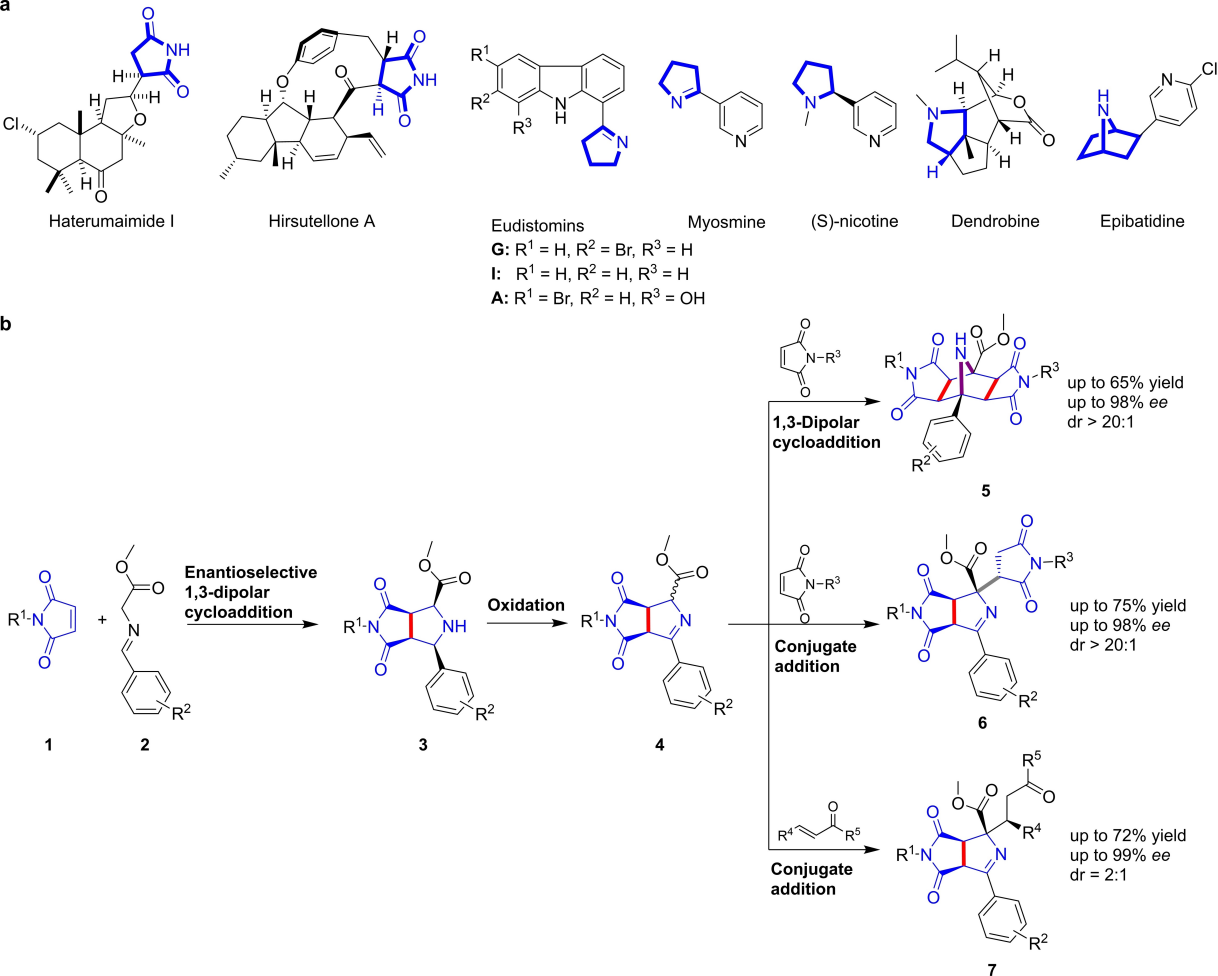
Design of a pseudo‐NP collection. a) Representative natural products embodying 5‐membered N‐heterocycles. b) Tandem catalysis sequence for the synthesis of a pseudo‐NP collection containing 5‐membered N‐heterocycles in different connectivities.

Five‐membered N‐heterocycles are defining structural units of numerous natural products with diverse bioactivities. For instance, succinimides occur in the haterumaimides, which have antitumor activity,^[5]^ and the fungal metabolite hirsutellone, which is active against *Mycobacterium tuberculosis* (Figure 1a).^[6]^ Pyrrolines are characteristic structural elements of eudistomin alkaloids with calmodulin antagonist activity (Figure 1a)^[7]^ and the tobacco alkaloid myosmine^[8]^ (Figure 1a). Pyrrolidines occur as isolated scaffolds in various structurally simple alkaloids like nicotine or fused to other scaffolds in structurally more complex alkaloids, such as dendrobine (Figure 1a). Additionally, two pyrrolidines are fused in a bicyclic [2.2.1] arrangement in the nicotin receptor agonist epibatidine (Figure 1a).

Inspired by this diverse occurrence of five‐membered N‐heterocycles in NPs, we designed and synthesized a pseudo‐NP collection that combines these fragments in different connectivities. Phenotypic investigation of bioactivity and target identification led to the discovery of Rhonin, a novel inhibitor of Hedgehog‐induced osteogenesis. Rhonin is the first small‐molecule ligand of the RHO GDP‐dissociation inhibitor1 (RHOGDI1) and inhibits binding of this chaperone to GDP‐bound RHO GTPases.

## Results and Discussion

### Establishment of a Tandem Catalysis Sequence

For the synthesis of a pseudo‐NP collection, we aimed to combine 5‐membered N‐heterocycle fragments in a complexity‐generating manner with different connectivities (Figure 1b), i.e., such that i) the fragments do not share atoms and are linked via one bond (monopodal connection; gray bonds, Figure 1b), ii) they share two atoms linked via a common bond (edge fusion; red bonds, Figure 1b) or they may be linked in a bicyclic arrangement sharing three atoms and two bonds (bridge fusion; magenta bonds, Figure 1b). Thereby related but different pseudo‐NPs could be synthesized based on a limited set of fragments.

It was initially planned to construct pyrrolidines by means of an enantioselective dipolar cycloaddition of azomethine ylides with maleimides. This would yield an edge‐fused pyrrolidine‐succinimide pseudo‐NP class, i.e., **3**. Subsequent oxidation of the pyrrolidine to an imine would give rise to a succinimide‐pyrroline combination **4** which can undergo further transformations. The imine could be converted to a new azomethine ylide which might react with maleimides in a second 1,3‐dipolar cycloaddition to yield a double fused pseudo‐NP class **5** combining two succinimides with a bicyclic azabicyclo[2.2.1] scaffold, characteristic of epibatidine. Nucleophilic addition to maleimides will generate a pseudo‐NP class **6** containing two fragments linked by an edge fusion to a second succinimide fragment via a monopodal connection. Finally, conjugate addition to different α,β‐unsaturated electrophiles would yield pseudo‐NPs **7** in which a succinimide and a pyrrolidine are combined, and the side chain may contain additional natural product fragments.

Using this divergent synthesis approach, several different pseudo‐NP types would be efficiently accessible by the unified strategy. This synthetic strategy offers several attractive features. The metal‐catalyzed 1,3‐dipolar cycloaddition and the subsequent regio‐ and chemoselective oxidation could potentially be coupled in a novel tandem catalytic approach in which the metal catalyst used for the cycloaddition could be employed in combination with an oxidizing agent. Such tandem catalysis sequences combining two or more mechanistically distinct chemical reactions are considered to be particularly attractive since they enable expedient generation of molecular complexity and efficiency of the reaction sequence.^[9]^ Hitherto, Δ^1^‐pyrrolines have been synthesized by means of cycloaddition of Münchnones to electron‐deficient alkenes.^[10]^ Thus, the tandem catalysis strategy outlined in Figure 1b also represents a novel method for the synthesis of this compound class.

**Figure 2:**
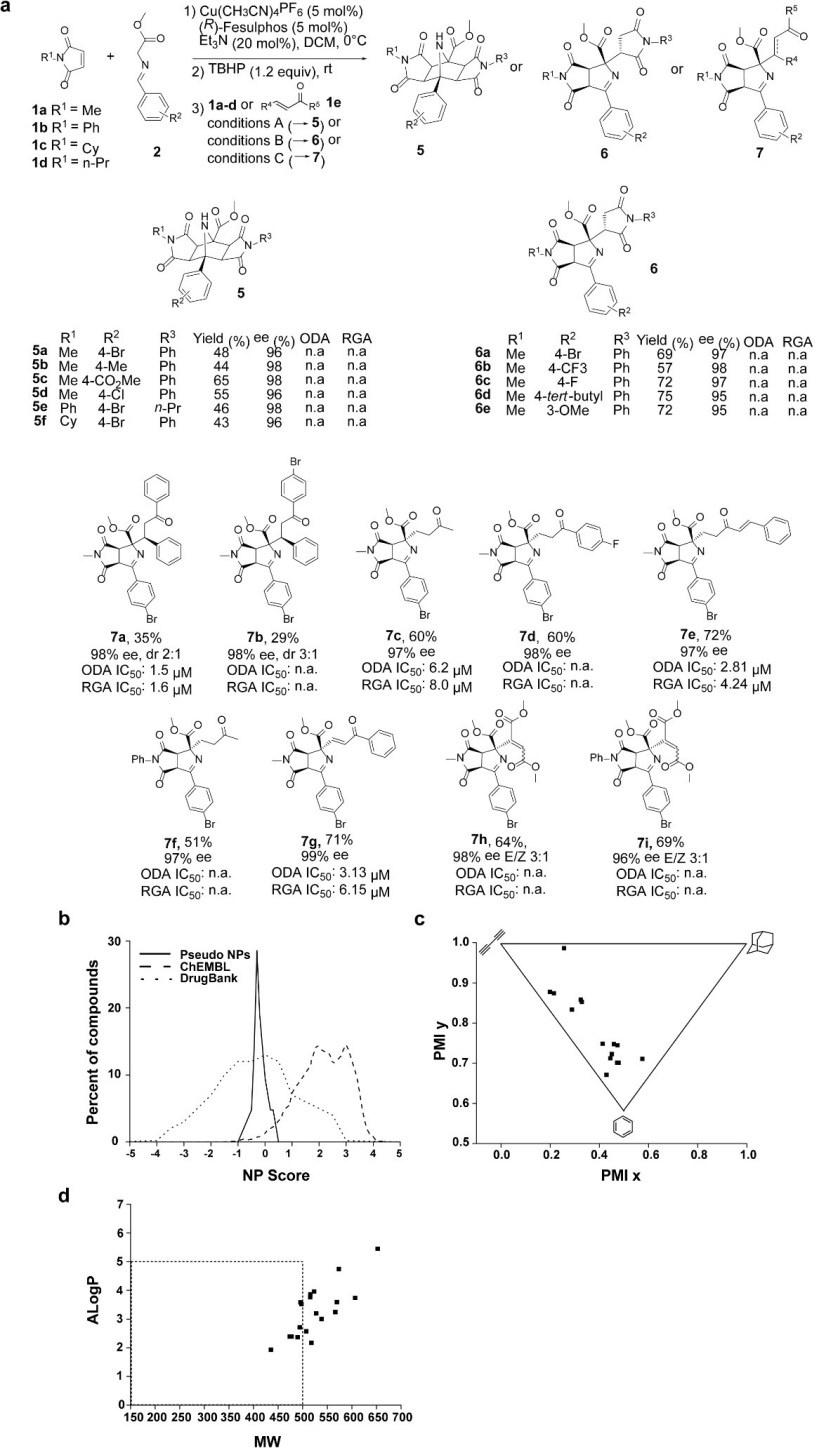
Synthesis of pseudo‐NPs that occupy a distinct portion of chemical space. a) Reaction conditions: A) **1 a**–**d** (3 equiv), DBU (0.5 equiv), THF, rt; B) **1 a**–**d** (1.5 equiv), Et_3_N (1.5 equiv), DCM, rt; C) **1 e** (1.5 equiv), DBU (0.5 equiv), DCM, rt. For **7 a**, the yield represents the epimeric mixture of the phenylethyl ketone. ODA: Osteoblast differentiation assay. RGA: Reporter gene assay. All ODA and RGA data are mean values of three independent experiments (*n*=3). b) NP‐likeness score comparison of NPs represented in ChEMBL (dashed curve), Drugbank (dotted curve) and succinimide‐pyrroline pseudo‐NPs (solid curve). c) PMI plot for succinimide‐pyrroline pseudo‐NPs. The average of PMI coordinate distribution is shown by a cross. d) ALogP vs MW plot of succinimide‐pyrroline pseudo‐NPs.

In order to identify suitable reaction conditions for the tandem catalysis sequence, azomethine ylide **2 a** (Figure 2a; R^2^=4‐Br) was allowed to react with *N*‐methylmaleimide **1 a** (R^1^=Me) in CH_2_Cl_2_ in the presence of Cu(CH_3_CN)_4_PF_6_ as catalyst and (*R*)*‐*Fesulphos [(*R_p_*)‐2‐(*tert*‐butylthio)‐1‐(diphenylphosphino)ferrocene)] as chiral ligand for the 1,3‐dipolar cycloaddition.[^11^, ^12^] Subsequent addition of TBHP as a terminal oxidant for the Cu^I^‐catalyzed oxidation gratifyingly yielded the desired pyrroline **4 a** (Figure 1b; R^1^=Me, R^2^=4‐Br) in good yield (82 %) and with complete regio‐ and chemoselectivity. The combination of these two steps with the envisaged additional cycloaddition and conjugate addition required careful optimization of the reaction conditions. After substantial experimentation, the use of 1.5 equivalents of both Et_3_N and maleimide in CH_2_Cl_2_ was found to be best for the formation of Michael addition products **6** (Tables S1 and S2). The double cycloaddition to tricyclic products **5** proceeded best in the presence of 0.5 equiv. of DBU in THF (Tables S3–S5). Furthermore, in the presence of CH_2_Cl_2_ and DBU, nucleophilic addition to acyclic Michael acceptors occurred and products **7** were obtained.

### Synthesis of a Pseudo‐NP Collection

The successful identification of conditions for the selective formation of the three envisaged compound classes enabled the assembly of a pseudo‐NP collection. In the synthesis of double cycloadducts **5** (Figure 2a, conditions A), the aromatic ring of the azomethine ylides **2** can be varied (Figure 2a, **5 a**–**5 d**). Electron‐donating and ‐accepting substituents on the phenyl ring were well tolerated and gave the cycloadducts **5 a**–**d** in good yields and with generally excellent enantioselectivity (see Figure 2a). In addition, both aryl and alkyl maleimides could be successfully employed in the reaction in different order and combinations (Figure 2a, **5 e**, **5 f**).

Under the conditions identified for the Michael addition to unsaturated cyclic electrophiles, a variety of azomethine ylide precursors embodying electron‐donating or ‐withdrawing substituents gave the corresponding products **6** in excellent yields and with high diastereo‐ and enantioselectivities (Figure 2a, **6 a**–**6 e**), regardless of the electronic nature and the position of the substituents on the phenyl ring in the dipole. Acyclic electrophiles, like chalcone and different vinyl‐ and ethynyl ketones, gave the corresponding products **7** in good yields and with high *ee* (Figure 2a, **7 a**–**7 i**). Notably, in the case of styryl‐vinyl ketone, a single regioisomer **7 e** was obtained in 72 % yield. Ethynyl‐phenyl ketone yielded the *E*‐isomer **7 g** in 71 % yield. In total 21 pseudo‐NPs were synthesized in multi‐milligram amounts (typically ca. 5 mg per compound).

The relative configuration of the cycloadducts was unambiguously assigned by means of a crystal structure obtained for *rac*‐**6 a**. By means of VCD spectroscopy,^[13]^ the absolute configuration of the major diastereomer of **7 a** was determined as (*S*)‐**7 a**. For **7 h**, a crystal structure analysis established the *E*‐configuration (see the Supporting Information for details). Since the diastereoselectivity of the last functionalization is determined by the two stereocenters established in the first cycloaddition, the absolute configuration of all other compounds was assigned by analogy. For a mechanistic proposal to rationalize the observed direction and level of stereochemical induction (see Scheme S1).

These results demonstrate that the synthesis strategy efficiently yields a pseudo‐NP collection including the formation of three stereocenters and a tetrasubstituted carbon atom in a highly efficient one‐pot sequence.

### Cheminformatic Analysis

The chemical space occupied by the new pseudo‐NPs was analyzed by employing the natural‐product score (NP‐score) distribution.^[14]^ Since the majority of the collection is defined by pyrrolines fused to succinimides, the NP‐score was calculated for the sub‐library defined by this scaffold and compared with both the score calculated for NPs in ChEMBL^[15]^ and the score calculated for marketed and experimental drugs listed in DrugBank.^[16]^ The pyrroline‐derived pseudo‐NPs display a narrow distribution in a region of the NP‐score graph which is sparsely covered by NPs (Figure 2b). The fact that the combination of NP‐derived fragments yields compounds with properties diverging from NPs may be counterintuitive. However, the fragment combination generated here is not encountered in nature, such that the NP‐score distribution of these pseudo‐NPs should be different to NPs themselves. Comparison to the set of compounds in DrugBank, which represent approved and experimental drugs, demonstrates that the pseudo‐NPs display NP‐scores in an area populated by synthetically accessible, biologically relevant molecules. However, an additional analysis of the principal moments of inertia (PMI),^[17]^ used as a measure of molecular shape, revealed that the pseudo‐NPs described here have a higher degree of three‐dimensionality (Figure 2c) compared to typical synthetically accessible compound collections.^[18]^ This observation is supported by the average distance of points from the rod‐disc axis calculated to 0.12, as well as the cumulative distance value which was calculated to 2.41 (see Figure S1). Additionally, the average fraction of *sp* ^3^ hybridised carbons of these pseudo‐NPs was calculated to 0.31, which is within literature suggested range of values^[19]^ (see Table S6). Further analysis using Lipinski‐rule‐of‐5 (Lipinski‐Ro5) criteria showed that only 42 % of the newly synthesized collection is included within the limits of drug‐like space (Figure 2d), indicating that de novo combinations of NP‐derived fragments may result in compound collections with enhanced biological relevance even when deviating from established metrics.

The analysis indicates that the succinimide‐pyrroline pseudo‐NPs may occupy a previously not accessible fraction of NP‐inspired chemical space, reflecting the fact that they are not obtainable via current biosynthetic pathways. This novel scaffold may be endowed by design with advantageous physiochemical properties, as the pseudo‐NP collection displays a NP‐score distribution closer to the region occupied by approved drugs, even if the majority of the collection falls outside the limits of Lipinski‐Ro5 space.

### Biological Evaluation of the Pseudo‐NP Collection

**Figure 3:**
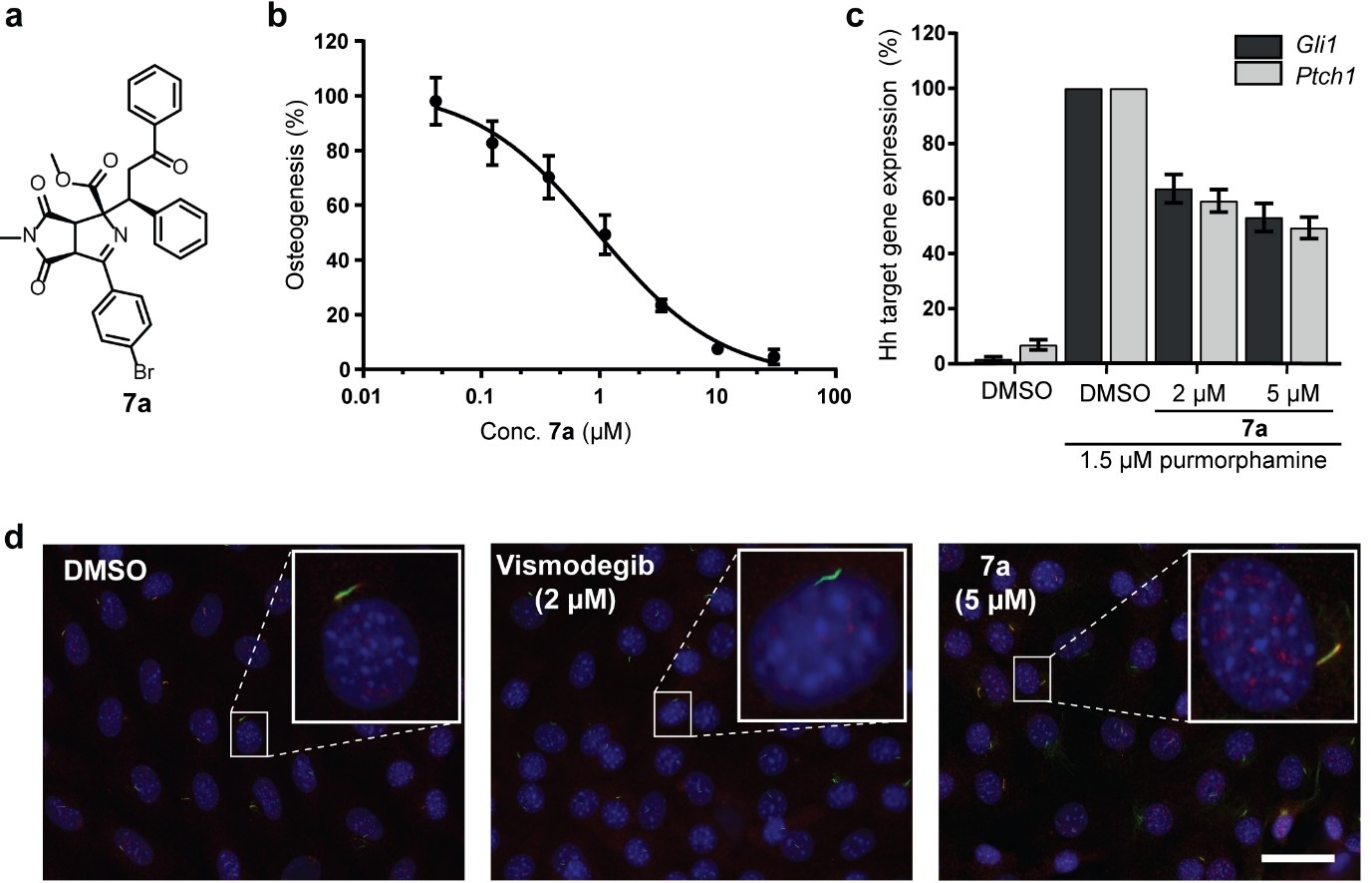
Compound **7 a** inhibits Hh‐induced osteogenesis. a) Structure of **7 a**. b) Hh‐induced osteogenesis in C3H/10T1/2 cells. Cells were treated with 1.5 μm purmorphamine and compound **7 a** for 96 h prior to detection of alkaline phosphatase activity (mean±SD, *n*=3). c) C3H/10T1/2 cells were treated with purmorphamine (1.5 μm) and of **7 a** or DMSO for 96 h prior to detection of the expression of *Ptch1*, *Gli1*, *Ap3d1* and *Gapdh* by means of RT‐qPCR. (mean± SD, *n*=3). d) Detection of SMO in cilia in NIH/3T3 cells. Blue: nuclei; red: SMO; green: acetylated tubulin. Insets: representative single cilia. Scale bar: 10 μm.

Investigation of biological activity of the pseudo‐NP collection in several cell‐based assays monitoring modulation of autophagy, Wnt signaling, reactive oxygen species (ROS) induction, Notch signaling and Hedgehog (Hh) signaling revealed that the pyrroline‐derived compounds are selective inhibitors of Hh pathway‐dependent osteogenesis in pluripotent mouse mesenchymal C3H/10T1/2 cells (see Table S7). Osteogenesis was induced using the Smoothened agonist purmorphamine. Despite the limited number of compounds, trends for structure–activity correlation became apparent. Thus, extension of the ketone part of the most potent hit **7 a**, e.g., by introduction of a *para*‐Br substituent into the aryl ketone part (to yield **7 b**), or by a *para‐*F into the phenyl ring (Figure 2a, compare **7 a** and **7 d**) abolished activity. The configuration of the stereocenter generated in the final conjugate addition to yield, e.g., **7 a**, has only minor impact on the bioactivity (Table S7, compare **7 a** and its epimer **7 a**–**epi**). A phenyl group is not strictly required in the electrophile for activity, since methyl‐vinyl ketone yielded active compound **7 c** (Figure 2a and Table S7, **7 c**). However, in the presence of a phenyl group derived either from the aryl ketone part or the aryl‐vinyl part of the electrophile, activity is higher (compare **7 c** to **7 a**, **7 g** and **7 e**). All active cycloadducts were derived from N‐methyl maleimide. If the methyl group was replaced by a phenyl substituent, activity was lost (compare **7 c** and **7 f**). The most potent compound **7 a** inhibited Hh‐induced osteogenesis with an IC_50_ value of 1.5±0.2 μm (Figure 3a and 3b). Interestingly, **7 a** did not inhibit the orthogonal GLI‐dependent reporter gene assay in Sonic hedgehog (Shh)‐LIGHT2 cells. However, **7 a** moderately and partially suppressed the expression of the Hh target genes *Ptch1* and *Gli1* to approx. 50 % at a concentration of 5 μM (Figure 3c).

Most Hh pathway inhibitors target the seven‐pass transmembrane protein Smoothened (SMO), e.g. Vismodegib and cyclopamine, and often affect SMO ciliary localization.^[20]^ However, **7 a** did not displace the SMO binder BODIPY‐cyclopamine from SMO (Figure S2) and did not affect the localization of SMO to cilia as indicated by the co‐localization of acetylated tubulin (as a ciliary marker) and SMO (Figure 3d). Thus, **7 a** inhibits purmorphamine‐induced osteogenesis most likely without targeting SMO.

### Osteogenesis Inhibitor 7 a Targets RHOGDI1

**Figure 4:**
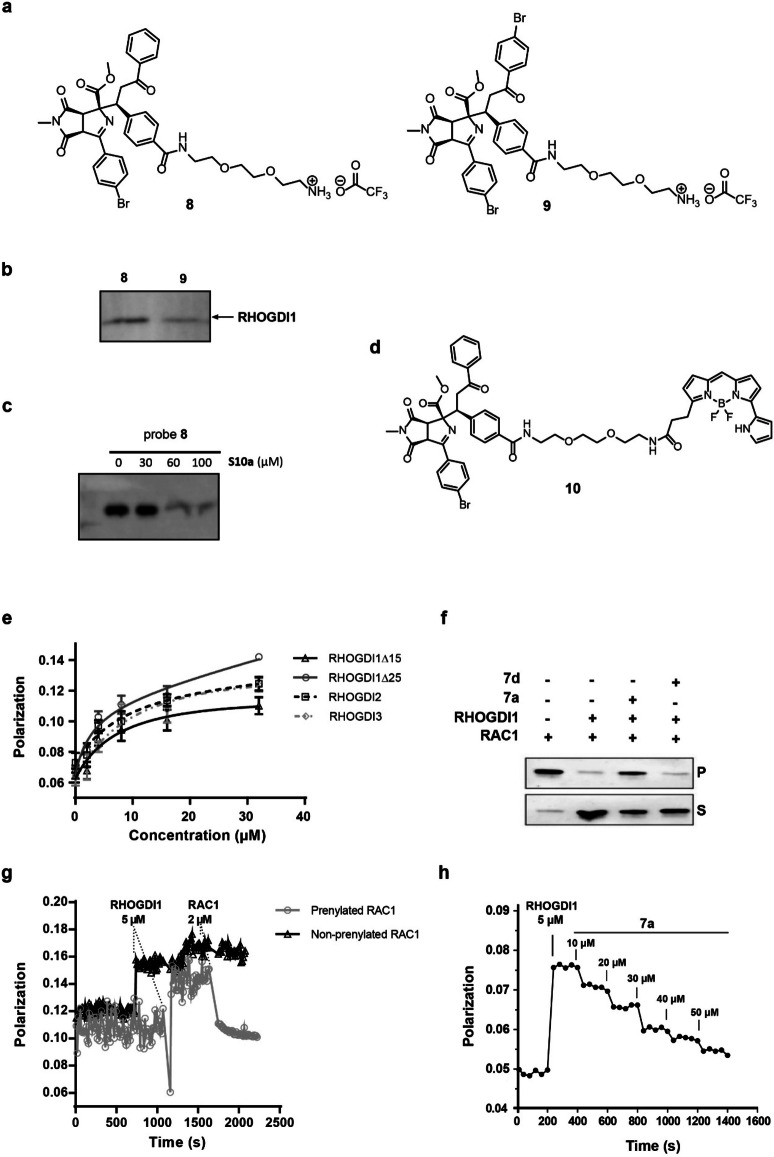
Compound **7 a** is a RHOGDI1 inhibitor. a) Structure of the affinity probes **8** and **9**. b) Affinity‐based enrichment of RHOGDI1 from NIH/3T3 lysates by probe **8** as compared to probe **9** and detection using a RHOGDI1 antibody. c) Competition pulldown was performed as in b in presence of **S10a** as a competitor. d) Structure of the fluorescent derivative **10**. e) Binding of derivative **10** to RHOGDI1‐3. *K*_D_ (RHOGDI1Δ15): 8.51 μm, *K*_D_ (RHOGDI1Δ25): 3.09 μm; *K*_D_ (RHOGDI2): 9.08 μm; *K*_D_ (RHOGDI3): 11.45 μm. Fluorescence polarization measurements using **10** and RHOGDI1‐3. Representative data (mean values±SD, *n*=3). f) Displacement of prenylated GDP‐bound RAC1 from liposomes by GST‐RHOGDI1 in the presence or absence of 50 μm **7 a** or inactive derivative **7 d** as determined using a liposome sedimentation assay. Representative data (*n*=3). P: pellet; S: supernatant. g) Competition of derivative **10** with RAC1. Fluorescence polarization measurements after adding 2 μM prenylated RAC1 or non‐prenylated RAC1 to 2 μm compound **10** and 5 μm RHOGDI1. Representative data (*n*=3). h) Fluorescence polarization measurements upon titration of **7 a** into a mixture of 5 μm FITC‐labelled GerGer‐Rab1 peptide and 50 μm RHOGDI1.

For target identification, affinity probes **8** and **9** (Figure 4a) were synthesized based on the structure–activity relationship. The corresponding Boc‐protected analogue of **8** retained significant osteogenesis inhibiting activity (**S10a**, IC_50_=12.0±1.2 μm, Table S7), whereas the Boc‐protected analogue of **9** was inactive (**S10b**, Table S7). Label‐free quantification of proteins that selectively bound to the active probe **8** as compared to the control probe **9** indicated RHO GDP‐dissociation inhibitor 1 (RHOGDI1), Filamin‐B and Filamin‐C as potential targets (Table S8). Subsequent immunoblotting after the pulldown confirmed the selective enrichment of RHOGDI1 but not of Filamin‐B and Filamin‐C (Figure 4b and Figure S3). Furthermore, excess of **S10a** prevented the enrichment of RHOGDI1 by probe **8** (Figure 4c). These findings point toward RHOGDI1 as a target of **7 a**.

RHOGDI1 is a chaperone for geranylgeranylated (GerGer) proteins, in particular the RHO GTPases.^[21]^ The major fraction (90–95 %) of prenylated RHO GTPases are maintained in a stable soluble state in the cytosol by RHOGDI1.^[22]^ **7 a** directly binds to RHOGDI1 as demonstrated for the fluorescent **7 a** derivative **10**, which displays a dissociation constant (*K*_D_) of 3.01 μm and 8.5 μm for RHOGDI1Δ15 and RHOGDI1Δ25, respectively (Figures 4d and 4e). RHOGDI1 can extract GDP‐bound inactive RHO GTPases from membranes and sequesters them in the cytosol. In an in vitro liposome sedimentation assay,^[23]^ addition of RHOGDI1 to liposomes loaded with prenylated GDP‐bound RAC1 resulted in extraction of RAC1, i.e., RAC1 was detected in the soluble fraction (Figure 4f). However, in the presence of **7 a** and RHOGDI1, RAC1 remained bound to the liposomes, i.e., RAC1 was detected in the insoluble fraction. This finding indicates that **7 a** inhibits the extraction of RAC1 by RHOGDI1. The structurally similar analog **7 d** (Figure 2a), which did not inhibit osteogenesis, also did not inhibit extraction of RAC1 from liposomes (Figure 4f). Similar results were obtained in a liposome flotation assay^[24]^ (Figures S4a and S4b). In addition to RAC1, **7 a** also inhibited the RHOGDI1‐mediated extraction of RHOA and CDC42 (Figure S4c and S4d). **7 a** slowed down the kinetics of geranylgeranylated RAC1 extraction mediated by RHOGDI1 in a surface plasmon resonance (SPR) setup using immobilized synthetic PI(4,5)P_2_‐rich liposomes loaded with geranylgeranylated GDP‐bound RAC1, whereas the inactive derivative **7 d** did not (Figures S5a–S5c). These results suggest that **7 a** may directly modulate the RHOGDI1‐RAC1 interaction. To gain insight into the binding site for **7 a** on RHOGDI1, competition between fluorescent derivative **10** and prenylated RAC1 was monitored. Addition of prenylated RAC1 to a pre‐formed **10**‐RHOGDI1 complex reduced fluorescence polarization, indicating displacement of **10** from RHOGDI1 (Figure 4g). However, non‐prenylated RAC1 was able to bind RHOGDI1 but could not displace the ligand. Moreover, increasing concentrations of **7 a** competed with the binding of RHOGDI1 to a GerGer‐RAB1 peptide, which was previously shown to bind to the prenyl‐binding pocket of RHOGDI1 (Figure 4h).^[25]^ Probe **10** also bound to RHOGDI2 and RHOGDI3 (Figure 4e). In order to determine selectivity for binding to RHOGDI, displacement of fluorescein‐labelled atorvastatin (*K*_D_=58 nm) from the prenyl‐binding pocket of the lipoprotein chaperone PDEδ was investigated.^[26]^ PDEδ preferably binds farnesylated lipoproteins like the RAS and RHEB. **7 a** does not compete with fluorescein‐labelled atorvastatin for binding to PDEδ (Figure S5d). Moreover, probe **10** binds to PDEδ only at higher concentrations (Figure S5e), thus demonstrating selectivity for RHOGDI.

Compound **7 a** exhibited limited solubility in our experiments. To enhance solubility, the phenyl rings attached to the pyrroline part of **7 a** were replaced with pyridines. For simplification of derivatization, these compounds were synthesized through an alternative two‐step protocol (see the Supporting Information and Scheme S2). The pyridine derivatives were either as potent or more potent than the original compound **7 a** in the osteogenesis assay while displaying better kinetic solubility (Table S9).

**Figure 5:**
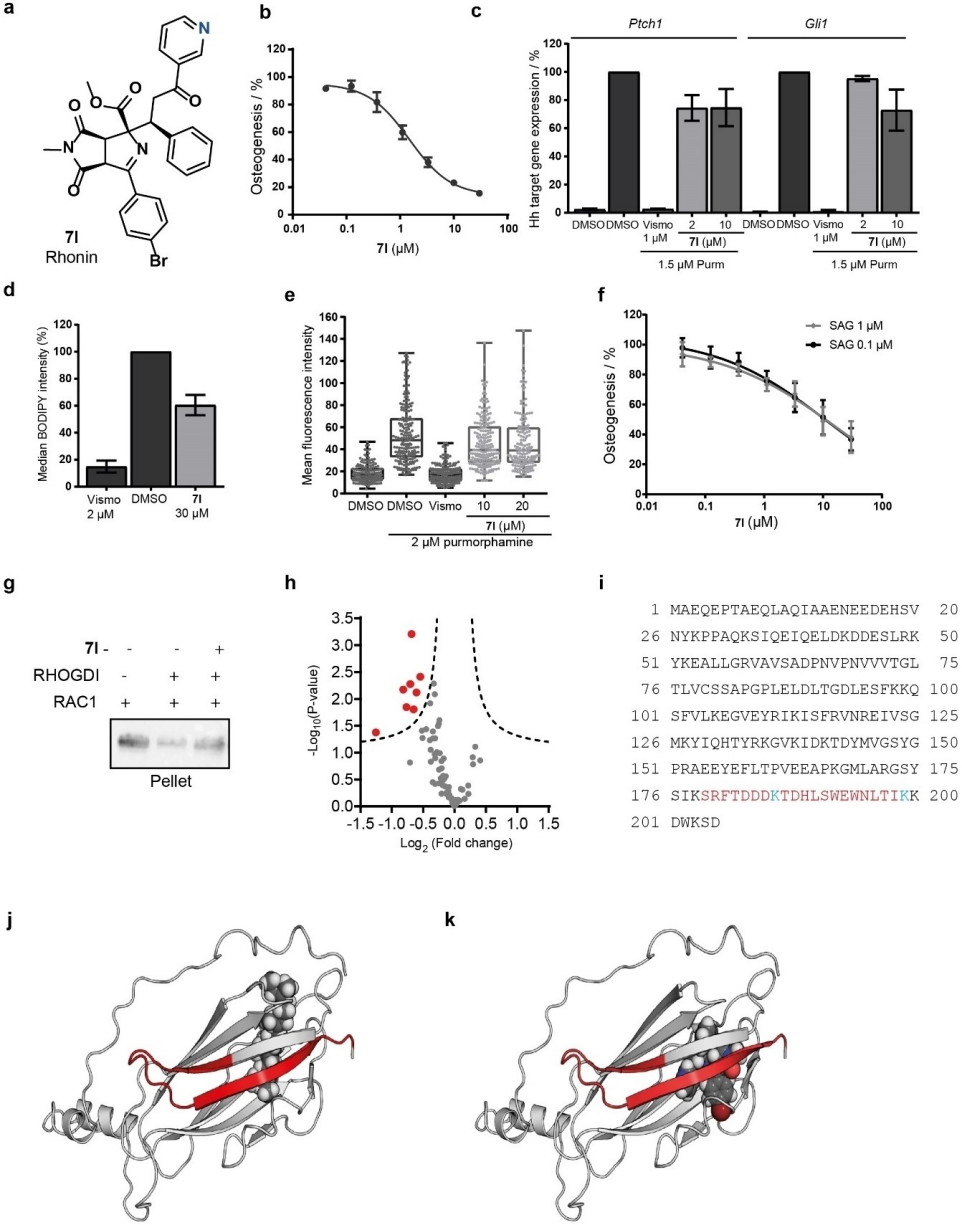
Rhonin (**7 l**) inhibits osteogenesis and binds to RHOGDI1. a) Structure of compound **7 l** termed Rhonin. b) C3H/10T1/2 cells were treated with 1.5 μm purmorphamine and compound **7 l** for 96 h prior to determination of alkaline phosphatase activity. Data are mean values±SD, *n*=3. c) C3H/10T1/2 cells were treated with purmorphamine (1.5 μm) and **7 l** or DMSO for 96 h prior to detection of the expression levels of *Ptch1*, *Gli1*, *Ap3d1* and *Gapdh* using of RT‐qPCR (mean values±SD, *n*=3). d) SMO binding assay upon treatment of cells with BODIPY‐cyclopamine followed by addition of **7 l**, Vismodegib or DMSO and quantification of SMO‐bound BODIPY‐cyclopamine using flow cytometry. e) Ciliary localization of SMO in NIH/3T3 cells. Representative results; each data point represents the intensity value of one single cilium. Statistical significance was evaluated using an unpaired t‐test with a confidence interval of 95 % (*p*≤n.s.). f) Influence of **7 l** on osteogenesis in presence of 1 μm or 0.1 μm of SAG and of **7 l** (mean values±SD, *n*=3). g) Displacement of prenylated GDP‐bound RAC1 from synthetic liposomes by GST‐RHOGDI1 in the presence or absence of 50 μm **7 l** as determined using a liposome sedimentation assay. Representative data (*n*=3). For uncropped blot see Figure S14. h) Limited proteolysis of RHOGDI1 in presence of 100 μm **7 l**. Volcano plot (FDR=0.05, S_0_=0.1) of the identified and quantified peptides of RHOGDI (≈95 % sequence coverage). i) Mapping of proteinase K‐protected peptides (amino acids 179–199) in the amino acid sequence of RHOGDI1. Protected lysines detected using the STPyne probe are shown in blue. j) and k) Mapping of proteinase K‐protected peptides in the structure of RHOGDI1 with the bound geranylgeranyl group (j) and a computationally predicted model of the RHOGDI1‐**7 l** complex (k). Red coloration: region protected from proteinase K‐mediated proteolysis in presence of compound **7 l**. The structures were prepared based on the PDB entry 1HH4.

We selected compound **7 l** (280 mg were readily obtained) for further investigations as it most potently inhibited purmorphamine‐induced osteogenesis (Figure 5a and 5b), while displaying good kinetic solubility of 47.3 μm (Table S9) and permeability (41.1 % flux in a parallel artificial membrane permeability assay; PAMPA). Similar to **7 a**, compound **7 l** did not suppress GLI‐dependent reporter gene expression and only slightly reduced the expression of the Hh target genes *Ptch1* and *Gli1* (Figure 5c and Figure S6a). However, **7 l** inhibited the expression of the alkaline phosphatase gene (*Alpl*) which is in line with suppression of osteogenesis (Figure S6b). We detected a partial decrease in BODIPY‐cyclopamine fluorescence in SMO‐expressing cells (Figure 5d). However, in the presence of **7 l** and upon stimulation with purmorphamine, SMO was localized to the cilia (Figure 5e) and cilia formation was not affected (Figure S6c). To further address putative SMO targeting by **7 l**, we explored **7 l‐**mediated suppression of osteogenesis in the presence of low and high concentrations of the SMO agonist SAG. Compounds like Vismodegib that target the heptahelical bundle of SMO, display decreased potency in the presence of high doses of SAG, which itself binds to the heptahelical bundle as well (Figure S6d).^[27]^ Derivative **7 l** displayed similar potency in the presence of 0.1 μm and 1 μm SAG and behaved like GANT61 (Figure 5f and Figure S6e). Thus, **7 l** does not appear to influence osteogenesis through modulation of SMO.

In agreement with the observations for **7 a**, compound **7 l** inhibited RHOGDI1‐mediated extraction of RAC1 from liposomes (Figure 5g). Whereas non‐prenylated TAMRA‐GDP‐RAC1 bound to RHOGDI1 with a *K*_D_ of 5.7 μm, which is in agreement with previous reports,^[28]^ in the presence of **7 l** the *K*_D_ value for the RHOGDI1‐RAC1 interaction increased to 133.3 μm, which indicates that **7 l** does interfere with RHOGDI1‐RAC1‐GDP complex formation (Figures S6f). Based on the obtained results, the measured affinity between probe **10** and RHOGDI1 is *K*_D_=3.1–8.5 μm (from Figure 4e) and is in line with the *K*_i_ for **7 l** of ca. 2.2 μm (as determined by the Cheng–Prusoff equation using data from Figures S6f). The *K*_D_ for the binding of probe **10** to RHOGDI1 might appear to be too low to interfere with the extremely high affinity GerGer‐RAC1/RHOGDI1 interaction (*K*_D_=ca. 10^−11^ M),^[29]^ and this would be true if **7 l** and GerGer‐RAC1 were competing directly for binding to RHOGDI1 in the absence of other factors. However, as shown in Figure 4f, there is a clear displacement of RAC1 from its complex with RHOGDI1 in the presence of liposomes. The reason for this is that there is already substantial competition for RAC1 binding to RHOGDI1 from the high concentration of lipids that are able to bind RAC1 with relatively high affinity, and this competition can be modified by **7 l**. As shown in Figure S7, there is a predicted displacement of RAC1 from RHOGDI1 in the micromolar to hundreds of micromolar range of **7 l** concentration. At 50 μm **7 l**, ca. 50 % of RAC1 is bound to liposomes, in approximate agreement with the results of Figure 4f and Figure 5g. Effectively, **7 l** acts as a buffer that reduces the free concentration of RHOGDI1 and this leads to the effects seen.

To map the binding site of **7 l** in RHOGDI1, we performed a limited proteolysis analysis of RHOGDI1. Mass spectrometry revealed that several peptides in the 179–199 amino acid sequence were protected from proteinase K‐mediated proteolysis in the presence of compound **7 l** (Figure 5h and i and Table S10). These proteolysis‐protected peptides are located in the protein structure adjacent to the geranylgeranyl binding site (Figure 5j and 5k). This finding suggests binding of **7 l** in the GerGer‐binding pocket. As the conformotypic peptides contained a lysine residue, we employed the lysine‐reactive probe STPyne (Figure S6g) to label lysines in a lysate of HEK293T cells expressing human RHOGDI1. In the presence of compound **7 l**, lysines 186 and 199 in RHOGDI1 were less efficiently labeled, thus indicating a limited access of STPyne to these residues due to compound binding (Figure 5i and Table S11). Furthermore, a possible binding pose and contacts of the ligand in the GerGer pocket were predicted using computational methods. Docking into the binding site was performed using an induced fit docking (IFD) methodology, which was followed by a molecular dynamics (MD) simulation with explicit waters. The COSY and NOESY 2D NMR results for **7 l** in chloroform clearly show that the imine is the preferred tautomer in solution. An enamine structure was considered for the docking as well because the solution situation may not be directly comparable to the compound in complex with the protein.^[30]^ Ab‐initio IFD methodology afforded credible poses for both imine‐like and enamine‐like tautomers of **7 l** in the flexible binding site of RHOGDI1 (Figure S8). The best poses for the two tautomers afforded satisfactory docking scores, with enamine giving somewhat better results. None of the poses generated for the imine matched with the experimentally obtained structure–activity information for the tested compounds, whereas the orientation found for the enamine‐like structure seemed to be in agreement with the physical data. The difference in the observed results could be explained by the significant geometry change of the compound core in the two tautomers (Figure S9). Therefore, further calculations and computational analysis were performed with the enamine. The ligand in the resulting pose is stabilized in the protein pocket throughout the entire MD simulation (120 ns; Figure S10 and Table S12). Notable interactions of compound **7 l** with the C‐terminal β‐strand of RHOGDI1 (Trp194, Leu196 and Ile198) were observed (Figure S11 and Video S1), thus, providing a plausible explanation for the observed increase in the proteolytic stability of the terminal protein region in the presence of **7 l**. These findings confirm RHOGDI1 as a direct target of compound **7 l**. Therefore, compound **7 l** was termed Rhonin.

### RHOGDI is a Negative Regulator of Osteogenesis

**Figure 6:**
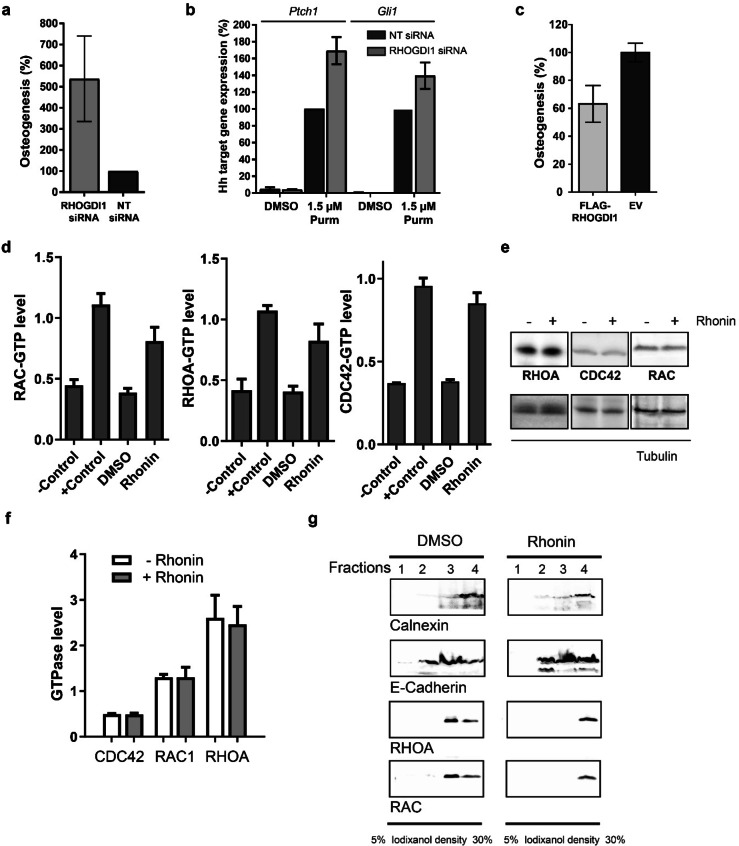
RHOGDI1 is a negative regulator of osteogenesis. a) and b) Influence of RHOGDI1 knockdown. a) Osteogenesis assay upon RHOGDI1 knockdown. NT: control siRNA (mean values±SD, *n*=3). Knockdown efficiency: 88 %. See also Figure S12a. b) *Ptch1* and *Gli1* expression upon RHOGDI1 knockdown in C3H/10T1/2 cells (mean values±SD, *n*=3). c) Influence of RHOGDI overexpression on osteogenesis (mean values±SD, *n*=3). See also Figure S12b. d) Detection of GTP‐bound RHO GTPases by means of G‐LISA upon treatment with 10 μm Rhonin for 24 h.—Control: lysis buffer;+Control: respective constitutively active GTPase (mean values±SD, *n*=3). e) and f) Influence of Rhonin (10 μM) on the total cellular levels of RHO GTPases upon treatment for 24 h detected using immunoblotting (e). Quantification of band intensities in relation to the loading control tubulin is shown in f (mean values±SD, *n*=3). g) Distribution of RHO GTPases in different cellular fractions upon treatment with Rhonin (10 μm). On a separate gel, calnexin and E‐cadherin were detected as markers for ER and plasma membrane, respectively. For uncropped blots see Figure S14.

To examine the role of RHOGDI1 in purmorphamine‐induced osteogenesis, we depleted RHOGDI1 by means of a small interfering RNA (knockdown efficiency: 88 %; Figure S12a). Purmorphamine‐mediated osteoblast differentiation was increased upon RHOGDI1 depletion using siRNA (Figure 6a). By analogy, RHOGDI1 knockdown along with simultaneous activation of the Hh pathway increased the levels of the Hh target genes *Ptch1* and *Gli1* (Figure 6b). Conversely, RHOGDI1 overexpression decreased Hh pathway activity (Figure 6c and Figure S12b). These results indicate that RHOGDI1 is a negative regulator of purmorphamine‐induced osteogenesis.

Our findings establish a link between RHOGDI1 and osteogenesis. The influence of RHO GTPases on osteoblast differentiation is cell‐ and context‐dependent and would depend on the employed system since activity of RHO GTPases will depend on different factors, i.e. phosphorylation, ubiquitination, GEFs, GAPs and possibly other effectors.^[31]^ In human mesenchymal stem cells, RHOA and ROCK were positively correlated with commitment to the osteoblast lineage.^[32]^ We detected suppression of purmorphamine‐induced osteogenesis by inhibitors of RAC or ROCK (see Table S13). RHOGDI1 modulation by Rhonin has an effect on Hh‐induced osteogenesis opposite to RHOGDI1 depletion. Such divergence between chemical and genetic perturbations has been observed before and, actually, may differentiate a chemical‐biological analysis form a genetic investigation.^[33]^ Genetic knockout or knockdown remove or reduce the target protein, whereas small molecules modulate individual binding sites or functions. RHOGDI1 recognizes its target GTPases via two binding sites. While Rhonin affects binding to the prenyl‐binding pocket, RHOGDI1 knockdown abolishes binding to both sites.

### Rhonin Activates RHO GTPases by Inhibiting RHOGDI1

Since RHOGDI1 is a regulator of RHO GTPases, small‐molecule modulator of RHOGDI1 should affect the activity of RHO GTPases. Rhonin increased the levels of GTP‐bound RHO GTPases (Figure 6d), which is in accordance with inhibition of RHOGDI1 activity. Rhonin did not alter the total levels of the three RHO GTPases (Figure 6e and 6f). Interference with RHOGDI1 function should alter the subcellular localization of RHO GTPases.^[22]^ Indeed, treatment with Rhonin led to a shift of the membrane‐bound RHO GTPases from the plasma membrane to the endoplasmic reticulum (ER) membrane (Figure 6g). Thus, upon treatment with Rhonin the amount of RHOA and RAC1 at the ER membrane increases. This finding suggests mislocalization of RHO GTPases. RHO GTPases are involved in cell migration which may depend on different RHO GTPases, cell type or stimulus.^[34]^ Therefore, we investigated the migration of NIH/3T3 cells in the presence of Rhonin using a wound healing assay. Similar to the RHOA inhibitor I (C3 toxin), Rhonin moderately inhibited wound closure and, thus, cell migration (Figure S13).

## Conclusion

We validate the “pseudo‐natural product” concept by the design, synthesis and evaluation of a compound collection that combines five‐membered N‐heterocycles (i.e. pyrrolidines, pyrrolines and succinimides) characteristic for NP classes with different structure and different biosynthetic origin, in novel arrangements and with different connectivities. The novel pseudo‐NP Rhonin proved to be an inhibitor of Hh‐induced osteogenesis but does not efficiently target canonical Hh signaling and SMO in particular and may therefore target downstream osteogenic pathways.^[35]^ In conclusion, we report the first small molecule that directly targets RHOGDI, impairs RHOGDI function as well as the activity of RHO GTPases and promises to be an invaluable tool to explore RHO GTPase‐related biology.

## Conflict of interest

G.K. is an employee of AstraZeneca, U.K.

1

## Supporting information

As a service to our authors and readers, this journal provides supporting information supplied by the authors. Such materials are peer reviewed and may be re‐organized for online delivery, but are not copy‐edited or typeset. Technical support issues arising from supporting information (other than missing files) should be addressed to the authors.

Supporting Information

Supporting Information

## Data Availability

The data that support the findings of this study are available from the corresponding author upon reasonable request.
