# Supplementary material for: The Pseudo‐Natural Product Rhonin Targets RHOGDI
Source: Angew Chem Int Ed Engl. 2022 Mar 2;61(18):e202115193. doi: 10.1002/anie.202115193 (PMC9313812; doi:10.1002/anie.202115193)
Supplement: Supplementary file 1 — Supporting Information [file ANIE-61-0-s001.pdf]

Angewandte Chemie
Eine Zeitschrift der Gesellschaft Deutscher Chemiker

Supporting Information

The Pseudo-Natural Product Rhonin Targets RHOGDI

*M. Akbarzadeh, J. Flegel, S. Patil, E. Shang, R. Narayan, M. Buchholzer, N. S. Kazemein Jasemi, M. Grigalunas, A. Krzyzanowski, D. Abegg, A. Shuster, M. Potowski, H. Karatas, G. Karageorgis, N. Mosaddeghzadeh, M.-L. Zischinsky, C. Merten, C. Golz, L. Brieger, C. Strohmann, A. P. Antonchick, P. Janning, A. Adibekian, R. S. Goody, M. R. Ahmadian, S. Ziegler, H. Waldmann**

Author Contributions

H.W. Conceptualization:Lead; Investigation:Lead; Supervision:Lead
M.A. Investigation:Equal
J.F. Investigation:Equal
S.P. Investigation:Equal; Writing – original draft:Equal
E.S. Investigation:Supporting
R.N. Investigation:Supporting
M.B. Investigation:Supporting
N.J. Investigation:Supporting
M.G. Investigation:Supporting
A.K. Investigation:Supporting
D.A. Investigation:Supporting
A.S. Investigation:Supporting
M.P. Investigation:Supporting
H.K. Investigation:Supporting
G.K. Investigation:Supporting
N.M. Investigation:Supporting
M.-L.Z. Investigation:Supporting
C.M. Investigation:Supporting
C.G. Investigation:Supporting
L.B. Investigation:Supporting
C.S. Investigation:Supporting
A.A. Supervision:Lead
P.J. Investigation:Supporting
A.A. Supervision:Lead
R.G. Investigation:Supporting
M.A. Supervision:Lead
S.Z. Investigation:Lead; Supervision:Lead

Supplementary Information

Supplementary Tables

Supplementary Table S1. Optimization of Metal-catalyst and Chiral Ligand

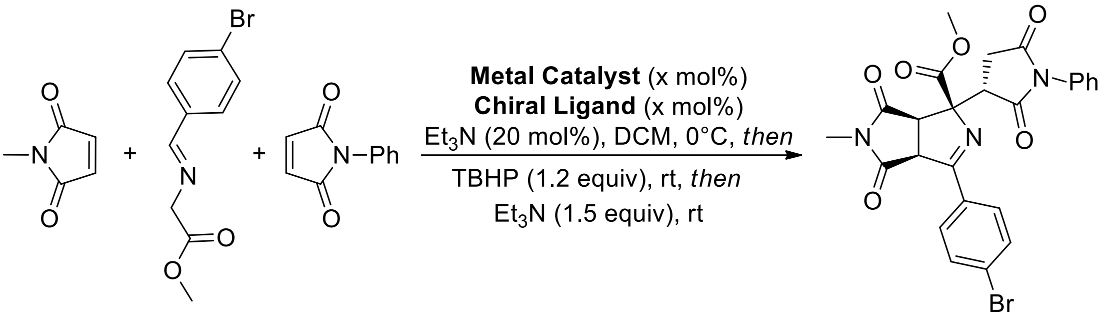

1a **2a** **1'a** **6a**

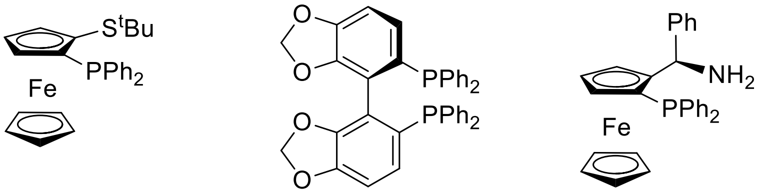

S. No.	Metal Catalyst (X mol%)	Chiral Ligand (X mol%)	Yield (%)	ee (%)
1	Cu(CH_3CN)$_4$PF_6 (5)	(*R*)-Fesulphos, **Xa** (5)	56	95
2	Cu(CH_3CN)$_4$PF_6 (5)	(*R*)-SEGPHOS, **Xb** (5)	53	24
3	Cu(CH_3CN)$_4$PF_6 (5)	**Xc**	56	22
**4**	**Cu(CH_3CN)$_4$BF_4 (5)**	**(*R*)-Fesulphos (5)**	**66**	**97**
5	Cu(CH_3CN)$_4$BF_4 (3)	(*R*)-Fesulphos (3)	60	98
6	Cu(CH_3CN)$_4$BF_4 (1)	(*R*)-Fesulphos (1)	57	97

Reaction Procedure: Required amount of Cu(I) catalyst and chiral ligand was transferred to a dry reaction vial with a stir bar and 0.5 mL of dry CH_2Cl_2 was added and stirred for 10 mins at rt. Then, the system was cooled to 0°C with ice-bath, *N*-methylmaleimide (1 equiv) and azomethine ylide (1.05 equiv) was successively added followed by the addition of Et_3N (1.5 equiv). The reaction mixture was left to stir at 0°C till the completion (0.5 – 1h) as monitored by TLC. After the completion of the cycloaddition, TBHP (5.5 M decane soln) (1.2 equiv) was

added to the reaction mixture and left to stir at rt till completion (monitored by TLC). Finally, *N*-phenylmaleimide (1.5 equiv) and Et_3N (1.5 equiv) was added and stirred at rt till completion.

Supplementary Table S2. Optimization of amount of oxidant

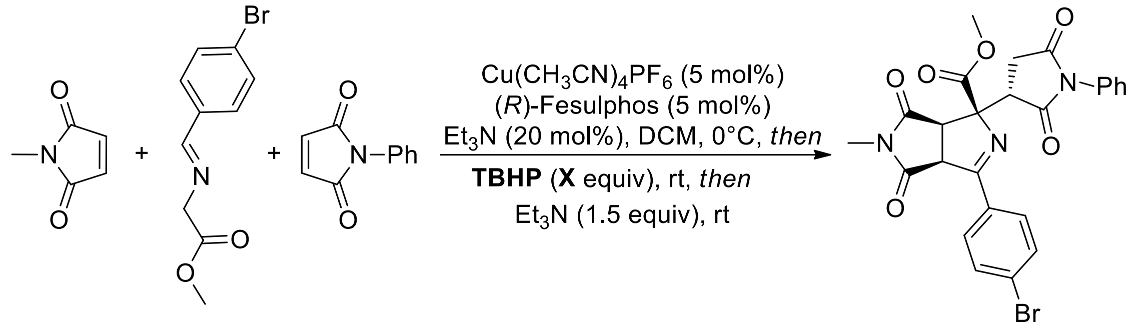

1a **2a** **1'a** **6a**

S. No.	TBHP (X equiv)	Yield (%)	ee (%)
1	1.0	55	97
2	1.2	56	97
3	1.5	65	97
4	2.0	46	97
**5**	**1.2**[a]	**69**	**97**
6	1.2[b]	48	97

[a] 70% aq. soln. of TBHP was used. [b] *N*-Phenyl maleimide was added in the second step along with TBHP.

Supplementary Table S3. Optimization of solvent for the second cycloaddition

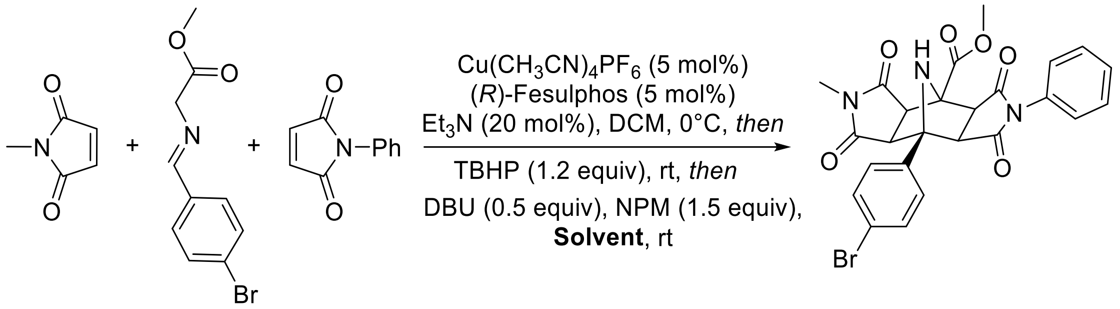

1a **2a** **1'a** **5a**

S. No.	Solvent	Yield (%)
1	Toluene	33
2	Et_2O	28
3	DME	24
**4**	**THF**	**36**

Supplementary Table S4. Optimization of base for the second cycloaddition

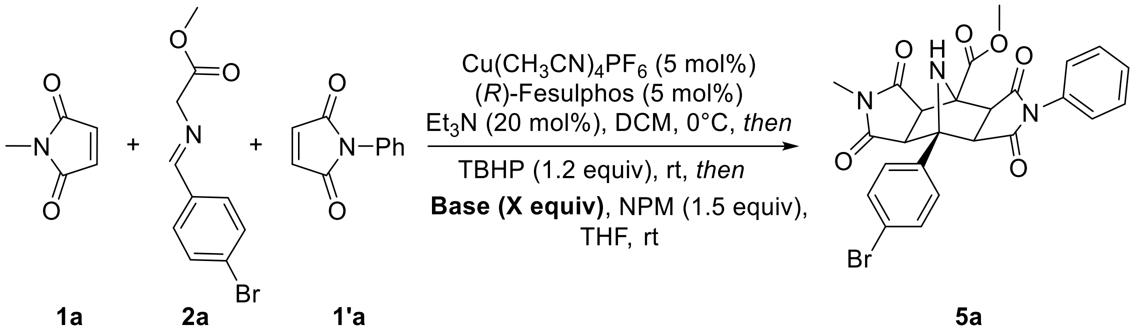

1a **2a** **1'a** **5a**

S. No.	Solvent (equiv)	Yield (%)
1	Et_3N (1.5)	33
2	DBU (0.25)	26
3	TBD (0.5)	26
4	DBU (0.5)	34
5	DBU (1.0)	23
**6**[a]	**DBU (0.5)**	**43**

[a] TBHP (70% aq.) was used. DBU: 1,8-Diazabicyclo[5,4,0]undec-7-ene, TBD: 1,5,7-Triazabicyclo[4.4.0]dec-5-ene

Supplementary Table S5. Further Optimization of reaction conditions

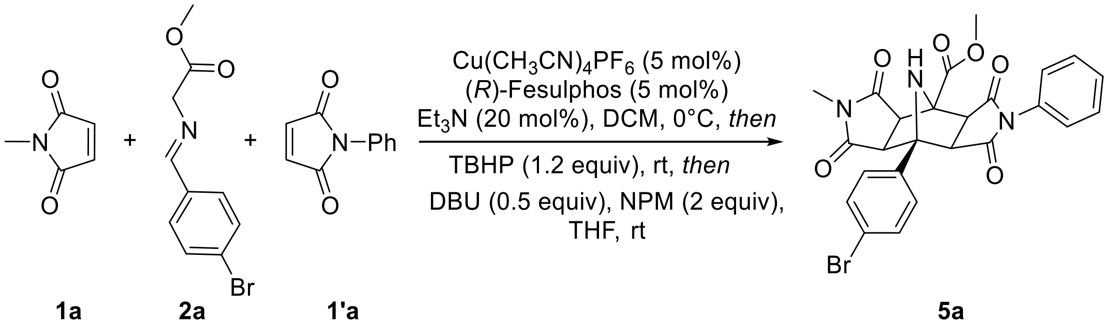

S. No.	Conditions	Yield (%)
**1**	**NPM added dropwise as solution in THF**	**48**
2	NPM added via syringe pump over 6h	30
3	DBU added dropwise as solution	39

Supplementary Table S6. Range of molecular properties calculated for the reported pseudo-NPs and literature suggested range values for each property.

	MW	HBA	HBD	tPSA	Fsp3	LogP
**Maximum**	650.0	9	1	139.4	0.43	5.21
**Minimum**	434.0	6	0	93.1	0.21	0.23
**Average**	526.9	7.0	0.3	108.7	0.31	2.34
**Literature Suggested Range**	150 - 500[1]	< 10[1]	< 5[1]	< 140	0.3 – 0.6[2]	0 - 5[1]

Supplementary Table S7. Inhibition of osteogenesis assay. C3H10T1/2 cells were incubated with 1.5 µM purmorphamine and compounds or DMSO as a control for 96 h. The activity of alkaline phosphatase was assessed by means of a luminescence readout. Values for cells, which were treated with DMSO and purmorphamine, were set to 100% and data were measured in triplicates. Compounds are marked inactive when no inhibition was observed at the highest test concentration of 30 µM. All data are mean values of three independent experiments (n = 3) ± SD.

**Compound**	**Osteogenesis IC_{50} (µM)**
**5a**	inactive
**5b**	inactive
**5c**	inactive
**5d**	inactive
**5e**	inactive
**5e**	inactive
**5f**	inactive
**6a**	inactive
**6b**	inactive
**6c**	inactive
**6d**	inactive
**6e**	inactive
**7a**	1.5 ± 0.2
**7a-epi**	1.8 ± 0.0
**7b**	inactive
**7c**	6.2 ± 1.3
**7d**	inactive
**7e**	2.81 ± 0.4
**7f**	inactive
**7g**	3.13 ± 0.2
**7h**	inactive
**7i**	inactive
**S10a**	12.9 ± 0.9
**S10b**	inactive

Supplementary Table S8. List of proteins enriched by the active probe 8 as identified by means of affinity chromatography. Active probe **8** and inactive probe **9** were immobilized on NHS ester magnetic beads. NIH/3T3 lysate was passed on to the probes and target protein identification was performed employing HRMS analysis as described in the methods section. The ratios indicate the fold excess of protein enriched with the active probe **8** as compared to the inactive *pull-down* probe **9**. Apart from filamin –B and –C and RHOGDI1, all enriched proteins were known frequent binders in pulldown assays.

**Protein name**	**Gene symbol**	**Ratio**
Filamin-B	Flnb	36
**RHO GDP-dissociation inhibitor 1**	**Arhgdia**	**33**
Filamin-C	Flnc	27
60S ribosomal protein L32	Rpl32	9
Guanine nucleotide-binding protein subunit beta-2-like 1;Guanine nucleotide-binding protein subunit beta-2-like 1, N-terminally processed	Gnb2l1	6
ATPase inhibitor, mitochondrial	Atpif1	4
Hsc70-interacting protein	St13	4
Tropomyosin alpha-4 chain	Tpm4	4
Ribosome-recycling factor, mitochondrial	Mrrf	3

Supplementary Table S9. Analogues with improved solubility as compared to compound 7a. The activities in the purmorphamine-induced osteoblast differentiation assay (ODA) are shown (mean values, n=3 ± SD).

Compound number	Structure	ODA IC_{50} ± SD (µM)	Kinetic solubility (µM)
**7a**	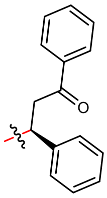	1.3 ± 0.3	5.7
**7j**	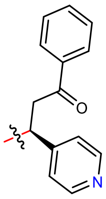	1.59 ± 0.8	56.5
**7k**	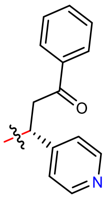	1.43 ± 0.51	34.1
**7l**	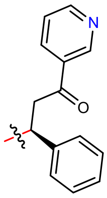	1.52 ± 0.22	47.3
**7m**	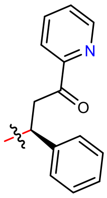	3.9 ± 1.13	14.8

Compound number	Structure	ODA IC_{50} ± SD (μM)	Kinetic solubility (μM)
**7n**	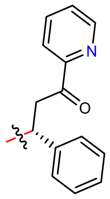	1.64 ± 0.56	29.0

Supplementary Table S10. List of RHOGDI peptides identified by limited proteolysis (LiP)-MS. RHOGDI was treated with 100 µM Rhonin or DMSO prior to digestion.

Sequence	Amino acid before	Last amino acid	Start position	End position	Significant	Log2 fold change Rhonin-100 µM/DMSO	-LOG (P-value)
FQGAMGGSA	Y	A	7	15		-0.35	1.39
MGGSAENEEDEHSVNYKPPAQK	A	K	11	32		0.22	0.39
MGGSAENEEDEHSVNYKPPA	A	A	11	30		0.08	0.11
ENEEDEHSVNYKPPA	A	A	16	30		-0.19	0.52
NEEDEHSVNYKPPA	E	A	17	30		-0.19	0.39
NEEDEHSVNYKPPAQK	E	K	17	32		-0.24	0.43
QKSIQEIQELDKDDESLRK	A	K	31	49		-0.13	0.23
SIQEIQELDK	K	K	33	42		-0.19	0.54
SIQEIQELDKDDESLR	K	R	33	48		-0.18	0.57
SIQEIQELDKDDESL	K	L	33	47		0.00	0.01
SIQEIQELDKDDESLRKY	K	Y	33	50		-0.34	1.26
SIQEIQELDKDDESLRK	K	K	33	49		0.02	0.12
SIQEIQELDKDDE	K	E	33	45		-0.03	0.06
QEIQELDKDDESLR	I	R	35	48		-0.38	1.05
QEIQELDKDDESLRK	I	K	35	49		-0.18	0.51
IQELDKDDESLRK	E	K	37	49		-0.14	0.56
IQELDKDDESLR	E	R	37	48		-0.01	0.03
QELDKDDESLRK	I	K	38	49		-0.21	0.24
LDKDDESLRK	E	K	40	49		-0.08	0.21
DKDDESLRK	L	K	41	49		-0.28	1.01
DKDDESLRKY	L	Y	41	50		0.02	0.06
DKDDESLR	L	R	41	48		-0.39	1.92
KYKEALLGR	R	R	49	57		-0.51	1.27
YKEALLGRV	K	V	50	58		-0.31	0.42
YKEALL	K	L	50	55		0.16	0.14
YKEALLGR	K	R	50	57		-0.24	0.99
EALLGR	K	R	52	57		-0.03	0.11
VAVSADPNVPNVVVTGL	R	L	58	74		0.10	0.33

Sequence	Amino acid before	Last amino acid	Start position	End position	Significant	Log2 fold change Rhonin-100 µM/DMSO	-LOG (P-value)
VAVSADPNVPNVVVT	R	T	58	72		0.04	0.07
AVSADPNVPNVVVTGL	V	L	59	74		-0.09	0.36
AVSADPNVPNVVVT	V	T	59	72		-0.31	0.97
VSADPNVPNVVVTGLTL	A	L	60	76		-0.04	0.11
VSADPNVPNVVVTGL	A	L	60	74		0.06	0.12
SADPNVPNVVVTGL	V	L	61	74		-0.17	0.89
PNVVVT	V	T	67	72		-0.71	0.82
PGPLELDLTGDLESFK	A	K	82	97		0.33	1.11
ELDLTGDLESFKK	L	K	86	98		0.22	0.23
DLTGDLESFKK	L	K	88	98		-0.07	0.16
KKQSFVLKEGVEYR	F	R	97	110		-0.15	0.37
KQSFVLKEGVEYR	K	R	98	110		0.03	0.07
QSFVLK	K	K	99	104		-0.19	0.48
QSFVLKEGVEYR	K	R	99	110		-0.26	1.50
KEGVEYR	L	R	104	110		-0.09	0.17
IKISFRVNREIVSGMK	R	K	111	126		-0.07	0.17
ISFRVNREIVSGMK	K	K	113	126		-0.10	0.23
VNREIVSGMKYIQHTYR	R	R	117	133		0.29	0.79
VNREIVSGMK	R	K	117	126		-0.25	1.54
NREIVSGMK	V	K	118	126		-0.32	2.09
EIVSGMKYIQHTYRK	R	K	120	134		0.41	0.86
EIVSGMK	R	K	120	126		-0.35	0.45
YIQHTYRK	K	K	127	134		-0.31	0.90
KGVKIDKT	R	T	134	141		-0.39	1.23
KGVKIDKTDYMVGSY	R	Y	134	148		-0.52	1.41
KGVKIDKTDYMVGSYGPR	R	R	134	151		-0.03	0.07
GVKIDKTDYMVGSY	K	Y	135	148		-0.38	0.83
GVKIDKTDYMVGSYGPRAE	K	E	135	153		-0.17	0.51
GVKIDKTDY	K	Y	135	143		-0.24	1.61

Sequence	Amino acid before	Last amino acid	Start position	End position	Significant	Log2 fold change Rhonin-100 µM/DMSO	-LOG (P-value)
GVKIDKTDYMVGSYGPR	K	R	135	151		-0.05	0.15
IDKTDYMVGSYGPRAE	K	E	138	153		-0.45	1.44
IDKTDYMVGSY	K	Y	138	148		-0.22	0.52
IDKTDYMVGSYGPR	K	R	138	151		0.04	0.07
DKTDYMVGSYGPR	I	R	139	151		-0.01	0.03
TDYMVGSYGPR	K	R	141	151		-0.10	0.29
DYMVGSYGPR	T	R	142	151		-0.21	0.84
MVGSYGPR	Y	R	144	151		-0.25	0.68
GSYGPR	V	R	146	151		-0.11	0.23
EYEFLTPVEEAPK	E	K	154	166		-0.33	2.29
YEFLTPVEEAPK	E	K	155	166		-0.21	0.71
EFLTPVEEAPK	Y	K	156	166		-0.31	1.27
FLTPVEEAPK	E	K	157	166		-0.09	0.21
GSYSIKSR	R	R	172	179		0.02	0.04
SRFTDDDKTDHLSWEW	K	W	178	193	+	-0.61	2.12
SRFTDDDKTDHLSW	K	W	178	191	+	-0.69	3.21
SRFTDDDKTDHL	K	L	178	189	+	-0.65	1.81
FTDDDKTDHLSWEWNL	R	L	180	195	+	-0.71	2.28
FTDDDKTDHLSW	R	W	180	191	+	-0.82	2.17
TDDDKTDHL	F	L	181	189	+	-1.25	1.38
TDHLSWEWNLTIKK	K	K	186	199		0.12	0.31
TDHLSWEWNLTIK	K	K	186	198		0.29	0.91
SWEWNLTIKK	L	K	190	199	+	-0.77	1.85
SWEWNLTIK	L	K	190	198	+	-0.55	2.41
TIKKDWKD	L	D	196	203		-0.13	0.25

Supplementary Table S11: Extracted list of STPyne PC cleaved RHOGDI peptides identified by catch-and-release proteomics. HEK293T lysate overexpressing RHOGDI-F was treated with 100 µM Rhonin or DMSO, followed by 30 µM STPyne. Asterisks indicate modified lysines.

Sequence	Modified lysine position	Protein names	Average ratio intensity STPyne-Rhonin-100 µM/DMSO	SD ratio intensity STPyne-Rhonin-100 µM/DMSO
FTDDDKTDHLSWEWNLTIK*K	199	Rho GDP-dissociation inhibitor 1	0.59	0.02
FTDDDK*TDHLSWEWNLTIK	186	Rho GDP-dissociation inhibitor 1	0.60	0.06
QSFVLK*EGVEYR	105	Rho GDP-dissociation inhibitor 1	0.86	0.04
EIVSGMK*YIQHTYRK	127	Rho GDP-dissociation inhibitor 1	0.87	0.05
GSYSIK*SR	178	Rho GDP-dissociation inhibitor 1	0.88	0.03
GVK*IDKTDYMVGSYGPR	138	Rho GDP-dissociation inhibitor 1	0.90	0.14
AEEYEFLTPVEEAPK*GMLAR	167	Rho GDP-dissociation inhibitor 1	0.92	0.11
IDK*TDYMVGSYGPR	141	Rho GDP-dissociation inhibitor 1	0.97	0.14
IK*ISFR	113	Rho GDP-dissociation inhibitor 1	1.04	0.04
SIQEIQELDK*DDESLR	43	Rho GDP-dissociation inhibitor 1	1.05	0.09
YK*EALLGR	52	Rho GDP-dissociation inhibitor 1	1.08	0.08
K*QSFVLKEGVEYR	99	Rho GDP-dissociation inhibitor 1	1.16	0.13
YIQHTYRK*	135	Rho GDP-dissociation inhibitor 1	1.45	0.41

Supplementary Table S12. Quality analysis results for the Rhonin-RHOGDI1 MD simulation (0-120 ns). The MD studies were performed in duplicate. The values are given as mean ± standard deviation.

Replicate	Total energy (kcal/mol)	Potential energy (kcal/mol)	Temperature (K)	Pressure (bar)	Volume (Å^3)
1	-118023.6 ± 93.8	-143159.8 ± 80.4	298.7 ± 0.6	0.92 ± 45	421168.8 ± 381.7
2	-117802.2 ± 93.2	-142888.8 ± 79.9	298.7 ± 0.6	1.05 ± 45	420301.2 ± 383.1

Supplementary Table S13. Influence of Rac, Cdc42 and ROCK inhibitors on osteoblast differentiation. C3H10T1/2 cells were incubated with 1.5 µM purmorphamine and compounds or DMSO as a control for 96 h. The activity of alkaline phosphatase was assessed by means of a luminescence readout. Values for cells, which were treated with DMSO and purmorphamine, were set to 100% and data were measured in triplicates. Data are mean values of three independent experiments (n = 3) ± SD.

**Compound**	**Activity**	**Osteogenesis**<br>**IC_{50} ± SD (µM)**
ZCL278	CDC42 GTPase inhibitor	75% activity at 2 µM
EHop-016	RAC GTPase inhibitor	0.881 ± 0.08
EHT 1864	RAC GTPase inhibitor	> 2
GSK534911A	ROCK 1 inhibitor	1.55 ± 0.5
SR-3677	ROCK 2 inhibitor	1.72 ± 0.1
H-1152	ROCK inhibitor	2.87 ±0.0

Supplementary Schemes

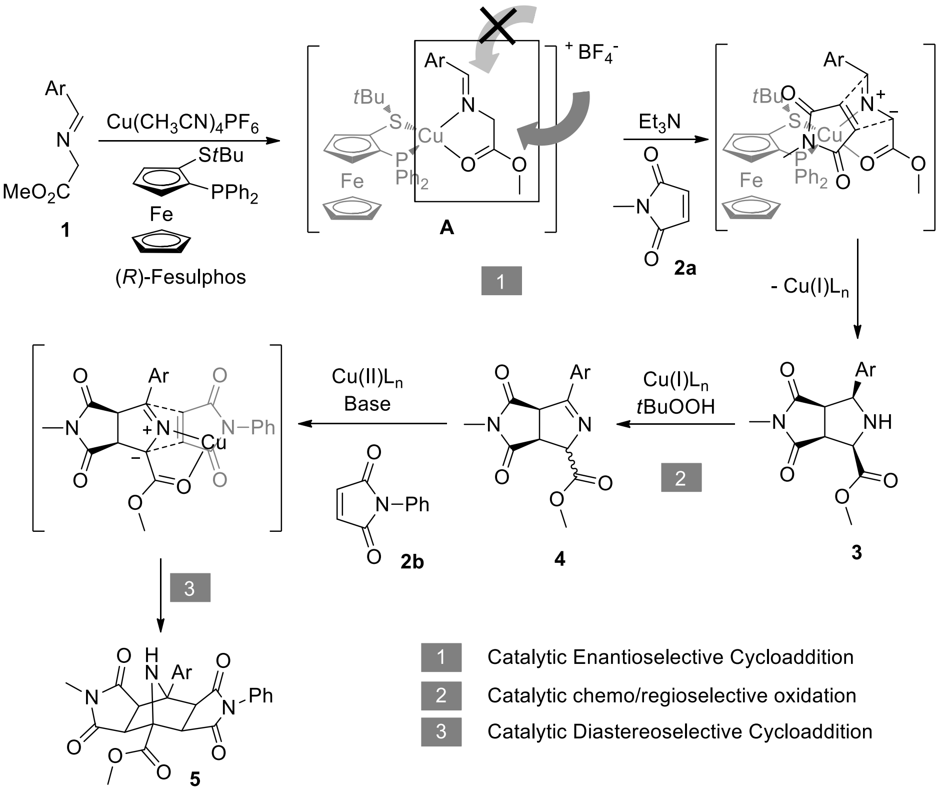

Scheme S1. Proposed mechanism and stereoselection for the enantioselective double [3+2] cycloaddition reaction.

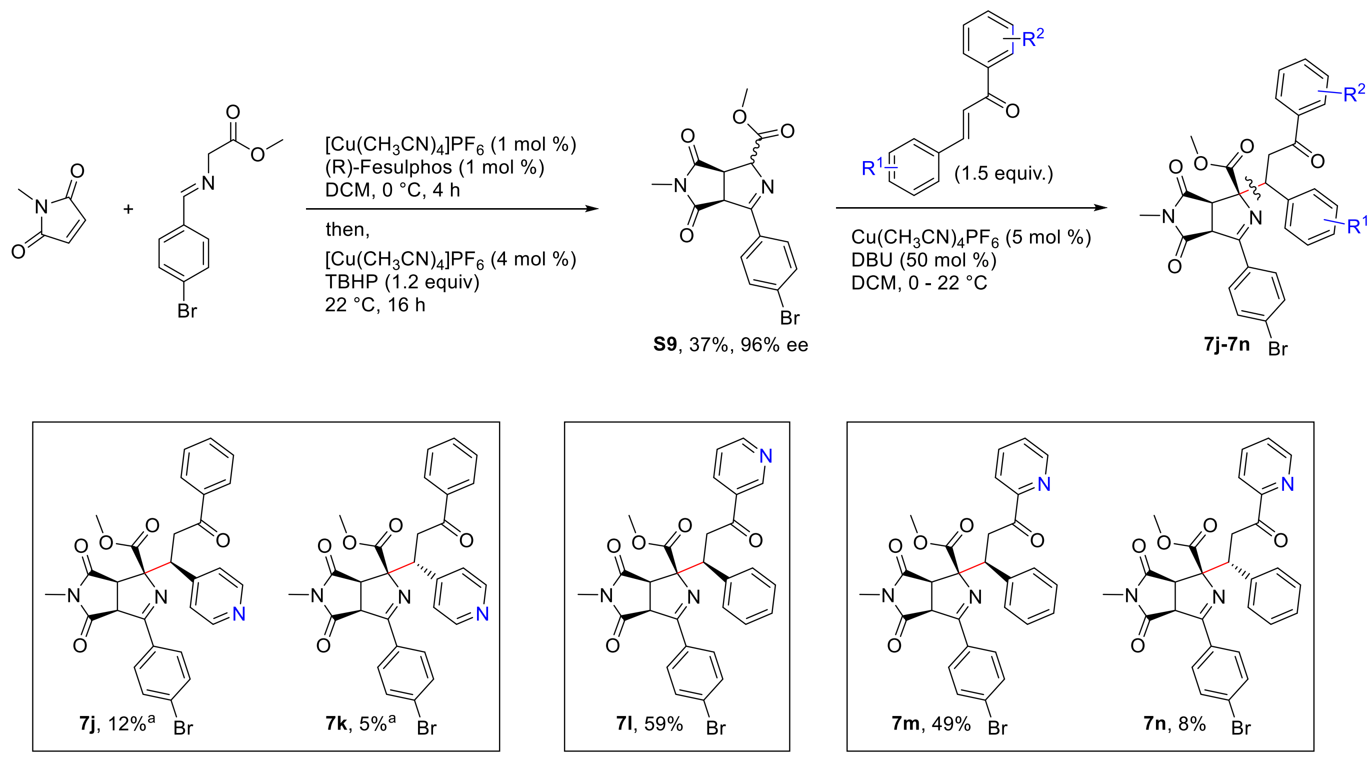

Scheme S2. Two pot synthesis of pyridine-containing compounds **7j-7n**. The compounds shown inside of the boxes are the diastereomer(s) that could be isolated from a single reaction. [a]Isolated as the TFA salt after preparative HPLC purification.

Supplementary Figures

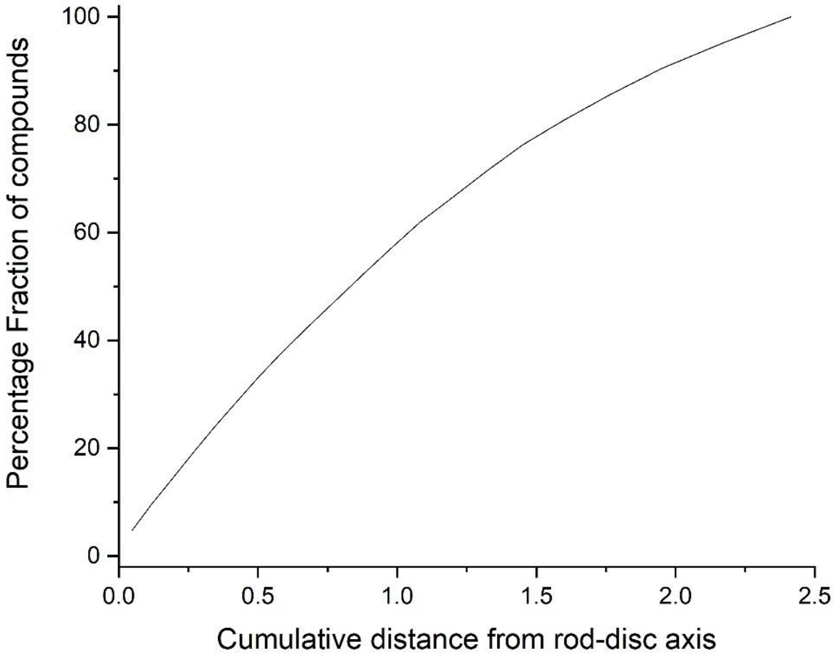

Supplementary Figure S1. Cumulative distance from the rod-disc axis of the PMI plot against the Percentage Fraction of compounds. The cumulative distance increases in a non-linear fashion demonstrating the shape diversity of compound collection. The cumulative distance for all compounds was calculated to 2.41 units, and the average distance was calculated to 0.12.

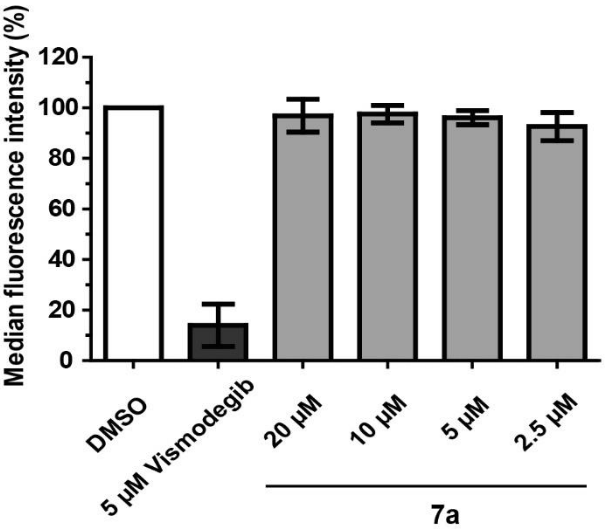

Supplementary Figure S2. 7a does not displace Bodipy-cyclopamine. HEK293T cells ectopically expressing SMO were treated with different concentrations of **7a** or Vismodegib and DMSO as controls in the presence of BODIPY-cyclopamine (5 nM) for 5 h. The graph shows the percentage of cell-bound BODIPY-cyclopamine as detected by fluorescence-activated cell sorting analysis. Data are mean values of three independent experiments (n=3) ± SD.

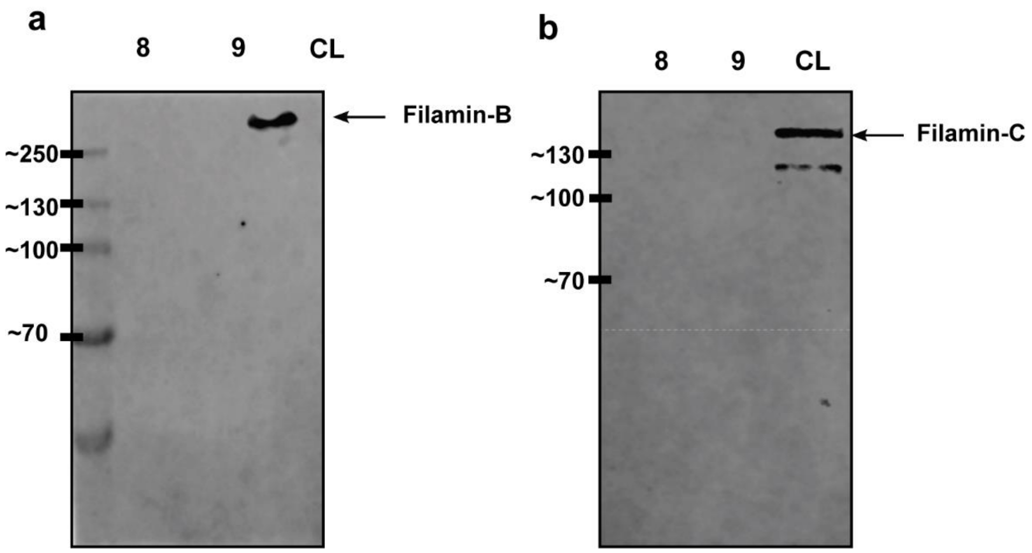

Supplementary Figure S3. Affinity-based chromatography. Active probe **8** and inactive probe **9** were immobilized on NHS ester magnetic beads and exposed to NIH/3T3 lysates. Bound proteins were eluted and analysed by immunoblotting using specific antibodies against Filamin-B (**a**) or Filamin-C (**b**). Representative images of two independent experiment are shown. CL: Cell lysate.

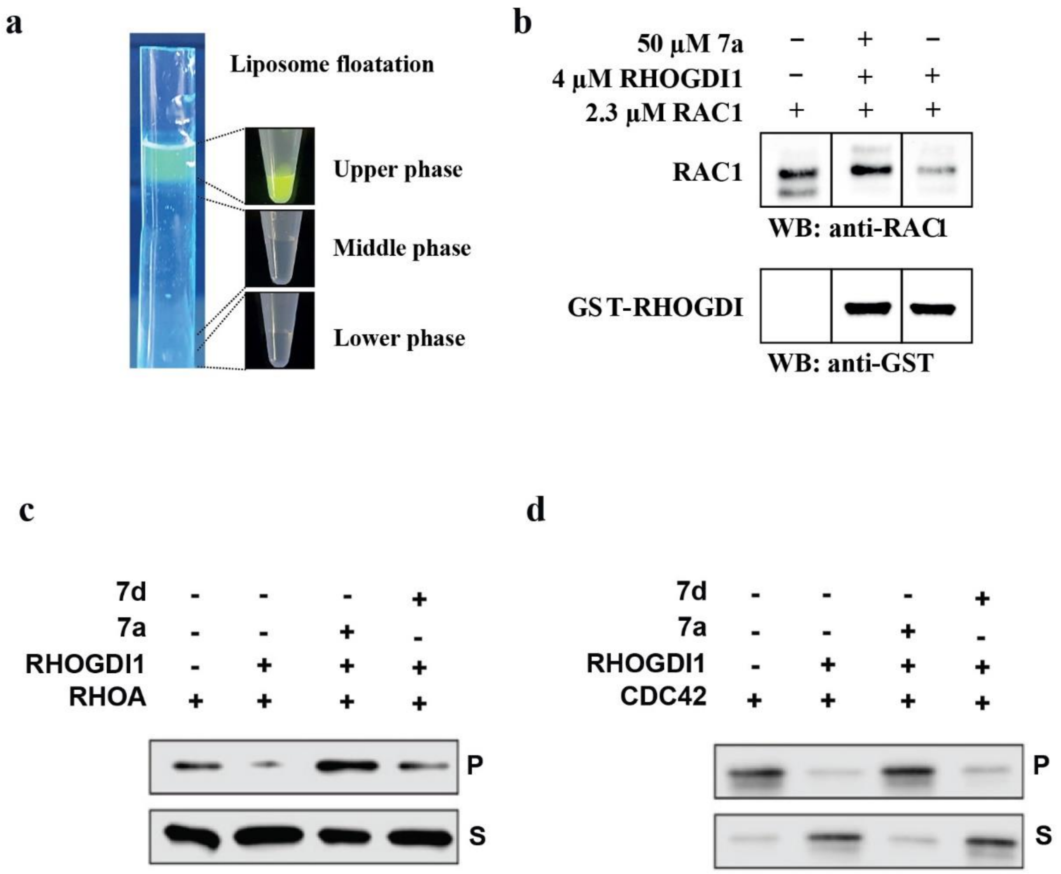

Supplementary Figure S4. Compound 7a impairs RHOGDI1 function. (**a**) Assay setup for the liposome sedimentation assay. (**b**) Displacement of prenylated GDP-bound RAC1 from synthetic liposomes by GST-RHOGDI1 in the presence or absence of 50 µM **7a** was measured using liposome floatation assay. Data is representative of three independent experiments. Liposome-containing phase represented by fluorescent colour as result of 5% fluorescent NDB-PE was analysed by Western blotting. Data are representatives of three independent experiments. The uncropped blots are shown in Figure S14. (**c-d**) Displacement of prenylated GDP-bound RHO GTPases (RHOA and CDC42) from synthetic liposomes by GST-RHOGDI1 in the presence or absence of 50 µM **7a** or inactive derivative **7d** was determined using a liposome sedimentation assay. Data is representative of three independent experiments. P: pellet; S: supernatant.

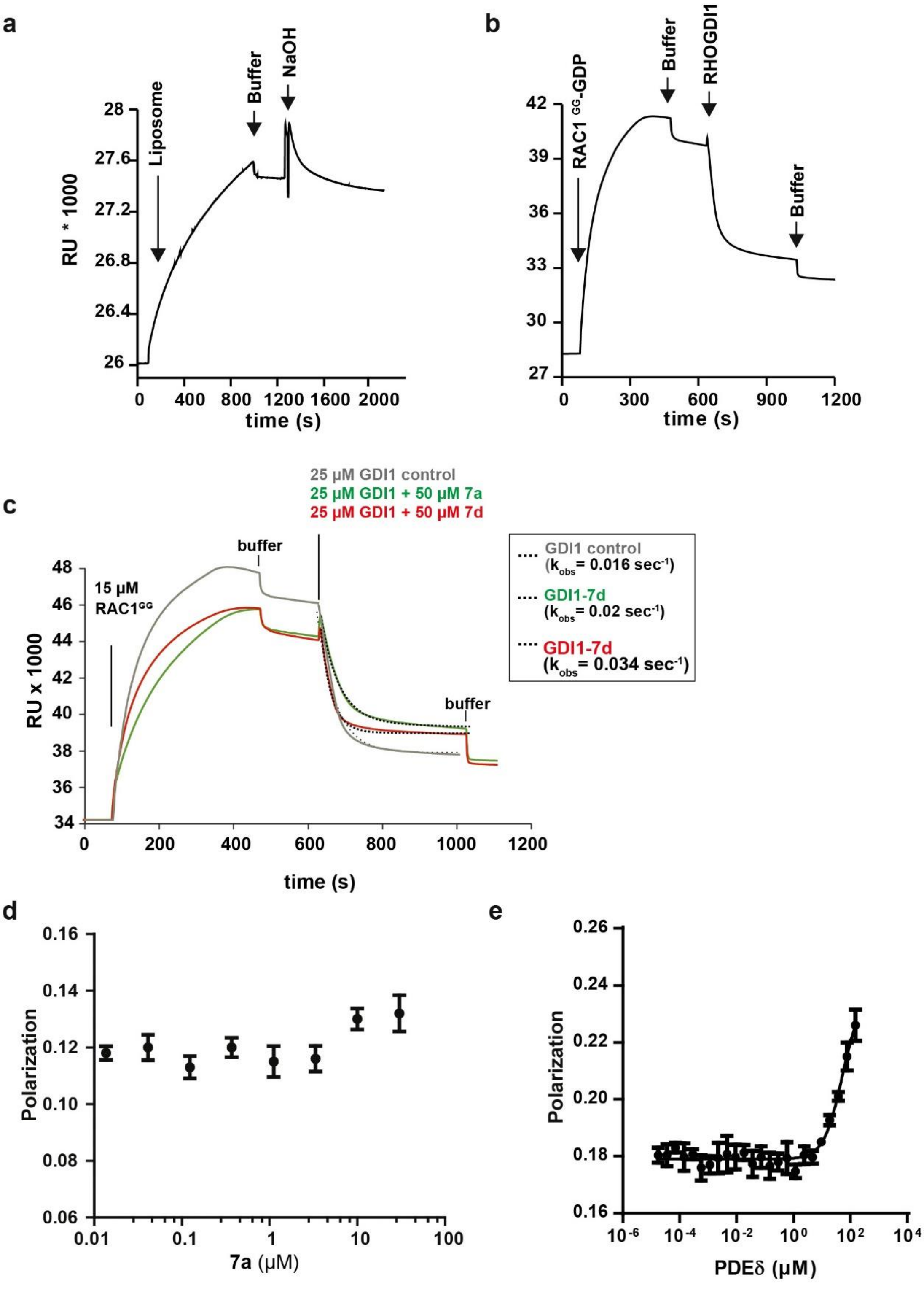

Supplementary Figure S5. Compound 7a binds to RHOGDI1 (**a-c**) Surface plasmon resonance measurements. (**a**) Synthetic PI(4,5)P_2-rich liposomes were immobilized on the surface of L1 sensor chip. (**b**) Geranylgeranylated, GDP-bound RAC1 (RAC1GG–GDP) was loaded on the liposome-immobilized L1 sensor chip followed by addition of RHOGDI1. (**c**)

RHOGDI1 displaced liposome -bound RAC1 from the surface of L1 chip with a k_{off} of 0.016 s^{-1}. Addition of a mixture of 25 μM RHOGDI1 and **7a** or the inactive derivative **7d** to liposome-bound RAC1. Calculated rate constants by mono-exponential fitting of RU decays were 0.02 and 0.032 s^{-1} for RHOGDI1-mediated RAC1 displacement from the liposomes in the presence of **7a** and **7d**, respectively. (**d**) Competition-based fluorescence polarization experiments were performed by titration of **7a** against 10 nM fluorescein-labelled atorvastatin and GST-PDEδ. (**e**) Fluorescence polarization experiment using 10 nM probe **10** and titration of PDEδ.

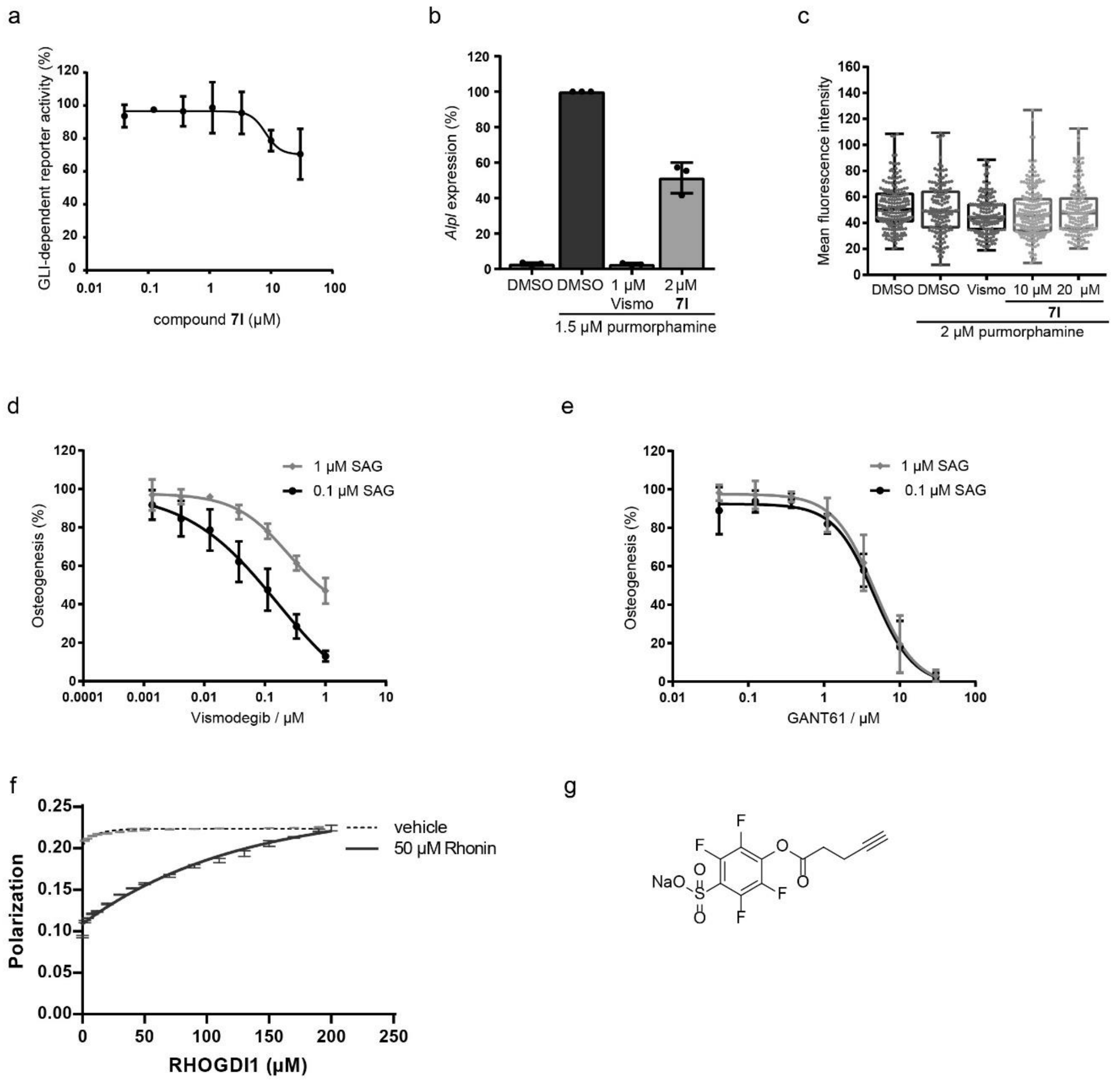

Supplementary Figure S6 (related to Figure 5). Characterization of compound **7l** (Rhonin). (**a**) GLI-responsive reporter gene assay in SHH-LIGHT2 cells. (**b**) Influence of Rhonin on the expression of *Alpl*. C3H10T1/2 cells were treated with 1.5 µM purmorphamine and the compounds for 96 h prior to RT-qPCR to detect *Alpl* mRNA levels. Data were normalzed to *Gapdh* and *Ap3d1* gene levels and are mean values (n = 3) ± SD. (**c**) Detection of cilia in NIH/3T3 cells. NIH/3T3 cells were cultured in serum reduced (0.5 % FCS) medium for 24 h to induce cilia formation, followed by a pre-treatment of cells with different concentrations of **7l** or Vismodegib or DMSO as a control in serum reduced medium. After another 24h cells were treated with 2 µM purmorphamine or DMSO as a control and with different concentrations of **7l**, or Vismodegib or DMSO as a control for 24 h. Cells were then fixed and

cilia were stained with an antibody against acetylated tubulin. SMO was marked with an anti-SMO antibody and DAPI was used to stain the nuclei. For quantification of cilia, at least 100 cilia per condition and biological replicate were analyzed for the intensity of anti-acetylated tubulin antibody staining. The results of one representative biological replicate are shown and each data point represents the intensity value of one single cilium. Statistical significance was evaluated using an unpaired t-test with a confidence interval of 95 % (p ≤ n.s.). (**d** and **e**) Influence Vismodegib (d) or GANT61 (e) on osteoblast differentation of C3H10T1/2 cells in presence of low (0.1 µM) or high (1 µM) concentration of the SMO agonist SAG. Data are mean values of three independent biological replicates (n=3) ± SD. (**f**) Fluorescent polarization measurements were performed by titrating 1 µM TAMRA-GDP-bound RAC1 with increasing concentrations of RHOGDI1 in the absence (K_D = 5.7 µM) and presence (K_D = 133 µM) of 50 µM Rhonin. Data are mean values (n = 3) ± SD. (**g**) Structure of the lysine-reactive probe STPyne.

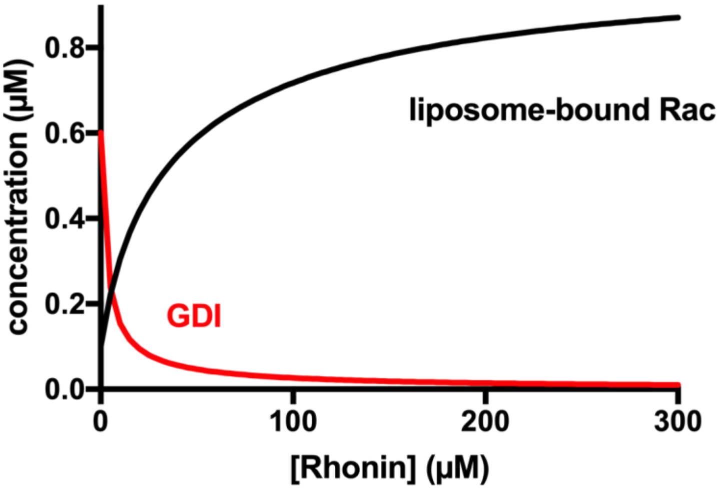

Supplementary Figure S7. Simulation of a titration of Rhonin into a system containing GerGer-RAC1 (1 µM), RHOGDI1 (1.5 µM) and liposomes (1.35 mM surface exposed concentration of lipids) under the conditions of Figure 4f and Figure 5g. K_D values used: RHOGDI1:GerGer-RAC1 10^{-11} M; liposome:GerGer-RAC1 0.2 µM; RHOGDI1:Rhonin 2.2 µM, and an effective K_D for binding of a geranylgeranyl group to liposomes of 0.2 µM (defined with respect to the total exposed lipid concentration[3]). Black curve shows the increase of concentration of liposome bound GerGer-RAC1, the red curve shows the decrease in concentration of free RHOGDI1. The simulations were performed with KinTek Explorer (http://kintekcorp.com/software/).

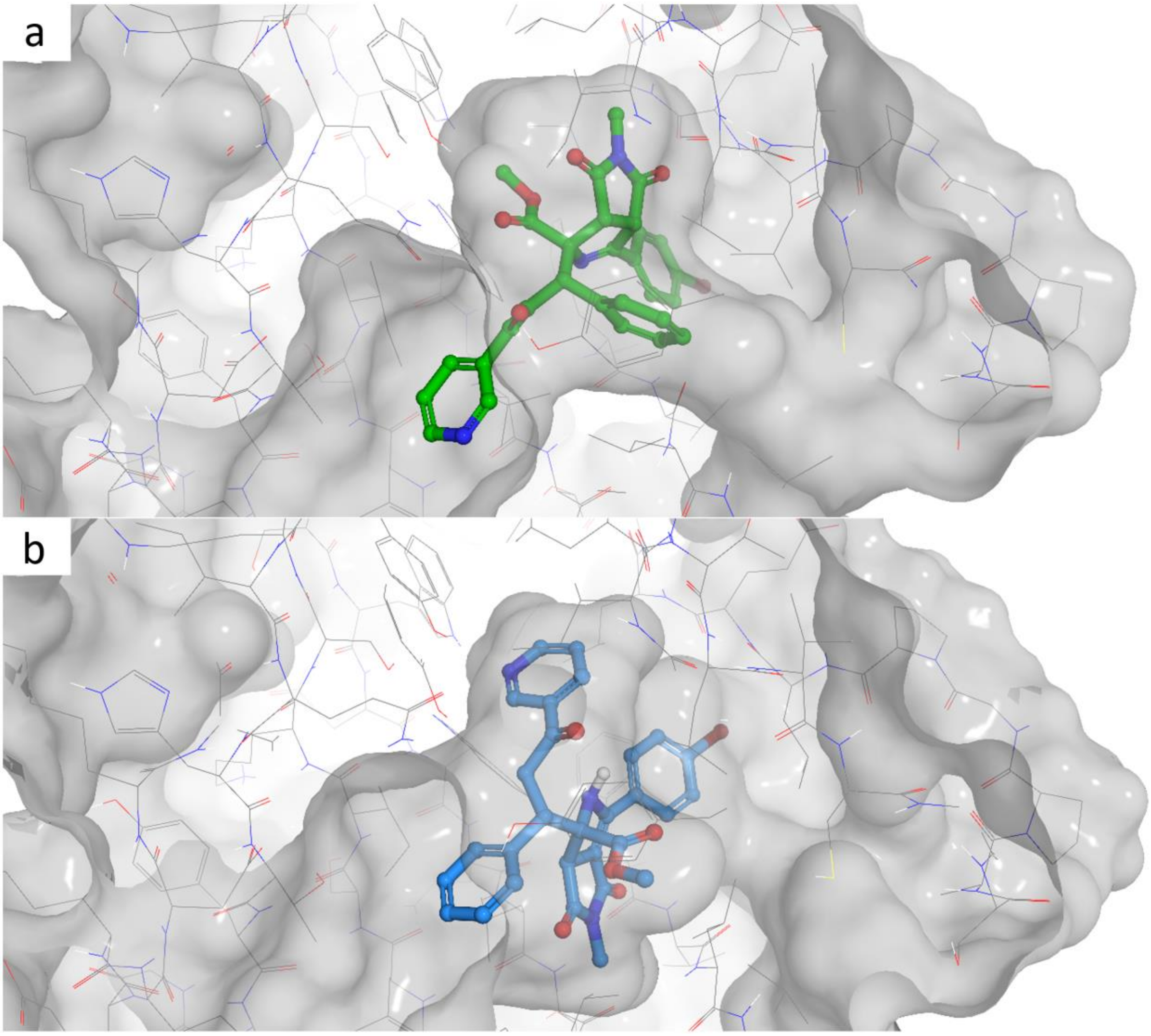

Supplementary Figure S8. Best IFD poses generated for imine-like structure (**a**; IDF Score -404.4, Glide G-Score -11.8) and enamine-like structure (**b**; IFD Score -404.7; Glide G-Score -13.1) of **7l** inside RhoGDI1. It appears that none of the afforded poses for the imine-like tautomer were in agreement with the SAR results, whereas, the best pose of the enamine-like tautomer seems to match with the experimental observations.

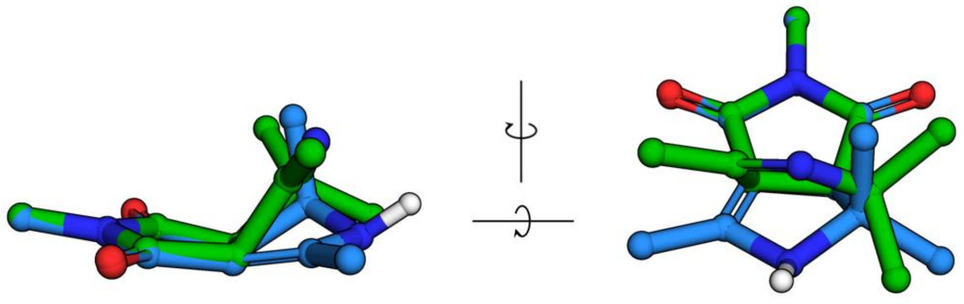

Supplementary Figure S9. Structural superposition visualising a difference in the core geometry of the minimised imine-like (green) and enamine-like (blue) tautomers of **7l**.

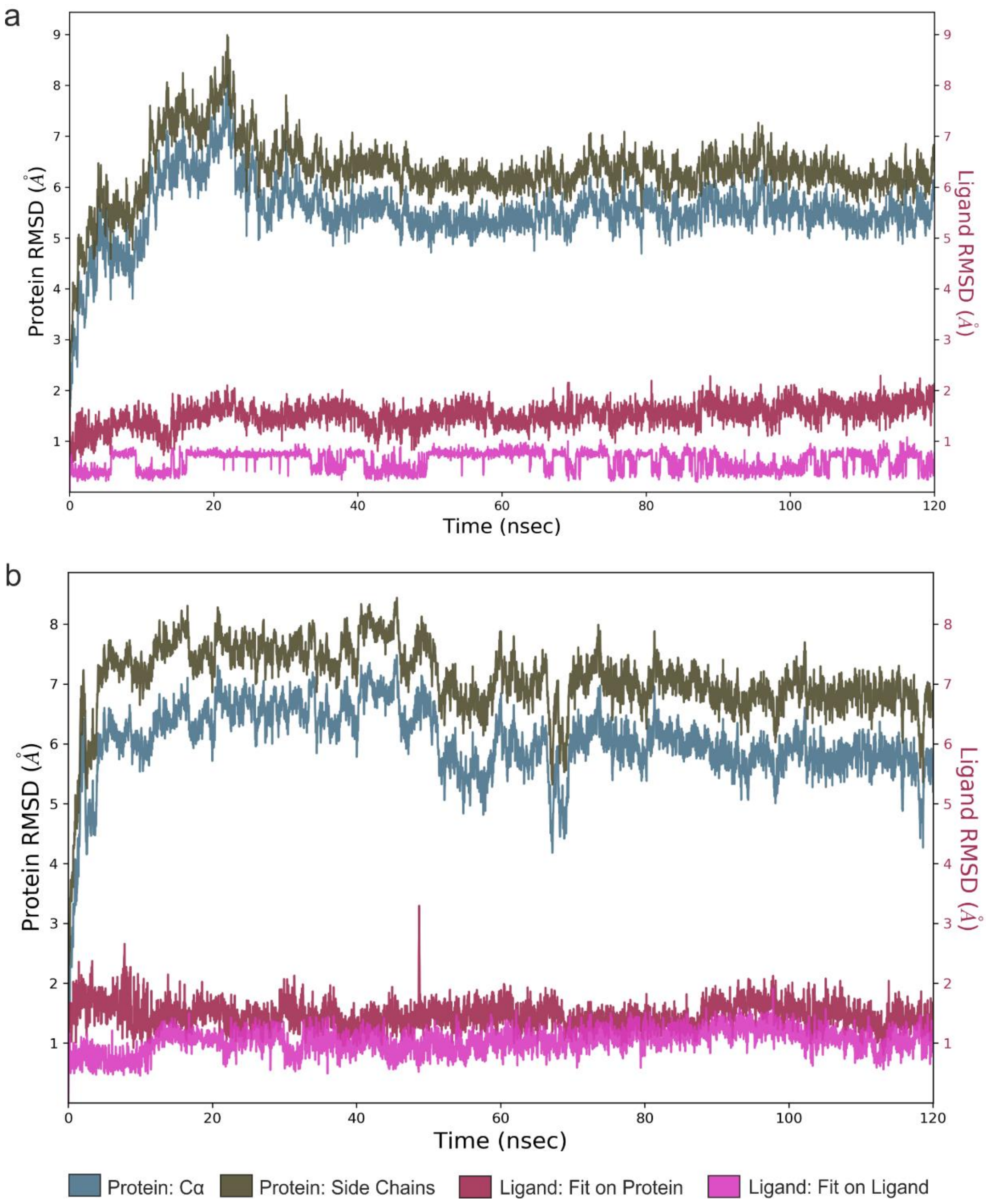

Supplementary Figure S10 (related to Figure 5). Protein and ligand (Rhonin, **7l**) root mean square deviation (RMSD) values for two MD production runs. (**a**) Production run number one. (**b**) Production run number two. The ligand was stabilized in the binding pocket throughout both simulations.

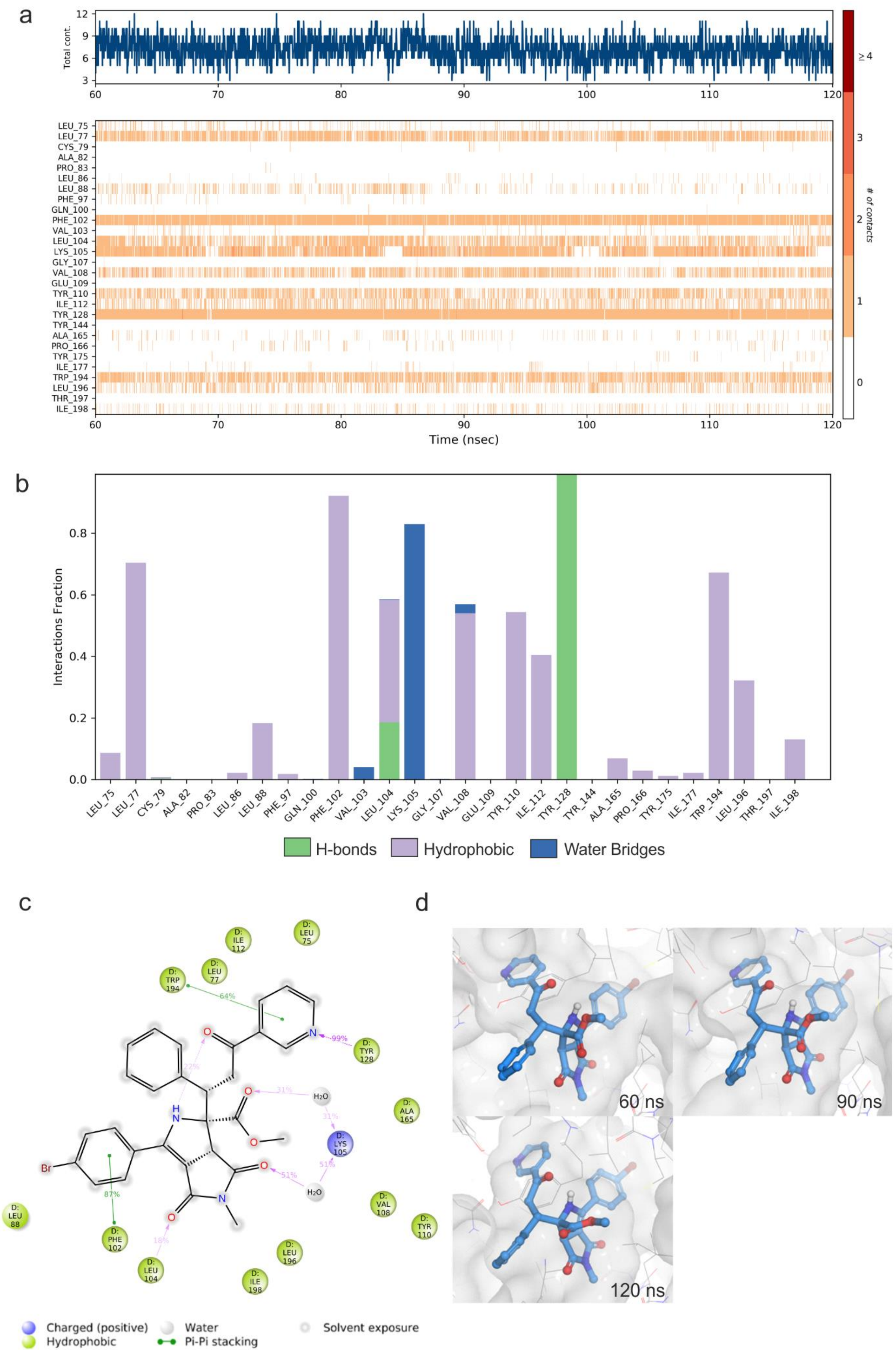

a
Total cont.
# of contacts
≥4
LEU_75
LEU_77
CYS_79
ALA_82
PRO_83
LEU_86
LEU_88
PHE_97
GLN_100
PHE_102
VAL_103
LEU_104
LYS_105
GLY_107
VAL_108
GLU_109
TYR_110
ILE_112
TYR_128
TYR_144
ALA_165
PRO_166
TYR_175
ILE_177
TRP_194
LEU_196
THR_197
ILE_198
Time (nsec)
b
Interactions Fraction
H-bonds
Hydrophobic
Water Bridges
c
Charged (positive)
Hydrophobic
Water
Pi-Pi stacking
Solvent exposure
d
60 ns
90 ns
120 ns

Supplementary Figure S11. Rhonin (7l)-RHOGDI1 interactions observed during the MD simulation (60-120 ns) (related to Figure 5)**.** The interactions are presented for the production run number one, whereas production run number two gave nearly identical results. (**a**) Protein-ligand contacts. The top panel shows the total number of contacts. The bottom panel indicates the residues interacting with the ligand over the time of the simulation. (**b**) Overview of categorized protein-ligand contacts. (**c**) Schematic 2D representations of the ligand-protein contacts. Interactions occurring more than 5% of the simulation time (during 60-120 ns of the run) are shown. (**d**) Snapshots of Rhonin bound to the GerGer binding pocket at 60 ns, 90 ns and 120 ns.

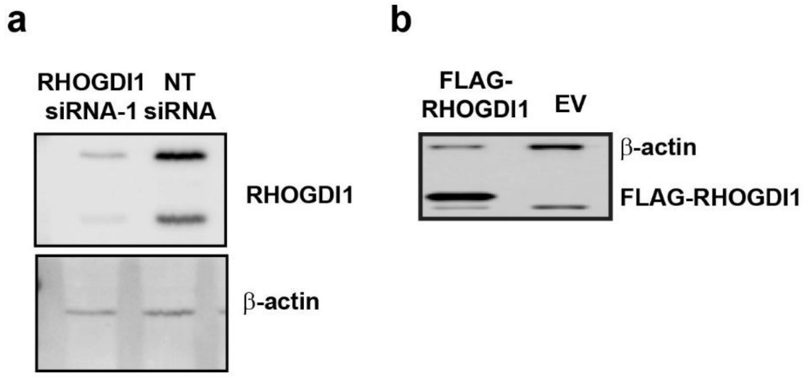

Supplementary Figure S12 (related to Figure 6). Depletion or overexpression of RHOGDI1. **(a)** C3H/10T1/2 cells were transfected with 10 nM RHOGDI1 siRNA and control siRNA (NT). 48 h later cells were re-plated and incubated for 96 h. Knockdown efficiency: 88 % (from four independent experiments). **(b)** C3H/10T1/2 cells were transfected with 800 ng FLAG-RHOGDI1 plasmid or empty vector (EV). Levels of RHOGDI1 and β-actin were detected by means of immunoblotting using antibodies against RHOGDI1 and β-actin, respectively.

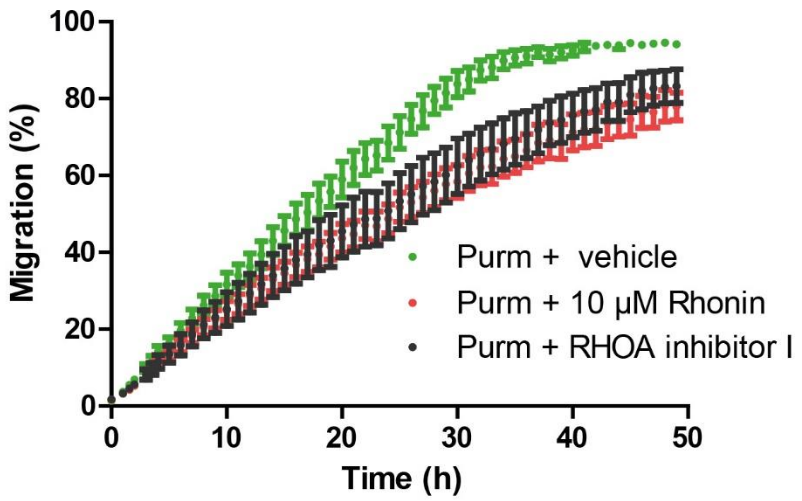

Supplementary Figure S13. Influence of 10 µM Rhonin on cell migration in NIH/3T3 cells upon stimulation with purmorphamine (Purm, 2 µM). The RHO inhibitor I was used as a control. Cell migration was monitored by means of a wound healing assay. Data are representative of three independent experiments (n=3, N = 3), mean values ± SD.

a

Figure 5g

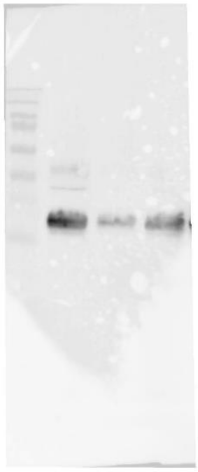

b

Figure 6g

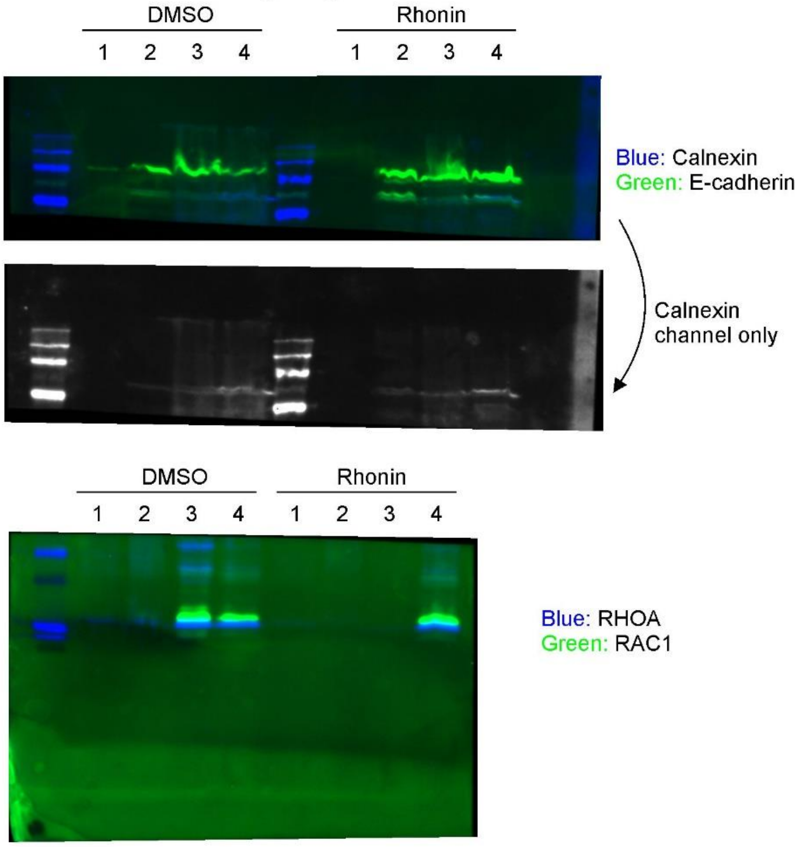

c

Figure S4b

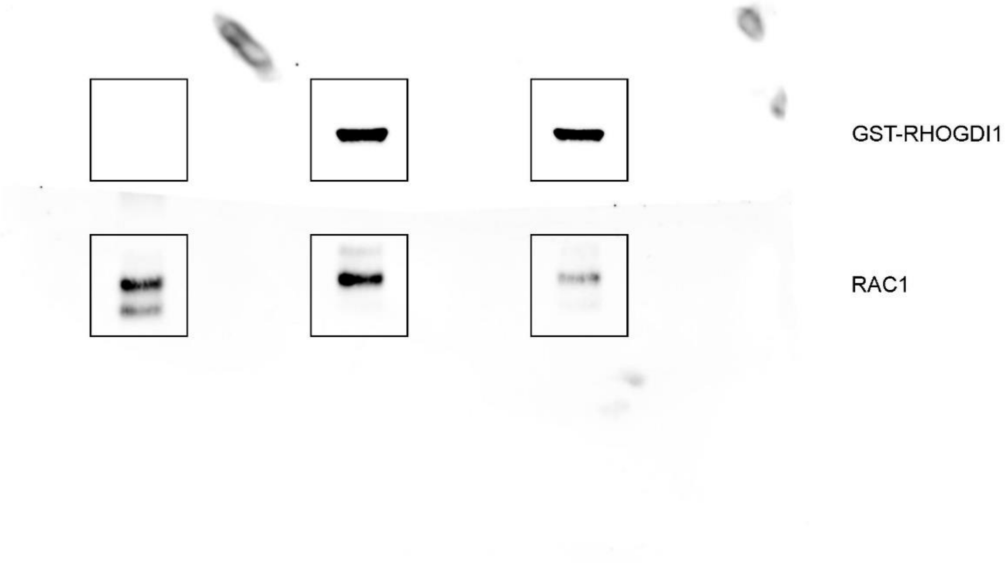

Supplementary Figure S14. Uncropped immunoblots.

Experimental section

Chemistry

General information

Unless otherwise noted, all commercially available compounds were used as provided without further purifications. Dry solvents (THF, toluene) were used as commercially available; CH_2Cl_2 was purified by the Solvent Purification System *M-BRAUN Glovebox Technology SPS-800*. Solvents for chromatography were technical grade.

Analytical thin-layer chromatography (TLC) was performed on *Merck silica gel aluminium plates* with F-254 indicator. Compounds were visualized by irradiation with UV light or potassium permanganate staining. Column chromatography was performed using *silica gel Merck 60* (particle size 0.040-0.063 mm). Solvent mixtures are understood as volume/volume. ^{1}H-NMR and ^{13}C-NMR were recorded on a *Bruker DRX400* (400 MHz), *Bruker DRX500* (500 MHz) and *INOVA500* (500 MHz) using $CDCl_3$ or $(CD_3)_2SO$ as solvent. Data are reported in the following order: chemical shift (δ) values are reported in ppm with the solvent resonance as internal standard (CDCl3: δ = 7.26 ppm for ^{1}H, δ = 77.16 ppm for ^{13}C; $(CD_3)_2SO$: δ = 3.30 ppm for ^{1}H, δ = 39.52 ppm for ^{13}C); multiplicities are indicated br s (broadened singlet), s (singlet), d (doublet), t (triplet), q (quartet) m (multiplet); coupling constants (*J*) are given in Hertz (Hz).

High resolution mass spectra were recorded on a *LTQ Orbitrap* mass spectrometer coupled to an *Acceka HPLC*-System (HPLC column: *Hypersyl GOLD*, 50 mm x 1 mm, particle size 1.9 µm, ionization method: electron spray ionization). Fourier transform infrared spectroscopy (FT-IR) spectra were obtained with a *Bruker Tensor 27* spectrometer (ATR, neat) and are reported in terms of frequency of absorption (cm^{-1}). Optical rotations were measured in a *Schmidt + Haensch Polartronic HH8* polarimeter. The enantiomeric excesses were determined by HPLC analysis using a chiral stationary phase column (column: CHIRALCEL IC, eluent: (DCM/EtOH = 100/2) / *iso*-hexane). The chiral HPLC methods were calibrated with the corresponding racemic mixtures. The ratio of diastereomers was determined by 1H-NMR analysis. Chemical yields refer to pure isolated substances. Yields, enantiomeric excesses, and diastereoselectivity are given in the tables.

The chemicals and solvents were purchased from the companies Sigma-Aldrich, Acros Organics, ABCR and Alfa Aesar. (*Rp*)-2-(*tert*-Butylthio)-1-(diphenyl-phosphino)ferrocene (purity: 98%), Tetrakis(acetonitrile)copper(I) hexafluorophosphate (purity; 97%) and Tetrakis(acetonitrile)copper(I) tetrafluoroborate (purity: 97%) were purchased from Sigma-Aldrich.

General procedures

GP-1: General Optimized Procedure for the formation for double cycloaddition product 5:

$Cu(CH_3CN)_4PF_6$ (5 mol%) and (*R*)-Fesulphos (5 mol%) was transferred to a dry reaction vial with a stir bar and 0.5 mL of dry CH_2Cl_2 was added and stirred for 10 mins at rt. Then, first maleimide **1** (1 equiv) and azomethine ylide **2** (1.05 equiv) was successively added followed by the addition of Et_3N (20 mol%). The reaction mixture was left to stir at 0°C till the completion (0.5 – 1h) as monitored by TLC. After the completion of the cycloaddition, TBHP (70% aq. soln) (1.2 equiv) was added to the reaction mixture and left to stir at rt till completion (monitored by TLC). Then, all the volatiles were removed in vacuo and THF (1mL/0.1 mmol) was added to the reaction vial. To this solution, DBU (0.5 equiv) was added and finally, *N*-phenylmaleimide (3 equiv as solution in 0.5 mL THF) was added dropwise and stirred at rt till completion. The crude reaction mixture was directly put on the column and separated using EA/PE (40-80%) mixture. The products **5** are very weakly UV active but can be visualized using $KMnO_4$ stain.

GP-2: General Optimized Procedure for the formation for Michael Addition product 6 and 7:

$Cu(CH_3CN)_4PF_6$ (5 mol%) and (*R*)-Fesulphos (5 mol%) was transferred to a dry reaction vial with a stir bar and 0.5 mL of dry CH_2Cl_2 was added and stirred for 10 mins at rt. Then, maleimide **1** (1 equiv) and azomethine ylide **2** (1.05 equiv) was successively added followed by the addition of Et_3N (20 mol%). The reaction mixture was left to stir at 0°C till the completion (0.5 – 1h) as monitored by TLC. After the completion of the cycloaddition, TBHP (70% aq. soln) (1.2 equiv) was added to the reaction mixture and left to stir at rt until completion (monitored by TLC). Finally, to this solution, base and electrophile was added and the mixture was stirred at rt until completion. The crude reaction mixture was directly put on

the column and separated using EA/PE (30-60%) mixture. The products **6/7** are UV active and can be visualized under standard UV lamp.

Biology

Materials

Dulbecco's Modified Eagle's medium (DMEM), L-glutamine, sodium pyruvate, penicillin/streptomycin, fetal bovine serum (FBS) and fetal calf serum (FCS) were obtained from PAN Biotech, Germany. The chemiluminescent substrate CDP-Star was purchased from Roche, Switzerland. Dual-Luciferase Reporter Assay System was obtained from Promega, USA. Anti-SMO rabbit Ab (#ab38686) and anti-β-actin rabbit polyclonal Ab (#ab8227) were purchased from Abcam, USA. Anti-N-acetylated tubulin mouse Ab (#T6793) was purchased from Sigma Aldrich, Germany. Anti-RHOGDI-1 mouse Ab (#sc-373724) was purchased from Santa Cruz Biotechnology, USA. Anti-Filamin-B polyclonal Ab (# PA5-21345) and anti-Filamin-C polyclonal Ab (# PA5-45573) were purchased from Thermo Fisher Scientific, Germany. Phalloidin coupled to TRITC was obtained from Sigma, Germany. Anti-rabbit-Alexa594 goat Ab (#A11012) and anti-mouse-Alexa488 donkey (#A21202) were purchased from Invitrogen, Germany. Anti-mouse-IR800 donkey Ab (#926-32212) and anti-rabbit-IR680 donkey (#926-68072) antibodies were obtained from LI-COR, Biosciences, USA, anti RhoA (67B9) Rabbit Ab (#2117) were purchased from Cell Signaling Technologies. Anti-CDC42 Clone 44/CDC42 (RUO) mouse (# 610929) was purchased from Bioscience. Anti-Rac1 mouse Ab, clone 23A8 (# 05-389) was purchased from Merck.

pGEN-mSMO, was a gift from Philip Beachy, Addgene plasmid # 37673, Addgene, USA.[4] BFP-SMO plasmid was described previously.[5] pCMV3-N-FLAG-mArhgdia (MG52485-NF) and pCMV3-N-Flag-NCV (CV016) were purchased from Sino Biologicals, Beijing, China. siRNA duplexes (mouse Arhgdia (192662) siRNA – CGACUGACCUUGGUAUGCA, J-064240-05-0002; non-targeting siRNA - UGGUUUACAUGUCGACUAA, D-001810-01-05) were purchased from GE Healthcare Europe GmbH, Germany.

Cell lines

NIH/3T3 cells were obtained from DSMZ, Germany (DSMZ ACC 59) and were cultured in DMEM (high glucose) supplemented with 10% heat-inactivated FCS, 2 mM L-glutamine and 1 mM sodium pyruvate. Shh-LIGHT2 cells (NIH/3T3 cells stably transfected with a Gli-responsive firefly luciferase reporter plasmid and a pRL-TK constituitive Renilla luciferase expression vector)[4] were cultured in the same culturing medium supplemented with 400 μg ml^{-1} G418 and 150 μg ml^{-1} Zeocin as selecting agents. The murine osteoblasts C3H/10T1/2 (ATCC CCL-226) were obtained from ATCC, USA and were cultured in DMEM (high glucose) supplemented with 10% heat-inactivated FCS, 6 mM L-glutamine, 1 mM sodium pyruvate. HEK293T cells (ATCC, England) were grown in DMEM containing 10% FBS, 100 U ml^{-1} penicillin and 0.1 mg ml^{-1} streptomycin. All cell lines were maintained at 37 °C and 5% CO_2 in a humidified atmosphere. All the cell lines were regularly assayed for mycoplasma and were confirmed to be mycoplasma-free. Transfection of DNA constructs and siRNAs were performed using FuGENE HD (Promega), Lipofectamine® LTX (Invitrogen) and DharmaFECT™ (GE Healthcare) transfection reagent according to the manufacturer's directions.

Osteoblast differentiation assay

The osteoblast differentiation assay was performed by the Compound Management and Screening Center (COMAS) in Dortmund, Germany. C3H/10T1/2 cells were seeded (800 per well) in white 384-well plates or (6000 per well) in 96-well plates. After incubation for 16 h, compounds or DMSO were added to the cells using the acoustic nanoliter dispenser ECHO 520 (Labcyte) and osteogenesis was induced with 1.5 μM purmorphamine. Plates were incubated for 96 h at 37 °C and 5% CO_2. Alkaline phosphatase activity was measured by replacing the medium with osteogenesis-lysis buffer (lysis buffer: 100 mM Tris pH 9.5, 250 mM NaCl, 25 mM $MgCl_2$, 1% Triton X-100, sterile filtered) containing 1:100 chemiluminescent substrate CDP-Star® (Promega, 384-well plates: 35 μL, 96-well plates: 50 μL) for one hour in the dark at 25 °C. In 384-well plates, the luminescence was monitored using the Paradigm plate reader (Molecular Devices, USA), whereas Spark plate reader (Tecan, Austria) was used to measure luminescence in 96-well plates. Dose-response analyses were performed for all hit compounds

using a three-fold dilution series starting from a concentration of 30 μM. Half-maximal inhibitory concentrations (IC_{50}) were calculated using the Quattro software suite (Quattro Research, Planegg, Germany) or GraphPad Prism 7 (GraphPad, USA).

The effect of test compounds on the viability of C3H/10T1/2 cells was determined by CellTiter-Glo Luminescence cell viability assay (Promega). Cells were treated as described for the osteogenesis assay prior to addition of the CellTiter-Glo® reagent according to manufacturer's protocol. Compounds causing at least a 50% reduction in the osteogenesis assay and retaining cell viability of at least 80% were considered as hits. Dose-response analyses were performed for all hit compounds using a three-fold dilution series starting from a concentration of 30 μM. Half-maximal inhibitory concentrations (IC_{50}) were calculated using the Quattro software suite (Quattro Research GmbH).

GLI-dependent reporter gene assay

Shh-LIGHT2 cells were seeded (2.5×10^4 per well) in 96-well plates. After incubation for 16 h, medium was replaced by low serum-containing medium (0.5% FCS) supplemented with 1.5 μM purmorphamine and various concentrations of the compounds or DMSO as a control and cells were incubated for 48 h. Firefly and *Renilla* luciferase activities were determined by means of the Dual-Luciferase® Reporter Assay System (Promega) according to the manufacturer's protocol and luminescence was measured using the Spark plate reader (Tecan, Austria).

SMO binding assay

HEK293T cells were seeded (1.5×10^4 per well) on poly-D-lysine-coated coverslips placed in a 24-well plate. 24 h later cells were transfected with the SMO-expressing plasmid (pGEN-mSMO) using FuGENE HD (Promega) according to the manufacturer's protocol. After 48 h of incubation at 37 °C, cells were washed once with PBS, fixed with 3% paraformaldehyde for 10 min at room temperature and subsequently permeabilized with 0.1% (v/v) Triton X-100 in PBS for 5 min at room temperature. Cells were then washed three times with PBS, followed by treatment with various concentrations of the test compounds or DMSO as a control diluted

in fresh medium containing 0.5 % FBS and 5 nM BODIPY-cyclopamine for 4 h. Afterwards, cell nuclei were stained with 1 μg/ml DAPI for 10 min, samples were washed three times with PBS and subsequently mounted on glass slides using Aqua Polymount (Polysciences Inc). Images were acquired on an Axiovert Observer Z1 microscope (Carl Zeiss, Germany) using a Plan-Apochromat 63x/1.40 Oil DIC M27 objective.

Additionally, SMO binding was confirmed by flow cytometry. HEK293T cells were seeded (3×10^5 per well) in 6-well plate and transfected with BFP-SMO-expressing plasmid using FuGENE HD transfection reagent according to the manufacturer's instructions. After 48 h of incubation the medium was replaced by DMEM containing 0.5% FBS, 5 nM BODIPY-cyclopamine and various concentrations of the test compounds or DMSO as a control. Following incubation for 4 h cells were washed once with PBS, detached using trypsin/EDTA (0.05/0.02% (v/v) in PBS), and collected by centrifugation at 250 x g for 5 min at 4°C. Cells were washed twice and then suspended in ice-cold PBS. Cell suspensions were subjected to flow cytometry analysis employing the BD LSR II Flow Cytometer (laser line: 488 nm, emission filter: 530/30) to detect the presence of BODIPY. Data analysis was performed using the FlowJo software, version 7.6.5 (Tree Star Inc., USA).

Smoothened Trafficking

To detect ciliary localization of SMO, NIH/3T3 cells (2×10^4 per well) were seeded on coverslips in 24-well plates and cultured for 24 h at 37°C and 5% CO_2. Cells were then incubated for 24 h in DMEM containing 0.5% FCS to induce ciliation. The next day, cells were treated with purmorphamine (1.5 μM) for 2 h prior to addition the compounds, 1 μM Vismodegib or DMSO as a control, in DMEM containing 0.5 % FCS. 12 or 24 h later, cells were washed with PBS followed by fixation in 4% paraformaldehyde for 10 min. Cells were then permeabilized for 5 min with 0.3 % Triton-X100 in PBS and blocked 10 % FCS in PBS for 1 h at room temperature. Samples were then incubated with a rabbit anti-N-acetylated tubulin antibody (Cell Signaling Technology, dilution 1:1000) as a marker for cilia and a mouse anti-SMO antibody (Santa Cruz Biotechnology, dilution 1:500) at 4 °C. The next day, cells were washed three times with PBS and subsequently incubated with Alexa Fluor 594-conjugated goat anti-rabbit and Alexa Fluor 488-conjugated donkey anti-mouse antibodies (1:1000 dilutions) and DAPI (0.1 μg/mL) for 1 h at room temperature. Coverslips were washed

three times with PBS and mounted on glass slides using Aqua Polymount (Polysciences Inc). Images were acquired as Z-sections with Leica SP5 confocal microscope using a 63x 1.40 NA Oil objective (Leica Microsystems CMS GmbH, Mannheim, Germany). Image analysis was performed using the software ImageJ. Briefly, Z-stacks were converted to intensity sum projections, after previous background subtraction. For quantification, cilia were identified and marked based on the acetylated-tubulin staining intensities and the intensities of the anti-Smoothened antibody were measured within these areas. Results were visualized and significances were assessed using an unpaired t-test (confidence interval of 95%) with the software GraphPad Prism 7, GraphPad software, USA). Three independent biological replicates were performed and for each replicate at least 100 cilia per condition were analyzed.

Reverse transcription- quantitative PCR (RT-qPCR)

C3H10T1/2 cells were seeded (1×10^5 per well) in 6-well plates. 48 h later cells were treated with 1.5 µM purmorphamine and DMSO, or only DMSO, or with 1.5 µM purmorphamine and different concentrations of the test compounds for 96 h. Total RNA was isolated using the RNeasy mini kit (Qiagen) according to the manufacturer's protocol, including the optional DNase I digestion step. The concentration of purified RNA was determined by means of the Nanodrop 2000. 500 ng total RNA was used to prepare complementary DNA (cDNA) using QuantiTect Reverse Transcription Kit (Qiagen) according to the manufacturer's protocol. The expression of Hh target genes *Ptch1* and *Gli1* and the reference genes *Gapdh* and *Ap3d1* was determined by means of quantitative RT-PCR using the QuantiFast SYBR Green PCR Kit (Qiagen) and CFX96 Touch Real-Time PCR Detection System (Bio-Rad) and the following oligonucleotides:

Ptch1: 5′-CTCTGGAGCAGATTTCCAAGG-3′ and 5′- TGCCGCAGTTCTTTTGAATG-3′, *Gli1*: 5′-CACCGTGGGAGTAAACAGGCCTTCC-3′ and 5′-CCAGAGCGTTACACACCTGCCCTTC -3′, *Gapdh*: 5′-CAGTGCCAGCCTCGTC-3′ and 5′-CAATCTCCACTTTGCCACTG-3′ and *Ap3d1*: 5'-CAGAGGGCTCATCGGTACAC-3' and 5'- GCCGGAAGTCCAACTTCTCA-3'. *Gapdh* and *Ap3d1* served as housekeeping genes. The expression levels of *Ptch1* and *Gli1* were determined using the $2^{-\Delta\Delta}$Ct method.[6] Expression levels of *Ptch1* and *Gli1* were normalized to the levels of *Ap3d1* and *Gapdh* and were related to the value of purmorphamine-treated cells (set to 100 %).

Knockdown of RHOGDI1

C3H10T1/2 cells were transiently transfected with RHOGDI1 siRNA and non-targeting siRNA (NT) using DharmaFECT™ reagent by employing the manufacturer's protocol. Briefly, 6x10^5 C3H10T1/2 cells were seeded in T25 cell culture flask. Upon reaching ca. 70 % confluence, cells were transiently transfected with DharmaFECT™ transfection reagent. For the transfection, siRNAs and DharmaFECT™ transfection reagent were independently mixed with Opti-MEM® serum-free media in low-binding tubes and incubated for 5 min at room temperature. Subsequently, the lipid solution was added to the siRNA solution in the ratio 1:1 and incubated for 20 min at room temperature. The corresponding lipid:siRNA solution was diluted 1:5 with the respective cell culture medium and was added to cells , which were seeded on the previous day, by replacing the old medium and incubated for 48 h at 37 °C. Cells were detached and reseeded at 20,000 cells per well in 24-well plate for determining the target gene expression upon RHOGDI1 knockdown and qPCR experiments, or at 6,000 cells per well in 96-well plates for osteoblast differentiation assays. Knockdown efficiency was determined by immunoblotting using anti-RHOGDI1 specific antibody (Santa Cruz Biotechnology, dilution 1:1000).

Immunoblotting

Unless otherwise mentioned, the cells were lysed in 1X SDS buffer without bromophenol blue. Protein concentrations were determined using DC Assay (Bio-Rad). The cell lysates were then supplemented with bromophenol blue and boiled at 95 °C for 10 min. Lysates were loaded on 10 % polyacrylamide gels and run at a constant voltage of 80 V for 15 min followed by 120 V for approximately 1.5 h. Protein were transferred on PVDF membrane using semi-dry transfer at 25 V for 45 min or alternatively by using tank blotting at 100 V for 60 min. Membranes were washed and blocked in LI-COR blocking buffer for 1 h at room temperature. The blocking buffer was replaced with fresh LI-COR blocking buffer containing primary antibodies and incubated overnight at 4 °C. Membranes were washed with TBS-T (3 x 5 min) and incubated with the secondary antibody in blocking buffer for one hour at room temperature. Signals were visualized using Odyssey Fc imaging system (LI-COR Biosciences). Quantification of band

intensities was performed by densitometry of scanned signals with the aid of image studio software (Version 4.0.21, LI-COR Biosciences, ©2014).

RHOGDI1 overexpression

C3H10T1/2 cells were transiently transfected with pCMV3-N-FLAG-mArhgdia or pCMV3-N-Flag-NCV using Lipofectamine® LTX & PLUS ™ reagent according to manufacturer's protocol. Briefly, C3H10T1/2 cells were seeded in a T25 cell culture flask and incubated overnight at 37 °C and 5% CO_2. For the transfection, plasmid DNA and PLUS reagent were diluted in Opti-MEM® medium and incubated for 5 min at room temperature. Lipofectamine® LTX reagent was directly added to this mixture and incubated for 30 min at room temperature. The DNA-lipid complex was added dropwise to the cells in the flask and incubated for 24 h. Cell were then detached and reseeded at 6,000 cells per well in 96-well plate for determining the effect of RHOGDI1 overexpression on osteoblast differentiation as described above. For the osteoblast differentiation assay, 24 h post transfection, cells were treated with 1.5 µM purmorphamine and DMSO and osteoblast differentiation was monitored after 72 h. The Hh pathway activity in cells that were transfected with the empty vector and treated with purmorphamine was set to 100%.

Cell lysate preparation for chemical proteomics

NIH/3T3 cells were grown to 90-95% confluence and were then detached using cell dissociation solution for 5 min at 37 °C and 5% CO_2. Cells were suspended in cell culture medium and pelleted by centrifugation at 250 x g, 5 min at 4 °C. Cell pellets were then washed thrice with ice-cold PBS and lysed in lysis buffer containing 50 mM PIPES, 50 mM NaCl, 5 mM $MgCl_2$, 5 mM EGTA, 0.1 % NP40, 0.1 % Triton X-100, 0.1 % Tween®20, with freshly added 1 mM DTT, EDTA-free protease inhibitors (Complete EDTA-free, Roche) and phosphatase inhibitors (PhosphoSTOP, Roche). To assure complete cell lysis, the suspension was passed through 0.9 µm and 0.45 µm cannula 10 times each and incubated on ice for 40 min with intermittent vortexing every five minutes until a homogeneous mixture was obtained. The resulting homogenate was centrifuged at 18,000 x g for 30 min at 4 °C and the supernatant was subsequently snap frozen in liquid nitrogen and stored at -80 °C. Total protein content was determined by DC protein assay (Bio-Rad, Germany).

Affinity chromatography enrichment of compound-bound proteins

25 µL N-hydroxysuccinimide magnetic beads (GE Healthcare) were activated with 500 µL 1 mM HCl for 1 min. HCl was replaced by 500 µL coupling buffer (0.15 M triethanolamine, 0.5 M NaCl, pH 8.3) containing 10 µM of free amine affinity probes (**S11a** and **S11b**) and incubated for 2 h at room temperature with overhead rotation. The residual active groups of N-hydroxysuccinimide magnetic were quenched by alternating incubation with 500 µL block A buffer (0.5 M ethanolamine, 0.5 M NaCl, pH 8.3) and block B buffer (0.1 M sodium acetate, 0.5 M NaCl, pH 4.0) for 5 min three times each. For protein binding, the magnetic beads were equilibrated with 500 µL lysis buffer followed by incubation with 500 µL lysate (protein concentration: 2 mg/mL) 2 h at 4 °C with overhead rotation. In order to wash out non-specifically bound proteins, the beads were first washed with 500 µL lysis buffer containing 25 mM $MgCl_2$ followed by three times washing with 500 µL PBS. Tryptic digestion of bead-bound proteins was carried out in two stages. First, bound proteins were reduced by incubating the beads with 50 µL reducing buffer (1 mM DTT, 8 M urea in 50 mM Tris (pH 7.5)) at 30 °C for 30 min with shaking. Later, the reduced proteins were alkylated by addition of 5.5 µL alkylating solution (1 mM DTT, 50 mM chloroacetamide, 8 M urea in 50 mM Tris pH 7.5) and further incubation in the dark at 30 °C for 30 min while shaking at 350 rpm. The reduced and alkylated proteins were digested by adding 1 µg LysC at 37 °C for 1 h while shaking at 350 rpm. The supernatant was transferred to a new tube and the beads were further incubated with 50 mM Tris-HCl (pH 7.5) containing 1 µg trypsin at 37 °C for 1 h while shaking at 350 rpm. Both supernatants were combined together and further digested by adding 2 µg trypsin for 16 h at 37 °C with continuous shaking. The enzymatic digestion was stopped by addition of 2 µL proteomics grade trifluoroacetic acid (TFA). In order to purify the resulting peptides, STAGE tips were prepared by loading the 200 µL microtips with two layers of C18 (octadecyl) disks. The tips with C18 disks were activated by addition of 100 µL methanol. Furthermore, the tips were calibrated by washing with 100 µL Buffer B (20% H_2O / 80% acetonitrile with 0.1% formic acid) and 100 µL Buffer A (H_2O with 0.1% formic acid). The peptide purification was achieved by passing the digestion solution over the activated STAGE tips. Bound peptides were eluted twice using 20 µL Buffer B and the solvent was evaporated by means of rotary speed vacuum evaporator.

Mass spectrometry and data evaluation

In order to analyse the tryptic peptides, the samples were purified by UltiMateTM 3000 RSLC nano system (Dionex, Germany) and MS/MS analysis was carried out using Q Exactive^{TM} HF Hybrid Quadrupole-Orbitrap Mass Spectrometer equipped with a nano-spray source (Nanospray Flex Ion Source, Thermo Scientific). Briefly, the tryptic peptides were solubilized in 20 µL 0.1% (v/v) TFA in water and 3 µL were injected onto a pre-column cartridge (5 µm, 100 Å, 300 µm ID * 5 mm, Dionex, Germany) using 0.1% (v/v) TFA in water as eluent with a flow rate of 30 µL/min. Desalting was performed for 5 min with eluent flow through followed by back-flushing of the sample during the whole analysis from the pre-column to the PepMap100 RSLC C18 nano-HPLC column (2 µm, 100 Å, 75 µm ID × 50 cm, nanoViper, Dionex, Germany) using a linear gradient starting with 95% water containing 0.1% (v/v) formic acid / 5% (v/v) acetonitrile containing 0.1% (v/v) formic acid and increasing to 70% water containing 0.1% (v/v) formic acid / 30% (v/v) acetonitrile containing 0.1% (v/v) formic acid after 95 min using a flow rate of 300 nL/min. The nano-HPLC was coupled to the Quadrupole-Orbitrap Mass Spectrometer using a standard coated SilicaTip (ID 20 µm, Tip-ID 10 µM, New Objective, Woburn, MA, USA), mass range of m/z 300 to 1650 was acquired with a resolution of 60000 for a full scan, followed by up to ten high energy collision dissociation (HCD) MS/MS scans of the most intense at least doubly charged ions with a resolution of 15000.

Data evaluation was performed using MaxQuant software[7] (v.1.5.3.30) including the Andromeda search algorithm and searching the mouse reference proteome of the Uniprot database. Briefly, the search was performed for full enzymatic trypsin cleavages allowing two miscleavages. For protein modifications carbamidomethylation was chosen as fixed and oxidation of methionine and acetylation of the N-terminus as variable modifications. The mass accuracy for full mass spectra was set to 20 ppm for the first and 4.5 ppm for the second search. The mass accuracy for MS/MS spectra was set to 20 ppm. The false discovery rates for peptide and protein identification were set to 1%. Relative quantification of proteins was carried out using the label-free quantification algorithm implemented in MaxQuant. Further data evaluation was performed using Perseus software[8] (v. 1.5.2.6). Proteins not identified with at least two peptides in at least one of the samples and known contamination were filtered off. Samples resulting from pulldown using the active probe were grouped together and those from

the pulldown using the inactive one as well. Label-free quantification (LFQ) intensities were logarithmized (log2) and proteins, which were not three times quantified in at least one of the groups, were filtered off. Missing values were imputed using small normal distributed values (width 0.3, down shift 1.8) and a two sided t-test (s0 = 1, FDR 0.05) was performed. Proteins which were statistically significant enriched by the active probe compared to the inactive one were considered as hits.

Fluorescence polarization

The binding of probe **10** to RHOGDI1-3 was confirmed by fluorescence polarization experiments. RHOGDI1 was titrated against 0.5 μM of the fluorescent Rhonin derivative **10** in buffer containing 20 mM Hepes, pH 7.4, 150 mM NaCl, 5 mM $MgCl_2$, 3 mM DTT and incubated for 1 h. The change in fluorescence polarization was monitored at an excitation / emission wavelengths of 535 nm and 593 nm, respectively. To understand the impact of geranylgeranyl moiety on the interaction of RHOGDI1 and RAC1, 5 μM RHOGDI was added to 2 μM fluorescent Rhonin in buffer containing 20 mM HEPES, pH 7.4, 150 mM NaCl, 5 mM $MgCl_2$, 3 mM DTT, led to increase in polarization signal. 2 μM of prenylated/ or non-prenylated RAC1 were added in two independent experiments. Geranygeranylated RAC1 (GerGer-RAC1) was dissolved in a buffer containing 1% CHAPS and 0.5% sodium cholate in order to remain stable.

To determine the effect of **7a** on the binding of RHOGDI1 to non-prenylated RAC1-GDP, fluorescence polarization measurements were performed by titrating 1 μM TAMRA-GDP-bound RAC1 with increasing concentrations of RHOGDI1 in the presence and absence of 50 μM **7a** in buffer containing 30 mM Tris-HCl, pH 7.5, 150 mM NaCl, 5 mM $MgCl_2$, 1% DMSO. The change in the fluorescence polarization was monitored at an excitation and emission wavelengths of 557 nm and 583 nm, respectively.

Competition-based fluorescence polarization were performed by titrating **7a** with 30 nM fluorescein-labelled atorvastatin and 60 nM GST-PDEδ in PBS buffer containing 0.05% CHAPS,1% DMSO. Direct binding to PDEδ was monitored as for RHOGDI using 10 nM probe **10** and PDEδ.

Liposome sedimentation assay

Liposomes were prepared by self-assembly of the lipids (500 µg) containing 20% (w/w) phosphatidylethanolamine, 45% (w/w) phosphatidylcholine, 20% (w/w) phosphatidylserine, 10% (w/w) cholesterol, and 5% (w/w) phosphatidylinositol 4,5-bisphosphate. Liposome assays were performed by mixing, sonicating (20 s with minimal power, 50% off and 50% on) and extruding (0.2 µM filter) the lipids in 300 µl of a buffer, containing 20 mM HEPES-NaOH pH 7.4, 50 mM NaCl, 3 mM DTT, 5 mM $MgCl_2$. 1 µM GerGer-RAC1-GDP was added to the liposomes and incubated for 20 min on ice. 1.5 µM GST-RHOGDI-1 and the compounds or DMSO were added to the liposome-GerGer-RAC1-GDP complex prior to incubation on ice for 30 min. The samples were centrifuged at 20,000 x g for 30 min at 4 °C. The resulting pellet and supernatant fractions were collected and analyzed by immunoblotting. 10 µl of each sample were loaded on a SDS-PAGE gel for Western blotting. Specific antibodies were used to visualize RAC1, CDC42 and RHOA.

Liposome flotation assay

10 µM of GerGer-RAC1-GDP was added to liposomes suspended in flotation assay buffer containing 20 mM HEPES-NaOH pH 7.4, 50 mM M NaCl, 3 mM DTT, 5 mM $MgCl_2$, and incubated for 20 min on ice. The liposomes were prepared by using self-assembling the lipids (500 µg lipids in 300 µl buffer), containing 15% (w/w) phosphatidylethanolamine, 0.5% (w/w) NBD-PE (N-(7-nitrobenz-2-oxa-1,3-diazol-4-yl)-1,2-dihexadecanoyl-sn-glycero-3-phosphoethanolamine, triethylammonium salt), 45% (w/w) phosphatidylcholine, 20% (w/w) phosphatidylserine, 10% (w/w)cholesterol, and 5% (w/w) phosphatidylinositol 4,5-bisphosphate. 15 µM of GST-RHOGDI-1 and compounds or DMSO was added to liposome-GerGer-RAC1-GDP complex and samples were further incubated on ice for 30 min. The samples were added to 30% (w/v) sucrose solution. The resulting suspension was overlaid with protein buffer containing 25% (w/v) sucrose and finally with 50 µl of buffer without sucrose. The resulting samples were centrifuged at 140000 x g for 1 h at 4 °C. The upper liposome-containing phase (detected by fluorescent NDB-PE) was collected and analyzed by immunoblotting.

Surface plasmon resonance (SPR)

Biacore® X100 instrument (Biacore, now GE Healthcare) was used to analyze the effect of **7a** on the GerGer-RAC1-GDP and RHOGDI1 interaction. Liposomes suspended in SPR buffer (20 mM HEPES, pH 7.4, 50 mM NaCl, 5 mM $MgCl_2$, 3 mM DTT) were immobilized by injecting 0.5 mM liposomes with flowrate of 5 µL/min on the surface of a Pioneer® L1 sensor chip (GE Healthcare) for the period of 900 s, as indicated by a constant signal. Unbound liposomes were removed by passing the buffer and 10 mM NaOH with flowrate of 5 µL/min for 30 s over the sensor chip. Buffer containing 15 µM GerGer-RAC1-GDP was passed with flowrate of 5 µL/min over the immobilized liposomes to facilitate the loading of GerGer-RAC1-GDP on to the liposomes. The effect of compounds on the association of GerGer-RAC1-GDP with liposomes and the resulting dissociation of GerGer-RAC1 upon passing 25 µM of RHOGDI1 was monitored by the change in the response unit signal.

Protein expression and purification

RHOGDI1 delta15, RHOGDI1 delta25, RHOGDI1-3 were produced in *E. coli (XL1-Blue) as a GST recombinant protein as described previously.*[9] *In summary, protein were expressed in E.coli BL21 upon addition of IPTG overnight. Cell were collected and lysed in a lysis buffer,* containing 30 mM Tris-NaOH pH 7.4, 150 mM NaCl, 1 mM DTT, and 5 mM $MgCl_2$. Cells were further lysed *using sonication. After centrifugation (20 min at 20,000 g) of lysate, the clear supernatant was used for purification in an ÄKTAprime system connected to a GSH column (GE-Healthcare).*

Insect cell purification system was used for expression and purification of prenylated proteins based on an established protocol.[9] *Sf*9/or *TNAO*38 insect cells (*Sf*9 used only for virus generation and amplification) were grown to the level of 1.5×10^6 cells/ml before they were transduced with Baculoviruses encoding genes related to the RHO GTPases. Cells were resuspended in lysis buffer, containing 20 mM HEPES-NaOH pH 7.4, 150 mM NaCl, 2 mM β-mercaptoethanol, 5 mM $MgCl_2$, 0.1 mM GDP, 10 mM imidazole and the optimized detergents including 1% CHAPS and 0.5% sodium cholate.[9] Cells were lysed by sonication in ice. After

centrifugation for 20 min in 20000 x g, supernatants were collected and loaded on a Ni-NTA column (Qiagen, Hilden, Germany). The protein was eluted by passing a buffer containing 300 mM imidazole into the Ni-NTA column. The eluted solution was further purified on an analytical Superdex 75 column (10/300 GL, GE-Healthcare, Uppsala, Sweden) using 20 mM HEPES-NaOH pH 7.4, 150 mM NaCl, 3 mM DTT, 5 mM $MgCl_2$ and 0.5% (w/v) Na-cholate as buffer system. Non-prenylated RAC1 and human RHOGDI1 were prepared from *E. coli as a GST recombinant protein as described previously.*[6]

For expression of GST-PDEδ, *E. coli* RosettaTM2 cells transformed with PDE6D expression plasmid (pGex4T5) were inoculated in TB medium containing 100 µg/ml ampicillin and 30 µg/ml chloramphenicol to a total volume of 10 L. The protein expression in *E. coli* was induced with 0.2 mM IPTG at OD600 = 1.33. Cells were cultivated overnight at 18 ° C with shaking. Cells then were pelleted by centrifugation at 4000xg for 20 min at 4 ° C and stored at -20 ° C. To purify the protein, the pellet was resuspended in lysis buffer (30 mM TRIS-HCl pH 7.5, 100 mM NaCl, 1 mM 2-mercaptoethanol and 1 mM PMSF. DNaseI (10 µg/ml) was added to the cell suspension and the sample was sonicated three times for 1 min on ice and disrupted three times with Microfluidizer. To separate the cell debris, the lysate was centrifuged for 35 min at 20000xg. GST-PDEδ was purified by affinity chromatography via binding to GSH-Sepharose using FPLC. The lysate purified from cell debris was slowly pumped onto a GSH column using a peristaltic pump (the column was previously washed with 3 column volume lysis buffer) and washed with 5 column volume lysis buffer (until absorption at 280 nm was constant). The elution occurred with 4 column volume elution buffer (30 mM TRIS-HCl pH 7.5, 100 mM NaCl, 1 mM 2-mercaptoethanol, 200 mM glutathione). The GST-PDEδ containing fractions were concentrated by ultrafiltration and then slowly using a 5 ml Superloops on a gel filtration column (HiLoad Superdex 26/60 Superdex 75 prep grade, GE Healthcare) equilibrated with 1 column volume gel filtration buffer (20 mM TRIS-HCl pH 7.5, 300 mM NaCl, 1 mM 2-mercaptoethanol).

Cell fractionation

Cell fractionation was performed as described by Boulter et al.[10] HEK293T cells were treated with DMSO as control and Rhonin (10 µM) for 24 h. Cells were collected (100 x 10^6 cells) and incubated with ice-cold hypotonic lysis buffer (10 mM Hepes pH 7.3, 1.5 mM $MgCl_2$, 5 mM KCl, 1 mM DTT and protease inhibitors) for 30 min and then passed through insulin syringe. Lysed cells were centrifuged at 700 x g for 3 min and the supernatants were centrifuged at

40,000 x g for 30 min at 4°C. Supernatants were collected as cytoplasmic fraction and pellets were resuspended into a homogenization buffer (0.25 M sucrose, 20 mM Tris/HCl pH 7.4, 25 mM KCl, 5 mM $MgCl_2$) and further centrifuged (2 h at 100,000 x g) using an iodixanol (OptiPrep, Axis Shield) gradient solution. Obtained fractions were used for immunoblotting.

Wound healing assay

NIH/3T3 cells were seeded in a 96 plate (10,000 cell per well/ plate name: Essen Bioscience NO 4379). NIH/3T3 cells were grown till 80% confluence and then serum starved (0 % FCS) for 24 h. Scratch was performed using the Incucyte Woundmaker Tool. Next, 10 µM Rhonin was added to the wells. As controls, 2 µg/ml RHO inhibitor I (cat#CT04/ cytoskeleton) and 0.5% DMSO were used. Cell migration was monitored using IncuCyte ZOOM for a period of 48 h. The images were analyzed by the Incucyte Scratch Wound Analysis Software.

G-LISA activation assays for RHOA, RAC1 and CDC42

Colorimetric G-LISA activation assays were performed according to the manufacturer's instructions (Cytoskeleton, Inc.) to determine the levels of GTP-bound GTPases upon compound treatment. Briefly, HEK293 cells were treated with 10 µM Rhonin or DMSO as a control for 24 h. Cells were rapidly lysed (<10 min) using 1 mg/ml Triton X100. 1 mg/ml of each lysate was added to the pre-cold Microfuges. Microfuge tubes were cooled for 30 min at 4 °C. Samples were washed and incubated with 200 µl antigen presenting buffer for 2 min. GTPase primary antibody (RAC1, CDC42 or RHOA) was added and the tubes and were incubated for 45 min at room temperature. Afterwards, tubes were washed again and incubated with the secondary antibody for another 45 min at room temperature. Finally, tubes were washed and incubated with detection reagent for 20 min at room temperature. Absorption was detected at 490 nm using a plate reader (Tecan Sparks).

Solubility determination

Aqueous solubility of compounds was assessed by determination of the kinetic solubility of a test compound in aqueous buffer compared to a solution in organic solvent (acetonitrile). DMSO compound stock solutions were diluted in DMEM or HEPES buffer (pH 7.4) to achieve

500 μM final compound concentrations. The kinetic solubility of the compound solutions in HEPES buffer pH 7.4 were then compared to a solution in the organic solvent (ACN) after vigorous shaking on an orbital shaker at room temperature for 90 min. Therefore, the spectrum 250 -500 nm was recorded using the Spectramax plate reader. Precipitated material was removed by filtration prior to analysis. Each sample was tested in triplicate.

Aqueous solubility was also determined using a turbidimetric assay. DMSO stock solutions were diluted in DMSO to produce a range of concentrations. These were then added to PBS buffer pH7.4 (final test compound concentrations: 1 μM, 3 μM, 10 μM, 30 μM, 100 μM, 1% final DMSO concentration, 7 replicates per concentration). After 2 h incubation period at room temperature, the absorbance at 620 nm was read for each concentration and each replicate using a Spectramax plate reader.

Permeability through artificial membranes assay (PAMPA)

Permeability through artificial membranes (phospholipid/cholesterol in dodecane) was assessed at an initial compound concentration of 500 μM in the donor compartment. Absorption of the receiver wells was determined by spectrophotometrical measurement using a Spectramax absorbance plate reader after 20 h of incubation. Permeation was calculated by normalization of compound flux across a blank filter.

Limited Proteolysis Mass Spectrometry (LiP-MS) experiments

Recombinant RHOGDI protein (1 μg) in PBS (14.2 μL) was incubated with 100 μM Rhonin (0.8 μL of 30eq.) or DMSO for one hour at r.t. Proteinase K (NEB, 1 μL of 50x stock in PBS for final 1:100 w/w ratio to RHOGDI) was then added and incubated at 25 °C for exactly one min. Samples were then boiled for 10 min at 95 °C to inactivate proteinase K. Proteins were then denatured in 6 M urea in 50 mM NH_4HCO_3, reduced with 10 mM neutralized TCEP (200 mM fresh stock in water) for 30 minutes at r.t. and alkylated with 25 mM iodoacetamide (400 mM fresh stock in water) for 30 minutes at r.t. in the dark. Samples were diluted to 2 M urea with 50 mM NH_4HCO_3, and digested with trypsin (Thermo Scientific, 0.25 μg) in the presence of 1 mM $CaCl_2$ (100x stock in water). The digestion was performed for 4 hours at 37°C.

Samples were acidified to a final concentration of 5% acetic acid, desalted over a self-packed C_{18} spin column, and dried. Peptides were resuspended in water with 0.1 % formic acid (FA) and analyzed using EASY-nLC 1200 nano-UHPLC coupled to Q Exactive HF-X Quadrupole-Orbitrap mass spectrometer (Thermo Scientific). The chromatography column consisted of a 45 cm long, 75 μm i.d. microcapillary capped by a 5 μm tip and packed with ReproSil-Pur 120 C18-AQ 2.4 μm beads (Dr. Maisch GmbH). LC solvents were 0.1 % FA in water (Buffer A) and 0.1 % FA in 90 % MeCN: 10 % water (Buffer B). Peptides were eluted into the mass spectrometer at a flow rate of 300 nL/minute over a 30-minute linear gradient (5-35 % Buffer B) at 65 °C. Data was acquired in data-dependent mode (top-20, NCE 28, R = 7'500) after full MS scan (R = 60'000, m/z 400-1'300). Dynamic exclusion was set to 10 seconds with peptide match set to prefer and isotope exclusion enabled. The MS data was analyzed with MaxQuant[11] and searched against the human proteome (Uniprot), a common list of contaminants (included in MaxQuant) and the amino acid sequence of recombinant RHOGDI. The first peptide search tolerance was set to 20 ppm and the main peptide search was set to 10 ppm with fragment mass tolerance at 0.02 Da. The false discovery rate for peptides, proteins and sites identification was set to 1 %. The minimum peptide length was set to 6 amino acids and peptide requantification was enabled. The minimal number of peptides per protein was set to two. Carbamidomethylation of cysteine was set as fixed modification and oxidation of methionine as variable modification. Both trypsin and proteinase K were used as proteases with up to 10 miss-cleavages. Modified peptides with PEP value ≤1% were considered. Statistical significance analysis was performed using Perseus[12] under false discovery rate of 0.5% and an S_0 of 0.1, indicating an adjusted p-value of 0.05.

Catch-and-release mass spectrometry experiments

HEK293T lysate overexpressing RHOGDI-F (5 mg in 2.35 mL) was treated with DMSO or 100 μM Rhonin for 1 hour at r.t followed by treatment with 30 μM STPyne[13] (Lumiprobe, 40720, 100x stock in DMSO) for 1 hour at r.t. Click chemistry was initiated with the addition of photocleavable (PC) biotin azide (Click Chemistry Tools, 10 μM, 50x stock in DMSO), tris(2-carboxyethyl)phosphine hydrochloride (TCEP) (1 mM, 50x fresh stock in water), tris[(1-benzyl-1H-1,2,3-triazol-4-yl)methyl]amine (TBTA) (100 μM, 16x stock in DMSO:tBuOH 1:4), and copper(II) sulfate (1 mM, 50x stock in water) to the lysates and left to react for one hour at r.t. Protein was precipitated by adding methanol/chloroform/water (4:1:3 v/v) to the reaction mixture and the turbid mixture was centrifuged for 10 minutes at 20'000 x g at 4 °C

yielding a protein layer between the aqueous and organic layers. The protein layer was isolated, dried and solubilized in 2 % SDS in PBS via sonication. The light and heavy proteomes were mixed 1:1 and centrifuged at 4'700 x g for five minutes and the supernatant was transferred to a new tube. PBS was added to give a final SDS concentration of 0.2 %. Streptavidin agarose beads (500 µL) were added and the mixture was rotated for four hours at r.t. Beads were washed with 1 % SDS in PBS (2x 10 mL), PBS (3x 10 mL), and water (3x 10 mL). Beads were resuspended in 6 M urea in PBS (500 µL), reduced with 10 mM neutralized TCEP (20x fresh stock in water) for 30 minutes at r.t., and alkylated with 25 mM iodoacetamide (400 mM fresh stock in water) for 30 minutes at r.t. in the dark. Beads were pelleted by centrifugation (1'400 x g, two minutes) and resuspended in 300 µL of 2 M urea in 50 mM NH_4HCO_3, 1 mM $CaCl_2$ (100x stock in water) and trypsin (2 µL of 0.5 µg/µL, Thermo Scientific). The digestion was performed for six hours at 37 °C. Beads were washed with 1 % SDS in PBS (1x 10 mL) and PBS (5x 10 mL). Beads were resuspended in 1 mL PBS and photocleavage was performed with irradiation at 365 nm in a CL-1000 UV crosslinker (UVP) for one hour at 4 °C. Beads were centrifuged and supernatant was transferred to a new tube. Beads were resuspended in 1 mL PBS and incubated for one hour at 37 °C with shaking then centrifuged, and the supernatants were combined. Beads were again resuspended in 1 mL PBS with 1 M NaCl and incubated overnight at 37 °C with shaking then centrifuged and the supernatants were combined. Peptides were desalted over a self-packed C18 spin column and dried. Peptides were resuspended in water with 0.1 % formic acid (FA) and analyzed using EASY-nLC 1200 nano-UHPLC coupled to Q Exactive HF-X Quadrupole-Orbitrap mass spectrometer (Thermo Scientific). The chromatography column consisted of a 45 cm long, 75 µm i.d. microcapillary capped by a 5 µm tip and packed with ReproSil-Pur 120 C18-AQ 2.4 µm beads (Dr. Maisch GmbH). LC solvents were 0.1 % FA in water (Buffer A) and 0.1 % FA in 90 % MeCN: 10 % water (Buffer B). Peptides were eluted into the mass spectrometer at a flow rate of 300 nL/minute over a 90-minute linear gradient (5-35 % Buffer B) at 65 °C. Data was acquired in data-dependent mode (top-20, NCE 28, R = 7'500) after full MS scan (R = 60'000, m/z 400-1'300). Dynamic exclusion was set to 10 seconds with peptide match set to prefer and isotope exclusion enabled. The MS data was analyzed with MaxQuant[11] and searched against the human proteome (Uniprot) and a common list of contaminants (included in MaxQuant). The first peptide search tolerance was set to 20 ppm and the main peptide search was set to 10 ppm with fragment mass tolerance at 0.02 Da. The false discovery rate for peptides, proteins and sites identification was set to 1 %. The minimum peptide length was set to 6 amino acids and

peptide requantification was enabled. For lysine site identification, the minimal number of peptides per protein was set to one. STPyne PC cleaved adducts (+180.1011 Da) on lysines and oxidation of methionine were set as variable modifications and carbamidomethylation on cysteine as fixed modification. Trypsin was used as protease allowing up to 2 miss-cleavages. Modified peptides with PEP value ≤1% were considered.

Computational Methods

Computational docking and simulations were performed using the Maestro environment, version 12.7.156, with the Schrodinger suite of software, release 2021-1 (Schrödinger Inc., USA). Structure of RHOGDI1 was extracted from PDB entry 1HH4 (chain D of the RAC1-RHOGDI1 complex). Protein preparation was done with the Protein Preparation Wizard (Schrödinger), where all waters associated with the chain D were kept, and the missing loop fragments and side chains were added with Prime.[14] The amino acid protonation states were refined using PROPKA at pH set to 7.0.[15] Restrained minimisation was applied using OPLS4 force field and the heavy atom convergence was set to 0.3 Å RMSD.[16] The ligand was prepared for docking using LigPrep (Schrödinger), with OPLS4 force field and ionisation at pH 7.0 ± 2.0 with Epik (v5.5) generating possible tautomeric states.[17]

DFT energy calculations (B3LYP/6-31G**, Jaguar v11.1, Schrödinger Inc.) in the gas phase for geometry optimised conformations of the tautomers revealed 9.27 kcal/mol energy difference favouring the imine-like structure, thus, indicating the imine to be the major tautomer in the absence of additional interactions.[18] Despite the imine being the predominant form, it is likely that the situation in the complex is different than in solution as discussed by Pospisil *et al.* and Martin.[19] Considering the above reasoning and the lack of additional information regarding which tautomer is the most relevant in the binding site, both imine-like and enamine-like structures were submitted for the induced fit docking (IFD; Schrödinger) with the prepared RHOGDI1 protein.[20] The docking grid was centred in the binding pocket of the geranylgeranyl group, and the Glide (v9.0) docking was set to XP.[21] Ligand in a pose with the best IFD Score was then used for a molecular dynamics (MD) simulation. Further computational-based investigation of the ligand-protein interactions through molecular dynamics (MD) simulations was conducted using the highest scoring pose (IFD Score and

GScore) of ligand which was in line with the structure-activity experimental results obtained for the synthesized compounds.

The flexible, unstructured N-terminal sequence of RHOGDI1 with a high B-factor (up to residue 30) was removed before commencing the MD simulation.[22] The calculations were performed with Desmond (D.E. Shaw Research, USA; Schrödinger),[23] using NVIDIA GeForce RTX 2070 GPU. System Builder (Schrödinger) was used to prepare the protein-ligand complex for the simulation. The SPC solvent model was applied,[24] with an orthorhombic box shape, a buffer padding of 15 Å, and the OPLS4 force field. Charges were neutralised with Na^+ ions, and NaCl was added at a concentration of 0.15 M, in order to mimic the physiological conditions. The simulation time was set to 120 ns, with recording intervals of 25 ps for the trajectory and 1.2 ps for the energy. The NPT ensemble was chosen, with the temperature set to 300 K and the pressure to 1.01325 bar. The RESPA integrator step was 2 fs, and the Nose-Hoover chain thermostat (relaxation time 1.0 ps) as well as the Martyna-Tobias-Klein barostat (relaxation time 2.0 ps) were used. The system was relaxed with the Maestro's build-in relaxation protocol before the commencement of the production run. The obtained results were analysed using the Simulation Quality Analysis and Simulation Interactions Diagram tools (Schrödinger). The MD studies were performed in duplicate, with both runs giving closely similar results. The protein-ligand interaction results are presented for one of the replicates.

Physical Characterization data

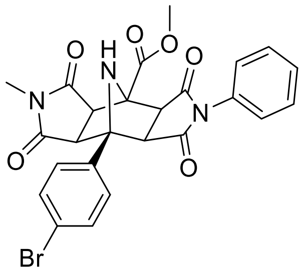

Methyl (3aR,4R,7aR,8S,8aS)-8-(4-bromophenyl)-2-methyl-1,3,5,7-tetraoxo-6-phenyldecahydro-4,8-epiminopyrrolo[3,4-f]isoindole-4(1H)-carboxylate

5a was obtained as amorphous colourless solid in 48% yield according to GP-1. ^{1}H NMR (500 MHz, $CDCl_3$) δ 7.61-7.56 (m, 2H), 7.40-7.26 (m, 4H), 7.11 (m, 3H), 3.96 (s, 3H), 3.59 (d, J = 7.0 Hz, 1H), 3.53 (d, J = 6.9 Hz, 1H), 3.43 (d, J = 6.9 Hz, 1H), 3.39 (d, J = 7.0 Hz, 1H), 2.87 (s, 3H) ppm; ^{13}C NMR (126 MHz, $CDCl_3$) δ 172.54, 171.93, 171.57, 170.66, 166.50, 130.94, 130.26, 130.15, 129.27, 128.25, 128.02, 126.18, 125.17, 122.16, 73.84, 71.76, 52.97, 52.76, 52.12, 51.45, 24.58 ppm; FT-IR: $\tilde{\nu}$ = 3296, 2975, 2159, 2030, 1976, 1701, 1596, 1493, 1434, 1288, 1245, 1191, 783 cm^{-1}; HRMS: calc. for $[M+H]^+$ $C_{25}H_{21}O_6N_3{}^{79}Br$: 538.06082, found: 538.06056; $C_{25}H_{21}O_6N_3{}^{81}Br$: 540.05878, found: 540.05835; $[\alpha]_D^{RT}$= +2.6 ($CHCl_3$, c = 0.5); HPLC conditions: CHIRAPAK IC column, (CH_2Cl_2/EtOH = 100/2) / *iso*-hexane = 70/30, flow rate = 0.5 mL min^{-1}, major enantiomer: t_R = 11.9 min; minor enantiomer: t_R = 13.5 min.

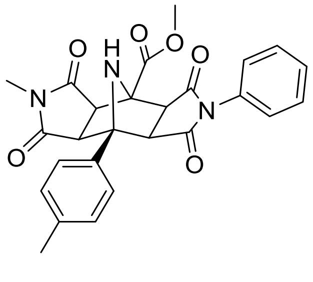

Methyl (3aR,4R,7aR,8S,8aS)-2-methyl-1,3,5,7-tetraoxo-6-phenyl-8-(p-tolyl)decahydro-4,8-epiminopyrrolo[3,4-f]isoindole-4(1H)-carboxylate

5b was obtained as amorphous colourless solid in 44% yield according to GP-1. ^{1}H NMR (500 MHz, $CDCl_3$) δ 7.57 (br s, 1H), 7.37 (t, J = 7.5 Hz, 2H), 7.33 (br s, 1H), 7.31 (t, J = 7.5 Hz, 1H), 7.19-7.08 (m, 4H), 4.00 (s, 3H), 3.60 (d, J = 6.7 Hz, 1H), 3.53 (d, J = 6.7 Hz, 1H), 3.43 (d, J = 6.8 Hz, 1H), 3.40 (d, J = 6.8 Hz, 1H), 2.88 (s, 3H), 2.70 (br s, 1H), 2.35 (s, 3H) ppm; ^{13}C NMR (126 MHz, $CDCl_3$) δ 173.69, 173.01, 172.71, 171.71, 167.49, 138.70, 131.41,

129.18, 129.09, 128.87, 126.22, 75.36, 72.82, 54.05, 53.91, 53.06, 53.01, 52.54, 25.51, 21.54 ppm; FT-IR: $\widetilde{\nu}$ = 3302, 2970, 2159, 1712, 1597, 1435, 1336, 1246, 783 cm^{-1}; HRMS: calc. for $[M+H]^+$ $C_{26}H_{24}O_6N_3$ = 474.16596, found: 474.16534; $[\alpha]_D^{RT}$ = +5.4 ($CHCl_3$, *c* = 0.5); HPLC conditions: CHIRAPAK IC column, (CH_2Cl_2/EtOH = 100/2) / *iso*-hexane = 70/30, flow rate = 0.5 mL min^{-1}, major enantiomer: t_R = 18.5 min; minor enantiomer: t_R = 16.5 min.

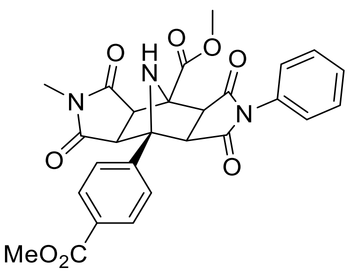

Methyl (3aR,4R,7aR,8S,8aS)-8-(4-(methoxycarbonyl)phenyl)-2-methyl-1,3,5,7-tetraoxo-6-phenyldecahydro-4,8-epiminopyrrolo[3,4-f]isoindole-4(1H)-carboxylate

5c was obtained as amorphous colourless solid in 65% yield according to GP-1. ^1H NMR (300 MHz, $(CD_3)_2CO$) δ 8.01-7.87 (m, 3H), 7.49-7.46 (m, 1H), 7.37-7.31 (m, 3H), 7.15-7.11 (m, 2H), 3.91-3.77 (m, 4H), 3.88 (s, 3H), 3.86 (s, 3H), 2.71 (s, 3H) ppm; ^{13}C NMR (176 MHz, $CDCl_3$) δ 173.44, 172.79, 172.45, 171.52, 167.29, 166.64, 137.26, 131.20, 130.67, 130.29, 130.02, 129.24, 129.01, 128.90, 126.18, 125.45, 75.01, 72.88, 54.03, 53.88, 53.13, 53.03, 52.47, 52.28, 25.59 ppm; FT-IR: $\widetilde{\nu}$ = 2159, 2030, 1699, 1597, 1435, 1280, 1111, 852, 748 cm^{-1}; HRMS: calc. for $[M+H]^+$ $C_{27}H_{24}O_8N_3$: 518.15579, found: 518.15527; $[\alpha]_D^{RT}$ = +1.4 (acetone, *c* = 0.5); HPLC conditions: CHIRAPAK IC column, (CH_2Cl_2/EtOH = 100/2) / *iso*-hexane = 70/30, flow rate = 0.5 mL min^{-1}, major enantiomer: t_R = 18.5 min; minor enantiomer: t_R = 22.7 min.

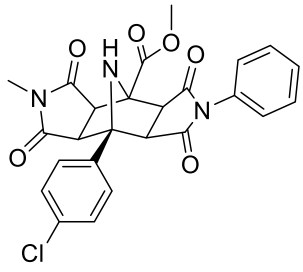

Methyl (3aR,4R,7aR,8S,8aS)-8-(4-chlorophenyl)-2-methyl-1,3,5,7-tetraoxo-6-phenyldecahydro-4,8-epiminopyrrolo[3,4-f]isoindole-4(1H)-carboxylate

5d was obtained as amorphous colourless solid in 55% yield according to GP-1. ^{1}H NMR (600 MHz, DMSO) δ 7.82 (d, *J* = 7.4 Hz, 1H), 7.42 (t, *J* = 7.7 Hz, 3H), 7.35 (t, *J* = 7.4 Hz, 1H), 7.26 (br, 2H), 7.14 – 7.05 (m, 2H), 4.53 (br, 1H), 3.78 (s, 3H), 3.75 (d, *J* = 6.9 Hz, 1H), 3.70 – 3.65 (m, 2H), 3.63 (d, *J* = 6.9 Hz, 1H), 2.65 (s, 3H) ppm; ^{13}C NMR (151 MHz, DMSO) δ 174.66, 174.07, 173.71, 172.89, 167.82, 133.48, 132.02, 131.69, 131.37, 128.59, 128.19, 127.89, 127.28, 126.73, 73.94, 71.84, 53.12, 52.98, 52.87, 52.59, 51.70, 24.80 ppm; FT-IR: $\widetilde{\nu}$ = 3263, 2972, 2159, 2030, 1706, 1597, 1494, 1434, 1290, 1180, 786 cm^{-1}; HRMS: calc. for $[M+H]^+$ $C_{25}H_{21}O_6N_3Cl$ = 494.11134, found: 494.11095; calc. for $[M+H]^+$ $C_{25}H_{21}O_6N_3{}^{37}Cl$ = 496.10839, found: 496.10817; $[\alpha]_D^{RT}$ = +5.8 ($CHCl_3$, *c* = 0.5); HPLC conditions: CHIRAPAK IC column, (CH_2Cl_2/EtOH = 100/2) / *iso*-hexane = 70/30, flow rate = 0.5 mL min^{-1}, major enantiomer: t_R = 9.8 min; minor enantiomer: t_R = 11.3 min.

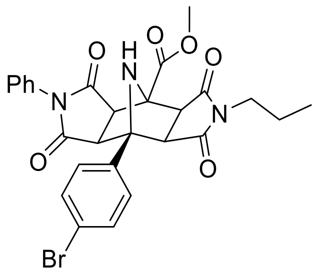

Methyl (3aR,4S,7aR,8R,8aS)-8-(4-bromophenyl)-1,3,5,7-tetraoxo-2-phenyl-6-propyldecahydro-4,8-epiminopyrrolo[3,4-f]isoindole-4(1H)-carboxylate

5e was obtained as amorphous colourless solid in 46% yield according to GP-1. ^{1}H NMR (500 MHz, CD_3CN) δ 7.65-7-65 (m, 1H), 7.58-7.57 (m, 1H), 7.43-7.40 (m, 3H), 7.37-7.34 (m, 1H), 7.18-7.16 (m, 1H), 7.12-7.10 (m, 2H), 3.85 (s, 3H), 3.66 (d, *J* = 7.1 Hz, 1H), 3.56 (dd, *J* = 7.0, 1.6 Hz, 2H), 3.51 (d, *J* = 7.0 Hz, 1H), 3.32 (br s, 1H), 3.28-3.19 (m, 2H), 1.42 (tq, J = 7.3, 7.3 Hz, 2H), 0.79 (t, *J* = 7.5 Hz, 3H) ppm; ^{13}C NMR (176 MHz, $CDCl_3$) δ 173.58, 172.87, 172.57, 171.67, 167.34, 131.91, 131.39, 131.34, 131.22, 130.33, 129.26, 129.03, 126.98, 126.17, 123.18, 74.85, 72.89, 53.77, 53.72, 53.06, 52.75, 52.61, 41.07, 21.12, 11.29 ppm; FT-IR: $\widetilde{\nu}$ = 3292, 2970, 2159, 2030, 1699, 1596, 1494, 1190, 1013, 748 cm^{-1}; HRMS: calc. for $[M+H]^+$ $C_{27}H_{25}O_6N_3{}^{79}Br$ = 566.09212, found: 566.09148; calc. for $[M+H]^+$ $C_{27}H_{25}O_6N_3{}^{81}Br$ = 568.09008, found: 568.08954; $[\alpha]_D^{RT}$ = +11.6 ($CHCl_3$, *c* = 0.5); HPLC conditions: CHIRAPAK IC column, (CH_2Cl_2/EtOH = 100/2) / *iso*-hexane = 70/30, flow rate = 0.5 mL min^{-1}, major enantiomer: t_R = 9.8 min; minor enantiomer: t_R = 9.1 min.

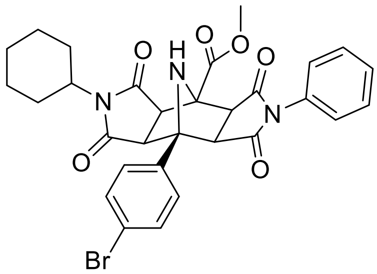

Methyl (3aR,4R,7aR,8S,8aS)-8-(4-bromophenyl)-2-cyclohexyl-1,3,5,7-tetraoxo-6-phenyldecahydro-4,8-epiminopyrrolo[3,4-f]isoindole-4(1H)-carboxylate

5f was obtained as amorphous colourless solid in 43% yield according to GP-1. ^{1}H NMR (500 MHz, $CDCl_3$) δ 7.62 (s, 1H), 7.55 (s, 1H), 7.49 – 7.42 (m, 1H), 7.39 (t, *J* = 7.5 Hz, 2H), 7.33 (t, *J* = 7.4 Hz, 1H), 7.18 – 7.07 (m, 3H), 4.00 (s, 3H), 3.81 (tt, *J* = 12.3, 3.7 Hz, 1H), 3.60 (d, *J* = 6.9 Hz, 1H), 3.46 (d, *J* = 6.9 Hz, 1H), 3.42 (d, *J* = 6.9 Hz, 1H), 3.31 (d, *J* = 6.9 Hz, 1H), 2.06 – 1.87 (m, 2H), 1.75 (d, *J* = 13.0 Hz, 2H), 1.58 (d, *J* = 12.3 Hz, 1H), 1.49 – 1.40 (m, 2H), 1.26 – 1.10 (m, 3H) ppm; ^{13}C NMR (126 MHz, $CDCl_3$) δ 173.62, 172.83, 172.51, 171.63, 167.40, 131.97, 131.35, 131.31, 131.15, 130.20, 129.27, 129.04, 126.96, 126.09, 123.21, 74.92, 72.95, 53.67, 53.22, 53.05, 52.59, 52.34, 52.33, 28.94, 28.64, 25.79, 25.76, 24.88 ppm; FT-IR: $\widetilde{\nu}$ = 2969, 2159, 2029, 1712, 1693, 1595, 1492, 1192, 749 cm^{-1}; HRMS: calc. for $[M+H]^+$ $C_{30}H_{29}O_6N_3{}^{79}Br$ = 606.12342, found: 606.12315; calc. for $[M+H]^+$ $C_{30}H_{29}O_6N_3{}^{81}Br$ = 608.12138, found: 608.12119; $[\alpha]_D^{RT}$ = +2.4 ($CHCl_3$, *c* = 0.5); HPLC conditions: CHIRAPAK IC column, (CH_2Cl_2/EtOH = 100/2) / *iso*-hexane = 70/30, flow rate = 0.5 mL min^{-1}, major enantiomer: t_R = 8.5 min; minor enantiomer: t_R = 9.8 min.

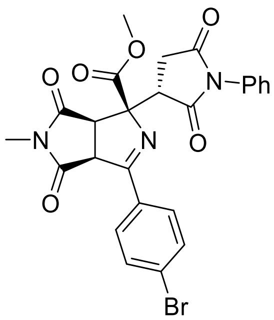

Methyl (1S,3aS,6aR)-3-(4-bromophenyl)-1-((S)-2,5-dioxo-1-phenylpyrrolidin-3-yl)-5-methyl-4,6-dioxo-1,3a,4,5,6,6a-hexahydropyrrolo[3,4-c]pyrrole-1-carboxylate

6a was obtained as amorphous colourless solid in 69% yield according to GP-2 using Et_3N (1.5 equiv) and *N*-phenylmaleimide (1.5 equiv). ^{1}H NMR (500 MHz, $CDCl_3$) δ 8.10 (d, *J* = 8.4 Hz,

2H), 7.65 (d, J = 8.4 Hz, 2H), 7.44 (t, J = 7.4 Hz, 2H), 7.38 (t, J = 7.3 Hz, 1H), 7.33 (d, J = 7.5 Hz, 2H), 4.78 – 4.59 (m, 2H), 3.66 (s, 3H), 3.12 – 3.03 (m, 1H), 3.01 – 2.90 (m, 5H) ppm; ^{13}C NMR (126 MHz, $CDCl_3$) δ 175.43, 174.84, 174.34, 171.89, 169.27, 168.82, 132.13, 131.96, 131.67, 130.13, 129.29, 128.83, 127.53, 126.80, 86.19, 57.38, 53.45, 51.19, 46.71, 32.99, 25.49 ppm; FT-IR: $\tilde{\nu}$ = 2973, 2159, 2030, 1976, 1700, 1498, 1288, 1180, 1011, 695 cm^{-1}; HRMS: calc. for $[M+H]^+$ $C_{25}H_{21}N_3O_6{}^{79}Br$: 538.06082 found: 538.06217 $C_{25}H_{21}N_3O_6{}^{81}Br$: 540.05878 found: 540.06018. $[\alpha]_D^{RT}$= +128 ($CHCl_3$, c = 0.5); HPLC conditions: CHIRAPAK IC column, (CH_2Cl_2/EtOH = 100/2) / *iso*-hexane = 70/30, flow rate = 0.5 mL min^{-1}, major enantiomer: t_R = 18.2 min; minor enantiomer: t_R = 11.3 min.

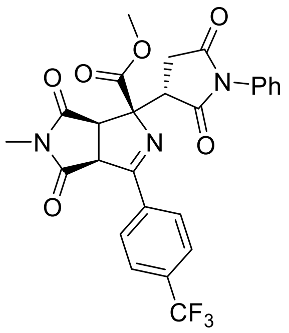

Methyl (1S,3aS,6aR)-1-((S)-2,5-dioxo-1-phenylpyrrolidin-3-yl)-5-methyl-4,6-dioxo-3-(4-(trifluoromethyl)phenyl)-1,3a,4,5,6,6a-hexahydropyrrolo[3,4-c]pyrrole-1-carboxylate

6b was obtained as amorphous colourless solid in 57% yield according to GP-2 using Et_3N (1.5 equiv) and *N*-phenylmaleimide (1.5 equiv). ^{1}H NMR (300 MHz, $CDCl_3$) δ 8.35 (d, J = 8.1 Hz, 2H), 7.78 (d, J = 8.1 Hz, 2H), 7.37-7.34 (m, 3H), 7.29-7.25 (m, 2H), 4.74-4.68 (m, 2H), 3.67 (s, 3H), 3.06 (dd, J = 15.8, 2.7 Hz, 1H), 2.99-2.88 (m, 2H), 2.92 (s, 3H) ppm; ^{13}C NMR (75 MHz, $CDCl_3$) δ 175.65, 174.82, 174.65, 171.98, 169.37, 168.94, 134.40, 133.87 (q, J = 32.7 Hz), 130.61, 129.31, 128.91, 126.87, 125.60 (q, J = 3.6 Hz), 123.8 (q, J = 270.2 Hz), 86.12, 57.63, 53.51, 51.33, 46.50, 33.01, 25.41 ppm; FT-IR: $\tilde{\nu}$= 2972, 2159, 1700, 1597, 1499, 1316, 1289, 1171, 1126, 1017, 853, 730 cm^{-1}; HRMS: calc. for $[M+H]^+$ $C_{26}H_{21}N_3O_6 F_3$: 528.13770 found: 528.13866; $[\alpha]_D^{RT}$= +103 ($CHCl_3$, c = 0.5); HPLC conditions: CHIRAPAK IC column, (CH_2Cl_2/EtOH = 100/2) / *iso*-hexane = 70/30, flow rate = 0.5 mL min^{-1}, major enantiomer: t_R = 14.6 min; minor enantiomer: t_R = 9.3 min.

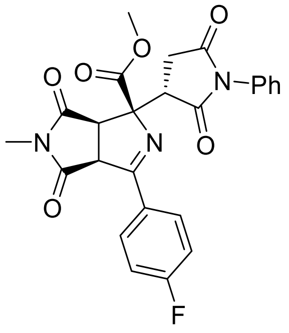

Methyl (1S,3aS,6aR)-1-((S)-2,5-dioxo-1-phenylpyrrolidin-3-yl)-3-(4-fluorophenyl)-5-methyl-4,6-dioxo-1,3a,4,5,6,6a-hexahydropyrrolo[3,4-c]pyrrole-1-carboxylate

6c was obtained as amorphous colourless solid in 72% yield according to GP-2 using Et_3N (1.5 equiv) and *N*-phenylmaleimide (1.5 equiv). ^{1}H NMR (500 MHz, $CDCl_3$) δ 8.25-8.18 (m, 2H), 7.39-7.23 (m, 5H), 7.19 (t, J = 8.0 Hz, 2H), 4.76-4.58 (m, 2H), 3.65 (s, 3H), 3.02 (d, J = 16.2 Hz, 1H), 2.97-2.76 (m, 2H), 2.90 (s, 3H) ppm; ^{13}C NMR (126 MHz, $CDCl_3$) δ 175.72, 174.89, 174.83, 172.27, 169.29, 169.08, 165.37 (d, J = 254.2 Hz), 132.62 (d, J = 8.9 Hz), 132.23, 129.24, 128.78, 127.70 (d, J = 3.0 Hz), 126.94, 115.77 (d, J = 21.8 Hz), 85.82, 57.79, 53.34, 51.60, 46.73, 33.08, 25.33 ppm; FT-IR: $\tilde{\nu}$ = 2973, 2159, 2030, 1700, 1600, 1450, 1434, 1345, 1288, 1236, 849, 749 cm^{-1}; HRMS: calc. for $[M+H]^+$ $C_{25}H_{21}N_3O_6F$: 478.14089 found: 478.14189. $[\alpha]_D^{RT}$ = +123 ($CHCl_3$, c = 0.5); HPLC conditions: CHIRAPAK IC column, (CH_2Cl_2/EtOH = 100/2) / *iso*-hexane = 70/30, flow rate = 0.5 mL min^{-1}, major enantiomer: t_R = 16.1 min; minor enantiomer: t_R = 10.2 min.

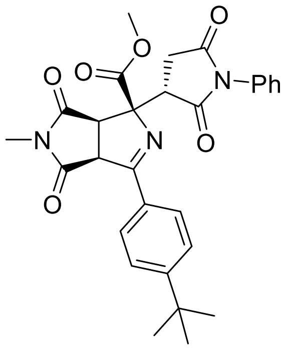

Methyl (1S,3aS,6aR)-3-(4-(tert-butyl)phenyl)-1-((S)-2,5-dioxo-1-phenylpyrrolidin-3-yl)-5-methyl-4,6-dioxo-1,3a,4,5,6,6a-hexahydropyrrolo[3,4-c]pyrrole-1-carboxylate

6d was obtained as amorphous colourless solid in 75% yield according to GP-2 using Et_3N (1.5 equiv) and *N*-phenylmaleimide (1.5 equiv). ^{1}H NMR (500 MHz, $CDCl_3$) δ 8.16 (d, J = 8.6 Hz, 2H), 7.53 (d, J = 8.6 Hz, 2H), 7.40-7.35 (m, 3H), 7.32-7.30 (m, 2H), 4.72 (d, J = 8.7 Hz, 1H),

4.67-4.65 (m, 1H), 3.65 (s, 3H), 3.06 (d, J = 13.7 Hz, 1H), 2.94-2.84 (m, 2H), 2.91 (s, 3H), 1.36 (s, 9H) ppm; ^{13}C NMR (126 MHz, $CDCl_3$) δ 175.69, 175.07, 174.77, 172.29, 169.91, 169.32, 156.13, 132.27, 130.07, 129.24, 128.73, 128.60, 126.91, 126.57, 125.62, 85.95, 57.47, 53.28, 51.32, 46.81, 44.94, 35.21, 33.03, 31.25, 25.31 ppm; FT-IR: $\tilde{\nu}$ = 2969, 1702, 1597, 1498, 1431, 1288, 1246, 1176, 1126, 822, 748 cm^{-1}; HRMS: calc. for $[M+H]^+$ $C_{29}H_{30}O_6N_3$: 516.21291, found: 516.21238; $[\alpha]_D^{RT}$ = +111 ($CHCl_3$, c = 0.5); HPLC conditions: CHIRAPAK IC column, (CH_2Cl_2/EtOH = 100/2) / *iso*-hexane = 70/30, flow rate = 0.5 mL min^{-1}, major enantiomer: t_R = 13.6 min; minor enantiomer: t_R = 9.1 min.

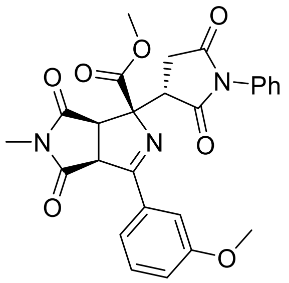

Methyl (1S,3aS,6aR)-1-((S)-2,5-dioxo-1-phenylpyrrolidin-3-yl)-3-(3-methoxyphenyl)-5-methyl-4,6-dioxo-1,3a,4,5,6,6a-hexahydropyrrolo[3,4-c]pyrrole-1-carboxylate

6e was obtained as amorphous colourless solid in 72% yield according to GP-2 using Et_3N (1.5 equiv) and *N*-phenylmaleimide (1.5 equiv). ^{1}H NMR (500 MHz, $CDCl_3$) δ 7.81 (d, J = 7.8 Hz, 1H), 7.79-7.78 (m, 1H), 7.42 (t, J = 8.0 Hz, 1H), 7.39-7.34 (m, 3H), 7.30-7.28 (m, 2H), 7.10 (dd, J = 8.2, 2.0 Hz, 1H), 4.69-4.65 (m, 2H), 3.89 (s, 3H), 3.65 (s, 3H), 3.05 (dd, J = 16.3, 3.0 Hz, 1H), 2.90 (s, 3H), 2.88-2.82 (m, 2H) ppm; ^{13}C NMR (126 MHz, $CDCl_3$) δ 175.78, 175.02, 174.93, 172.27, 170.17, 169.35, 159.68, 132.66, 132.26, 129.58, 129.25, 128.77, 126.92, 122.91, 118.78, 114.68, 85.76, 57.71, 55.60, 53.28, 51.48, 46.60, 33.02, 25.23 ppm; FT-IR: $\tilde{\nu}$ = 2955, 2516, 2159, 2030, 1976, 1699, 1580, 1433, 1384, 1334, 1180, 750, 692 cm^{-1}; HRMS: calc. for $[M+H]^+$ $C_{26}H_{24}N_3O_7$: 490.16088 found: 490.16213; $[\alpha]_D^{RT}$ = +128 ($CHCl_3$, c = 0.5); HPLC conditions: CHIRAPAK IC column, (CH_2Cl_2/EtOH = 100/2) / *iso*-hexane = 70/30, flow rate = 0.5 mL min^{-1}, major enantiomer: t_R = 17.7 min; minor enantiomer: t_R = 10.8 min.

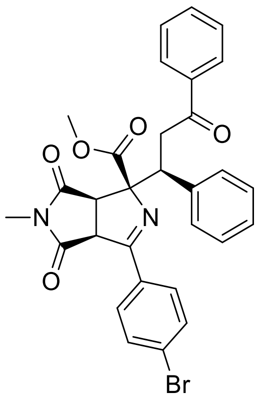

Methyl (1S,3aS,6aR)-3-(4-bromophenyl)-5-methyl-4,6-dioxo-1-((S)-3-oxo-1,3-diphenylpropyl)-1,3a,4,5,6,6a-hexahydropyrrolo[3,4-c]pyrrole-1-carboxylate

7a was obtained as amorphous colourless solid in 35% combined yield in a 2:1 dr according to the GP-2 using DBU (0.5 equiv) and chalcone (1.5 equiv). Data for the major diastereomer. ^{1}H NMR (500 MHz, $CDCl_3$) δ 7.98 (d, *J* = 8.6 Hz, 2H), 7.94 – 7.90 (m, 2H), 7.63 (d, *J* = 8.6 Hz, 2H), 7.52 (t, *J* = 7.4 Hz, 1H), 7.42 (t, *J* = 7.7 Hz, 2H), 7.19 – 7.14 (m, 1H), 7.13 – 7.04 (m, 4H), 4.50 (dd, *J* = 11.1, 2.6 Hz, 1H), 3.86 – 3.77 (m, 4H), 3.62 (dd, *J* = 17.4, 2.6 Hz, 1H), 3.54 (d, *J* = 8.2 Hz, 1H), 3.09 (d, *J* = 8.2 Hz, 1H), 2.84 (s, 3H) ppm; ^{13}C NMR (176 MHz, $CDCl_3$) δ 197.54, 175.12, 171.96, 170.53, 169.47, 137.85, 136.88, 133.19, 131.94, 131.26, 130.40, 130.37, 128.67, 128.49, 128.26, 127.76, 127.03, 87.64, 56.58, 53.22, 52.38, 49.20, 41.17, 25.40 ppm; FT-IR: $\widetilde{\nu}$ = 2974, 2159, 2030, 1976, 1698, 1592, 1489, 1431, 1248, 1176, 857, 747 cm^{-1}; HRMS: calc. for $[M+H]^{+}$ $C_{30}H_{26}N_2O_5{}^{79}Br$: 573.10196 found: 573.10388; $C_{30}H_{26}N_2O_5{}^{81}Br$: 575. 09991 found: 575. 10203. $[\alpha]_D^{RT}$ = +155 ($CHCl_3$, *c* = 0.5); HPLC conditions: CHIRAPAK IC column, (CH_2Cl_2/EtOH = 100/2) / *iso*-hexane = 80/20, flow rate = 0.5 mL min^{-1}, major enantiomer: t_R = 18.1 min; minor enantiomer: t_R = 7.2 min.

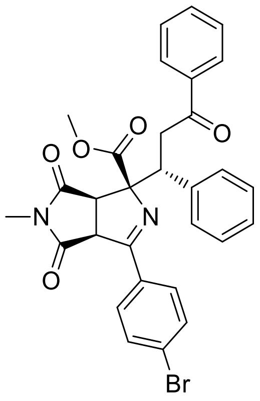

Methyl (1S,3aS,6aR)-3-(4-bromophenyl)-5-methyl-4,6-dioxo-1-((R)-3-oxo-1,3-diphenylpropyl)-1,3a,4,5,6,6a-hexahydropyrrolo[3,4-c]pyrrole-1-carboxylate

7a-epi Data for the minor diastereomer. ^{1}H NMR (500 MHz, $CDCl_3$) δ 7.98 (d, *J* = 8.6 Hz, 4H), 7.56 (dd, *J* = 8.9, 7.1 Hz, 3H), 7.45 (t, *J* = 7.7 Hz, 2H), 7.32 (d, *J* = 7.3 Hz, 2H), 7.16 (t, *J* = 7.4 Hz, 2H), 7.10 (dd, *J* = 8.3, 6.2 Hz, 1H), 4.56 (dd, *J* = 9.6, 3.6 Hz, 1H), 4.18 (d, *J* = 9.1 Hz, 1H), 3.84 – 3.75 (m, 2H), 3.71 (dd, *J* = 17.3, 3.7 Hz, 1H), 3.63 (s, 3H), 2.97 (s, 3H) ppm; ^{13}C NMR (176 MHz, $CDCl_3$) δ 197.59, 175.65, 172.50, 169.77, 166.97, 138.45, 136.99, 133.33, 131.81, 131.38, 130.50, 129.82, 128.78, 128.35, 128.21, 127.44, 126.93, 88.74, 57.69, 53.06, 50.17, 46.93, 39.68, 25.52 ppm; FT-IR: $\widetilde{\nu}$ = 2923, 2159, 1743, 1699, 1590, 1490, 1434, 1285, 1011, 809, 735 cm^{-1}; HRMS: calc. for $[M+H]^+$ $C_{30}H_{26}N_2O_5{}^{79}Br$: 573.10196 found: 573.10209; $C_{30}H_{26}N_2O_5{}^{81}Br$: 575. 09991 found: 575.09982; $[\alpha]_D^{RT}$ = +135 ($CHCl_3$, *c* = 0.2).

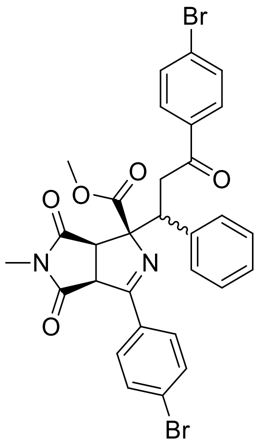

Methyl (1S,3aS,6aR)-3-(4-bromophenyl)-1-(3-(4-bromophenyl)-3-oxo-1-phenylpropyl)-5-methyl-4,6-dioxo-1,3a,4,5,6,6a-hexahydropyrrolo[3,4-c]pyrrole-1-carboxylate

7b was obtained as light yellow oil in 29% yield according to the GP-2 using DBU (0.5 equiv) and chalcone (1.5 equiv). ^{1}H NMR (500 MHz, $CDCl_3$) δ 7.98 (d, *J* = 7.1 Hz, 2H), 7.78 (d, *J* = 7.1 Hz, 2H), 7.63 (d, *J* = 7.5 Hz, 2H), 7.15-7.19 (m, 1H), 7.10 (t, *J* = 7.5 Hz, 2H), 7.04 (d, *J* = 7.2 Hz, 2H), 4.46 (dd, *J* = 11.1, 2.8 Hz, 1H), 3.78 (s, 3H), 3.76-3.71 (m, 1H), 3.60 (dd, *J* = 17.2, 2.7 Hz, 1H), 3.52 (d, *J* = 8.2 Hz, 1H), 3.12 (d, *J* = 8.2 Hz, 1H), 2.84 (s, 3H) ppm; ^{13}CNMR (126 MHz, $CDCl_3$) δ 196.44, 174.85, 171.65, 170.24, 169.55, 137.41, 135.36, 131.81, 131.12, 130.11, 130.05, 129.64, 128.38, 128.19, 127.71, 127.02, 87.31, 56.36, 53.08, 52.11, 49.01, 41.02, 25.23 ppm; HRMS: calc. for $[M+H]^+$ $C_{30}H_{25}O_5N_2{}^{79}Br_2$: 651.01247 found: 651.01254; $C_{30}H_{25}O_5N_2{}^{79}Br^{81}Br$: 653.01043 found: 653.01027.

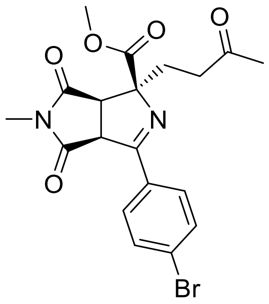

Methyl (1S,3aS,6aR)-3-(4-bromophenyl)-5-methyl-4,6-dioxo-1-(3-oxobutyl)-1,3a,4,5,6,6a-hexahydropyrrolo[3,4-c]pyrrole-1-carboxylate

7c was obtained as amorphous colourless solid in 60% yield according to the GP-2 using DBU (0.5 equiv) and buten-3-one (1.5 equiv). ^{1}H NMR (600 MHz, $CDCl_3$) δ 8.07 – 7.99 (m, 2H), 7.64 – 7.53 (m, 2H), 4.67 (d, J = 8.7 Hz, 1H), 3.67 (s, 3H), 3.53 (d, J = 8.6 Hz, 1H), 2.94 (s, 3H), 2.82 (ddd, J = 18.0, 9.2, 6.1 Hz, 1H), 2.71 (ddd, J = 18.0, 9.2, 5.4 Hz, 1H), 2.53 (ddd, J = 14.4, 9.2, 5.4 Hz, 1H), 2.18 – 2.08 (m, 4H) ppm; ^{13}C NMR (151 MHz, $CDCl_3$) δ 207.82, 175.00, 172.31, 169.94, 167.13, 131.89, 131.44, 130.44, 127.09, 84.39, 56.97, 53.40, 53.02, 38.90, 33.75, 30.19, 25.47 ppm; FT-IR: $\tilde{\nu}$ = 3302, 2972, 1750, 1698, 1593, 1490, 1431, 1345, 1286, 1245, 1178, 856, 765 cm^{-1}; HRMS: calc. for $[M+H]^+$ $C_{19}H_{20}O_5N_2{}^{79}Br$ = 435.05501, found: 435.05503; calc. for $[M+H]^+$ $C_{19}H_{20}O_5N_2{}^{81}Br$ = 437.05296, found: 437.05293; $[\alpha]_D^{RT}$ = +190 ($CHCl_3$, c = 0.5); HPLC conditions: CHIRAPAK IC column, (CH_2Cl_2/EtOH = 100/2) / *iso*-hexane = 40/60, flow rate = 0.5 mL min^{-1}, major enantiomer: t_R = 61.9 min; minor enantiomer: t_R = 56.0 min.

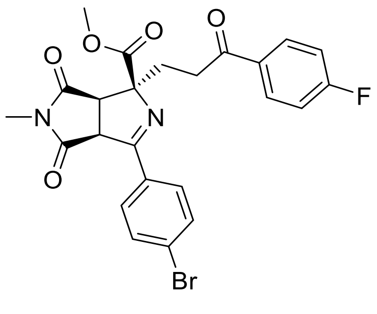

Methyl (1S,3aS,6aR)-3-(4-bromophenyl)-1-(3-(4-fluorophenyl)-3-oxopropyl)-5-methyl-4,6-dioxo-1,3a,4,5,6,6a-hexahydropyrrolo[3,4-c]pyrrole-1-carboxylate

7d was obtained as amorphous colourless solid in 60% yield according to the GP-2 using DBU (0.5 equiv) and 4-F phenylvinylketone (1.5 equiv). ^{1}H NMR (500 MHz, $CDCl_3$) δ 8.02 (d, *J* = 8.6 Hz, 2H), 7.98 (dd, *J* = 10.6, 8.6 Hz, 2H), 7.58 (d, *J* = 8.6 Hz, 2H), 7.11 (t, *J* = 8.6 Hz, 2H), 4.69 (d, *J* = 8.8 Hz, 1H), 3.68 (s, 3H), 3.59 (d, *J* = 8.8 Hz, 1H), 3.35 (ddd, *J* = 17.4, 9.5, 5.5 Hz, 1H), 3.24 (ddd, *J* = 17.4, 9.5, 5.5 Hz, 1H), 2.94 (s, 3H), 2.70 (ddd, *J* = 14.5, 9.5, 5.3 Hz, 1H), 2.27 (ddd, *J* = 14.5, 9.5, 5.3 Hz, 1H) ppm; ^{13}C NMR (126 MHz, $CDCl_3$) δ 197.73, 174.96, 172.31, 169.99, 167.17, 165.93 (d, *J* = 254.9 Hz), 133.24 (d, *J* = 3.0 Hz), 131.83, 131.43, 130.91 (d, *J* = 9.3 Hz), 130.52, 127.02, 115.81 (d, *J* = 21.9 Hz), 84.52, 57.00, 53.71, 53.03, 34.64, 34.04, 25.45 ppm; FT-IR: $\widetilde{\nu}$ = 2971, 2159, 1742, 1699, 1596, 1434, 1286, 1236, 1178, 840, 767 cm^{-1}; HRMS: calc. for $[M+H]^+$ $C_{24}H_{21}N_2O_5{}^{79}BrF$: 515.06124 found: 515.06217; calc. for $[M+H]^+$ $C_{24}H_{21}N_2O_5{}^{81}BrF$: 517. 05919, found: $C_{24}H_{21}N_2O_5{}^{81}BrF$: 517.06020; $[\alpha]_D^{RT}$ = +135 ($CHCl_3$, *c* = 0.5); HPLC conditions: CHIRAPAK IC column, (CH_2Cl_2/EtOH = 100/2) / *iso*-hexane = 70/30, flow rate = 0.5 mL min^{-1}, major enantiomer: t_R = 11.2 min; minor enantiomer: t_R = 14.1 min.

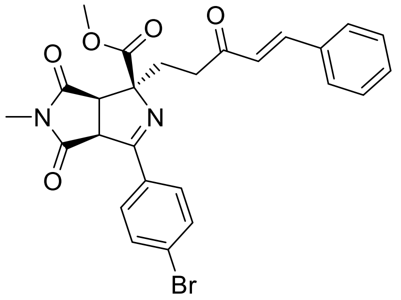

Methyl (1S,3aS,6aR)-3-(4-bromophenyl)-5-methyl-4,6-dioxo-1-((E)-3-oxo-5-phenylpent-4-en-1-yl)-1,3a,4,5,6,6a-hexahydropyrrolo[3,4-c]pyrrole-1-carboxylate

7e was obtained as amorphous colourless solid in 72% yield according to the GP-2 using DBU (0.5 equiv) and vinylstyrylketone (1.5 equiv). ^{1}H NMR (500 MHz, $CDCl_3$) δ 8.05 (d, *J* = 8.6 Hz, 2H), 7.58-7.51 (m, 5H), 7.39-7.38 (m, 3H), 6.72 (d, *J* = 16.2 Hz, 1H), 4.68 (d, *J* = 8.7 Hz, 1H), 3.69 (s, 3H), 3.59 (d, *J* = 8.7 Hz, 1H), 3.08-3.01 (m, 1H), 2.96-2.90 (m, 1H), 2.94 (s, 3H), 2.68-2.62 (m, 1H), 2.28-2.22 (m, 1H) ppm; ^{13}C NMR (126 MHz, $CDCl_3$) δ 199.21, 174.99, 172.31, 170.02, 167.23, 143.11, 134.50, 131.84, 131.51, 130.71, 130.44, 129.09, 128.45, 127.08, 126.03, 84.47, 57.02, 53.44, 53.03, 36.14, 34.34, 25.46 ppm; FT-IR: $\widetilde{\nu}$ = 2954, 2360, 1743, 1699, 1607, 1489, 1432, 1344, 1285, 1246, 1177, 1127, 891, 748 cm^{-1}; HRMS: calc. for $[M+H]^+$ $C_{26}H_{24}N_2O_5{}^{79}Br$: 523.08631 found: 523.08631; calc. for $[M+H]^+$ $C_{26}H_{24}N_2O_5{}^{81}Br$: 525. 08426, found: 525.08553; $[\alpha]_D^{RT}$ = +106 ($CHCl_3$, *c* = 0.5); HPLC conditions: CHIRAPAK

IC column, (CH_2Cl_2/EtOH = 100/2) / *iso*-hexane = 60/40, flow rate = 0.5 mL min^{-1}, major enantiomer: t_R = 26.6 min; minor enantiomer: t_R = 19.1 min.

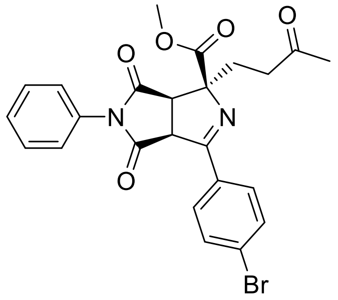

Methyl (1S,3aS,6aR)-3-(4-bromophenyl)-4,6-dioxo-1-(3-oxobutyl)-5-phenyl-1,3a,4,5,6,6a-hexahydropyrrolo[3,4-c]pyrrole-1-carboxylate

7f was obtained as amorphous colourless solid in 51% yield according to the GP-2 using DBU (0.5 equiv) and buten-3-one (1.5 equiv). ^{1}H NMR (500 MHz, $CDCl_3$) δ 8.07 (d, *J* = 8.7 Hz, 2H), 7.60 (d, *J* = 8.6 Hz, 2H), 7.45-7.42 (m, 2H), 7.39-7.36 (m, 1H), 7.23-7.21 (m, 2H), 4.81 (d, *J* = 9.1 Hz, 1H), 3.68 (d, *J* = 9.1 Hz, 1H), 3.68 (s, 3H), 2.89-2.73 (m, 2H), 2.58-2.53 (m, 1H), 2.28-2.22 (m, 1H), 2.18 (s, 3H) ppm; ^{13}C NMR (126 MHz, $CDCl_3$) δ 207.78, 174.09, 171.37, 170.06, 167.15, 131.88, 131.61, 131.54, 130.48, 129.32, 129.07, 127.12, 126.49, 85.35, 57.09, 53.27, 53.22, 38.97, 33.50, 30.20 ppm; FT-IR: $\widetilde{\nu}$ = 2971, 2159, 2030, 1976, 1709, 1590, 1490, 1431, 1347, 1178, 1008, 820, 768 cm^{-1}; HRMS: calc. for $[M+H]^+$ $C_{24}H_{22}O_5N_2{}^{79}Br$: 497.07066, found: 497.07050; calc. for $[M+H]^+$ $C_{24}H_{22}O_5N_2{}^{81}Br$: 499.06861, found: 499.06842; $[\alpha]_D^{RT}$= +180 ($CHCl_3$, *c* = 0.5); HPLC conditions: CHIRAPAK IC column, (CH_2Cl_2/EtOH = 100/2) / *iso*-hexane = 50/50, flow rate = 0.5 mL min^{-1}, major enantiomer: t_R = 26.8 min; minor enantiomer: t_R = 29.9 min.

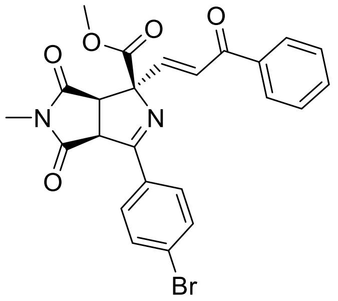

Methyl (1S,3aS,6aR)-3-(4-bromophenyl)-5-methyl-4,6-dioxo-1-((E)-3-oxo-3-phenylprop-1-en-1-yl)-1,3a,4,5,6,6a-hexahydropyrrolo[3,4-c]pyrrole-1-carboxylate

7g was obtained as amorphous colourless solid in 71% yield according to the GP-2 using DBU (0.5 equiv) and buten-3-one (1.5 equiv). ^{1}H NMR (600 MHz, $CDCl_3$) δ 8.18 – 8.12 (m, 2H), 7.91 (dd, *J* = 8.2, 1.1 Hz, 2H), 7.68 – 7.64 (m, 2H), 7.57 (dd, *J* = 10.5, 4.3 Hz, 1H), 7.52 – 7.44 (m, 3H), 6.95 (d, *J* = 15.5 Hz, 1H), 4.67 (d, *J* = 8.7 Hz, 1H), 3.79 – 3.72 (m, 4H), 2.98 (s, 3H). ^{13}C NMR (151 MHz, $CDCl_3$) δ 190.63 (s), 174.31 (s), 171.77 (s), 169.20 (s), 169.01 (s), 145.79 (s), 137.27 (s), 133.42 (s), 132.05 (s), 131.65 (s), 130.20 (s), 128.95 (s), 128.83 (s), 127.56 (s), 125.77 (s), 84.74 (s), 56.67 (s), 53.72 (s), 53.65 (s), 25.68 (s) ppm; FT-IR: $\tilde{\nu}$ = 2972, 2159, 2030, 1976, 1700, 1595, 1489, 1431, 1344, 1246, 1177, 822, 767 cm^{-1}; HRMS: calc. for $[M+H]^+$ $C_{24}H_{20}O_5N_2Br$ = 495.05501, found: 495.05444; calc. for $[M+H]^+$ $C_{24}H_{20}O_5N_2{}^{81}Br$ = 497.05296, found: 497.05240; $[\alpha]_D^{RT}$ = +196 ($CHCl_3$, *c* = 0.5); HPLC conditions: CHIRAPAK IC column, (CH_2Cl_2/EtOH = 100/2) / *iso*-hexane = 40/60, flow rate = 0.5 mL min^{-1}, major enantiomer: t_R = 47.5 min; minor enantiomer: t_R = 44.1 min.

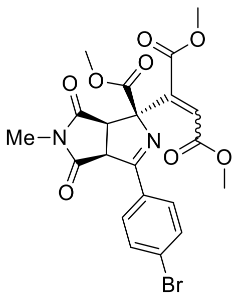

Dimethyl 2-((1R,3aS,6aR)-3-(4-bromophenyl)-1-(methoxycarbonyl)-5-methyl-4,6-dioxo-1,3a,4,5,6,6a-hexahydropyrrolo[3,4-c]pyrrol-1-yl)but-2-enedioate

7h was obtained as amorphous colourless solid in 64% yield (E/Z 3:1, and the major isomer was obtained) according to the GP-2 using Et_3N (1.0 equiv) and dimethyl acetylenedicarboxylate (1.5 equiv). The NMR data correspond to the major isomer. ^{1}H NMR (500 MHz, $CDCl_3$) δ 8.02 (d, *J* = 8.7 Hz, 2H), 7.61 (d, *J* = 8.7 Hz, 2H), 6.97 (s, 1H), 4.78 (d, *J* = 9.2 Hz, 1H), 3.94 (d, *J* = 9.2 Hz, 1H), 3.91 (s, 3H), 3.69 (s, 3H), 3.33 (s, 3H), 3.02 (s, 3H) ppm; ^{13}C NMR (126 MHz, $CDCl_3$) δ 173.44, 171.57, 171.46, 168.35, 166.05, 165.64, 138.48, 131.89, 131.77, 130.68, 130.29, 127.67, 86.19, 58.02, 53.74, 53.63, 52.89, 52.36, 25.65 ppm; FT-IR: $\tilde{\nu}$ = 2960, 2159, 2030, 1747, 1698, 1614, 1590, 1430, 1240, 1184, 1240, 1012, 812, 783, 753 cm^{-1}; HRMS: calc. for $[M+H]^+$ $C_{21}H_{20}N_2O_8{}^{79}Br$: 507.03975 found: 507.04124; $C_{21}H_{20}N_2O_8{}^{81}Br$: 509. 03771 found: $C_{21}H_{20}N_2O_8{}^{81}Br$: 509.03931. $[\alpha]_D^{RT}$ = +342 ($CHCl_3$, *c* = 0.5); HPLC conditions: CHIRAPAK IC column, (CH_2Cl_2/EtOH = 100/2) / *iso*-hexane = 40/60, flow rate = 0.5 mL min^{-1}, major enantiomer: t_R = 39.5 min; minor enantiomer: t_R = 22.6 min.

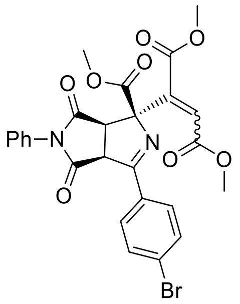

Dimethyl 2-((1R,3aS,6aR)-3-(4-bromophenyl)-1-(methoxycarbonyl)-4,6-dioxo-5-phenyl-1,3a,4,5,6,6a-hexahydropyrrolo[3,4-c]pyrrol-1-yl)but-2-enedioate

7i was obtained as amorphous colourless solid in 69% yield (E/Z 3:1, and the major isomer was obtained) according to the GP-2 using DBU (0.5 equiv) and buten-3-one (1.5 equiv). ^{1}H NMR (600 MHz, $CDCl_3$) δ 8.07 – 8.02 (m, 2H), 7.63 – 7.58 (m, 2H), 7.45 (t, J = 7.7 Hz, 2H), 7.40 – 7.34 (m, 3H), 7.02 (s, 1H), 4.97 (d, J = 9.5 Hz, 1H), 4.05 (d, J = 9.5 Hz, 1H), 3.89 (s, 3H), 3.73 (s, 3H), 3.34 (s, 3H) ppm; ^{13}C NMR (151 MHz, $CDCl_3$) δ 172.45, 171.53, 170.63, 168.51, 166.09, 165.55, 138.55, 132.07, 131.86, 131.85, 130.87, 130.18, 129.25, 128.90, 127.72, 126.77, 86.80, 58.02, 53.82, 53.68, 52.91, 52.41 ppm; FT-IR: $\tilde{\nu}$ = 2956, 2159, 2030, 1715, 1590, 1492, 1433, 1384, 1251, 1181, 1010, 821, 771, 743 cm^{-1}; HRMS: calc. for $[M+H]^+$ $C_{26}H_{22}O_8N_2Br$ = 569.05540, found: 569.05524; calc. for $[M+H]^+$ $C_{26}H_{22}O_8N_2{}^{81}Br$ = 571.05336, found: 571.05322; $[\alpha]_D^{RT}$ = +221 ($CHCl_3$, c = 0.5); HPLC conditions: CHIRAPAK IC column, (CH_2Cl_2/EtOH = 100/2) / *iso*-hexane = 30/70, flow rate = 0.5 mL min^{-1}, major enantiomer: t_R = 78.0 min; minor enantiomer: t_R = 74.3 min.

Pulldown probe synthesis and synthesis of compounds 7j-7n

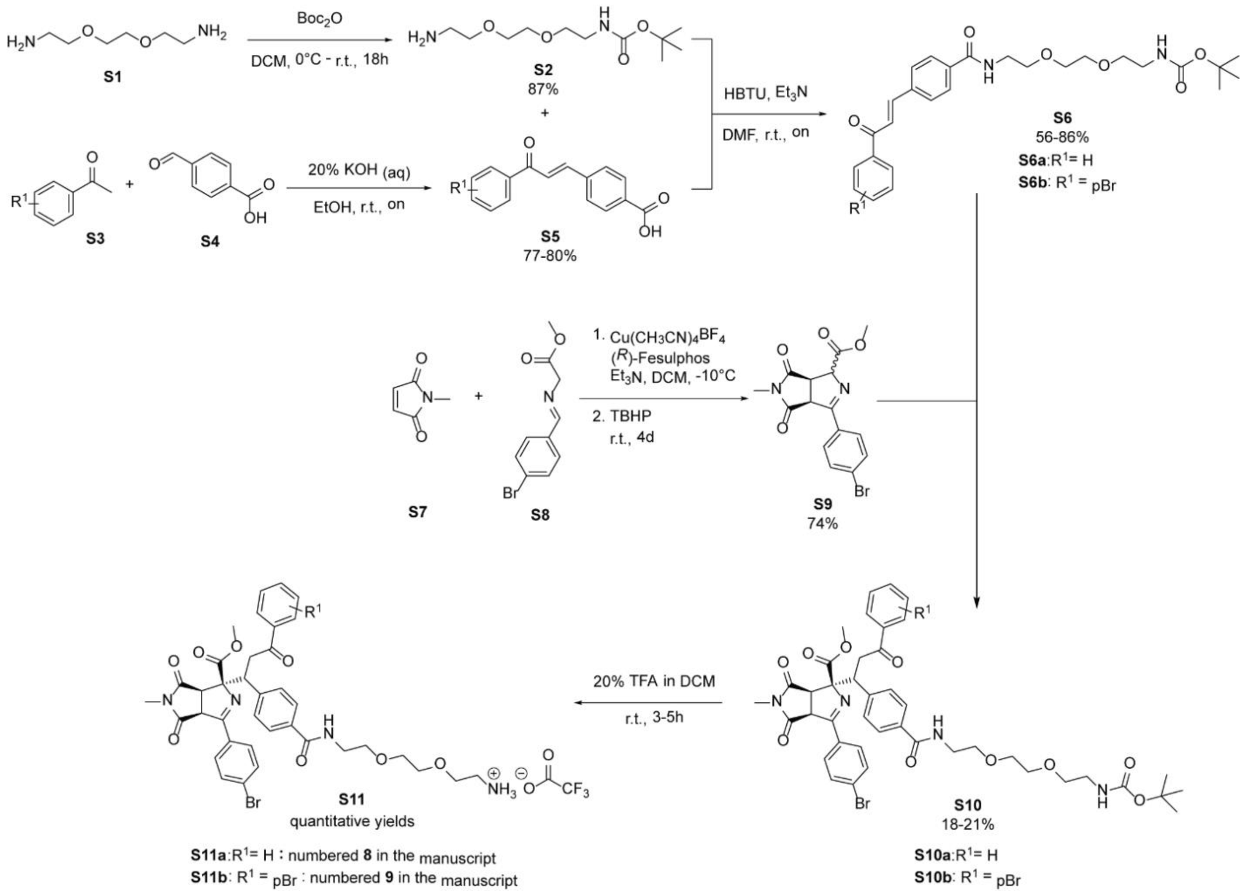

Scheme S3: Pulldown probe synthesis strategy.

Preparation of *tert*-butyl (2-(2-(2-aminoethoxy)ethoxy)ethyl)carbamate

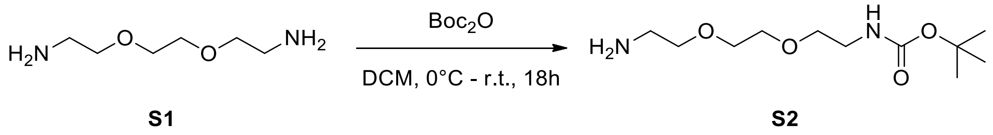

To the stirred solution of 2,2′-(ethylenedioxy)-bis(ethylamine) (3.395 g, 24.2 mmol, 5 equiv.) in dichloromethane (10 mL) was added Di-*tert*-butyl dicarbonate (1 g, 4.6 mmol, 1 equiv.) solved in dichloromethane (20 mL) over 8 hours at 0°C. The reactions mixture was further stirred over night at ambient temperature. The mixture was washed 2x with water, 1x with brine and dried over $NaSO_4$. The solvent was removed under vacuum to give the product, which was used without further purification. 87% yield; colorless oil; ^{1}H NMR (600 MHz, $CDCl_3$): δ = 5.16 (br s, 1H), 3.62 (s, 4H), 3.57 – 3.51 (m, 4H), 3.35 – 3.28 (m, 2H), 2.90 (m, 2H),1.44 ppm (s, 9H); ^{13}C NMR (151 MHz, $CDCl_3$): δ = 156.18, 79.33, 73.26, 70.35, 70.33, 41.75, 40.46, 28.55 ppm; HRMS: calcd. for $[M+H]^+$ $C_{11}H_{25}O_4N_2$ = 249.18088, found: 249.18125.

General procedure: Preparation of chalcones 1

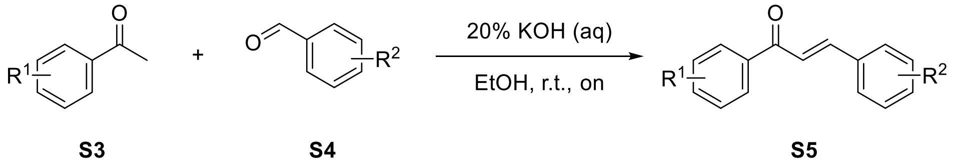

4-Carboxybenzaldehyde (1 mmol, 1 equiv.) and acetophenone (1 mmol, 1 equiv.) were dissolved in 5 mL ethanol. 600 µL of KOH (20% w/v aqueous solution) was added to the solution and the mixture was stirred over night at ambient temperature. The mixture was cooled to 0°C and acidified with 10% aqueous HCl solution. The formed precipitate was filtered and dissolved in ethyl acetate. The organic phase was washed with a saturated NaCl solution and dried over $MgSO_4$. The solvent was removed under vacuum to give the chalcone as an amorphous solid, which was used without further purification.

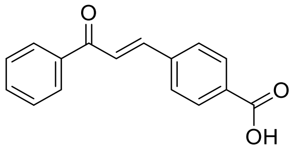

4-Carboxy chalcone (S5a)

80% yield; slightly yellow amorphous solid; ^{1}H NMR (500 MHz, Acetone-6d): δ = 11.45 (s, 2H), 8.21 – 8.16 (m, 6H), 8.11 (d, *J* = 8.3 Hz, 6H), 8.03 – 7.97 (m, 8H), 7.84 (d, *J* = 15.7 Hz, 3H), 7.68 (ddd, *J* = 8.5, 2.4, 1.2 Hz, 3H), 7.58 ppm (t, *J* = 7.6 Hz, 6H); ^{13}C NMR (126 MHz, Acetone-6d): δ = 189.78, 167.04, 143.32, 140.27, 138.81, 133.92, 132.66, 130.96, 129.61, 129.51, 129.40, 125.04 ppm; HRMS: calcd. for $[M+H]^+$ $C_{16}H_{13}O_3$ = 253.08592, found: 253.08594.

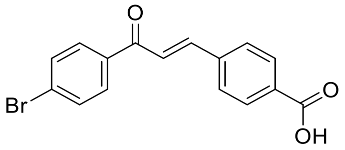

4´-Bromo-4-carboxy chalcone (S5b)

77% yield; colorless amorphous solid; ^{1}H NMR (500 MHz, THF-d8): δ = 11.56 (s, 1H), 8.08 – 8.03 (m, 4H), 7.88 – 7.84 (m, 4H), 7.74 – 7.69 ppm (m, 2H); ^{13}C NMR (126 MHz, THF-d8): δ = 188.42, 167.21, 144.06, 140.25, 138.11, 133.42, 132.90, 131.31, 131.13, 129.43, 128.65, 124.34 ppm.

Preparation of chalcones 2:

A 10 % aq. NaOH solution (2.5 ml) was added dropwise to a mixture of ketone (8.2 mmol) and aldehyde (8.2 mmol) in water (50 ml) at 0 °C under vigorous stirring. After the addition, the reaction was warmed to 22 °C and stirred overnight. The reaction was cooled in an ice bath, filtered, washed with cold water, and dried to afford the pure product.

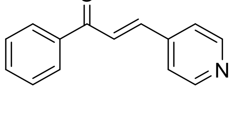

(E)-1-phenyl-3-(pyridin-4-yl)prop-2-en-1-one (S5c)

Following the preparation of chalcones 2 procedure, 1.61 g (94%) of the desired product was obtained as a white solid; ^{1}H NMR (600 MHz, Chloroform-*d*) δ 8.69 (d, *J* = 6.1 Hz, 2H), 8.02 (d, *J* = 7.1 Hz, 2H), 7.73 – 7.64 (m, 2H), 7.62 (t, *J* = 7.4 Hz, 1H), 7.53 (t, *J* = 7.7 Hz, 2H), 7.48 (d, *J* = 6.1 Hz, 2H); ^{13}C NMR (151 MHz, $CDCl_3$) δ 190.06, 150.81, 142.41, 141.74, 137.73,

133.61, 129.06, 128.86, 126.36, 122.30; HRMS: calc. for $[M+H]^+$ $C_{14}H_{11}O_5NO$ = 210.09134, found: 210.09135.

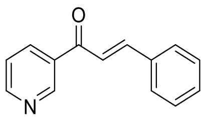

(E)-3-phenyl-1-(pyridin-3-yl)prop-2-en-1-one (S5d)

Following the preparation of chalcones 2 procedure, 1.48 g (86%) of the desired product was obtained as a yellow solid; ^{1}H NMR (500 MHz, Chloroform-*d*) δ 9.24 (d, *J* = 2.2 Hz, 1H), 8.81 (dd, *J* = 4.9, 1.7 Hz, 1H), 8.30 (dt, *J* = 7.9, 2.0 Hz, 1H), 7.85 (d, *J* = 15.7 Hz, 1H), 7.66 (dd, *J* = 6.7, 2.8 Hz, 2H), 7.53 – 7.42 (m, 5H); ^{13}C NMR (126 MHz, $CDCl_3$) δ 189.26, 153.24, 149.81, 146.24, 136.16, 134.54, 133.66, 131.19, 129.22, 128.79, 123.88, 121.45; HRMS: calc. for $[M+H]^+$ $C_{14}H_{11}O_5NO$ = 210.09134, found: 210.09131.

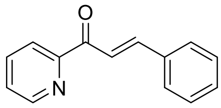

(E)-3-phenyl-1-(pyridin-2-yl)prop-2-en-1-one (S5e)

Following the preparation of chalcones 2 procedure, an impure mixture was obtained. This mixture was purified via MPLC (5-50% EtOAc in CyHex) to afford 1.44 g (84%) of the desired product as a yellow solid; ^{1}H NMR (600 MHz, Chloroform-*d*) δ 8.75 (ddd, *J* = 4.8, 1.7, 0.9 Hz, 1H), 8.31 (d, *J* = 16.0 Hz, 1H), 8.20 (d, *J* = 7.8 Hz, 1H), 7.95 (d, *J* = 16.1 Hz, 1H), 7.88 (td, *J* = 7.7, 1.7 Hz, 1H), 7.79 – 7.71 (m, 2H), 7.49 (ddd, *J* = 7.6, 4.7, 1.3 Hz, 1H), 7.45 – 7.40 (m, 3H); ^{13}C NMR (151 MHz, $CDCl_3$) δ 119.76, 84.49, 79.12, 75.05, 67.27, 65.41, 60.82, 59.11, 59.10, 57.15, 53.19, 51.11; HRMS: calc. for $[M+H]^+$ $C_{14}H_{11}O_5NO$ = 210.09134, found: 210.09129.

General procedure: Preparation of S6

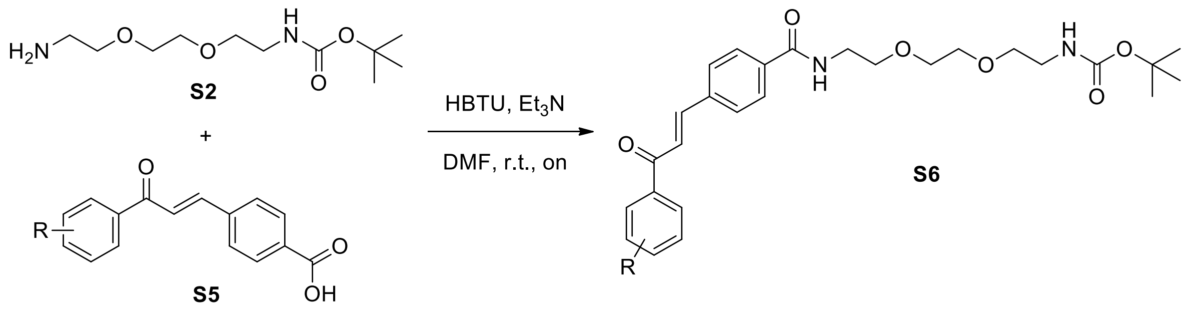

To the stirred solution of chalcone **S5** (0.20 mmol, 1 equiv.), linker **S2** (0.30 mmol, 1.5 equiv.) and triethylamine (0.70 mmol, 3.5 equiv.) in 2.5 mL DMF was added HBTU (0.70 mmol, 3.5 equiv.). The mixture was stirred at ambient temperature for 18 hours. The solvent was removed under reduced pressure. The residue was solved in dichloromethane and washed with a saturated $NaHCO_3$ solution. The aqueous phase was extracted with dichloromethane and the combined organic phases were washed with brine and dried over $MgSO_4$. After removing the solvent under reduced pressure the purification was done by silica column chromatography and the product was obtained using ethylacetate / methanol as eluent (from 0% to 2%).

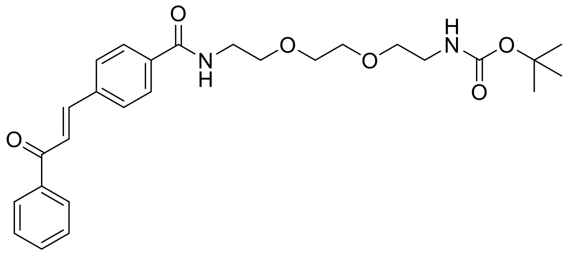

***tert*-butyl (*E*)-(2-(2-(2-(4-(3-oxo-3-phenylprop-1-en-1-yl)benzamido)ethoxy)ethoxy)-ethyl)carbamate (S6a)**

86% yield; yellow oil; ^{1}H NMR (400 MHz, Acetone-d6): δ = 8.18 – 8.14 (m, 2H), 8.01 – 7.91 (m, 5H), 7.81 (d, *J* = 15.7 Hz, 1H), 7.69 – 7.64 (m, 1H), 7.60 – 7.54 (m, 2H), 3.68 – 3.56 (m, 8H), 3.51 (t, *J* = 5.8 Hz, 2H), 3.22 (q, *J* = 5.8 Hz, 1H), 1.39 ppm (s, 9H).; ^{13}C NMR (101 MHz, Acetone-d6): δ = 189.88, 166.78, 143.66, 138.97, 138.57, 137.29, 133.83, 129.60, 129.42, 129.38, 128.64, 124.34, 70.95, 70.72, 70.33, 41.07, 40.53, 28.63 ppm; HRMS: calcd. for $[M+H]^+$ $C_{27}H_{35}O_6N_2$ = 483.24896, found: 483.24947.

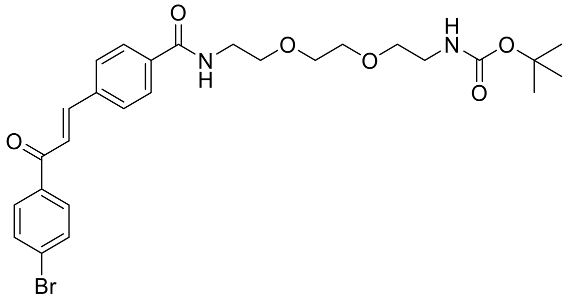

***tert*-butyl (*E*)-(1-(4-(3-(4-bromophenyl)-3-oxoprop-1-en-1-yl)phenyl)-1-oxo-5,8,11-trioxa-2-azatridecan-13-yl)carbamate (S6b)**

56% yield; yellow oil; ^{1}H NMR (600 MHz, $CDCl_3$): δ = 7.94 – 7.85 (m, 4H), 7.81 (d, *J* = 15.7 Hz, 1H), 7.69 (d, *J* = 8.1 Hz, 2H), 7.66 (d, *J* = 8.4 Hz, 2H), 7.54 (d, *J* = 15.7 Hz, 1H), 3.69 (s, 4H), 3.65 (s, 4H), 3.57 (t, *J* = 5.2 Hz, 2H), 3.31 (t, *J* = 5.0 Hz, 2H), 1.44 ppm (s, 9H); ^{13}C NMR (151 MHz, $CDCl_3$): δ = 189.23, 166.82, 144.09, 137.63, 136.78, 136.27, 132.16, 130.22, 128.65, 128.33, 127.95, 123.06, 70.53, 70.35, 40.09, 28.54 ppm; HRMS: calcd. for $[M+H]^+$ $C_{27}H_{34}{}^{79}BrO_6N_2$ = 561.15948, found: 561.16062; calcd. for $[M+H]^+$ $C_{27}H_{34}{}^{81}BrO_6N_2$ = 563.15743, found: 563.15847.

General procedure: 1,3-dipolar cycloaddition and oxidation

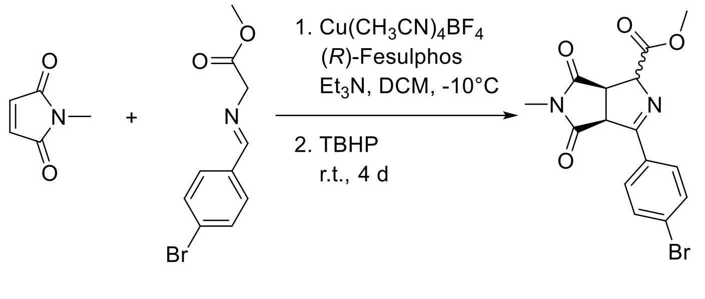

S7 **S8** **S9**

(R_p)-2-(*tert*-Butylthio)-1-(diphenylphosphino)ferrocene (1 mol%, 40 µmol) and tetrakis-(acetonitrile)copper(I) tetrafluoroborate (1 mol%, 40 µmol) were dissolved in dry dichloromethane and stirred at ambient temperature for 15 min. The resulting solution was cooled to 0°C and *N*-Methylmaleimide (1.0 eq., 4.05 mmol), α-iminoester **S8** (1.02 equiv., 4.13 mmol) and Et_3N (20 mol%, 0.81 mmol) were added. The solution was allowed to stirr at 0°C for 3 hours. After the *N*-Methylmaleimide was completely used up tetrakis-(acetonitrile)copper(I) tetrafluoroborate (4 mol%, 160 µmol) and *tert*-butyl hydroperoxide were added. The deep blue solution was allowed to stir at ambient temperature for 96 h. The

crude mixture was directly charged onto silica gel and the product was purified using petroleum ether / ethyl acetate as eluent.

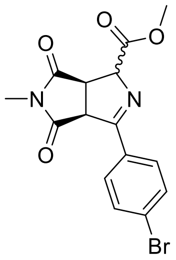

Methyl (3a*S*,6a*R*)-3-(4-bromophenyl)-5-methyl-4,6-dioxo-1,3a,4,5,6,6a-hexahydro-pyrrolo[3,4-*c*]pyrrole-1-carboxylate (S9)

S9 was obtained as amorphous pale yellow solid in 74% yield (1:0.8 mixture of diastereomers). The NMR data correspond to the major isomer. ^{1}H NMR (500 MHz, $CDCl_3$): δ = 8.04 (d, *J* = 8.5 Hz, 2H), 7.60 (d, *J* = 8.4 Hz, 2H), 5.23 (m, 1H), 4.70 (dd, *J* = 8.4, 2.5 Hz, 1H), 4.08 (dd, *J* = 8.4, 3.0 Hz, 1H), 3.84 (s, 1H), 2.96 (s, 2H); HRMS: calcd. for $[M+H]^+$ $C_{15}H_{14}{}^{79}BrO_4N_2$ = 365.01315, found: 365.01412; calcd. for $[M+H]^+$ $C_{15}H_{14}{}^{81}BrO_4N_2$ = 367.01110, found: 367.01170.

1,3-Dipolar cycloaddition and oxidation procedure 2:

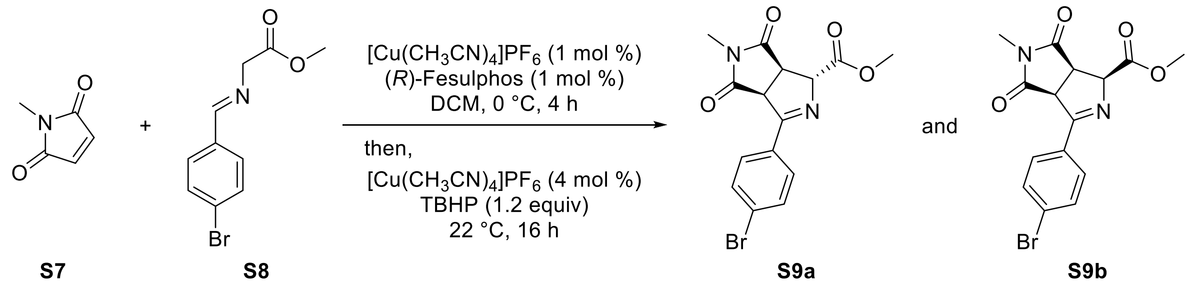

Compounds **S9a** and **S9b** afforded from procedure 2 were used as substrates to make **7j-n**. To a dry 50 RBF equipped with a stirbar was added $[Cu(CH_3CN)_4]PF_6$ (7.5 mg, 0.02 mmol, 0.01 equiv.), (*R*)-Fesulphos (9.2 mg, 0.02 mmol, 0.01 equiv.), and dry DCM (10 ml). The flask was capped at stirred at 22 °C for 30 min. After this time, the reaction was cooled to 0 °C, and the maleimide (222 mg, 2 mmol, 1 equiv.), the azomethine ylide (538 mg, 2.10 mmol, 1.05 equiv.), and triethylamine (56 µL, 0.4 mmol, 0.2 equiv.) were added successively. After all of the maleimide had been consumed (4 h), more $[Cu(CH_3CN)_4]PF_6$ (40 mg) was added to the

reaction followed by tert-butylhydrogenperoxide (70% in water, 329 μL, 2.40 mmol, 2.2 equiv.). The reaction was warmed to 22 °C and stirred for 16 h. After this time, the reaction was concentrated and directly purified by silica chromatography (15-50% EtOAc in CyHex) to afford two diastereomers.

The trans isomer **S9a** was isolated as a white solid (178 mg, 24%); ^{1}H NMR (700 MHz, Chloroform-*d*) δ 8.03 (d, *J* = 8.7 Hz, 2H), 7.58 (d, *J* = 8.7 Hz, 2H), 5.21 (t, *J* = 2.8 Hz, 1H), 4.69 (dd, *J* = 8.4, 2.6 Hz, 1H), 4.06 (dd, *J* = 8.4, 3.0 Hz, 1H), 3.82 (s, 3H), 2.94 (s, 3H); ^{13}C NMR (176 MHz, $CDCl_3$) δ 176.21, 172.53, 170.33, 168.44, 131.84, 131.30, 130.18, 127.12, 76.73, 56.54, 53.34, 47.55, 25.48; HRMS: calc. for $[M+H]^+$ $C_{15}H_{13}O_4N_2{}^{79}Br$ = 365.01315, found: 365.01339; calc. for $[M+H]^+$ $C_{15}H_{13}O_4N_2{}^{81}Br$ = 367.01110, found: 367.01089; $[\alpha]_D^{RT}$ = +111 ($CHCl_3$, *c* = 0.2); ee = 96%, HPLC conditions: CHIRAPAK IC column, *iso*-hexane / *iso*-propanol = 60/40, flow rate = 0.5 mL min^{-1}, major enantiomer: t_R = 63.1 min; minor enantiomer: t_R = 35.7 min.

The cis isomer **S9b** was isolated as a white solid (93 mg, 13%); ^{1}H NMR (700 MHz, Chloroform-*d*) δ 8.05 (d, *J* = 8.7 Hz, 2H), 7.59 (d, *J* = 8.6 Hz, 2H), 5.27 (dd, *J* = 10.0, 1.3 Hz, 1H), 4.61 (dd, *J* = 8.8, 1.4 Hz, 1H), 3.93 (t, *J* = 9.4 Hz, 1H), 3.77 (s, 3H), 2.94 (s, 3H); ^{13}C NMR (176 MHz, $CDCl_3$) δ 175.06, 172.25, 169.62, 168.73, 131.86, 131.37, 130.38, 127.05, 75.59, 57.04, 52.88, 46.97, 25.54; HRMS: calc. for $[M+H]^+$ $C_{15}H_{13}O_4N_2{}^{79}Br$ = 365.01315, found: 365.01335; calc. for $[M+H]^+$ $C_{15}H_{13}O_4N_2{}^{81}Br$ = 367.01110, found: 367.01108; $[\alpha]_D^{RT}$ = +263 ($CHCl_3$, *c* = 0.15); ee = 96%, HPLC conditions: CHIRAPAK IC column, *iso*-hexane / *iso*-propanol = 60/40, flow rate = 0.5 mL min^{-1}, major enantiomer: t_R = 17.6 min; minor enantiomer: t_R = 29.8 min.

General procedure: Michael Addition

$[Cu(CH_3)CN_4)]PF_6$ (3 mg, 0.008 mmol, 0.05 equiv.) was added to a round bottomed flask and dissolved in dry DCM (2 ml). The imine **9a** (either **9a**, **9b**, or a mixture of **9a** and **9b** could be employed with similar results) (58 mg, 0.16 mmol, 1 equiv., 96% ee) and a chalcone (0.24 mmol, 1.5 equiv) were added, and the reaction was cooled to 0 °C. DBU (12 μl, 0.08 mmol, 0.5 equiv.) was added dropwise and the reaction was stirred for 30 min – 3 hours.

Once the starting imine was completely consumed, the reaction was directly concentrated and purified by MPLC. In some cases, the diastereomers could be separated by HPLC. The desired products were assumed to have the same ee as the starting material (96% ee).

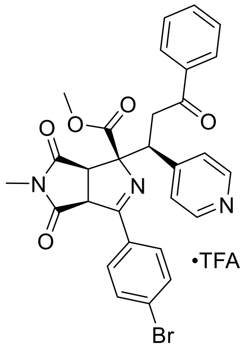

7j

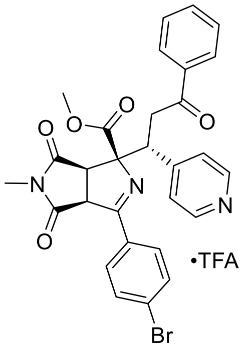

7k

The general procedure was followed with a reaction time of 30 min. After MPLC (30-100% EtOAc in CyHex), 43 mg (47%) of a 2:1 diastereomeric mixture was obtained. Further purification by preparative HPLC (MeOH/H_2O + 0.1 % TFA) afforded 13 mg (12%) of the first isomer and 6 mg (5%) of the second isomer as white powders.

Major isomer **7j**;^{1}H NMR (700 MHz, Chloroform-*d*) δ 8.58 (s, 2H), 8.01 (d, *J* = 8.2 Hz, 2H), 7.93 (d, *J* = 7.7 Hz, 2H), 7.65 (d, *J* = 8.4 Hz, 4H), 7.59 (t, *J* = 7.5 Hz, 1H), 7.47 (t, *J* = 7.7 Hz, 2H), 4.53 – 4.28 (m, 1H), 4.24 – 3.99 (m, 2H), 3.95 (d, *J* = 16.3 Hz, 1H), 3.69 (s, 3H), 3.48 (d, *J* = 8.7 Hz, 1H), 2.92 (s, 3H); ^{13}C NMR (176 MHz, $CDCl_3$) δ 196.64, 174.37, 171.24, 170.04, 168.82, 157.23, 143.08, 135.96, 134.07, 132.33, 131.48, 129.64, 128.99, 128.25, 128.06, 127.89, 87.22, 56.90, 53.53, 52.63, 48.97, 40.86, 25.57; HRMS: calc. for $[M+H]^+$ $C_{29}H_{24}O_5N_3{}^{79}Br$ = 574.09721, found: 574.09741; calc. for $[M+H]^+$ $C_{29}H_{24}O_5N_3{}^{81}Br$ = 576.09516, found: 576.09430; $[\alpha]_D^{RT}$ = +137 ($CHCl_3$, *c* = 0.2).

Minor isomer **7k**; ^{1}H NMR (700 MHz, Chloroform-*d*) δ 8.65 (d, *J* = 5.2 Hz, 2H), 8.08 (d, *J* = 8.2 Hz, 2H), 8.01 (d, *J* = 4.9 Hz, 2H), 7.82 (d, *J* = 6.9 Hz, 2H), 7.67 (d, *J* = 8.3 Hz, 2H), 7.58 (t, *J* = 7.4 Hz, 1H), 7.44 (t, *J* = 7.7 Hz, 2H), 4.63 (dd, *J* = 9.8, 3.0 Hz, 1H), 4.60 (d, *J* = 7.4 Hz, 1H), 3.70 (d, *J* = 7.9 Hz, 1H), 3.57 (s, 4H), 3.43 (d, *J* = 17.9 Hz, 1H), 2.97 (s, 3H); ^{13}C NMR (176 MHz, $CDCl_3$) δ 195.91, 174.32, 171.36, 169.17, 168.58, 159.21, 142.28, 135.84, 134.32, 132.31, 131.61, 129.65, 129.10, 128.23, 128.14, 127.86, 87.56, 57.28, 53.47, 51.82, 47.78, 39.67, 25.70; HRMS: calc. for $[M+H]^+$ $C_{29}H_{24}O_5N_3{}^{79}Br$ = 574.09721, found: 574.09740; calc. for $[M+H]^+$ $C_{29}H_{24}O_5N_3{}^{81}Br$ = 576.09516, found: 576.09413; $[\alpha]_D^{RT}$ = +139 ($CHCl_3$, *c* = 0.15).

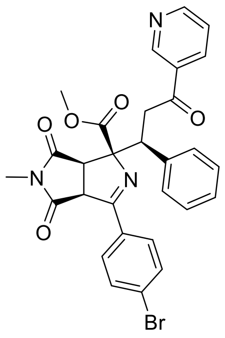

7l

The general procedure was followed on a 0.82 mmol scale with a reaction time of 3 h. A 6:1 dr was observed by crude NMR. After MPLC (25-100% EtOAc in CyHex), 280 mg (59%) of the major isomer **7l** was obtained as a white powder. The minor isomer was not obtained in pure form.

^{1}H NMR (700 MHz, Chloroform-*d*) δ 9.12 (d, *J* = 1.5 Hz, 1H), 8.74 (dd, *J* = 4.8, 1.7 Hz, 1H), 8.18 (dt, *J* = 8.0, 2.0 Hz, 1H), 7.98 (d, *J* = 8.6 Hz, 2H), 7.63 (d, *J* = 8.6 Hz, 2H), 7.37 (dd, *J* = 8.0, 4.8 Hz, 1H), 7.17 (t, *J* = 7.3 Hz, 1H), 7.11 (t, *J* = 7.7 Hz, 2H), 7.06 (d, *J* = 7.2 Hz, 2H), 4.48 (dd, *J* = 10.9, 2.7 Hz, 1H), 3.83 – 3.76 (m, 4H), 3.65 (dd, *J* = 17.4, 2.7 Hz, 1H), 3.52 (d, *J* = 8.2 Hz, 1H), 3.16 (d, *J* = 8.3 Hz, 1H), 2.84 (s, 3H); ^{13}C NMR (176 MHz, $CDCl_3$) δ 196.49, 175.06, 171.87, 170.39, 169.65, 153.43, 149.61, 137.56, 135.70, 132.13, 131.97, 131.25, 130.30, 130.23, 128.60, 127.95, 127.12, 123.73, 87.48, 56.61, 53.26, 52.28, 48.83, 41.57, 25.39; HRMS: calc. for $[M+H]^+$ $C_{29}H_{24}O_5N_3{}^{79}Br$ = 574.09721, found: 574.09725; calc. for $[M+H]^+$ $C_{29}H_{24}O_5N_3{}^{81}Br$ = 576.09516, found: 576.09473; $[\alpha]_D^{RT}$ = +171 ($CHCl_3$, *c* = 0.2).

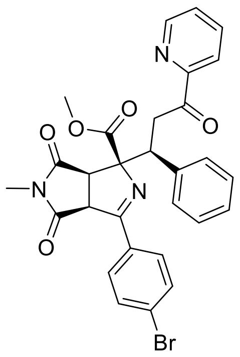

7m

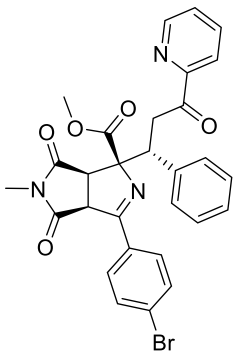

7n

The general procedure was followed with a reaction time of 3 h. A 6:1 dr was observed by crude NMR. After MPLC (10-50% EtOAc in CyHex), 45 mg (49%) of the major isomer and 7 mg (8%) of the minor isomer were obtained as white powders.

Major isomer **7m**; ^{1}H NMR (600 MHz, Chloroform-*d*) δ 8.66 (ddd, *J* = 4.8, 1.8, 0.9 Hz, 1H), 8.00 (d, *J* = 8.6 Hz, 2H), 7.86 (d, *J* = 7.9 Hz, 1H), 7.73 (td, *J* = 7.7, 1.7 Hz, 1H), 7.61 (d, *J* = 8.6 Hz, 2H), 7.41 (ddd, *J* = 7.5, 4.8, 1.3 Hz, 1H), 7.15 – 7.07 (m, 5H), 4.53 (dd, *J* = 11.0, 2.5 Hz, 1H), 4.26 (dd, *J* = 18.8, 11.0 Hz, 1H), 3.76 – 3.72 (m, 4H), 3.56 (d, *J* = 8.2 Hz, 1H), 3.11 (d, *J* = 8.2 Hz, 1H), 2.84 (s, 3H); ^{13}C NMR (151 MHz, $CDCl_3$) δ 199.17, 175.23, 172.10, 170.45, 168.97, 153.31, 148.98, 138.48, 136.85, 131.82, 131.33, 130.51, 130.49, 128.35, 127.56, 127.21, 126.81, 121.85, 87.73, 56.54, 53.07, 52.38, 48.67, 40.51, 25.35; HRMS: calc. for $[M+H]^+$ $C_{29}H_{24}O_5N_3{}^{79}Br$ = 574.09721, found: 574.09740; calc. for $[M+H]^+$ $C_{29}H_{24}O_5N_3{}^{81}Br$ = 576.09516, found: 576.09450; $[\alpha]_D^{RT}$ = +149 ($CHCl_3$, *c* = 0.2).

Minor isomer **7n**; ^{1}H NMR (600 MHz, Chloroform-*d*) δ 8.68 (ddd, *J* = 4.8, 1.7, 0.9 Hz, 1H), 8.02 (d, *J* = 8.6 Hz, 2H), 7.91 (d, *J* = 7.8 Hz, 1H), 7.78 (td, *J* = 7.7, 1.7 Hz, 1H), 7.58 (d, *J* = 8.6 Hz, 2H), 7.45 (ddd, *J* = 7.6, 4.8, 1.3 Hz, 1H), 7.36 (d, *J* = 7.1 Hz, 2H), 7.14 (t, *J* = 7.4 Hz, 2H), 7.08 (t, *J* = 7.3 Hz, 1H), 4.58 (dd, *J* = 10.4, 3.3 Hz, 1H), 4.26 (d, *J* = 8.9 Hz, 1H), 4.05 (dd, *J* = 17.3, 10.3 Hz, 1H), 3.97 (d, *J* = 8.9 Hz, 1H), 3.71 (dd, *J* = 17.3, 3.3 Hz, 1H), 3.60 (s, 3H), 2.96 (s, 3H); ^{13}C NMR (151 MHz, $CDCl_3$) δ 199.28, 175.46, 172.48, 169.70, 166.90, 153.30, 149.16, 138.98, 137.01, 131.78, 131.43, 130.54, 129.97, 128.06, 127.36, 127.28, 126.83, 122.11, 88.61, 57.63, 52.86, 50.59, 47.25, 38.75, 25.53; HRMS: calc. for $[M+H]^+$ $C_{29}H_{24}O_5N_3{}^{79}Br$ = 574.09721, found: 574.09728; calc. for $[M+H]^+$ $C_{29}H_{24}O_5N_3{}^{81}Br$ = 576.09516, found: 576.09485; $[\alpha]_D^{RT}$ = +135 ($CHCl_3$, *c* = 0.12).

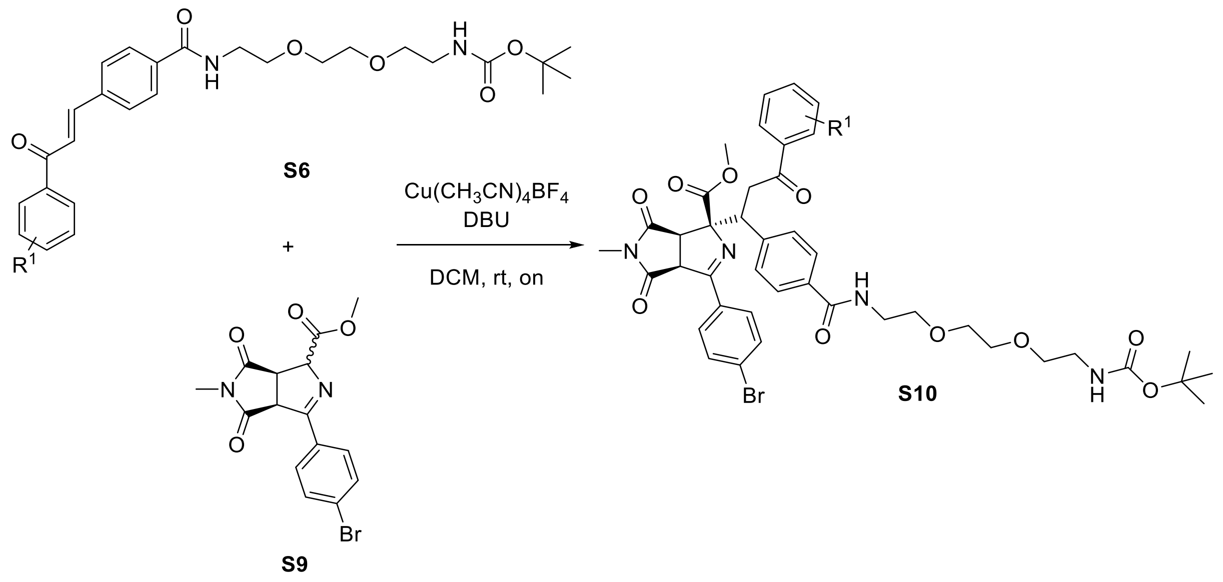

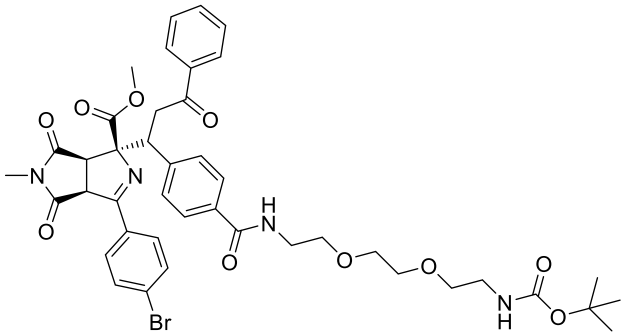

Methyl (1*S*,3a*S*,6a*R*)-3-(4-bromophenyl)-1-((*R*)-1-(4-((2,2-dimethyl-4-oxo-3,8,11-trioxa-5-azatridecan-13-yl)carbamoyl)phenyl)-3-oxo-3-phenylpropyl)-5-methyl-4,6-dioxo-1,3a,4,5,6,6a-hexahydropyrrolo[3,4-c]pyrrole-1-carboxylate (S10a)

18% yield; amorphous white solid; ^{1}H NMR (600 MHz, CD_3CN): δ = 8.05 (d, *J* = 8.5 Hz, 2H), 7.91 (d, *J* = 7.8 Hz, 2H), 7.69 (d, *J* = 8.5 Hz, 2H), 7.58 (t, *J* = 7.4 Hz, 1H), 7.54 – 7.49 (m, 2H), 7.46 (t, *J* = 7.7 Hz, 2H), 7.21 (d, *J* = 8.1 Hz, 2H), 7.01 (s, 1H), 5.35 (s, 1H), 4.41 (dd, *J* = 11.0, 2.3 Hz, 1H), 3.90 (dd, *J* = 17.6, 11.0 Hz, 1H), 3.66 (s, 3H), 3.65 – 3.61 (m, 1H), 3.57 – 3.50 (m, 8H), 3.47 – 3.42 (m, 2H), 3.40 (t, *J* = 5.3 Hz, 2H), 3.10 (q, *J* = 5.7 Hz, 2H), 2.75 (s, 3H), 1.36 ppm (s, 9H); ^{13}C NMR (151 MHz, CD_3CN): δ = 198.84, 176.27, 173.19, 171.09, 170.51, 167.37, 156.89, 142.79, 137.77, 134.62, 134.18, 132.62, 132.43, 131.79, 131.45, 129.61, 128.97, 127.63, 127.07, 88.22, 79.25, 70.89, 70.86, 70.58, 70.16, 57.90, 53.54, 53.12, 49.60, 41.90, 40.99, 40.35, 28.60, 25.51 ppm; HRMS: calcd. for $[M+H]^+$ $C_{42}H_{48}{}^{79}BrO_{10}N_4$ =

847.25483, found: 847.25687; calcd. for $[M+H]^+$ $C_{42}H_{48}{}^{81}BrO_{10}N_4$ = 849.25279, found: 849.25538.

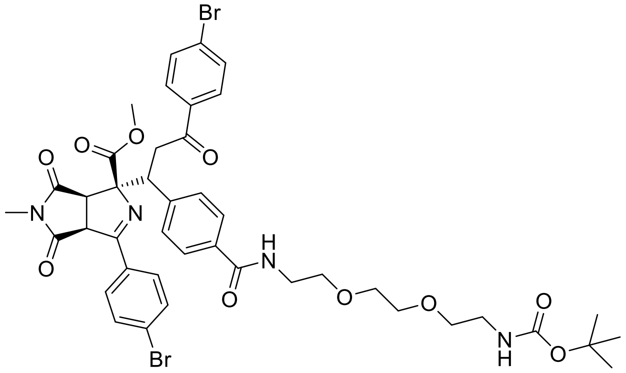

Methyl (1*S*,3a*S*,6a*R*)-3-(4-bromophenyl)-1-((*R*)-3-(4-bromophenyl)-1-(4-((2,2-dimethyl-4-oxo-3,8,11-trioxa-5-azatridecan-13-yl)carbamoyl)phenyl)-3-oxopropyl)-5-methyl-4,6-dioxo-1,3a,4,5,6,6a-hexahydropyrrolo[3,4-c]pyrrole-1-carboxylate (S10b)

21% yield; amorphous white solid; ^{1}H NMR (600 MHz, CD_3CN): δ = 8.04 (d, *J* = 8.5 Hz, 2H), 7.81 (d, *J* = 8.5 Hz, 2H), 7.69 (d, *J* = 8.5 Hz, 2H), 7.63 (d, *J* = 8.5 Hz, 2H), 7.51 (d, *J* = 7.9 Hz, 2H), 7.21 (d, *J* = 7.90 Hz, 2H), 7.01 (s, 1H), 5.34 (s, 1H), 4.39 (dd, *J* = 10.8, 2.5 Hz, 1H), 3.85 (dd, *J* = 17.6, 10.8 Hz, 1H), 3.65 (s, 3H), 3.63 (dd, *J* = 17.6, 2.5 Hz, 1H), 3.57 – 3.49 (m, 8H), 3.48 – 3.42 (m, 2H), 3.40 (t, *J* = 5.2 Hz, 2H), 3.10 (q, *J* = 5.7 Hz, 2H), 2.75 (s, 3H), 1.36 ppm (s, 9H); ^{13}C NMR (151 MHz, CD_3CN): δ = 198.05, 176.26, 173.18, 171.07, 170.57, 167.36, 156.91, 142.64, 136.67, 134.66, 132.77, 132.62, 132.44, 131.76, 131.44, 130.85, 128.60, 127.66, 127.10, 88.17, 79.26, 70.89, 70.86, 70.60, 70.17, 57.91, 53.56, 53.08, 49.51, 41.93, 40.99, 40.35, 28.60, 25.51 ppm; HRMS: calcd. for $[M+H]^+$ $C_{42}H_{47}{}^{79}Br_2O_{10}N_4$ = 925.16535, found: 925.16781; calcd. for $[M+H]^+$ $C_{42}H_{47}{}^{79}Br^{81}BrO_{10}N_4$ = 927.16330, found: 927.16592; calcd. for $[M+H]^+$ $C_{42}H_{47}{}^{81}Br_2O_{10}N_4$ = 929.16125, found: 929.16440.

Cleavage of the Boc-protecting group

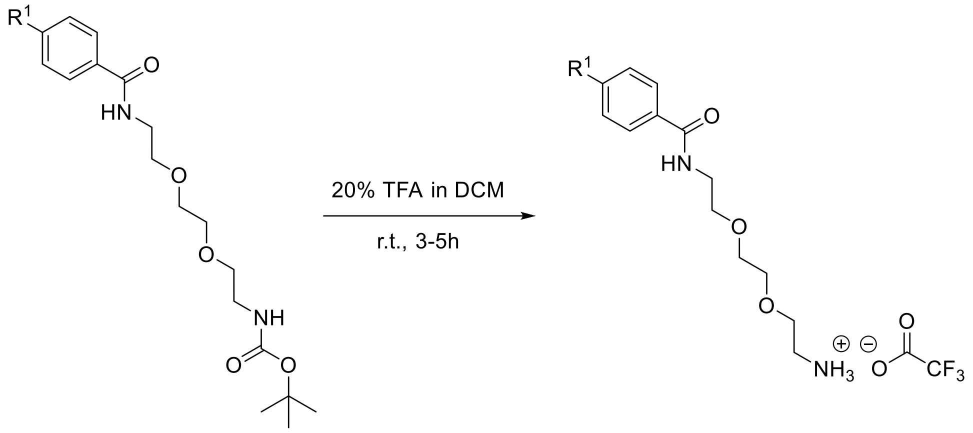

S10a/S10b **S11a/S11b**

The Boc-protected amine (1 equiv., 3.5 µmol) was solved in 20% TFA in DCM. The solution was stirred for 3-5h at ambient temperature. The solvent was removed under reduced pressure and the product was dried under high vacuum.

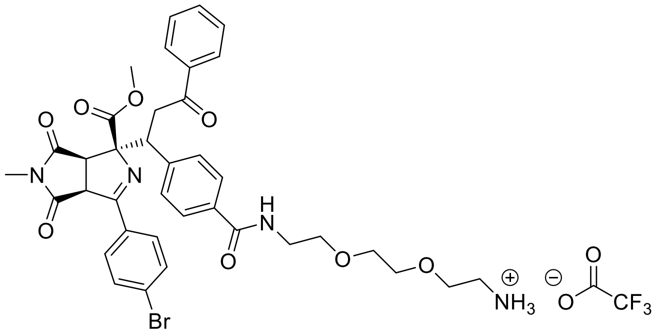

2-(2-(2-(4-((*S*)-1-((1*S*,3a*S*,6a*R*)-3-(4-bromophenyl)-1-(methoxycarbonyl)-5-methyl-4,6-dioxo-1,3a,4,5,6,6a-hexahydropyrrolo[3,4-*c*]pyrrol-1-yl)-3-oxo-3-phenylpropyl)benz-amido)ethoxy)ethoxy)ethan-1-aminium 2,2,2-trifluoroacetate (S11a: numbered 8 in the manuscript)

Quantitative yield; amorphous white solid; ^{1}H NMR (400 MHz, CD_3CN): δ = 8.04 (d, *J* = 8.6 Hz, 2H), 7.96 – 7.90 (m, 2H), 7.70 (d, *J* = 8.6 Hz, 2H), 7.59 (t, *J* = 7.4 Hz, 1H), 7.53 (d, *J* = 8.3 Hz, 2H), 7.47 (t, *J* = 7.7 Hz, 2H), 7.25 (d, *J* = 8.2 Hz, 3H), 6.79 (s, 3H), 4.42 (dd, *J* = 11.0, 2.5 Hz, 1H), 3.92 (dd, *J* = 17.6, 11.0 Hz, 1H), 3.71 – 3.63 (m, 4H), 3.63 – 3.46 (m, 12H), 3.06 – 2.97 (m, 2H), 2.76 (s, 3H); ^{13}C NMR (101 MHz, CD_3CN): δ = 198.93, 176.25, 173.20, 171.05, 170.50, 143.28, 137.74, 134.26, 132.63, 132.43, 131.82, 131.59, 129.65, 128.99, 127.68, 127.10, 88.22, 71.11, 70.74, 67.01, 57.91, 53.57, 53.13, 49.62, 41.83, 40.67, 40.34, 25.54 ppm; HRMS: calcd. for $[M+H]^+$ $C_{37}H_{40}{}^{79}BrO_8N_4$ = 747.20240, found: 747.20523; calcd. for $[M+H]^+$ $C_{37}H_{40}{}^{81}BrO_8N_4$ = 749.20036, found: 749.20256.

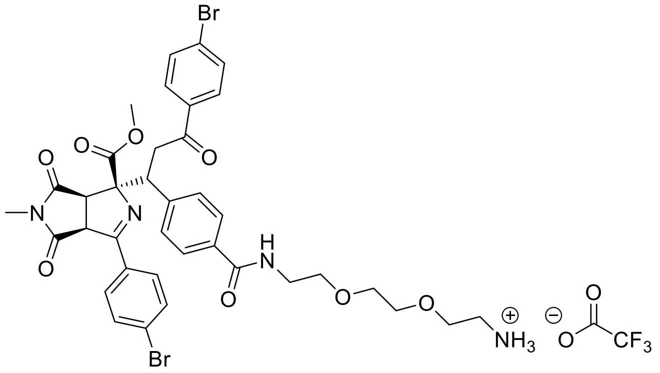

2-(2-(2-(4-((S)-3-(4-bromophenyl)-1-((1S,3aS,6aR)-3-(4-bromophenyl)-1-(methoxy-carbonyl)-5-methyl-4,6-dioxo-1,3a,4,5,6,6a-hexahydropyrrolo[3,4-c]pyrrol-1-yl)-3-oxopropyl)benz-amido)ethoxy)ethoxy)ethan-1-aminium 2,2,2-trifluoroacetate (S11b: numbered 9 in the manuscript)

Quantitative yield; amorphous white solid; ^{1}H NMR (400 MHz, CD_3CN): δ = 8.04 (d, *J* = 8.6 Hz, 2H), 7.82 (d, *J* = 8.6 Hz, 2H), 7.70 (d, *J* = 8.6 Hz, 2H), 7.64 (d, *J* = 8.6 Hz, 2H), 7.53 (d, *J* = 8.2 Hz, 2H), 7.24 (d, *J* = 8.2 Hz, 3H), 6.90 (s, 3H), 4.40 (dd, *J* = 10.8, 2.6 Hz, 1H), 3.86 (dd, *J* = 17.6, 10.8 Hz, 1H), 3.66 (d, *J* = 5.8 Hz, 3H), 3.63 – 3.46 (m, 12H), 3.02 (d, *J* = 3.1 Hz, 2H), 2.76 ppm (s, 3H); ^{13}C NMR (101 MHz, CD_3CN): δ = 198.12, 176.24, 173.18, 171.04, 170.56, 143.08, 136.65, 134.23, 132.80, 132.63, 132.43, 131.79, 131.56, 130.87, 128.67, 127.71, 127.12, 88.18, 71.08, 70.75, 67.04, 57.92, 53.59, 53.07, 49.52, 41.86, 40.62, 40.35, 25.53 ppm; HRMS: calcd. for $[M+H]^+$ $C_{37}H_{39}{}^{79}Br_2O_8N_4$ = 825.11292, found: 825.11557; calcd. for $[M+H]^+$ $C_{37}H_{39}{}^{79}Br^{81}BrO_8N_4$ = 827.11087, found: 827.11329; calcd. for $[M+H]^+$ $C_{37}H_{39}{}^{81}Br_2O_8N_4$ = 829.10882, found: 829.11166.

Preparation of S12

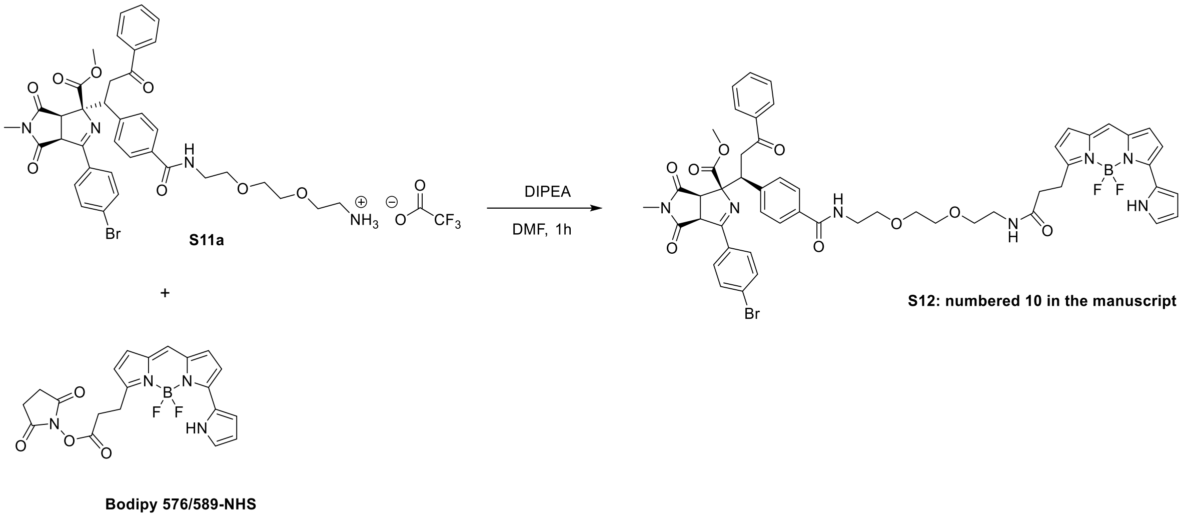

Methyl (1S,3aS,6aR)-3-(4-bromophenyl)-1-((S)-1-(4-((2-(2-(2-(3-(5,5-difluoro-7-(1H-pyrrol-2-yl)-5H-4l4,5l4-dipyrrolo[1,2-c:2',1'-f][1,3,2]diazaborinin-3-yl)propanamido)ethoxy)ethoxy)ethyl)carbamoyl)phenyl)-3-oxo-3-phenylpropyl)-5-methyl-4,6-dioxo-1,3a,4,5,6,6a-hexahydropyrrolo[3,4-c]pyrrole-1-carboxylate (S12: numbered 10 in the manuscript)

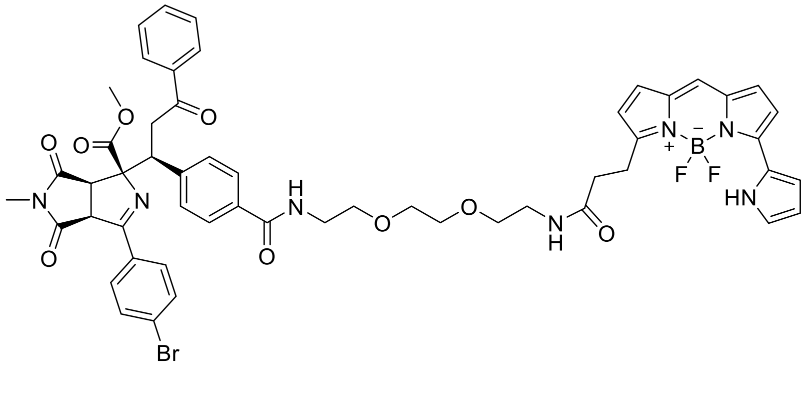

To a solution of S11a (0.008 mmol, 6 mg) and DIPEA (4 µl, 0.024 mmol, 3 equiv.) in 2 ml DMF, Bodipy576/589-NHS (4 mg, 0.009 mmol, 1.1 equiv.) was added. The reaction mixture was stirred at room temperature for 1h under ambient environment before concentrating under vacuo. The remaining crude was purified over RP-HPLC (Nucleodur C18 Gravity, 21 mm × 125 mm, 5 µm) then lyophilized yielding 3 mg S12.

38% yield; purple solid; ^{1}H NMR (700 MHz, CDCl3) δ 10.37 (s, 1H), 8.01 – 7.98 (m, 2H), 7.91 (dd, J = 8.3, 1.3 Hz, 2H), 7.63 – 7.61 (m, 2H), 7.54 – 7.51 (m, 1H), 7.50 (d, J = 8.0 Hz,

2H), 7.44 – 7.40 (m, 2H), 7.15 – 7.12 (m, 3H), 7.02 (d, J = 4.6 Hz, 1H), 6.97 – 6.95 (m, 2H), 6.84 (d, J = 4.5 Hz, 1H), 6.81 (d, J = 3.9 Hz, 1H), 6.34 (dt, J = 4.0, 2.4 Hz, 1H), 6.26 (d, J = 3.9 Hz, 1H), 4.52 (dd, J = 11.0, 2.6 Hz, 1H), 3.81 (dd, J = 17.5, 11.0 Hz, 1H), 3.76 (s, 3H), 3.70 (dd, J = 17.5, 2.7 Hz, 1H), 3.58 – 3.49 (m, 7H), 3.48 – 3.44 (m, 3H), 3.36 (q, J = 5.4 Hz, 2H), 3.30 (d, J = 8.2 Hz, 1H), 3.28 (t, J = 7.7 Hz, 2H), 2.84 (s, 3H), 2.60 (t, J = 7.7 Hz, 2H); ^{13}C NMR (176 MHz, CDCl3) δ 197.51, 175.04, 171.73, 170.22, 169.67, 167.05, 155.33, 150.66, 141.94, 137.47, 136.66, 133.92, 133.62, 133.41, 132.05, 131.77, 131.38, 130.44, 130.21, 128.77, 128.25, 127.25, 127.12, 126.48, 126.07, 123.74, 123.45, 120.54, 118.05, 117.00, 111.69, 87.56, 77.34, 77.16, 76.98, 70.33, 70.25, 69.88, 69.81, 56.76, 53.30, 52.21, 48.82, 41.44, 39.82, 39.27, 35.98, 29.85, 25.42 ppm; HRMS: calcd. for $[M+H]^+$ $C_{53}H_{52}O_9N_7BBrF_2$ 1058.30655; Found 1058.30852. Calculated for $C_{53}H_{52}O_9N_7B^{81}BrF_2$ 1060.30451; Found 1058.30663.

X-ray Crystallographic data

Table S14. X-ray data of 5a.

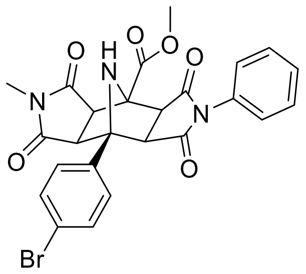

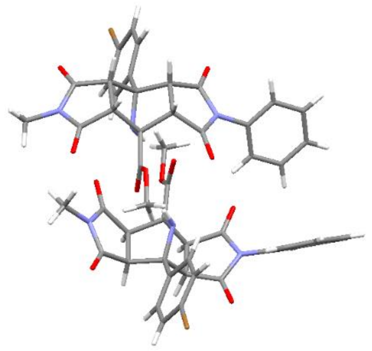

Empirical formula	$C_{25}H_{20}BrN_3O_6$
Formula weight	538.35
Temperature/K	100.0
Crystal system	monoclinic
Space group	C2
a/Å	27.739(3)
b/Å	10.3884(11)
c/Å	19.799(2)
α/°	90
β/°	102.043(4)
γ/°	90
Volume/Å^3	5579.9(10)
Z	8
ρ_{calc}g/cm^3	1.282
μ/mm^{-1}	1.513
F(000)	2192.0
Crystal size/mm^3	0.787 × 0.097 × 0.09
Radiation	MoKα (λ = 0.71073)
2Θ range for data collection/°	4.554 to 54
Index ranges	-35 ≤ h ≤ 35, -13 ≤ k ≤ 13, -25 ≤ l ≤ 25
Reflections collected	184891

Independent reflections	12179 [R_{int} = 0.0670, R_{sigma} = 0.0264]
Data/restraints/parameters	12179/2/639
Goodness-of-fit on F^2	1.009
Final R indexes [I>=2σ (I)]	R_1 = 0.0467, wR_2 = 0.1086
Final R indexes [all data]	R_1 = 0.0546, wR_2 = 0.1139
Largest diff. peak/hole / e Å^{-3}	0.85/-0.81

Table S15. X-ray data of *rac*-6a.

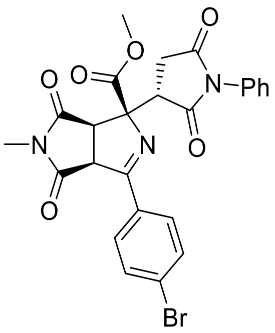

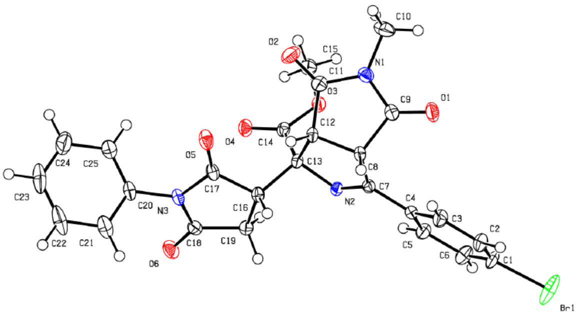

Empirical formula	$C_{25}H_{20}BrN_3O_6$
Formula weight	538.35
Temperature/K	150
Crystal system	monoclinic
Space group	$P2_1/c$
a/Å	14.8112(3)
b/Å	8.8480(2)
c/Å	17.9402(6)
α/°	90
β/°	103.306(3)
γ/°	90
Volume/Å^3	2287.94(11)
Z	4

ρ_{calc}g/cm^3	1.563
μ/mm^{-1}	1.845
F(000)	1096.0
Crystal size/mm^3	0.23 × 0.2 × 0.14
Radiation	MoKα (λ = 0.71073)
2Θ range for data collection/°	4.666 to 58.374
Index ranges	$-19 \leq h \leq 20$, $-11 \leq k \leq 12$, $-24 \leq l \leq 24$
Reflections collected	54572
Independent reflections	5843 [R_{int} = 0.0398, R_{sigma} = 0.0254]
Data/restraints/parameters	5843/0/318
Goodness-of-fit on F^2	1.057
Final R indexes [I>=2σ (I)]	R_1 = 0.0413, wR_2 = 0.0945
Final R indexes [all data]	R_1 = 0.0619, wR_2 = 0.1048
Largest diff. peak/hole / e Å^{-3}	1.16/-1.59

Table S16. X-ray data of 7h.

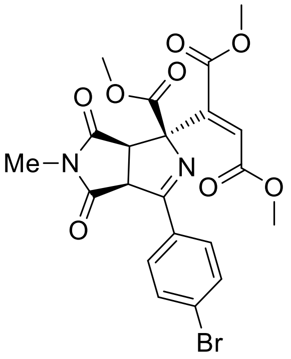

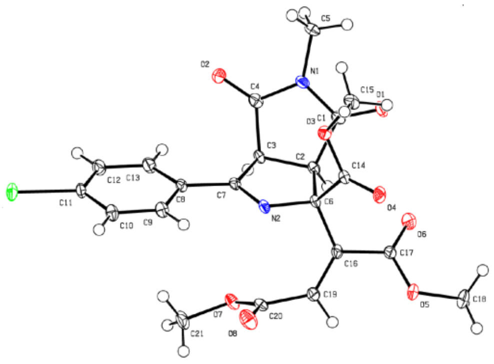

Empirical formula	C21H19BrN2O8
Formula weight	507.29
Temperature/K	100.01
Crystal system	orthorhombic

Space group	P212121
a/Å	9.5089(4)
b/Å	9.6773(4)
c/Å	23.1036(10)
α/°	90
β/°	90
γ/°	90
Volume/Å3	2126.00(16)
Z	4
ρcalcg/cm3	1.585
μ/mm-1	3.100
F(000)	1032.0
Crystal size/mm3	0.93 × 0.53 × 0.45
Radiation	CuKα (λ = 1.54178)
2Θ range for data collection/°	7.652 to 151.928
Index ranges	-11 ≤ h ≤ 11, -11 ≤ k ≤ 12, -28 ≤ l ≤ 25
Reflections collected	19473
Independent reflections	4280 [Rint = 0.0365, Rsigma = 0.0317]
Data/restraints/parameters	4280/0/293
Goodness-of-fit on F2	1.104
Final R indexes [I>=2σ (I)]	R1 = 0.0248, wR2 = 0.0605
Final R indexes [all data]	R1 = 0.0249, wR2 = 0.0606
Largest diff. peak/hole / e Å-3	0.36/-0.78

The crystallographic data for the structure of **7l** has been published as supplementary publication number 2047703 in the Cambridge Crystallographic Data Centre. A copy of these data can be obtained for free by applying to CCDC, 12 Union Road, Cambridge CB2 IEZ, UK, fax: 144-(0)1223-336033 or e-mail: deposit@ccdc.cam.ac.uk.

Table S17. X-ray data of 7l.

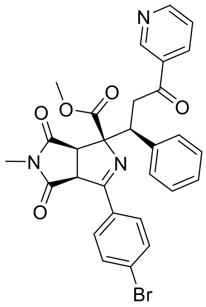

7l

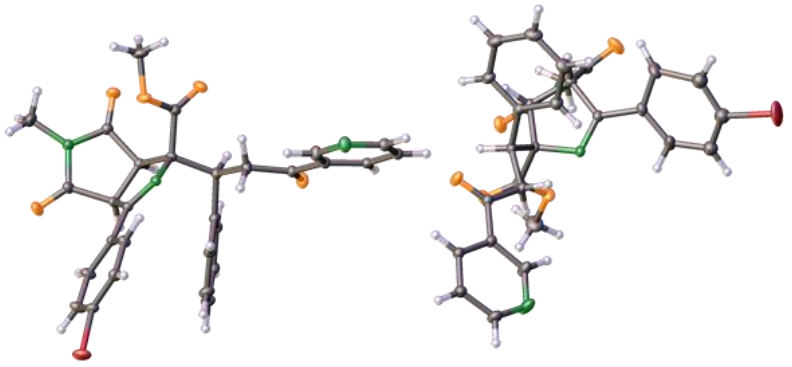

Compound	**7l**
Empirical formula	$C_{29}H_{24}BrN_3O_5$
Formula weight	574.42
Temperature/K	100.0
Crystal system	monoclinic
Space group	$P2_1$
a/Å	14.9507(6)
b/Å	9.4671(3)
c/Å	18.8434(7)
α/°	90
β/°	105.763(2)
γ/°	90
Volume/Å^3	2566.79(16)
Z	4
ρ_{calc}g/cm^3	1.486
μ/mm^{-1}	1.647
F(000)	1176.0
Crystal size/mm^3	0.557 × 0.247 × 0.158

Radiation	MoKα (λ = 0.71073)
2Θ range for data collection/°	4.064 to 66.46
Index ranges	$-23 \leq h \leq 23$, $-14 \leq k \leq 14$, $-29 \leq l \leq 29$
Reflections collected	419925
Independent reflections	19625 [R_{int} = 0.0343, R_{sigma} = 0.0125]
Data/restraints/parameters	19625/1/710
Goodness-of-fit on F^2	1.062
Final R indexes [I>=2σ (I)]	R_1 = 0.0252, wR_2 = 0.0682
Final R indexes [all data]	R_1 = 0.0267, wR_2 = 0.0691
Largest diff. peak/hole / e Å^{-3}	1.28/–0.90
Flack parameter	–0.0011(9)

Absolute configuration of 7a

The configuration of the additional stereocenter in **7a** has been determined by means of VCD spectroscopy. Experimental IR and VCD spectra were obtained for a solution of **7a** in $CDCl_3$ (87 mM ≈ 50 mg/ml) at 100 μm path length. The spectra were recorded on a Bruker Vertex 70 equipped with a PMA 50 module for VCD measurements accumulating about 18000 scans for the VCD spectrum. The baseline was corrected by subtraction of the solvent spectrum.

In order to analyse the experimental IR and VCD spectra, a conformational analysis followed by spectra calculations for all possible stereoisomers was carried out. As most stereocenters could be deduced from crystallographic data of other products, solely the new and thus unknown stereocenter of the side group had to be evaluated. Hence, only two stereoisomers (with R and S configuration in the side group) were considered. At the B3LYP/6-31+G(2d,p)/IEFPCM($CHCl_3$) level of theory, both isomers were found to feature four

conformers (Table S12), of which not all are contributing equally to the overall conformational distribution.

Table S18. Relative energies for the conformers of (S)-7a and (R)-7a

	#	ΔE[1]	ΔG[1]	Pop-ΔE[2]	Pop-ΔG[2]
**(S)-7a**	C1	0.23	0.34	39.49	34.37
	C2	3.44	2.42	0.18	1.02
	C3	2.12	1.74	1.63	3.25
	C4	0.00	0.00	58.70	61.36
**(R)-7a**	C1	0.55	0.92	18.74	11.62
	C2	0.25	0.36	31.53	30.08
	C3	1.89	1.64	1.98	3.44
	C4	0.00	0.00	47.76	54.86

[1] in kcal/mol

[2] population based on ΔE or ΔG in %

[a] with respect to E_{ZPC}= -4215.45464 hartree and G= -4215.52683 hartree

[b] with respect to E_{ZPC}= -4215.45238 hartree and G= -4215.52432 hartree

Based on the relative Gibbs free energies given in **Table S18**, we simulated the IR and VCD spectra for both isomers by Boltzmann-averaging of the corresponding single-conformer spectra. A comparison of the obtained final spectra with the experimental IR and VCD spectra is provided in **Figure S14.** The visual comparison of the predicted VCD pattern of **(S)-7a** reveals a much better agreement with the experimental data than for the (R)-isomer. The most important and characteristic bands are highlighted in the figure, and can be found in the regions between 1300-1200 and 1100-1000 cm^{-1}. In particular the latter region of the spectrum is very characteristic, as only the (S)-isomer spectrum features the experimentally observed –/–/+/–/– pattern. Hence, from this VCD analysis, it is concluded that the unknown stereocenter in **7a** features an (S)-configuration.

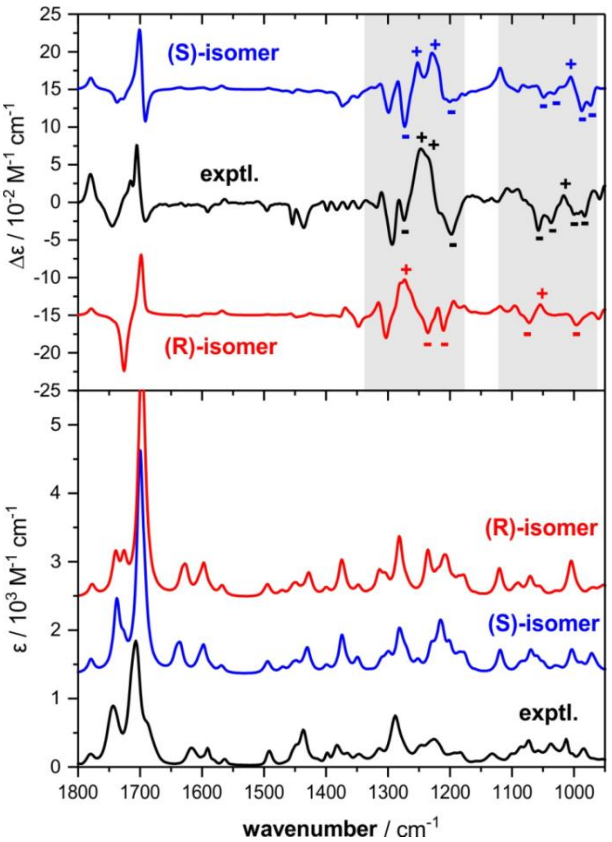

Supplementary Figure S14. Comparison of the obtained computed spectra of the (S)- and (R)-diastereomers of **7a** with the experimental IR and VCD spectra

Table S19. Quantitative comparison of the experimental spectra of **7a-epi** with the two considered diastereomers *(S)*- and *(R)*-**7a**.

**calcd.**	**σ**	**Σ_{IR}**	**Σ_{VCD}**	**Δ**	**Σ_{VDF}**
(S)	0.98	83	63	67	60
(R)	0.99	84	26	60	10

HPLC-MS trace of 7l:

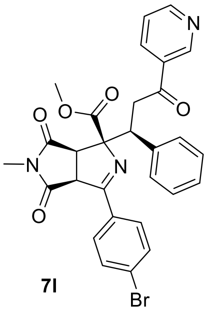

7l

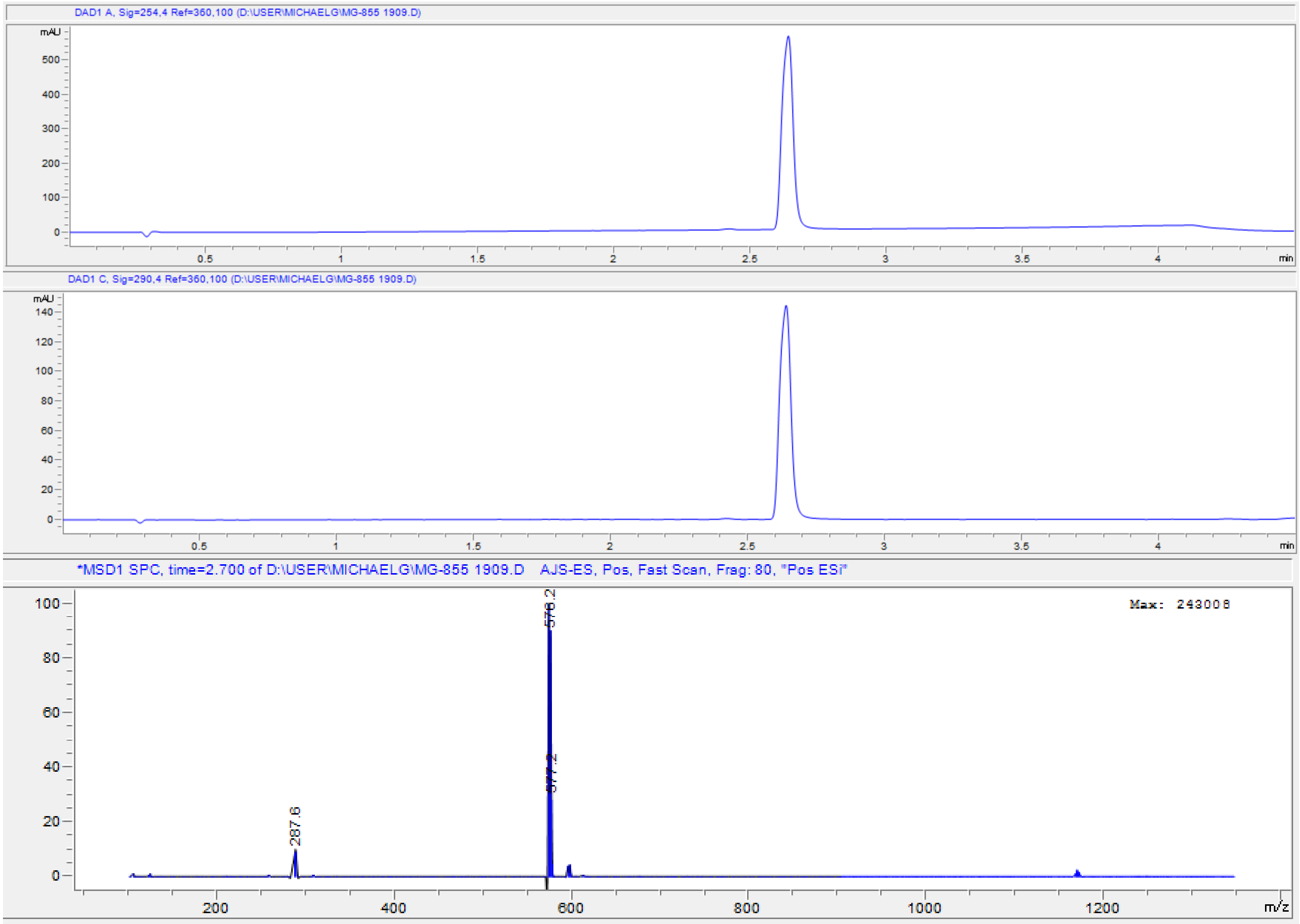

NMR and HPLC data

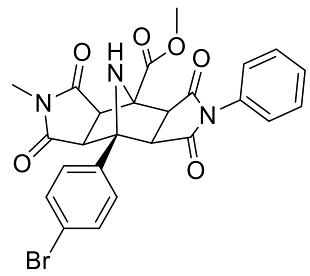

5a

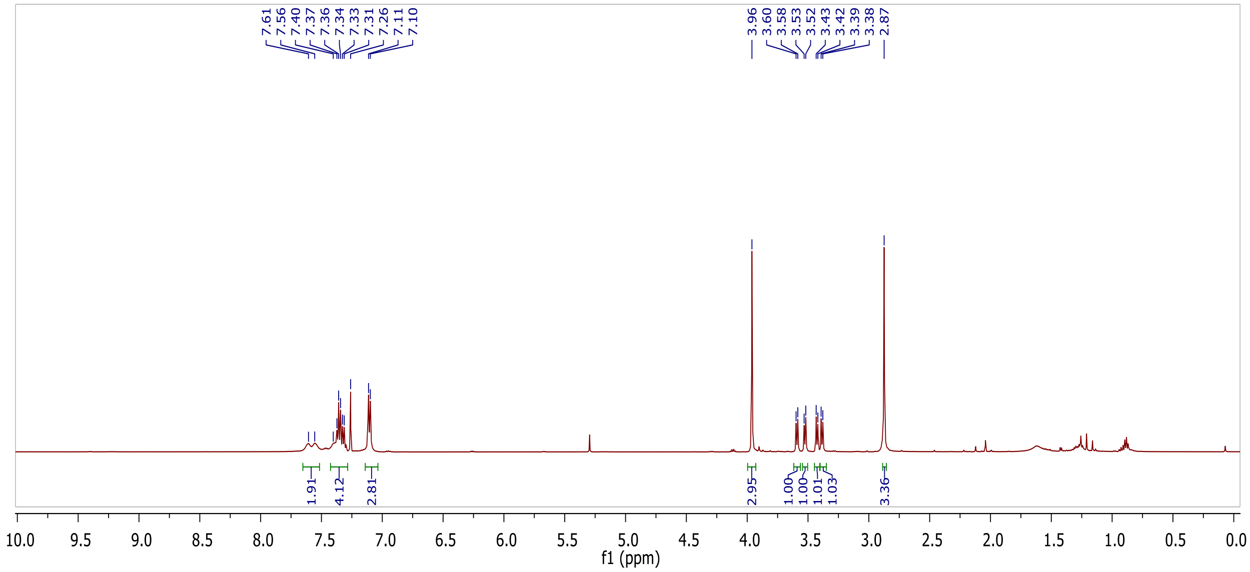

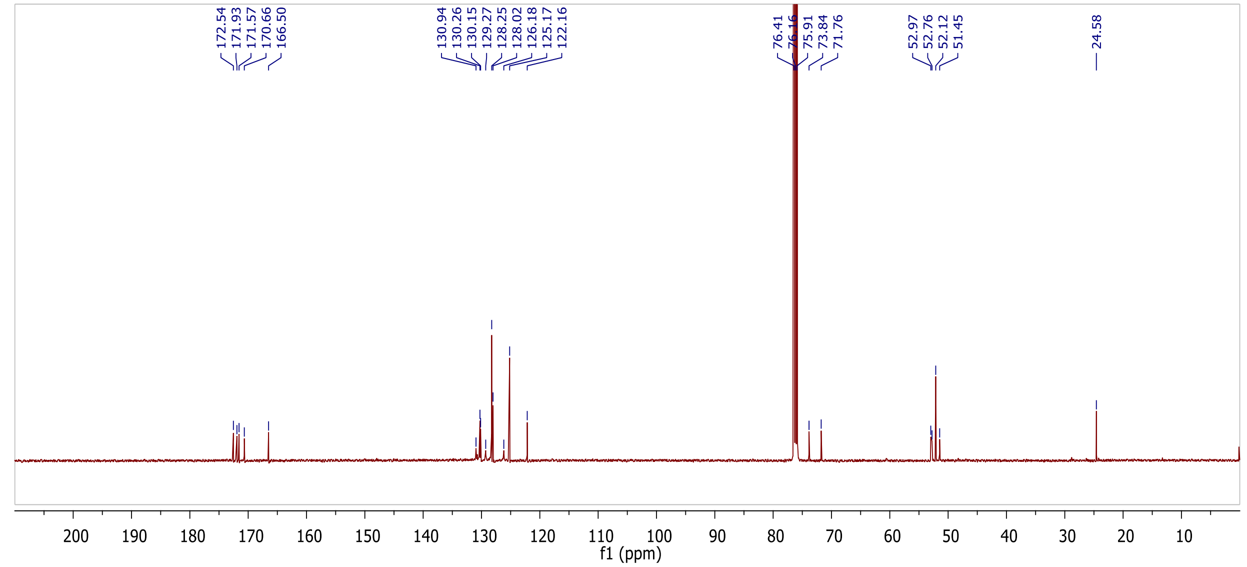

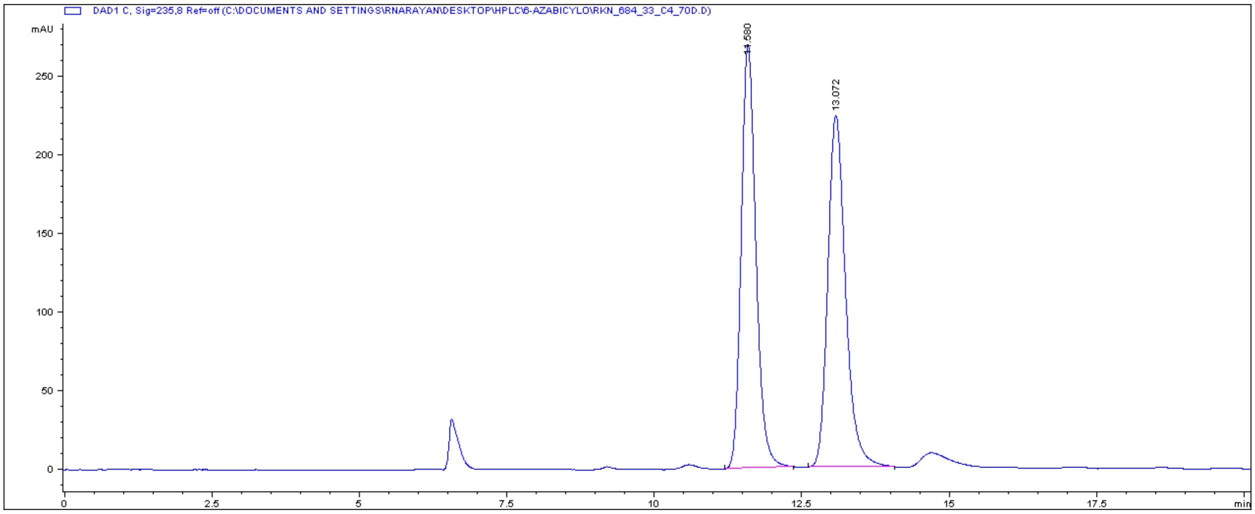

Signal 3: DAD1 C, Sig=235,8 Ref=off

Peak #	RetTime [min]	Type	Width [min]	Area [mAU*s]	Height [mAU]	Area %
1	11.580	BB	0.2662	4672.50830	269.60245	49.8565
2	13.072	BB	0.3190	4699.39697	223.78870	50.1435

Totals : 9371.90527 493.39114

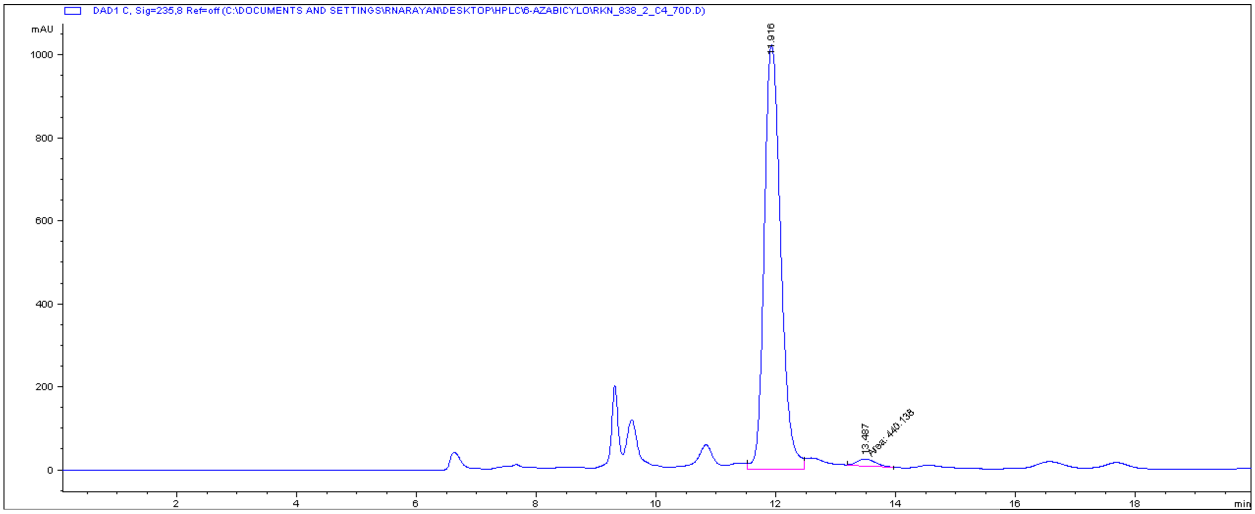

Signal 3: DAD1 C, Sig=235,8 Ref=off

Peak #	RetTime [min]	Type	Width [min]	Area [mAU*s]	Height [mAU]	Area %
1	11.916	VV	0.3007	1.97560e4	1025.88342	97.8207
2	13.487	MM	0.3912	440.13837	18.75139	2.1793

Totals : 2.01962e4 1044.63482

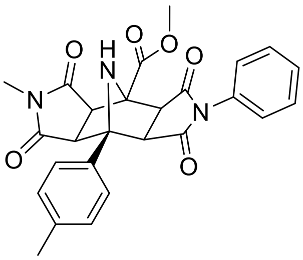
O
H
N
O
O
O
N
N
O
O

5b

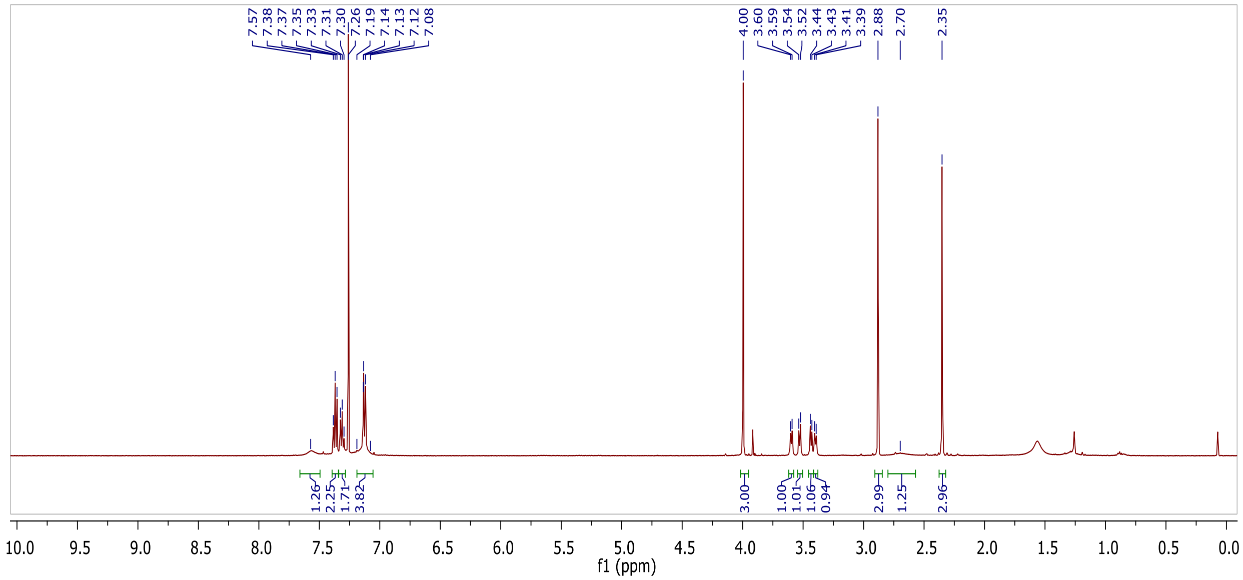
7.57
7.38
7.37
7.35
7.33
7.31
7.30
7.26
7.19
7.14
7.13
7.12
7.08
4.00
3.60
3.59
3.54
3.52
3.44
3.43
3.41
3.39
2.88
2.70
2.35
1.26
2.25
1.71
3.82
3.00
1.00
1.01
1.06
0.94
2.99
1.25
2.96
10.0
9.5
9.0
8.5
8.0
7.5
7.0
6.5
6.0
5.5
5.0
4.5
4.0
3.5
3.0
2.5
2.0
1.5
1.0
0.5
0.0
f1 (ppm)

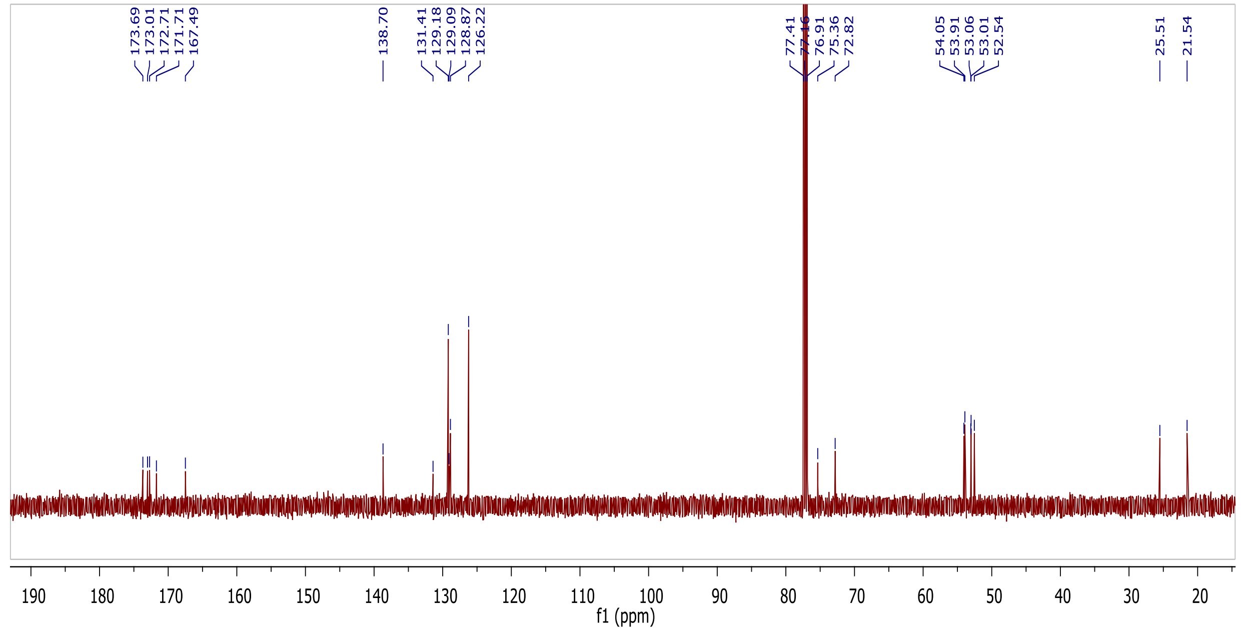
173.69
173.01
172.71
171.71
167.49
138.70
131.41
129.18
129.09
128.87
126.22
77.41
77.16
76.91
75.36
72.82
54.05
53.91
53.06
53.01
52.54
25.51
21.54
190
180
170
160
150
140
130
120
110
100
90
80
70
60
50
40
30
20
f1 (ppm)

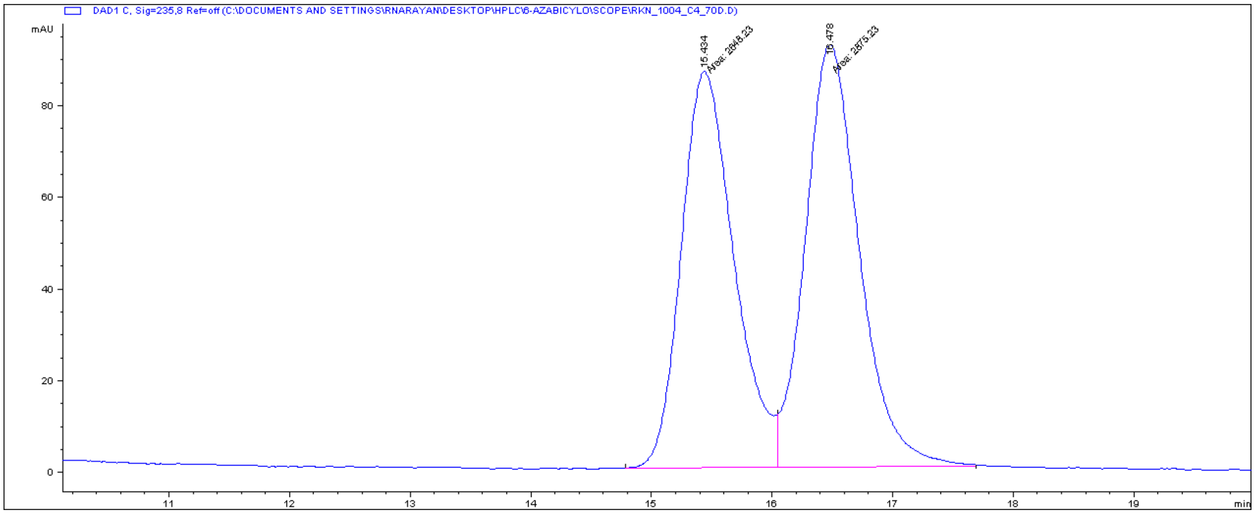

Signal 3: DAD1 C, Sig=235,8 Ref=off

Peak #	RetTime [min]	Type	Width [min]	Area [mAU*s]	Height [mAU]	Area %
1	15.434	MF	0.5187	2695.81665	86.62797	49.2406
2	16.478	FM	0.5045	2778.97266	91.80975	50.7594

Totals : 5474.78931 178.43772

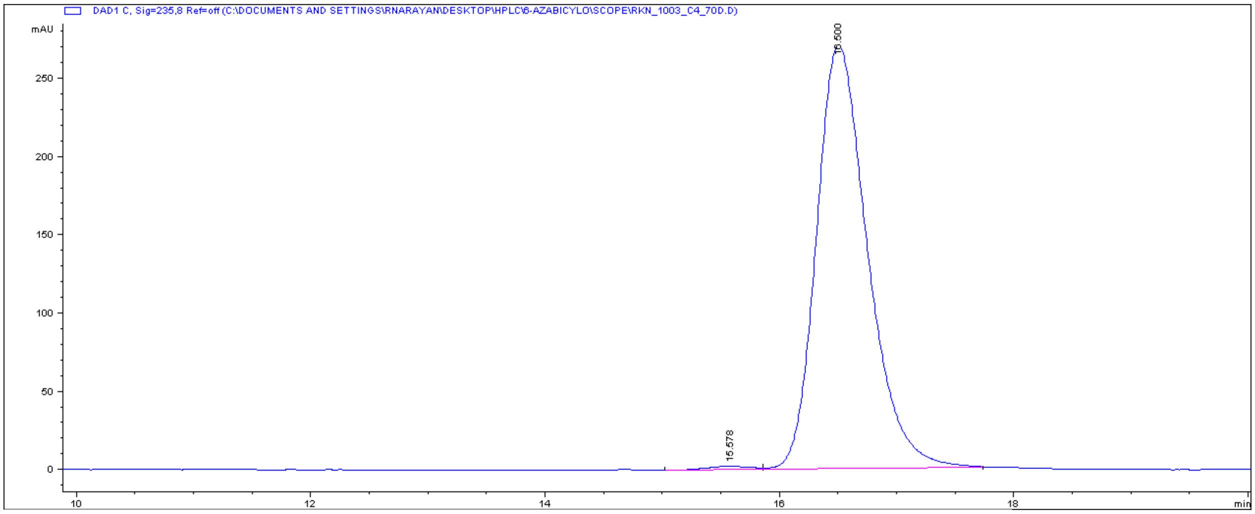

Signal 3: DAD1 C, Sig=235,8 Ref=off

Peak #	RetTime [min]	Type	Width [min]	Area [mAU*s]	Height [mAU]	Area %
1	15.578	BV	0.2975	52.77014	2.15626	0.6392
2	16.500	VB	0.4630	8202.32422	271.05862	99.3608

Totals : 8255.09436 273.21489

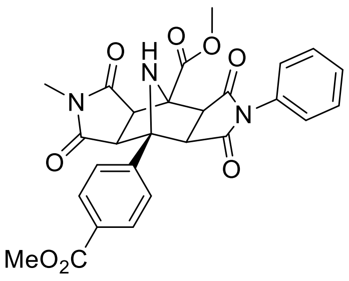
O
H
N
O
O
O
N
N
O
O
MeO2C

5c

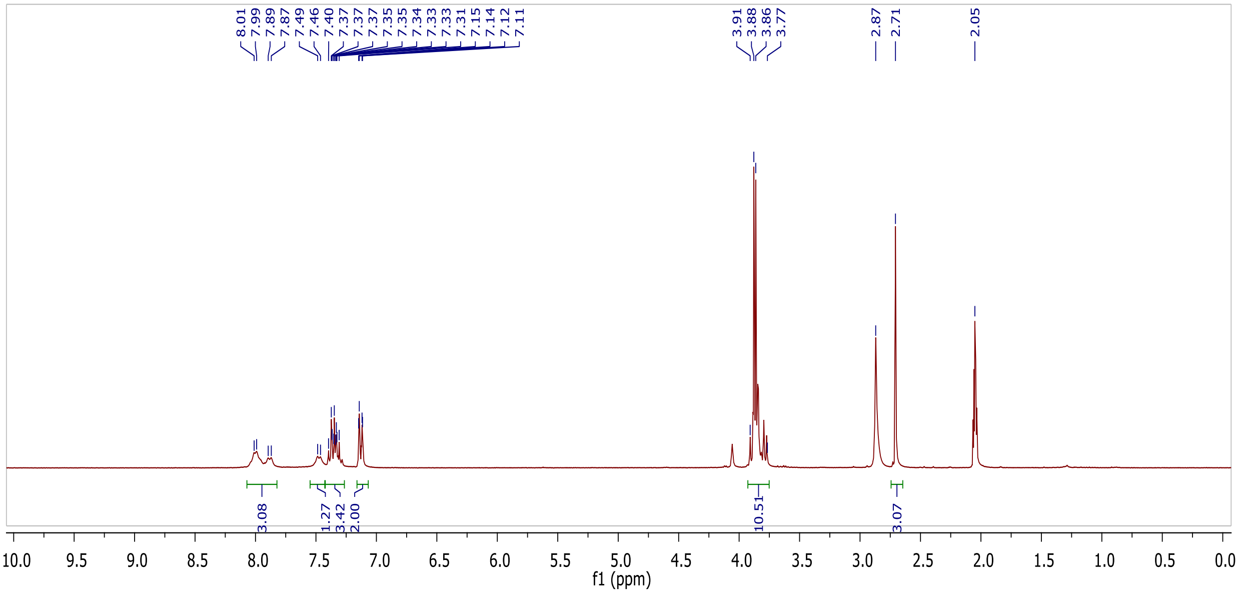
8.01
7.99
7.89
7.87
7.49
7.46
7.40
7.37
7.37
7.37
7.35
7.35
7.34
7.33
7.33
7.31
7.15
7.14
7.12
7.11
3.91
3.88
3.86
3.77
2.87
2.71
2.05
3.08
1.27
3.42
2.00
10.51
3.07
10.0 9.5 9.0 8.5 8.0 7.5 7.0 6.5 6.0 5.5 5.0 4.5 4.0 3.5 3.0 2.5 2.0 1.5 1.0 0.5 0.0
f1 (ppm)

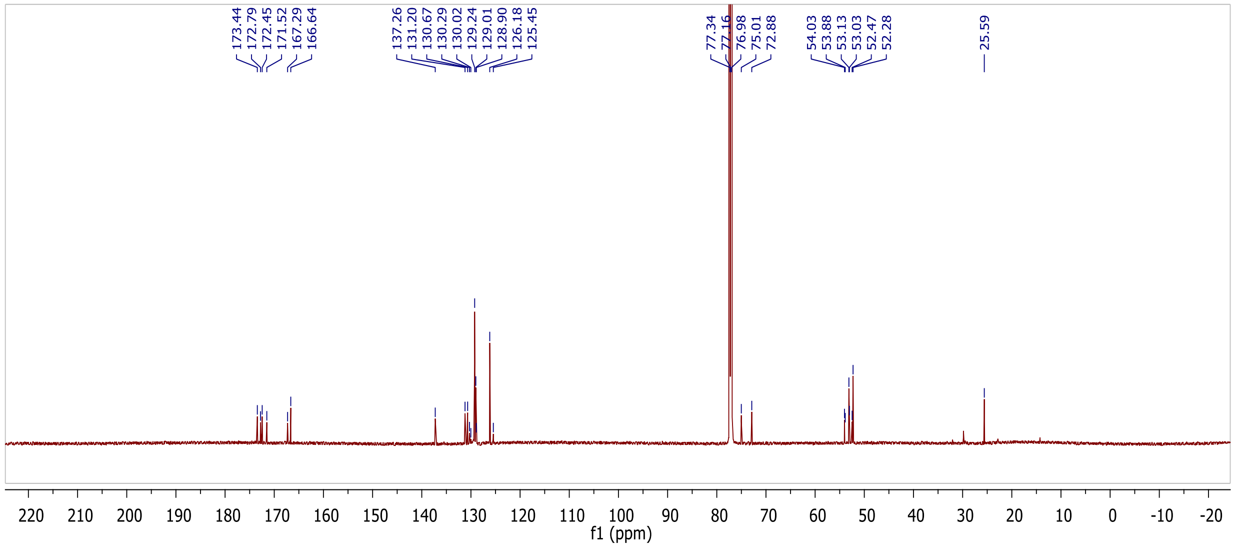
173.44
172.79
172.45
171.52
167.29
166.64
137.26
131.20
130.67
130.29
130.02
129.24
129.01
128.90
126.18
125.45
77.34
77.16
76.98
75.01
72.88
54.03
53.88
53.13
53.03
52.47
52.28
25.59
220 210 200 190 180 170 160 150 140 130 120 110 100 90 80 70 60 50 40 30 20 10 0 -10 -20
f1 (ppm)

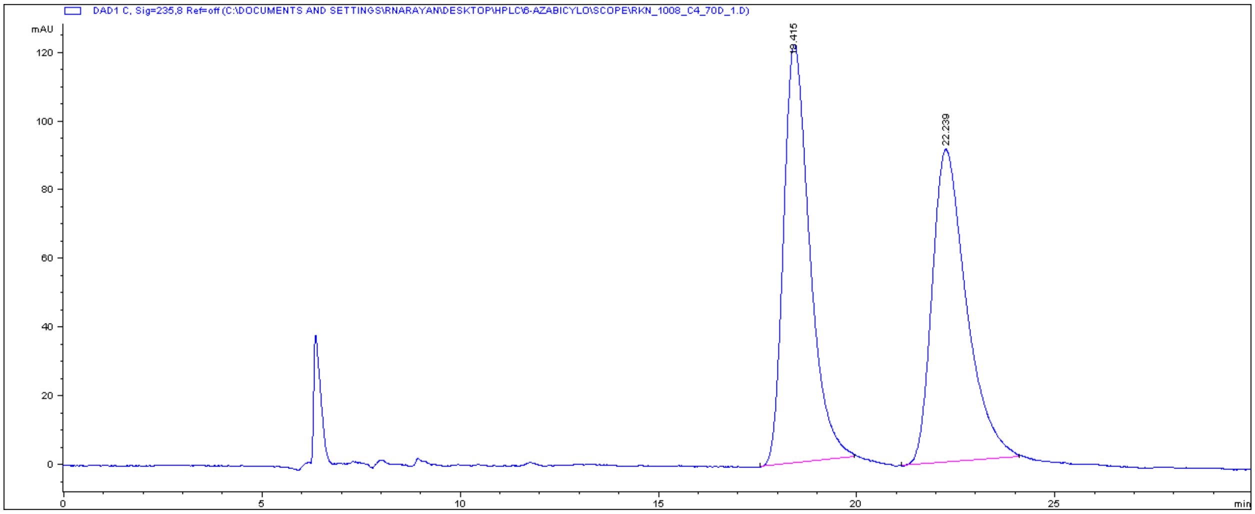

Signal 3: DAD1 C, Sig=235,8 Ref=off

Peak #	RetTime [min]	Type	Width [min]	Area [mAU*s]	Height [mAU]	Area %
1	18.415	BB	0.6779	5603.57031	121.98078	50.4651
2	22.239	VB	0.7186	5500.28223	91.33018	49.5349

Totals : 1.11039e4 213.31096

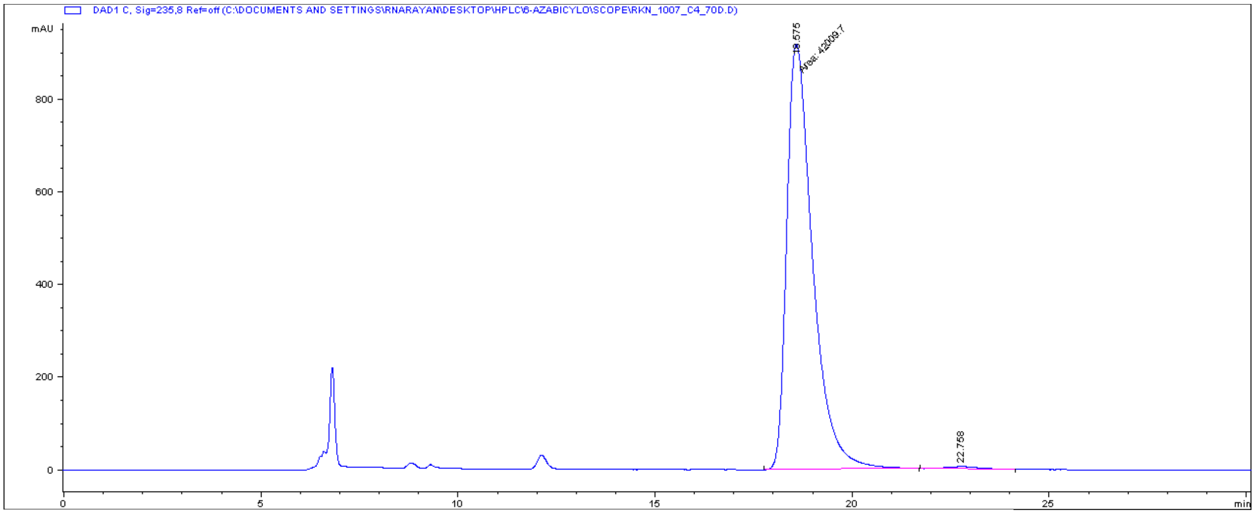

Signal 3: DAD1 C, Sig=235,8 Ref=off

Peak #	RetTime [min]	Type	Width [min]	Area [mAU*s]	Height [mAU]	Area %
1	18.575	MM	0.7623	4.20097e4	918.52069	99.3219
2	22.758	BV	0.6850	286.83105	4.95760	0.6781

Totals : 4.22965e4 923.47829

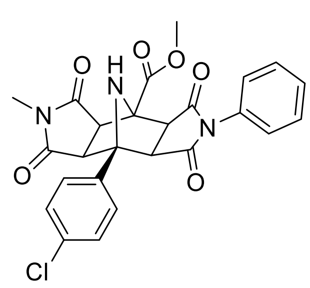
O
H
N
O
O
O
N
N
O
O
Cl

5d

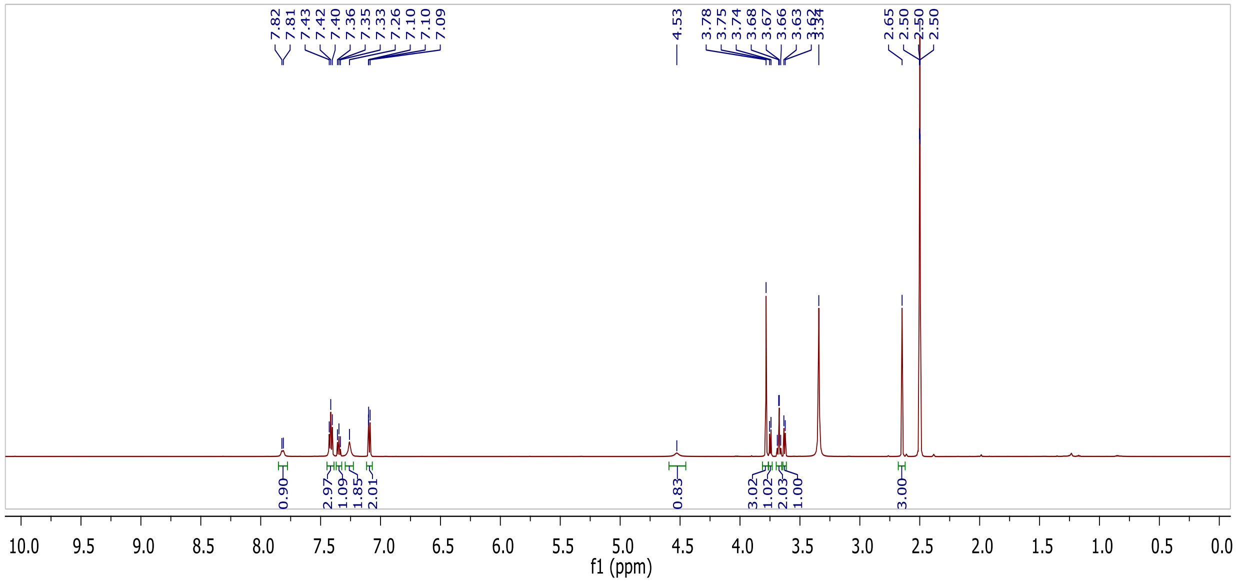
7.82
7.81
7.43
7.42
7.40
7.36
7.35
7.33
7.26
7.10
7.10
7.09
4.53
3.78
3.75
3.74
3.68
3.67
3.66
3.63
3.62
3.34
2.65
2.50
2.50
2.50
0.90
2.97
1.09
1.85
2.01
0.83
3.02
1.02
2.03
1.00
3.00
10.0 9.5 9.0 8.5 8.0 7.5 7.0 6.5 6.0 5.5 5.0 4.5 4.0 3.5 3.0 2.5 2.0 1.5 1.0 0.5 0.0
f1 (ppm)

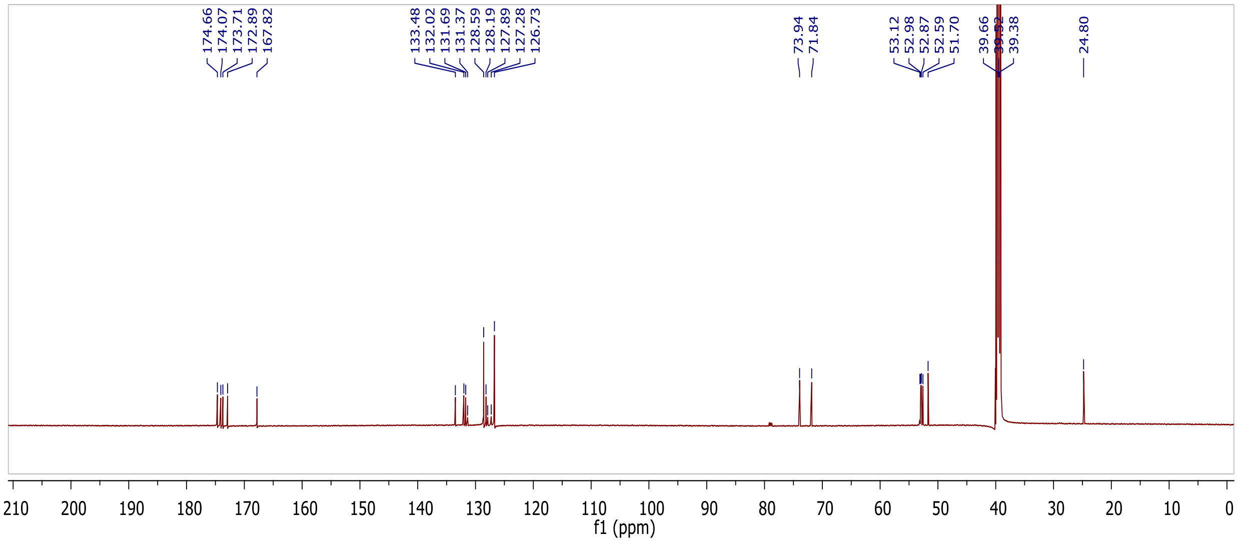
174.66
174.07
173.71
172.89
167.82
133.48
132.02
131.69
131.37
128.59
128.19
127.89
127.28
126.73
73.94
71.84
53.12
52.98
52.87
52.59
51.70
39.66
39.38
24.80
210 200 190 180 170 160 150 140 130 120 110 100 90 80 70 60 50 40 30 20 10 0
f1 (ppm)

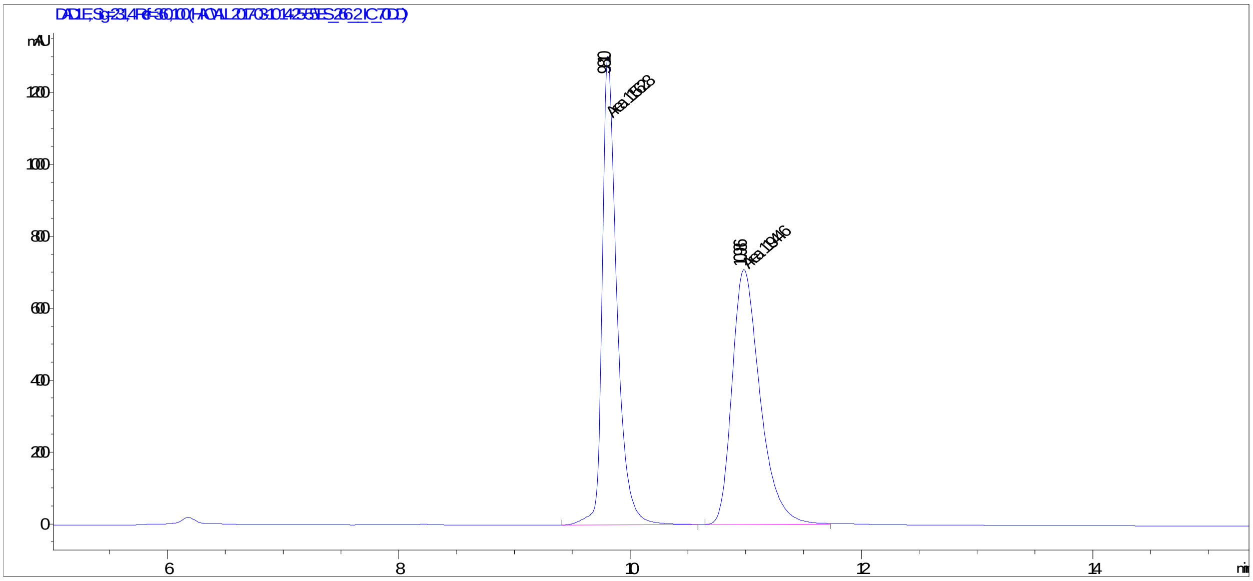

#	Time	Area	Height	Width	Area%	Symmetry
1	9.81	11562.8	1306.8	0.1475	49.188	0.617
2	10.986	11944.6	709.7	0.2805	50.812	0.701

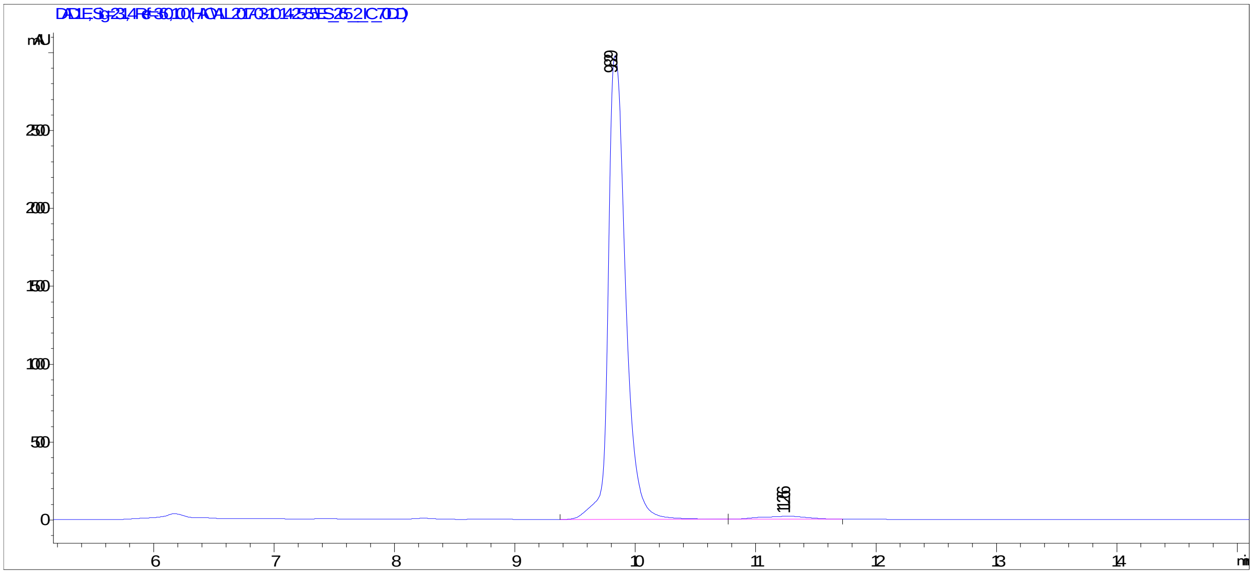

#	Time	Area	Height	Width	Area%	Symmetry
1	9.829	30014.6	2978.3	0.1556	98.275	0.624
2	11.266	527	19.4	0.3806	1.725	1.411

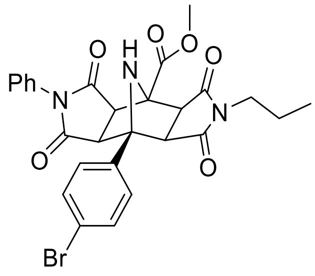
Ph
N
O
H
N
O
O
O
N
O
Br

5e

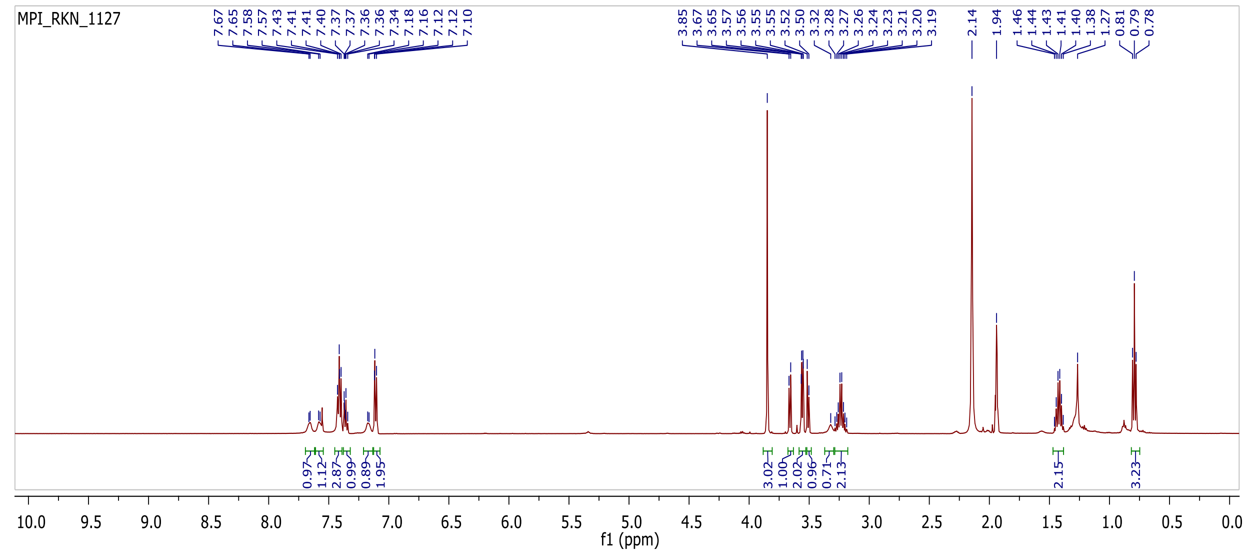
MPI_RKN_1127
7.67
7.65
7.58
7.57
7.43
7.41
7.40
7.37
7.37
7.36
7.36
7.34
7.18
7.16
7.12
7.12
7.10
3.85
3.67
3.65
3.57
3.56
3.55
3.55
3.52
3.50
3.32
3.28
3.27
3.26
3.24
3.23
3.21
3.20
3.19
2.14
1.94
1.46
1.44
1.43
1.41
1.40
1.38
1.27
0.81
0.79
0.78
0.97
1.12
2.87
0.99
0.89
1.95
3.02
1.00
2.02
0.96
0.71
2.13
2.15
3.23
10.0 9.5 9.0 8.5 8.0 7.5 7.0 6.5 6.0 5.5 5.0 4.5 4.0 3.5 3.0 2.5 2.0 1.5 1.0 0.5 0.0
f1 (ppm)

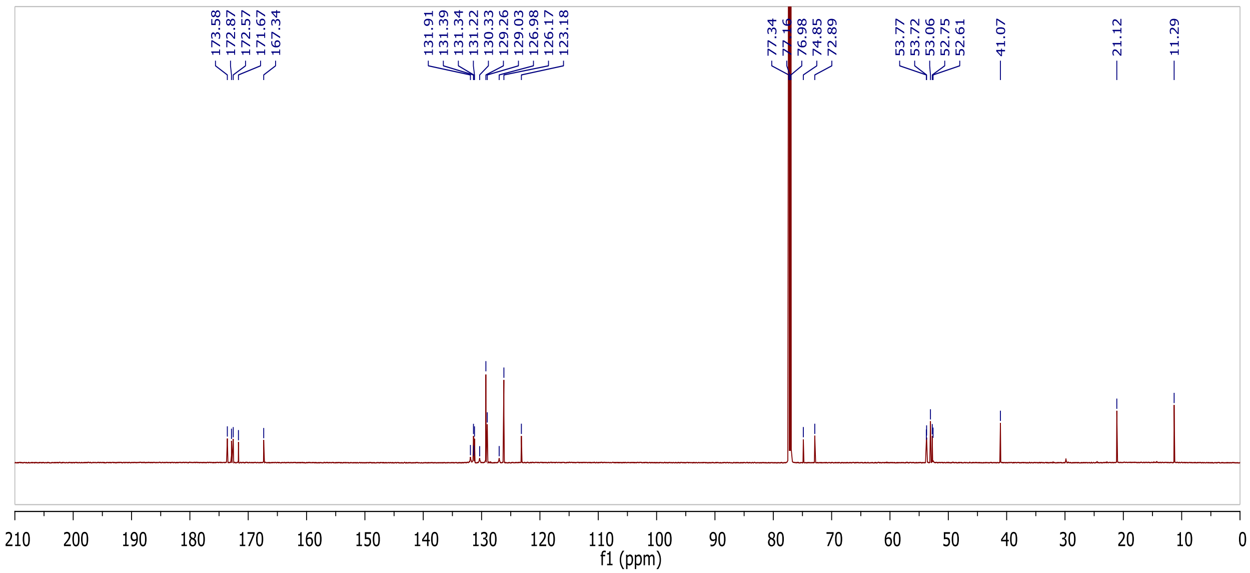
173.58
172.87
172.57
171.67
167.34
131.91
131.39
131.34
131.22
130.33
129.26
129.03
126.98
126.17
123.18
77.34
77.16
76.98
74.85
72.89
53.77
53.72
53.06
52.75
52.61
41.07
21.12
11.29
210 200 190 180 170 160 150 140 130 120 110 100 90 80 70 60 50 40 30 20 10 0
f1 (ppm)

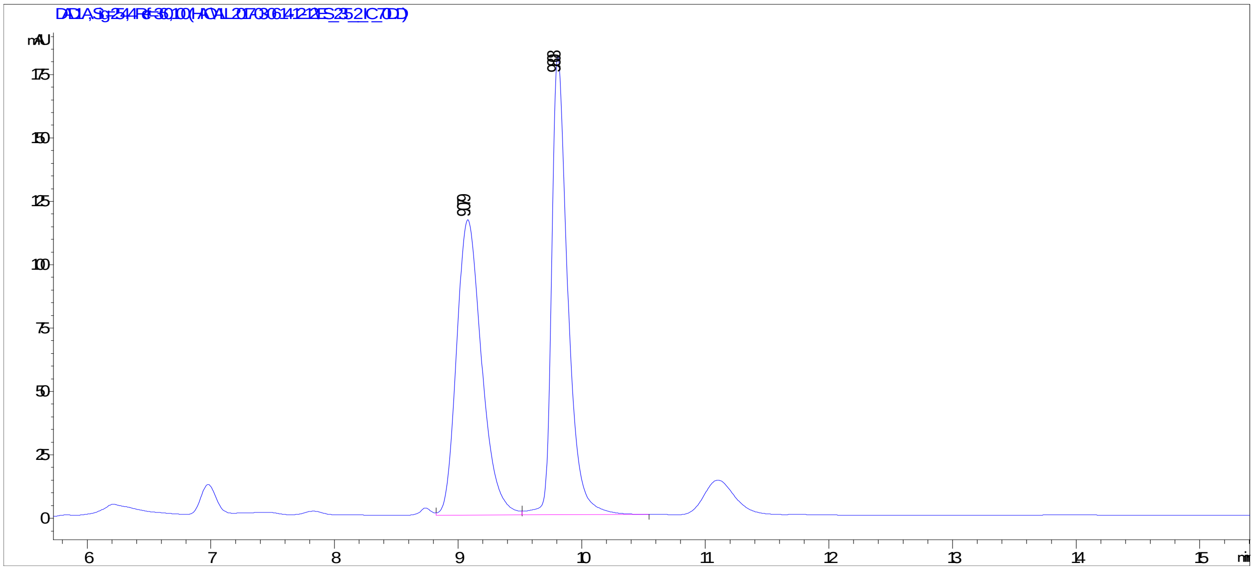

#	Time	Area	Height	Width	Area%	Symmetry
1	9.079	1647.7	116.5	0.217	49.825	0.75
2	9.808	1659.2	181.3	0.1387	50.175	0.584

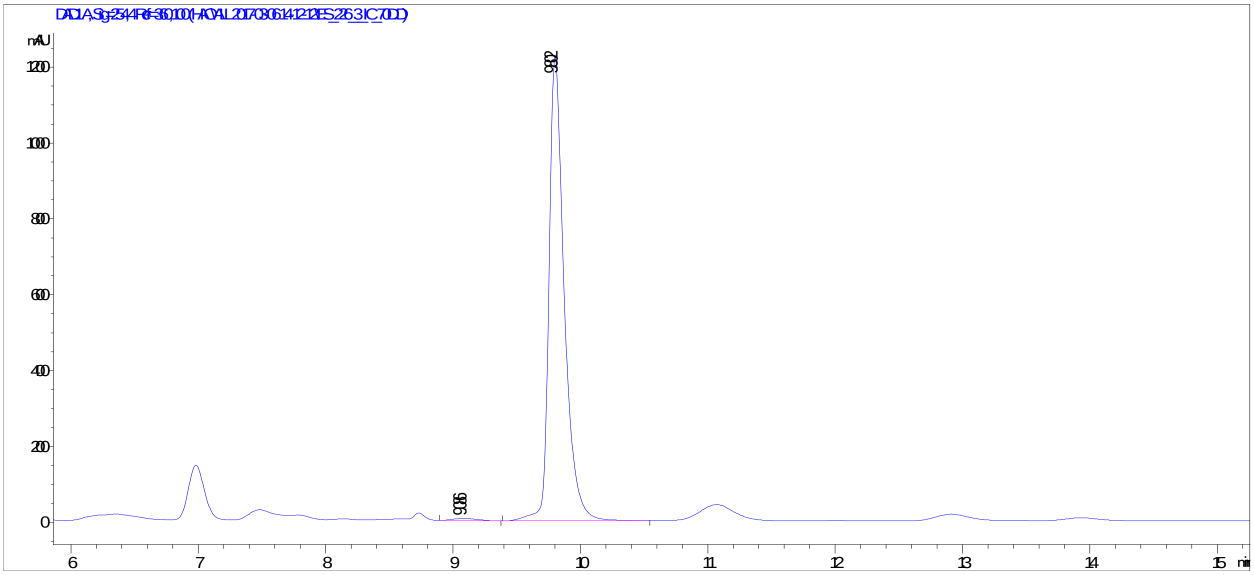

#	Time	Area	Height	Width	Area%	Symmetry
1	9.086	83	6.1	0.2163	0.809	0.809
2	9.802	10184.1	1225.7	0.1229	99.191	0.622

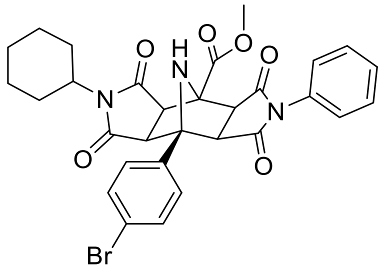
O
H
N
O
O
O
N
N
O
O
O
Br

5f

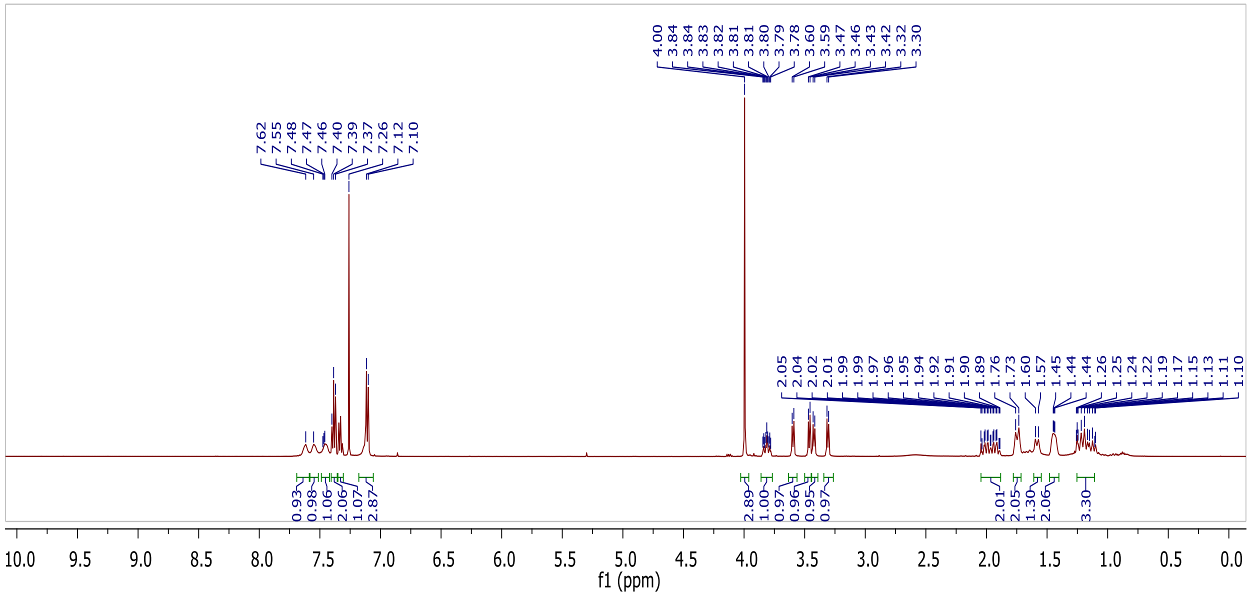
7.62
7.55
7.48
7.47
7.46
7.40
7.39
7.37
7.26
7.12
7.10
4.00
3.84
3.84
3.83
3.82
3.81
3.81
3.80
3.79
3.78
3.60
3.59
3.47
3.46
3.43
3.42
3.32
3.30
2.05
2.04
2.02
2.01
1.99
1.99
1.97
1.96
1.95
1.94
1.92
1.91
1.90
1.89
1.76
1.73
1.60
1.57
1.45
1.44
1.44
1.26
1.25
1.24
1.22
1.19
1.17
1.15
1.13
1.11
1.10
0.93
0.98
1.06
2.06
1.07
2.87
2.89
1.00
0.97
0.96
0.95
0.97
2.01
2.05
1.30
2.06
3.30
10.0
9.5
9.0
8.5
8.0
7.5
7.0
6.5
6.0
5.5
5.0
4.5
4.0
3.5
3.0
2.5
2.0
1.5
1.0
0.5
0.0
f1 (ppm)

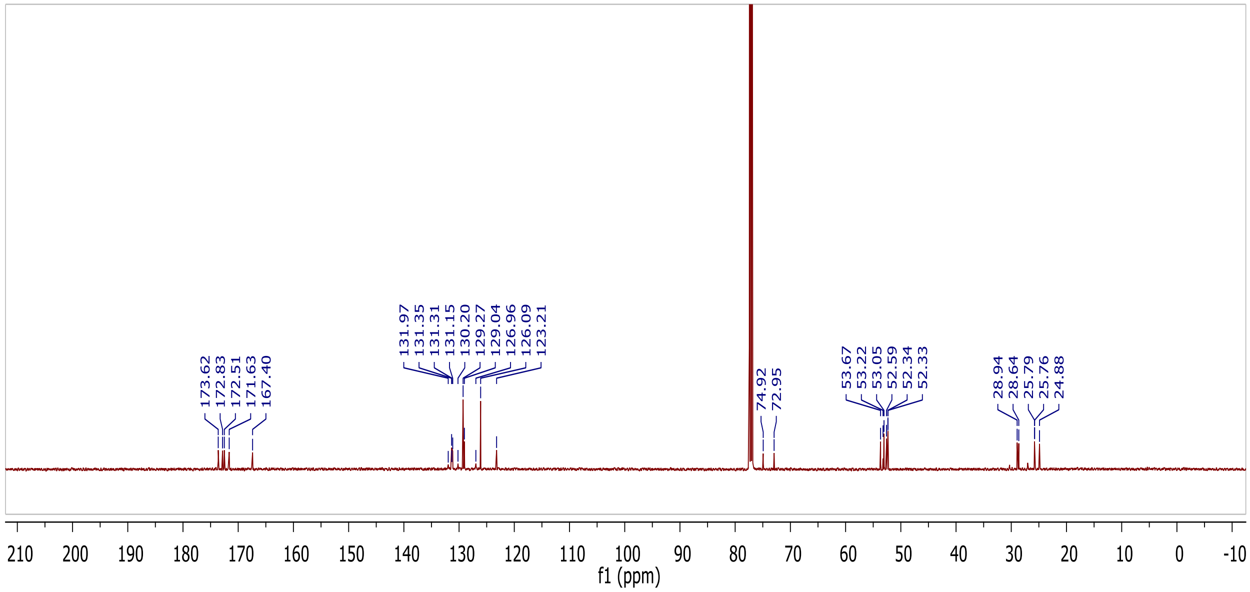
173.62
172.83
172.51
171.63
167.40
131.97
131.35
131.31
131.15
130.20
129.27
129.04
126.96
126.09
123.21
74.92
72.95
53.67
53.22
53.05
52.59
52.34
52.33
28.94
28.64
25.79
25.76
24.88
210
200
190
180
170
160
150
140
130
120
110
100
90
80
70
60
50
40
30
20
10
0
-10
f1 (ppm)

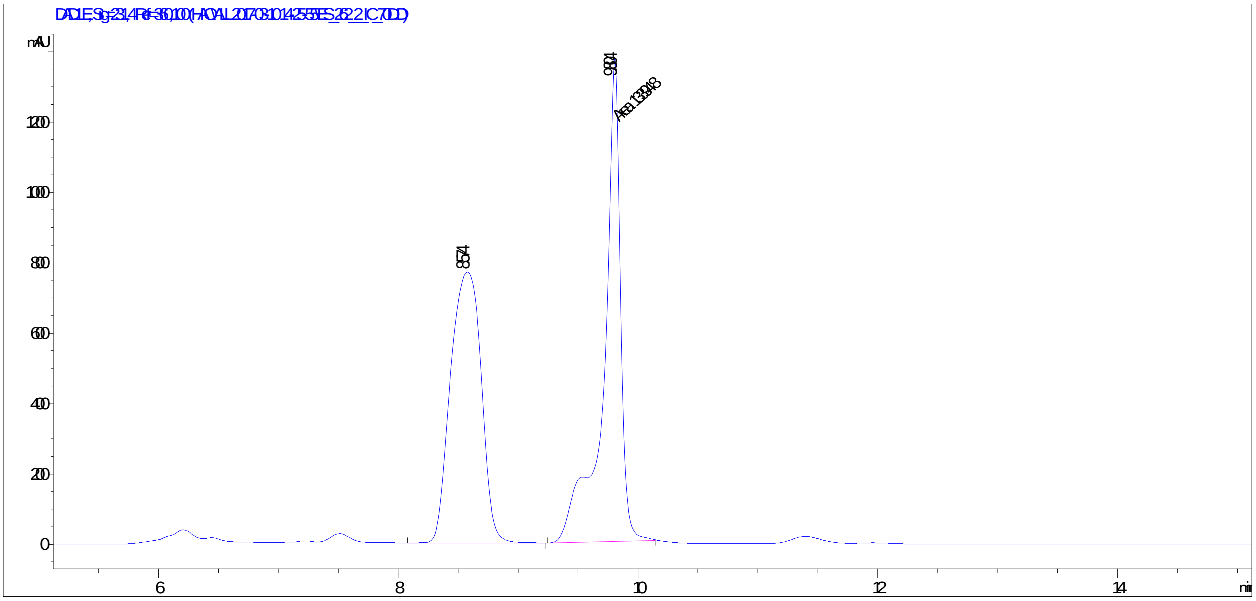

#	Time	Area	Height	Width	Area%	Symmetry
1	8.574	13705.7	769.5	0.2978	50.574	1.161
2	9.804	13394.8	1377.2	0.1621	49.426	1.893

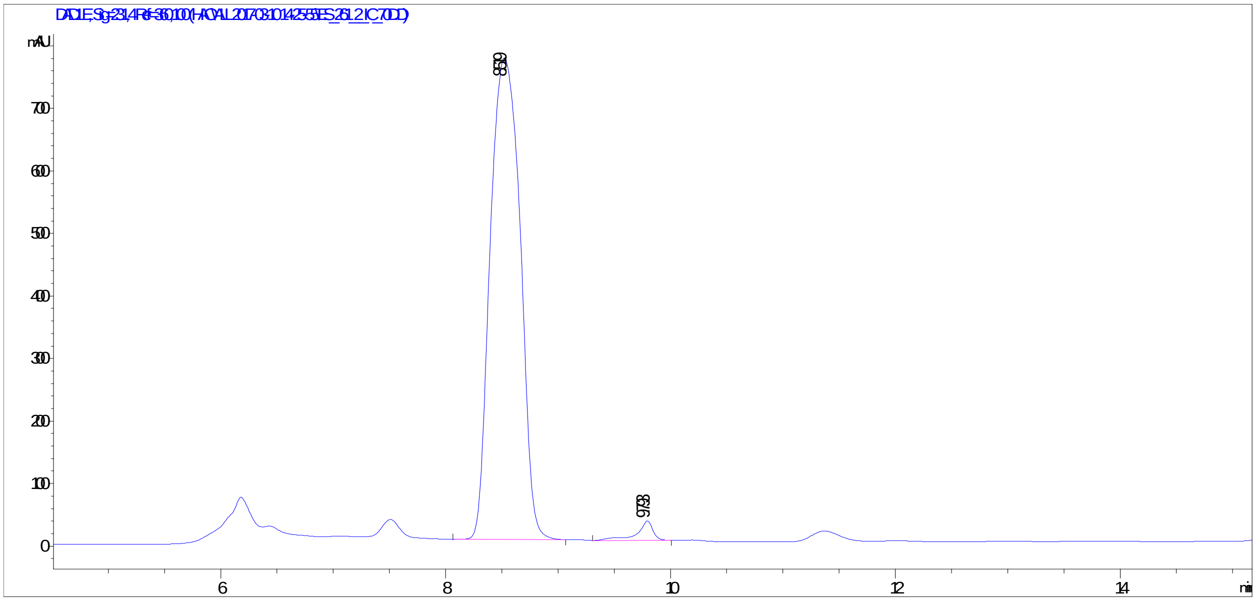

#	Time	Area	Height	Width	Area%	Symmetry
1	8.519	14246.4	770.3	0.3138	97.875	0.872
2	9.793	309.3	31.1	0.1381	2.125	2.068

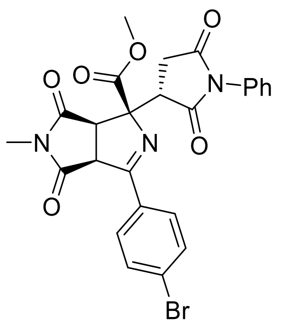

6a

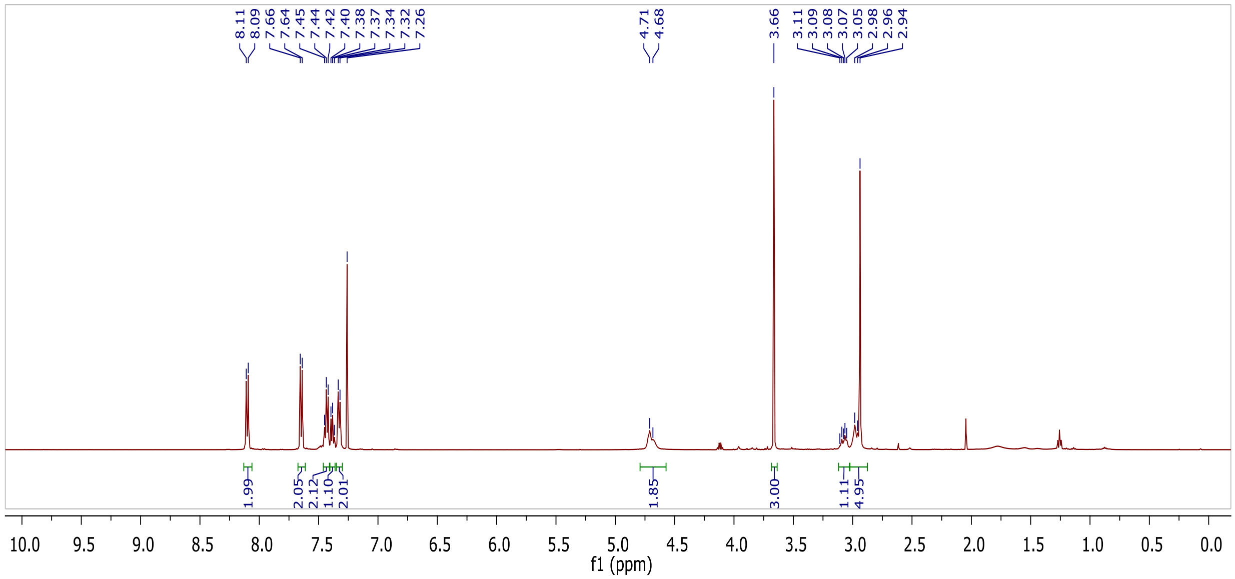
8.11
8.09
7.66
7.64
7.45
7.44
7.42
7.40
7.38
7.37
7.34
7.32
7.26
4.71
4.68
3.66
3.11
3.09
3.08
3.07
3.05
2.98
2.96
2.94
1.99
2.05
2.12
1.10
2.01
1.85
3.00
1.11
4.95
10.0 9.5 9.0 8.5 8.0 7.5 7.0 6.5 6.0 5.5 5.0 4.5 4.0 3.5 3.0 2.5 2.0 1.5 1.0 0.5 0.0
f1 (ppm)

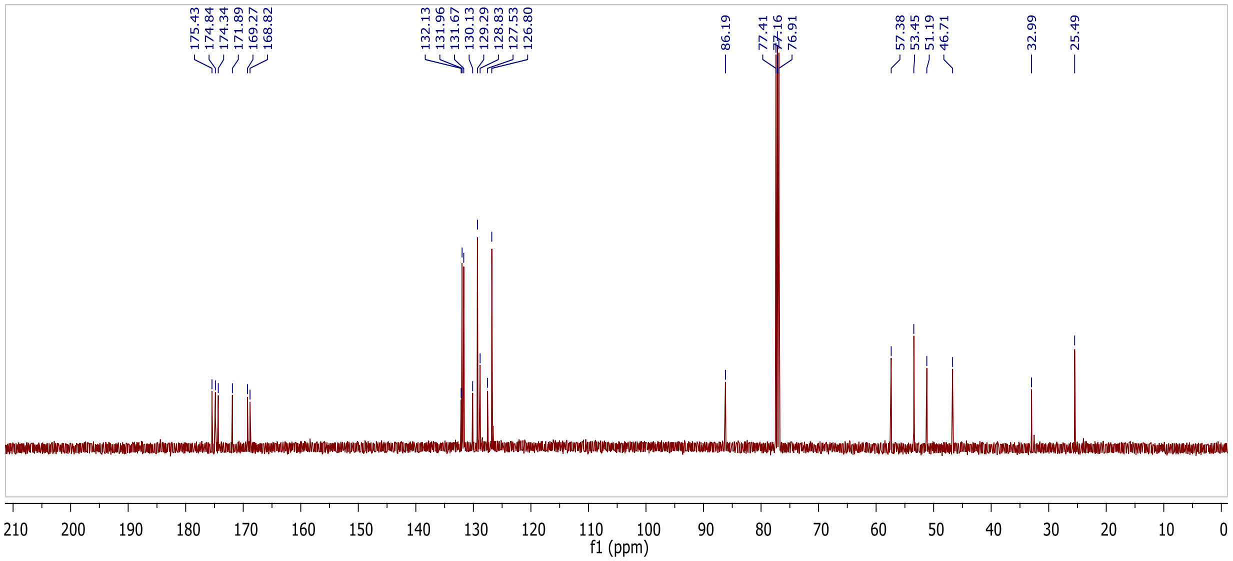
175.43
174.84
174.34
171.89
169.27
168.82
132.13
131.96
131.67
130.13
129.29
128.83
127.53
126.80
86.19
77.41
77.16
76.91
57.38
53.45
51.19
46.71
32.99
25.49
210 200 190 180 170 160 150 140 130 120 110 100 90 80 70 60 50 40 30 20 10 0
f1 (ppm)

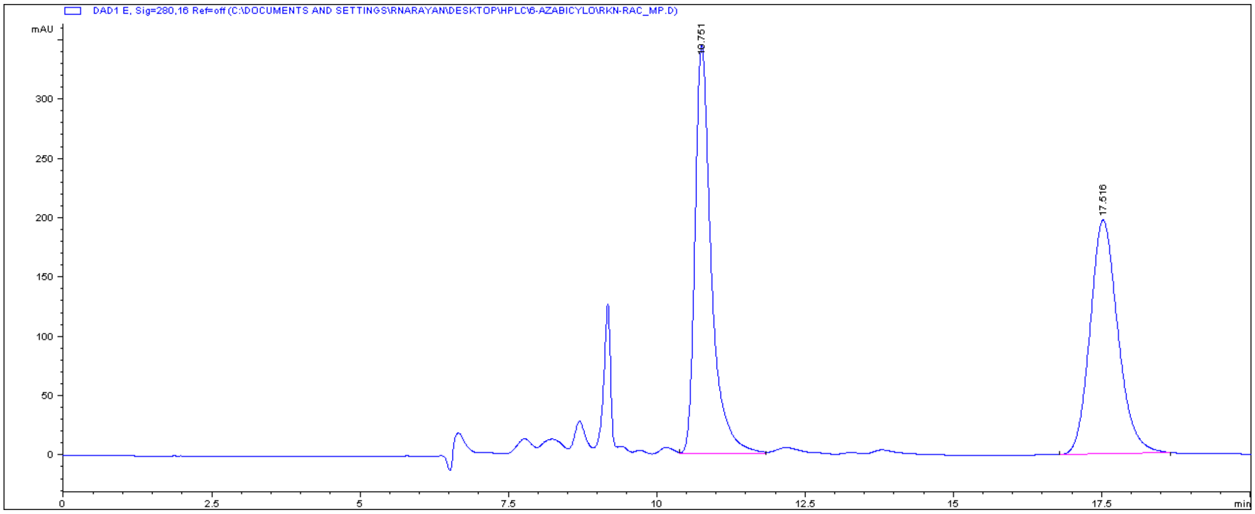

Signal 5: DAD1 E, Sig=280,16 Ref=off

Peak #	RetTime [min]	Type	Width [min]	Area [mAU*s]	Height [mAU]	Area %
1	10.751	VB	0.2829	6599.97266	345.40903	50.5561
2	17.516	BB	0.5017	6454.79004	197.28824	49.4439

Totals : 1.30548e4 542.69727

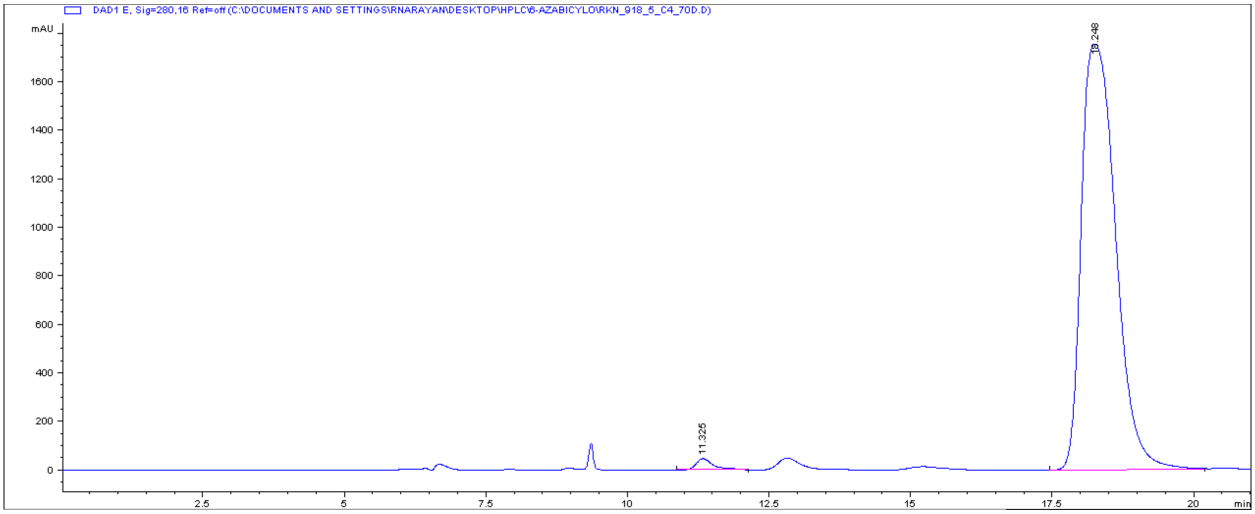

Signal 5: DAD1 E, Sig=280,16 Ref=off

Peak #	RetTime [min]	Type	Width [min]	Area [mAU*s]	Height [mAU]	Area %
1	11.325	BB	0.3270	1031.27820	46.46158	1.3703
2	18.248	BB	0.6648	7.42264e4	1757.16162	98.6297

Totals : 7.52577e4 1803.62320

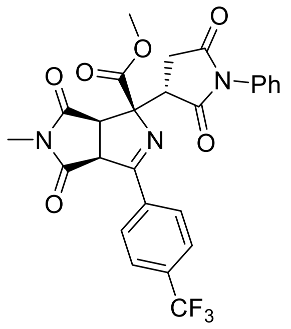

6b

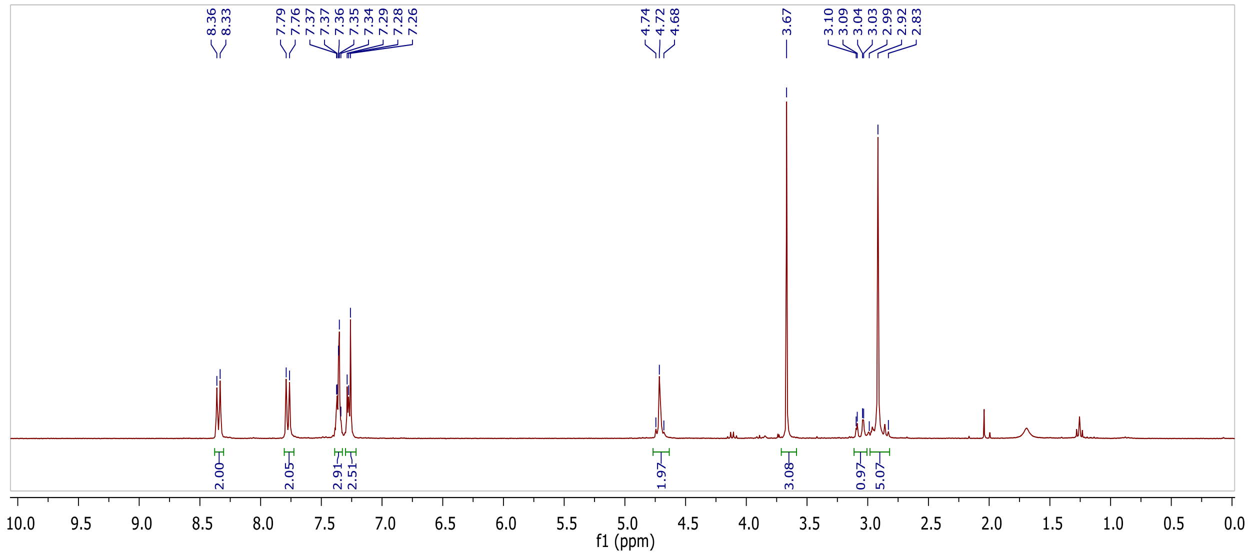
8.36
8.33
7.79
7.76
7.37
7.36
7.35
7.34
7.29
7.28
7.26
4.74
4.72
4.68
3.67
3.10
3.09
3.04
3.03
2.99
2.92
2.83
2.00
2.05
2.91
2.51
1.97
3.08
0.97
5.07
f1 (ppm)

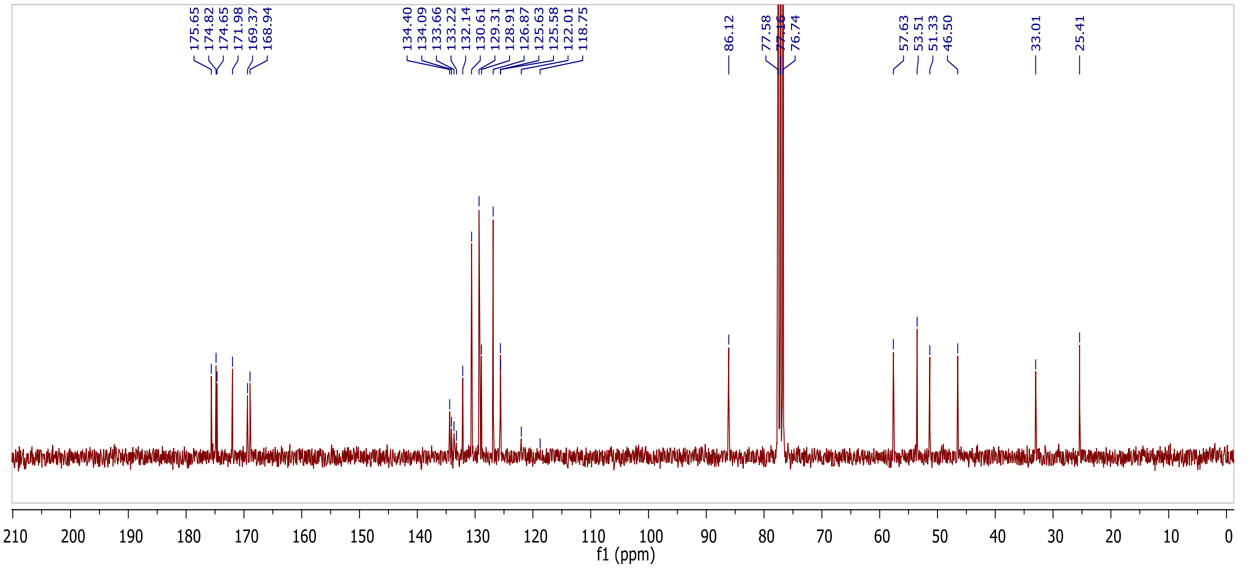
175.65
174.82
174.65
171.98
169.37
168.94
134.40
134.09
133.66
133.22
132.14
130.61
129.31
128.91
126.87
125.63
125.58
122.01
118.75
86.12
77.58
77.16
76.74
57.63
53.51
51.33
46.50
33.01
25.41
f1 (ppm)

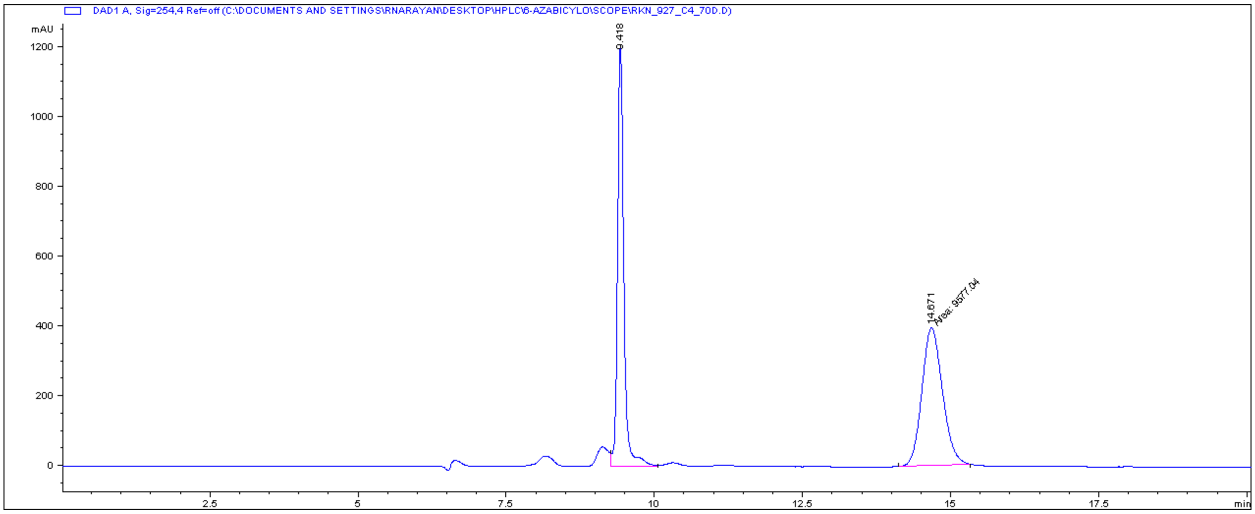

Signal 1: DAD1 A, Sig=254,4 Ref=off

Peak #	RetTime [min]	Type	Width [min]	Area [mAU*s]	Height [mAU]	Area %
1	9.418	VB	0.1098	8917.16309	1212.69092	48.2160
2	14.671	MM	0.4040	9577.03906	395.09808	51.7840

Totals : 1.84942e4 1607.78900

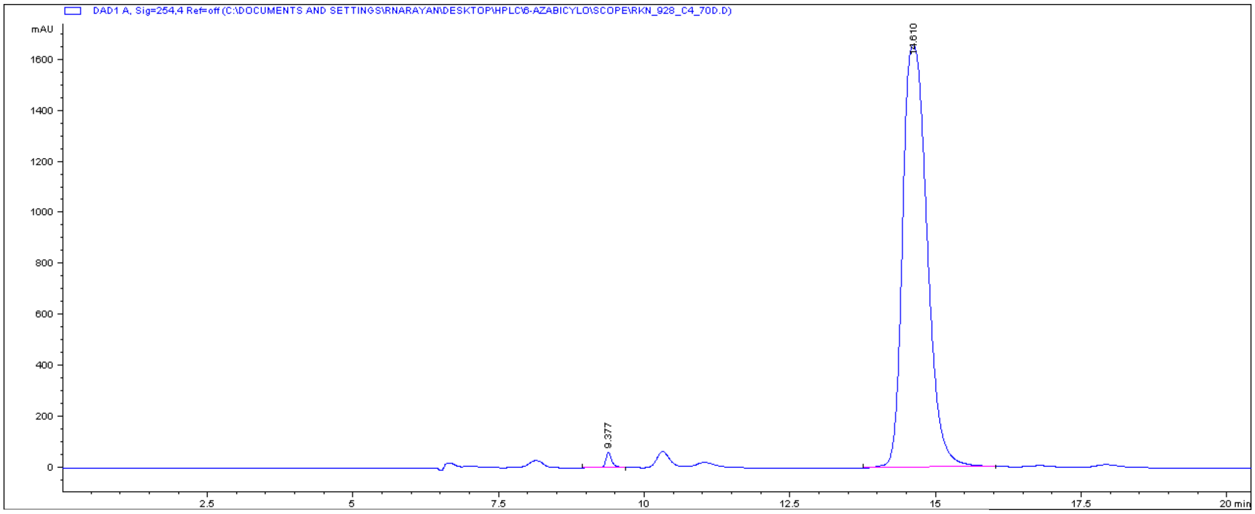

Signal 1: DAD1 A, Sig=254,4 Ref=off

Peak #	RetTime [min]	Type	Width [min]	Area [mAU*s]	Height [mAU]	Area %
1	9.377	BB	0.1122	476.96997	61.69894	0.9796
2	14.610	BB	0.4553	4.82132e4	1657.55554	99.0204

Totals : 4.86902e4 1719.25448

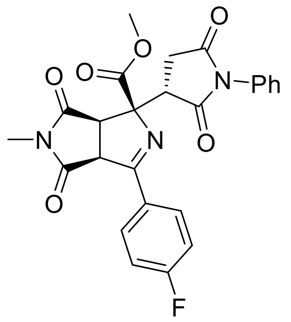
O
O
O
N
Ph
O
N
N
O
O
F

6c

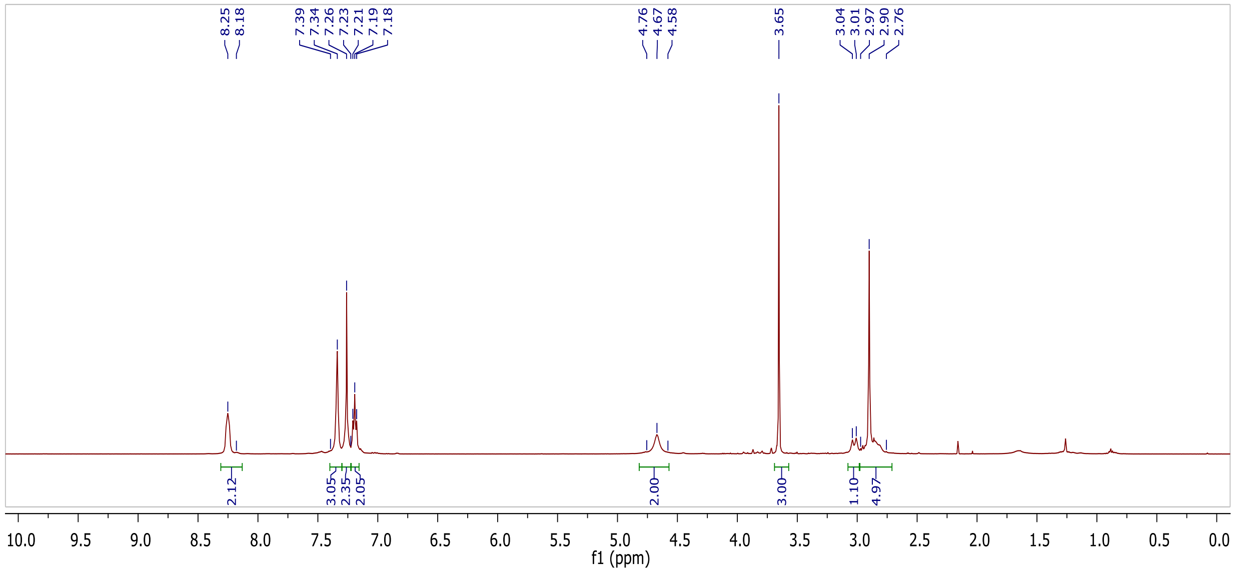
8.25
8.18
7.39
7.34
7.26
7.23
7.21
7.19
7.18
4.76
4.67
4.58
3.65
3.04
3.01
2.97
2.90
2.76
2.12
3.05
2.35
2.05
2.00
3.00
1.10
4.97
10.0 9.5 9.0 8.5 8.0 7.5 7.0 6.5 6.0 5.5 5.0 4.5 4.0 3.5 3.0 2.5 2.0 1.5 1.0 0.5 0.0
f1 (ppm)

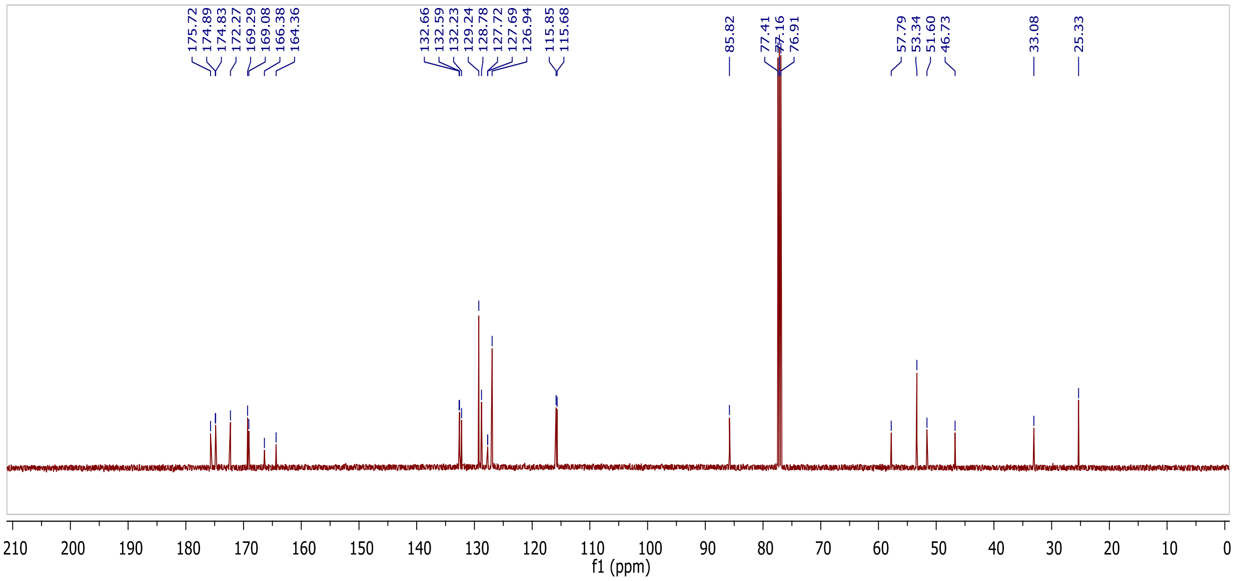
175.72
174.89
174.83
172.27
169.29
169.08
166.38
164.36
132.66
132.59
132.23
129.24
128.78
127.72
127.69
126.94
115.85
115.68
85.82
77.41
77.16
76.91
57.79
53.34
51.60
46.73
33.08
25.33
210 200 190 180 170 160 150 140 130 120 110 100 90 80 70 60 50 40 30 20 10 0
f1 (ppm)

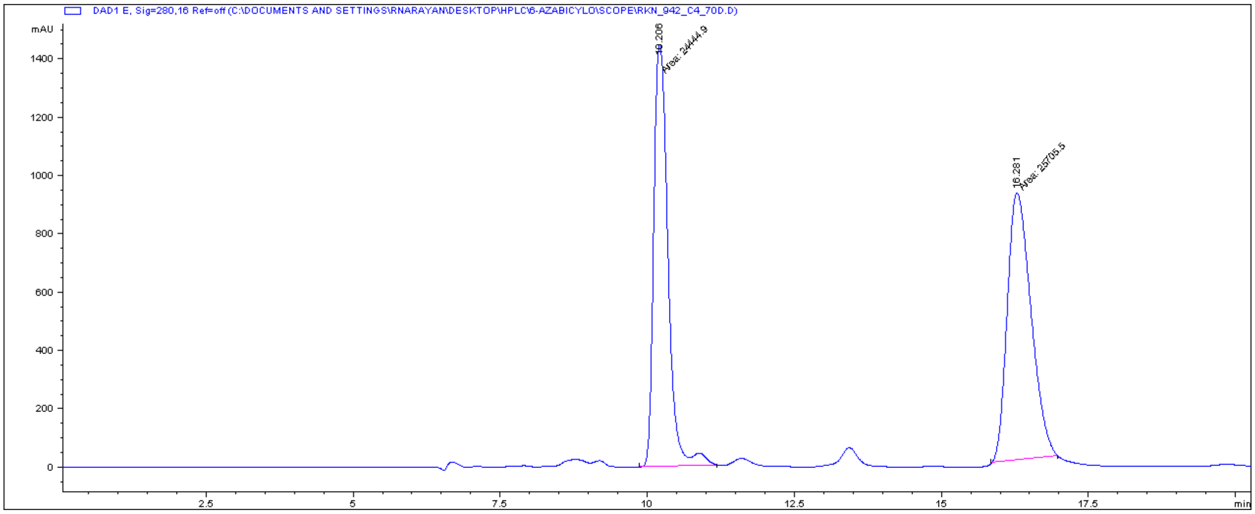

Signal 5: DAD1 E, Sig=280,16 Ref=off

Peak #	RetTime [min]	Type	Width [min]	Area [mAU*s]	Height [mAU]	Area %
1	10.206	MM	0.2816	2.44449e4	1446.95764	48.7432
2	16.281	MM	0.4674	2.57055e4	916.69397	51.2568

Totals : 5.01504e4 2363.65161

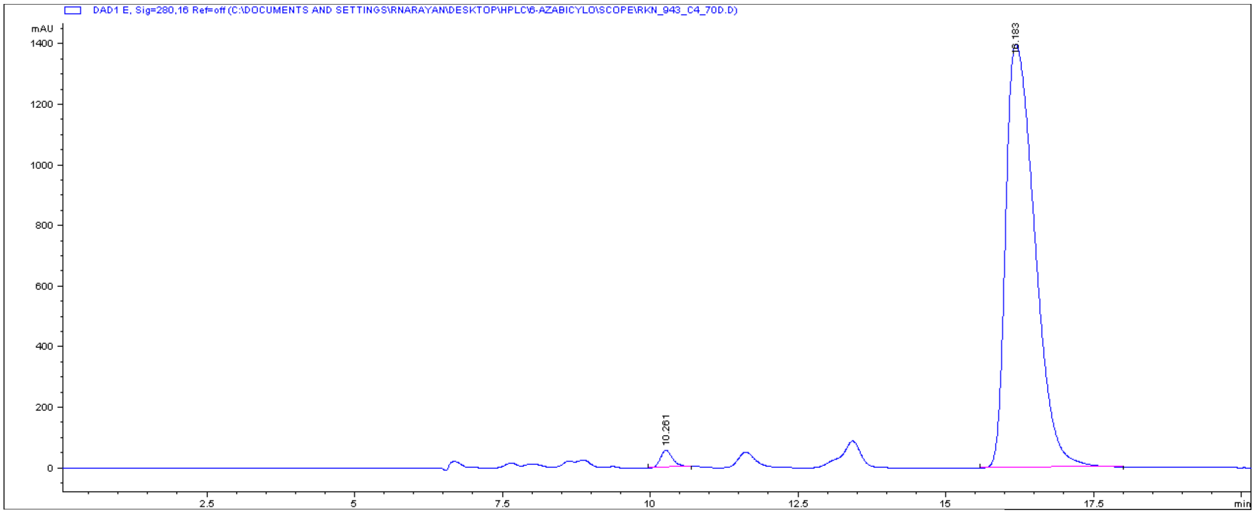

Signal 5: DAD1 E, Sig=280,16 Ref=off

Peak #	RetTime [min]	Type	Width [min]	Area [mAU*s]	Height [mAU]	Area %
1	10.261	BB	0.2223	822.65491	56.36641	1.6942
2	16.183	BB	0.5362	4.77348e4	1398.62500	98.3058

Totals : 4.85574e4 1454.99141

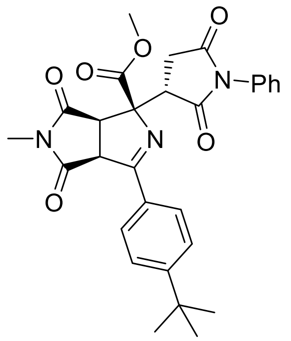

6d

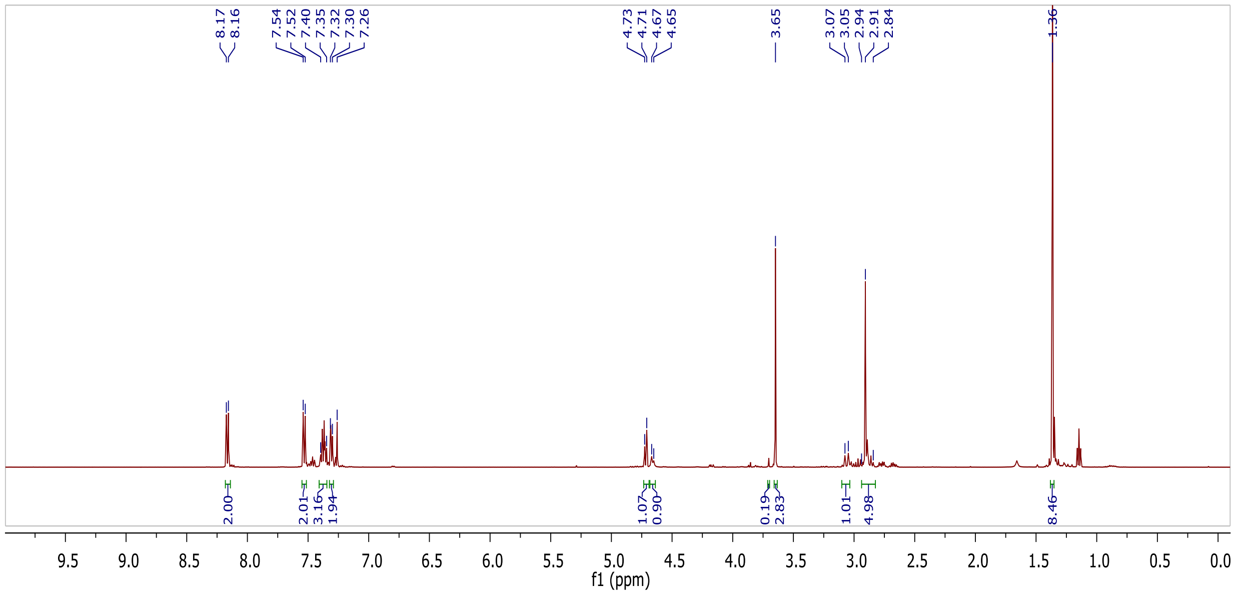
8.17
8.16
7.54
7.52
7.40
7.35
7.32
7.30
7.26
4.73
4.71
4.67
4.65
3.65
3.07
3.05
2.94
2.91
2.84
1.36
2.00
2.01
3.16
1.94
1.07
0.90
0.19
2.83
1.01
4.98
8.46
9.5 9.0 8.5 8.0 7.5 7.0 6.5 6.0 5.5 5.0 4.5 4.0 3.5 3.0 2.5 2.0 1.5 1.0 0.5 0.0
f1 (ppm)

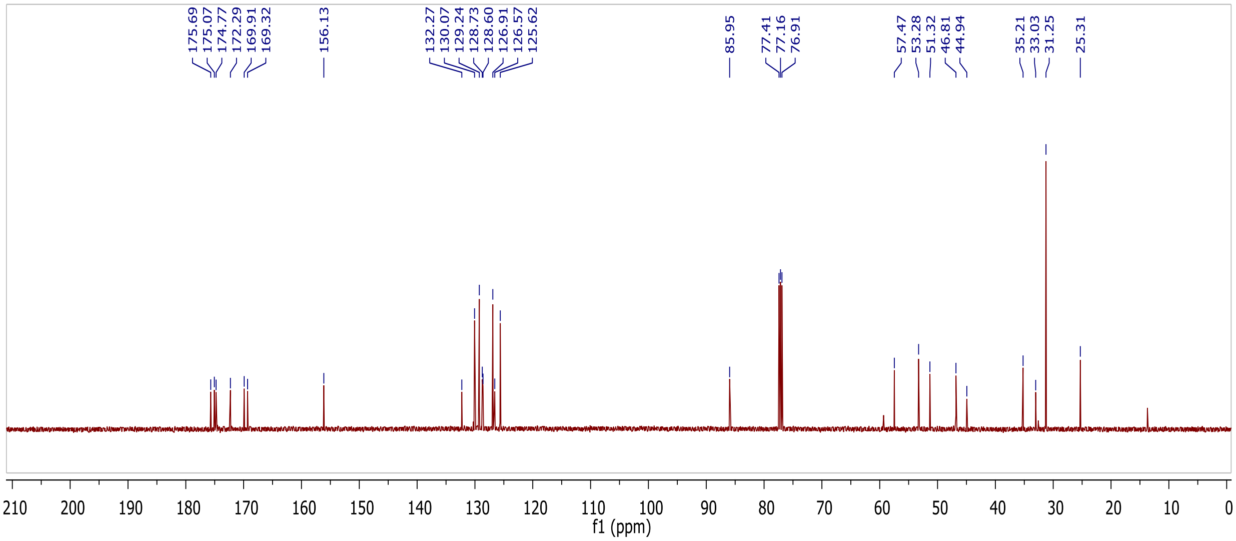
175.69
175.07
174.77
172.29
169.91
169.32
156.13
132.27
130.07
129.24
128.73
128.60
126.91
126.57
125.62
85.95
77.41
77.16
76.91
57.47
53.28
51.32
46.81
44.94
35.21
33.03
31.25
25.31
210 200 190 180 170 160 150 140 130 120 110 100 90 80 70 60 50 40 30 20 10 0
f1 (ppm)

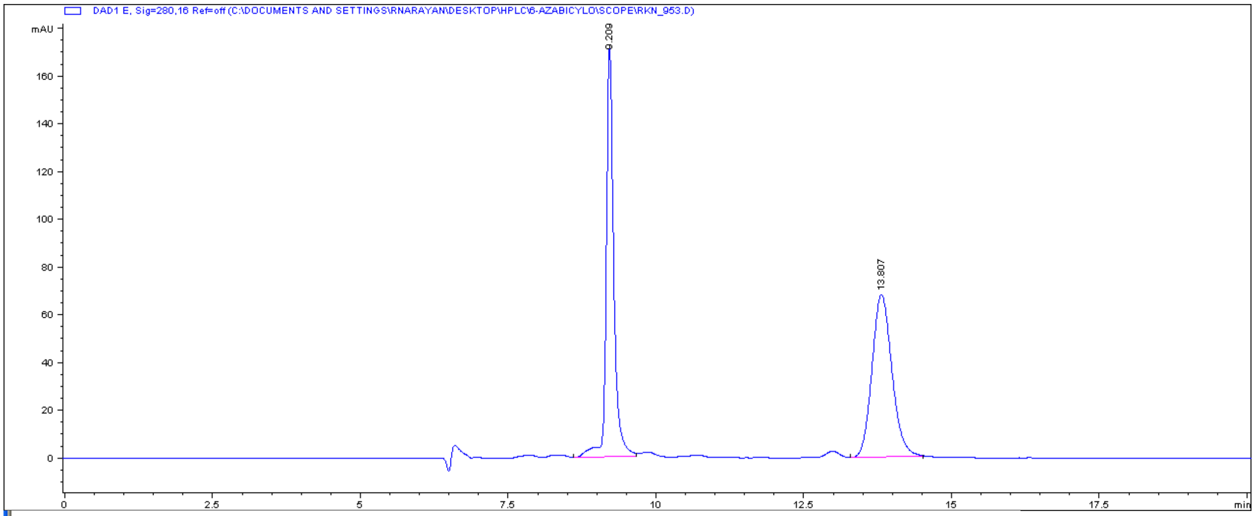

Signal 5: DAD1 E, Sig=280,16 Ref=off

Peak #	RetTime [min]	Type	Width [min]	Area [mAU*s]	Height [mAU]	Area %
1	9.209	VB	0.1316	1536.71521	173.13629	48.8932
2	13.807	VB	0.3648	1606.28723	68.20111	51.1068

Totals : 3143.00244 241.33740

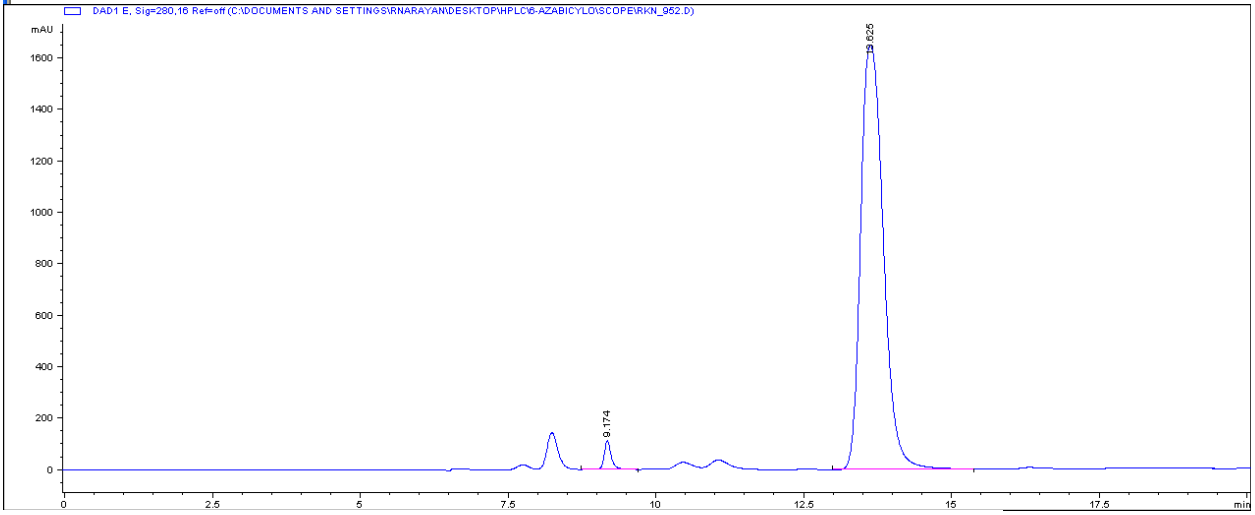

Signal 5: DAD1 E, Sig=280,16 Ref=off

Peak #	RetTime [min]	Type	Width [min]	Area [mAU*s]	Height [mAU]	Area %
1	9.174	BB	0.1332	1018.50854	112.94594	2.2581
2	13.625	VB	0.4215	4.40855e4	1651.11938	97.7419

Totals : 4.51040e4 1764.06532

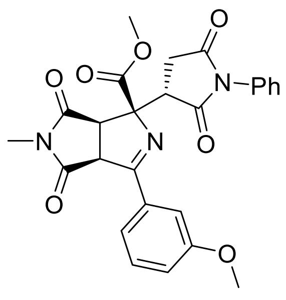
O
O
N
Ph
O
O
O
O
N
N
O
O

6e

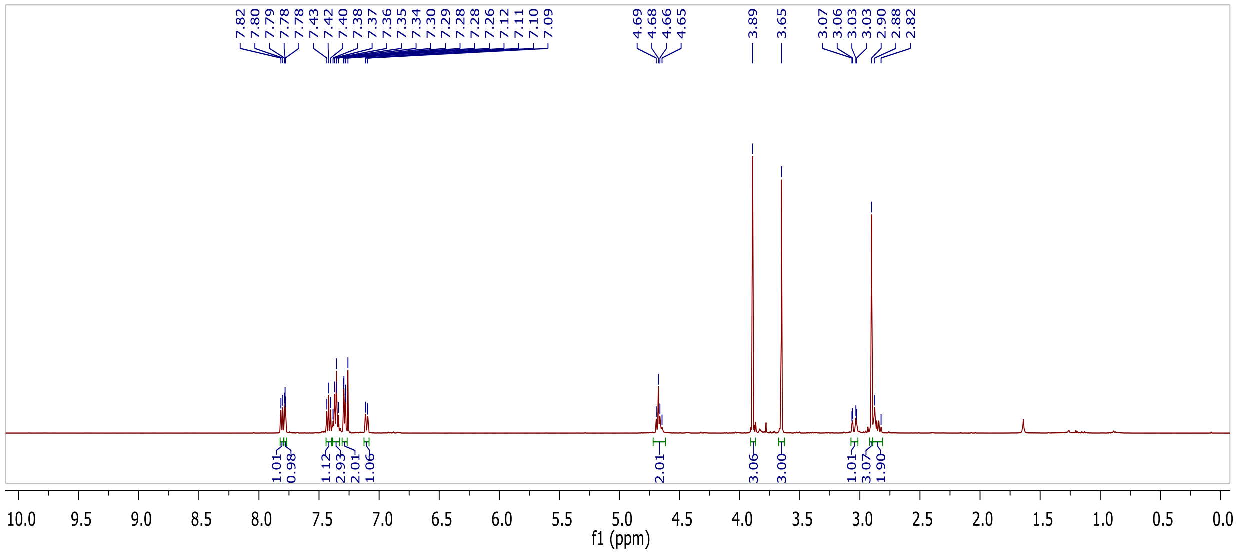
7.82
7.80
7.79
7.78
7.43
7.42
7.40
7.38
7.37
7.36
7.35
7.34
7.30
7.29
7.28
7.28
7.26
7.12
7.11
7.10
7.09
4.69
4.68
4.66
4.65
3.89
3.65
3.07
3.06
3.03
3.03
2.90
2.88
2.82
1.01
0.98
1.12
2.93
2.01
1.06
2.01
3.06
3.00
1.01
3.07
1.90
10.0
9.5
9.0
8.5
8.0
7.5
7.0
6.5
6.0
5.5
5.0
4.5
4.0
3.5
3.0
2.5
2.0
1.5
1.0
0.5
0.0
f1 (ppm)

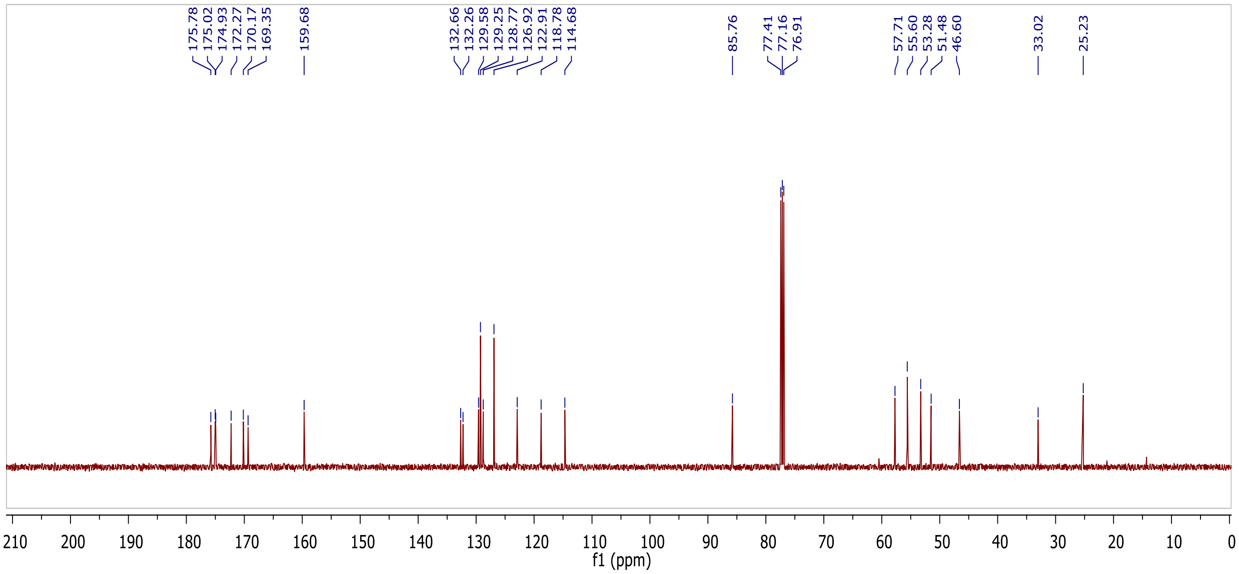
175.78
175.02
174.93
172.27
170.17
169.35
159.68
132.66
132.26
129.58
129.25
128.77
126.92
122.91
118.78
114.68
85.76
77.41
77.16
76.91
57.71
55.60
53.28
51.48
46.60
33.02
25.23
210
200
190
180
170
160
150
140
130
120
110
100
90
80
70
60
50
40
30
20
10
0
f1 (ppm)

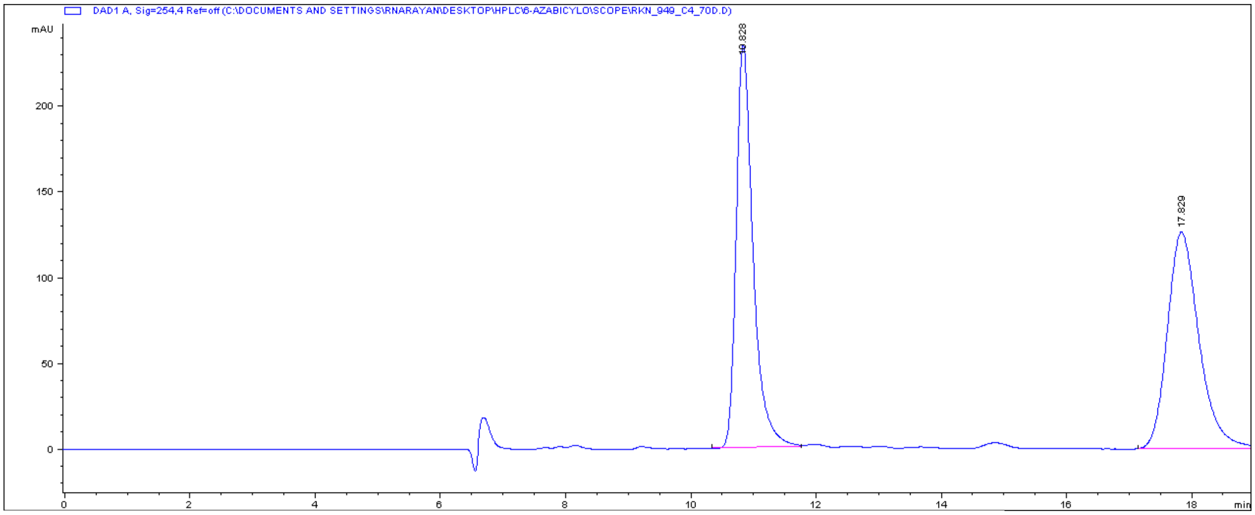

Signal 1: DAD1 A, Sig=254,4 Ref=off

Peak #	RetTime [min]	Type	Width [min]	Area [mAU*s]	Height [mAU]	Area %
1	10.828	BB	0.2814	4428.05273	235.44859	49.8356
2	17.829	BBA	0.5303	4457.27441	126.74397	50.1644

Totals : 8885.32715 362.19257

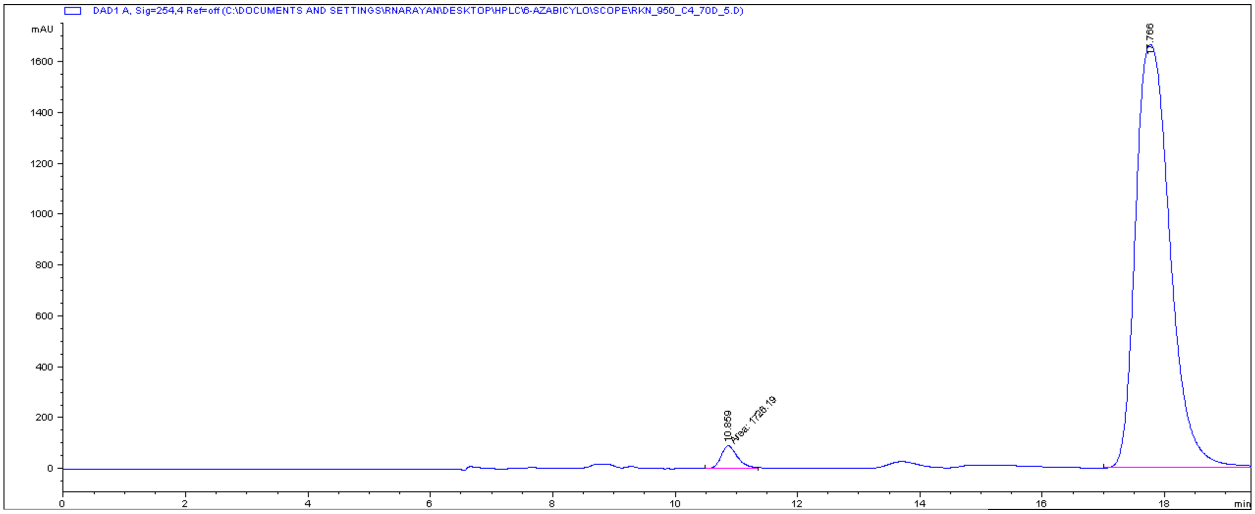

Signal 1: DAD1 A, Sig=254,4 Ref=off

Peak #	RetTime [min]	Type	Width [min]	Area [mAU*s]	Height [mAU]	Area %
1	10.859	MM	0.3204	1726.18738	89.79773	2.5783
2	17.766	BB	0.6089	6.52254e4	1665.47998	97.4217

Totals : 6.69516e4 1755.27771

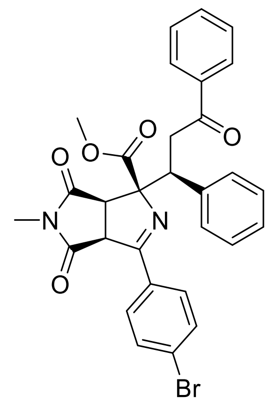
O
O
O
N
N
O
O
Br

7a

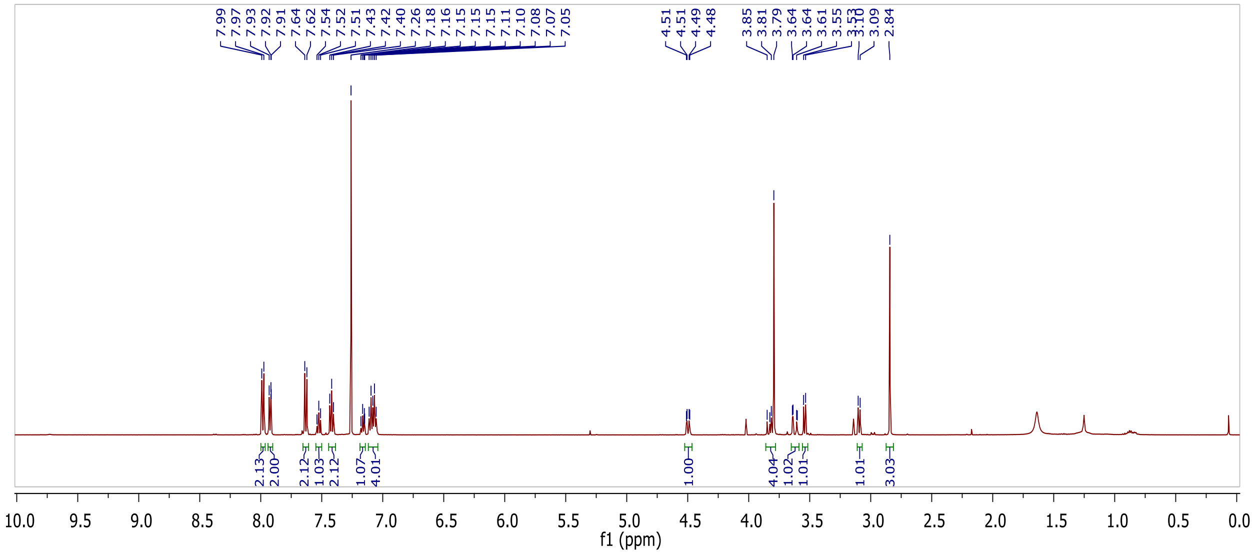
7.99
7.97
7.93
7.92
7.91
7.64
7.62
7.54
7.52
7.51
7.43
7.42
7.40
7.26
7.18
7.16
7.15
7.15
7.15
7.11
7.10
7.08
7.07
7.05
4.51
4.51
4.49
4.48
3.85
3.81
3.79
3.64
3.64
3.61
3.55
3.53
3.10
3.09
2.84
2.13
2.00
2.12
1.03
2.12
1.07
4.01
1.00
4.04
1.02
1.01
1.01
3.03
10.0 9.5 9.0 8.5 8.0 7.5 7.0 6.5 6.0 5.5 5.0 4.5 4.0 3.5 3.0 2.5 2.0 1.5 1.0 0.5 0.0
f1 (ppm)

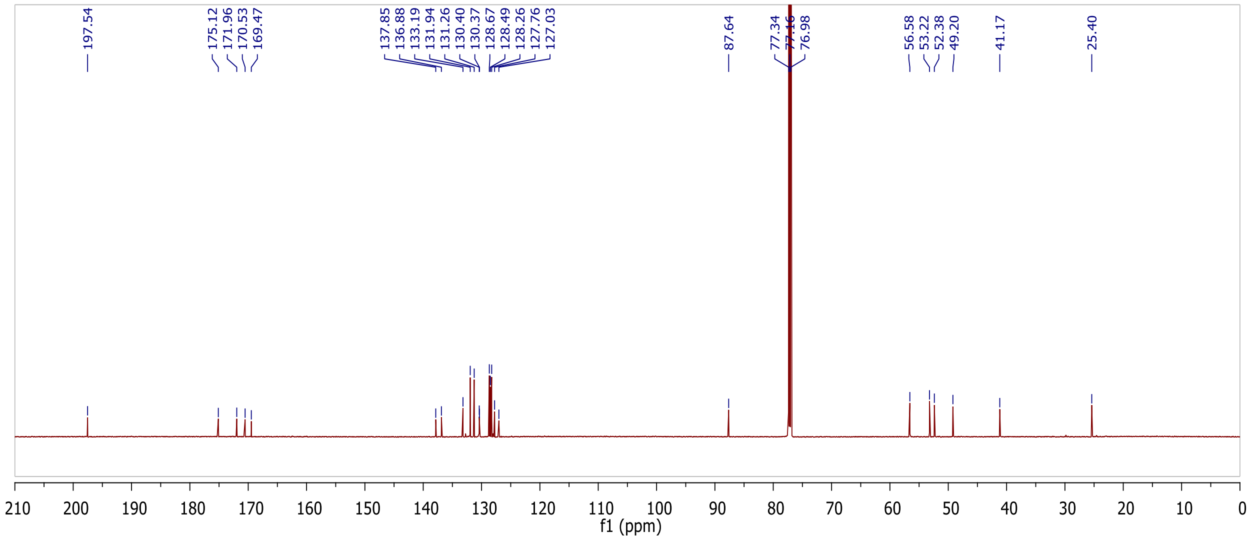
197.54
175.12
171.96
170.53
169.47
137.85
136.88
133.19
131.94
131.26
130.40
130.37
128.67
128.49
128.26
127.76
127.03
87.64
77.34
77.16
76.98
56.58
53.22
52.38
49.20
41.17
25.40
210 200 190 180 170 160 150 140 130 120 110 100 90 80 70 60 50 40 30 20 10 0
f1 (ppm)

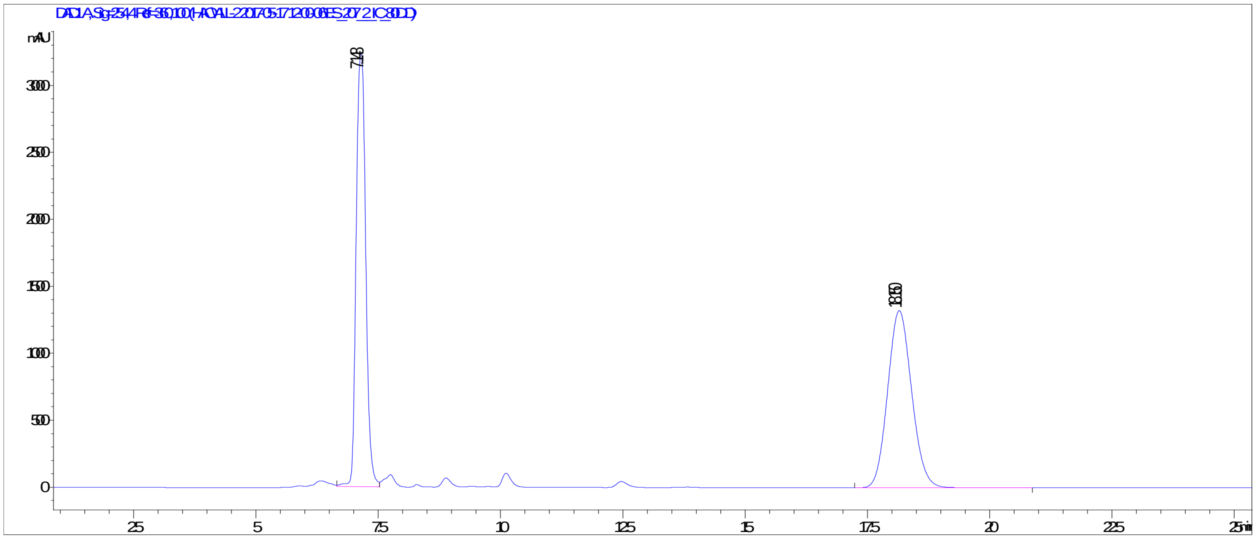

#	Time	Area	Height	Width	Area%	Symmetry
1	7.148	43965.8	3245	0.2161	49.361	0.975
2	18.15	45104.9	1322.6	0.5279	50.639	0.888

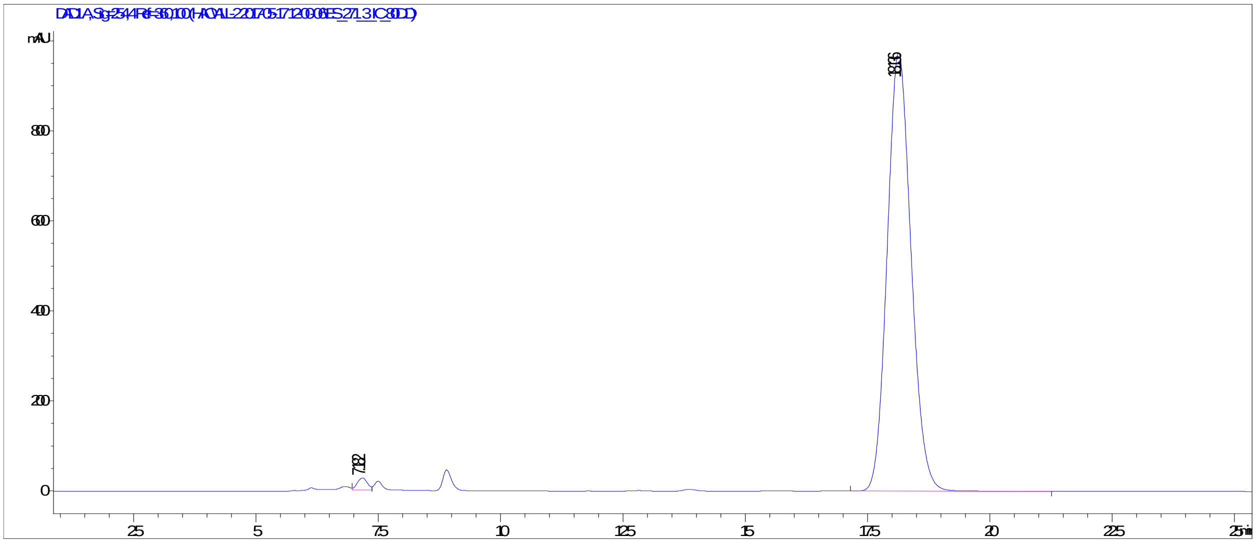

#	Time	Area	Height	Width	Area%	Symmetry
1	7.182	373.6	26.3	0.2259	1.118	1.107
2	18.136	33036.6	974.4	0.5256	98.882	0.871

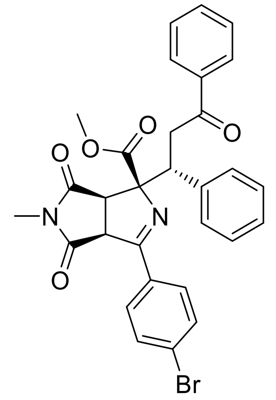
O
O
O
N
N
O
O
Br

7a-epi

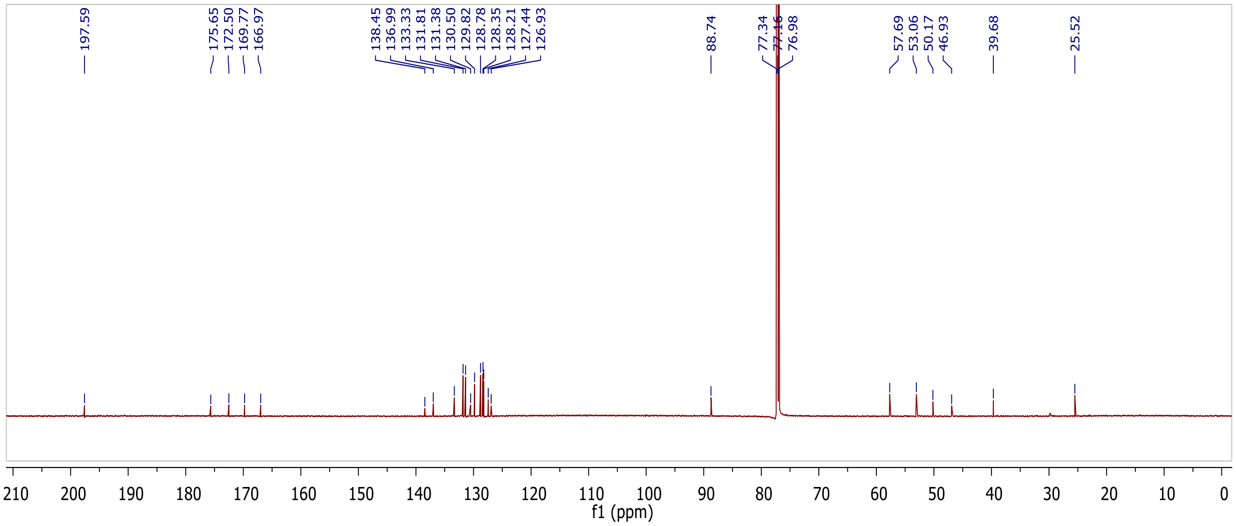
197.59
175.65
172.50
169.77
166.97
138.45
136.99
133.33
131.81
131.38
130.50
129.82
128.78
128.35
128.21
127.44
126.93
88.74
77.34
77.16
76.98
57.69
53.06
50.17
46.93
39.68
25.52
210 200 190 180 170 160 150 140 130 120 110 100 90 80 70 60 50 40 30 20 10 0
f1 (ppm)

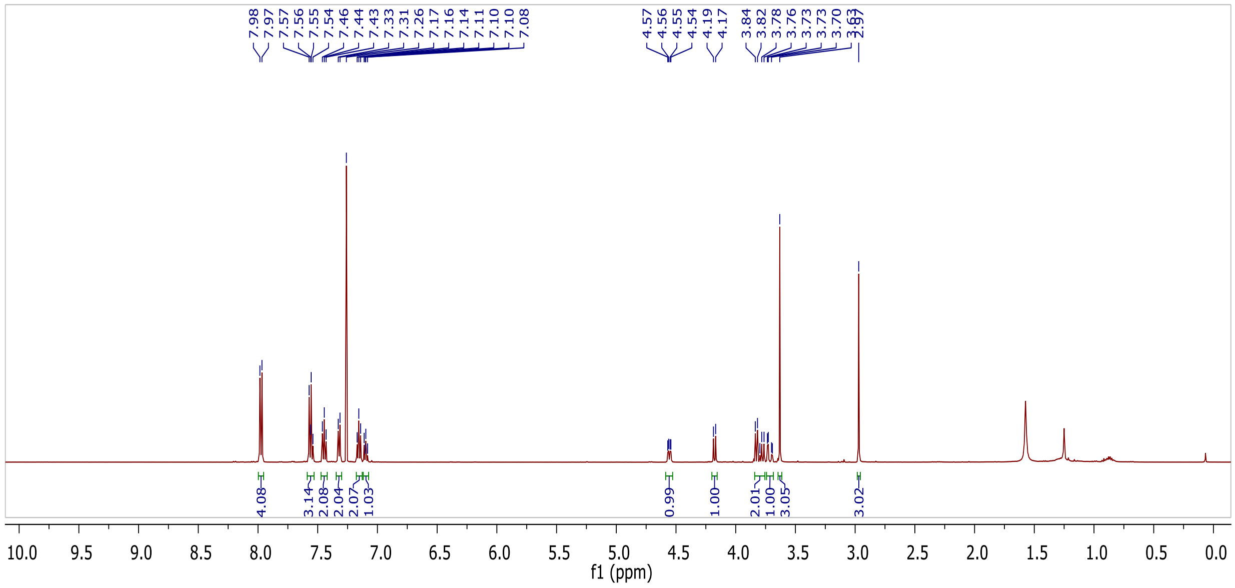
7.98
7.97
7.57
7.56
7.55
7.54
7.46
7.44
7.43
7.33
7.31
7.26
7.17
7.16
7.14
7.11
7.10
7.08
4.57
4.56
4.55
4.54
4.19
4.17
3.84
3.82
3.78
3.76
3.73
3.73
3.70
3.63
2.97
4.08
3.14
2.08
2.04
2.07
1.03
0.99
1.00
2.01
1.00
3.05
3.02
10.0 9.5 9.0 8.5 8.0 7.5 7.0 6.5 6.0 5.5 5.0 4.5 4.0 3.5 3.0 2.5 2.0 1.5 1.0 0.5 0.0
f1 (ppm)

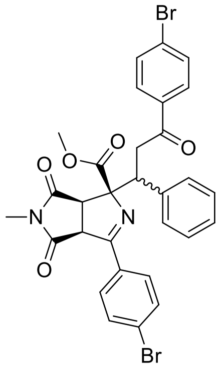
Br
O
O
O
O
N
N
O
Br

7b

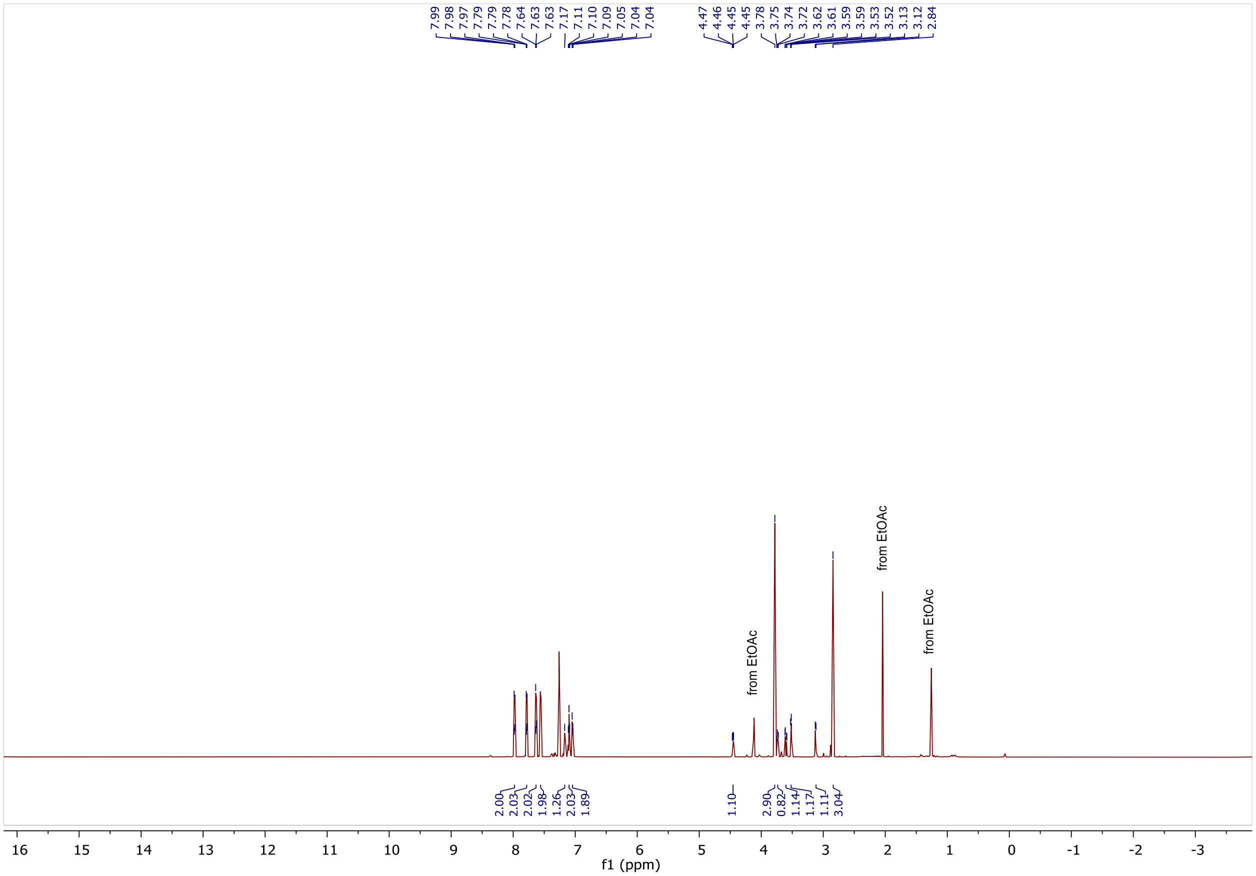
7.99
7.98
7.97
7.79
7.79
7.78
7.64
7.63
7.63
7.17
7.11
7.10
7.09
7.05
7.04
7.04
4.47
4.46
4.45
4.45
3.78
3.75
3.74
3.72
3.62
3.61
3.59
3.53
3.52
3.13
3.12
2.84
from EtOAc
from EtOAc
from EtOAc
2.00
2.03
2.02
1.98
1.26
2.03
1.89
1.10
2.90
0.82
1.14
1.17
1.11
3.04
f1 (ppm)

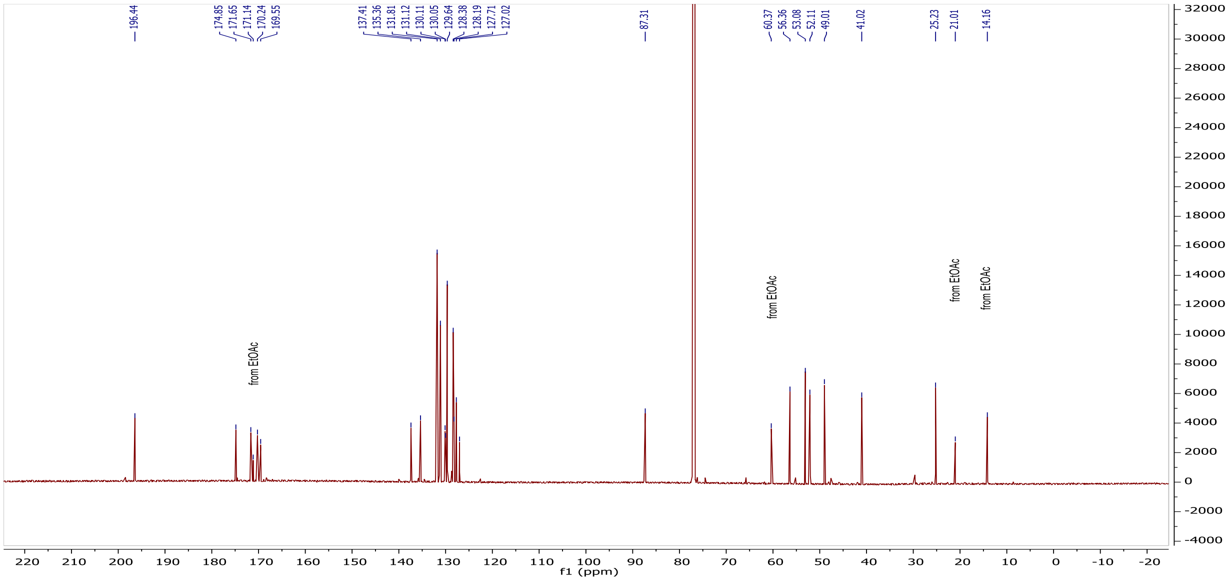

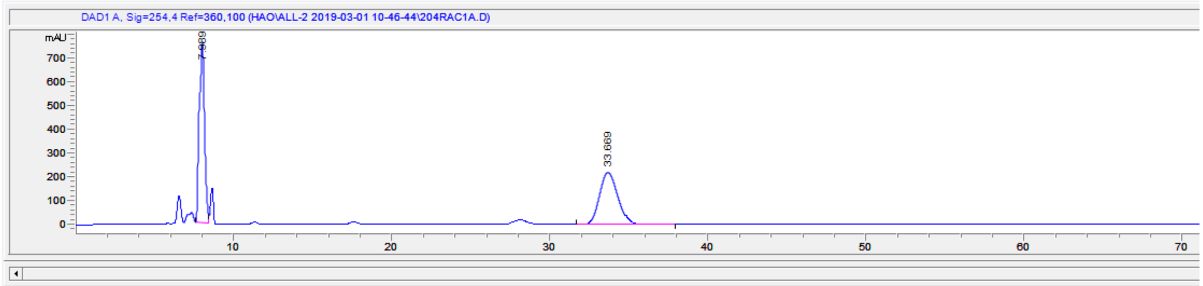

#	Time	Area	Height	Width	Area%	Symmetry
1	7.989	17741	764.1	0.3149	50.275	1.325
2	33.669	17547	219.8	1.2535	49.725	0.832

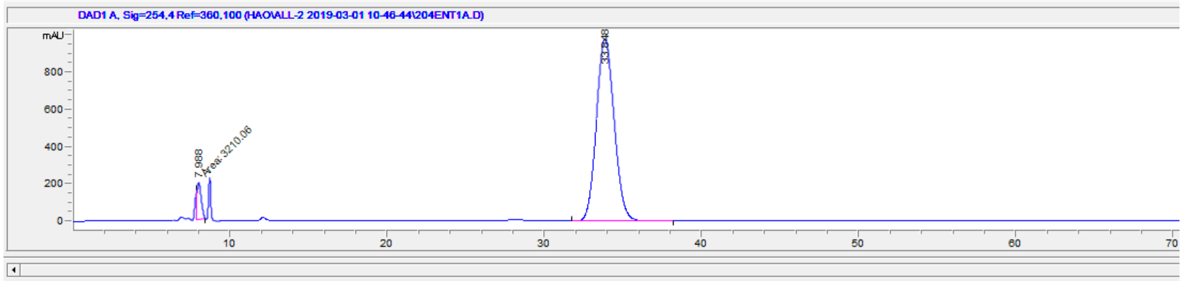

#	Time	Area	Height	Width	Area%	Symmetry
1	7.988	3210.1	201.3	0.2658	3.968	0.484
2	33.848	77690.6	977	1.2397	96.032	0.899

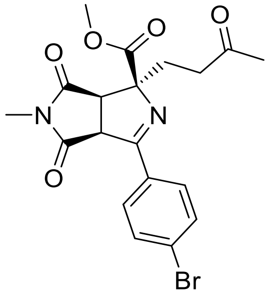

O
O
O
N
N
O
O
Br

7c

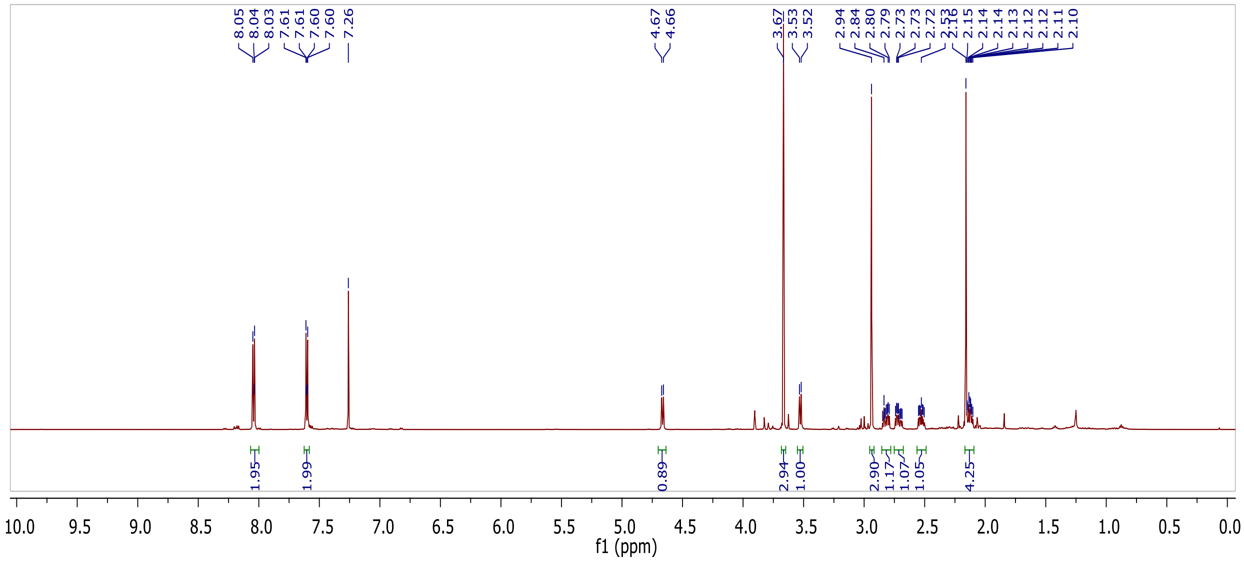

8.05
8.04
8.03
7.61
7.61
7.60
7.60
7.26
4.67
4.66
3.67
3.53
3.52
2.94
2.84
2.80
2.79
2.73
2.73
2.72
2.53
2.15
2.14
2.14
2.13
2.13
2.12
2.12
2.11
2.10
1.95
1.99
0.89
2.94
1.00
2.90
1.17
1.07
1.05
4.25
10.0
9.5
9.0
8.5
8.0
7.5
7.0
6.5
6.0
5.5
5.0
4.5
4.0
3.5
3.0
2.5
2.0
1.5
1.0
0.5
0.0
f1 (ppm)

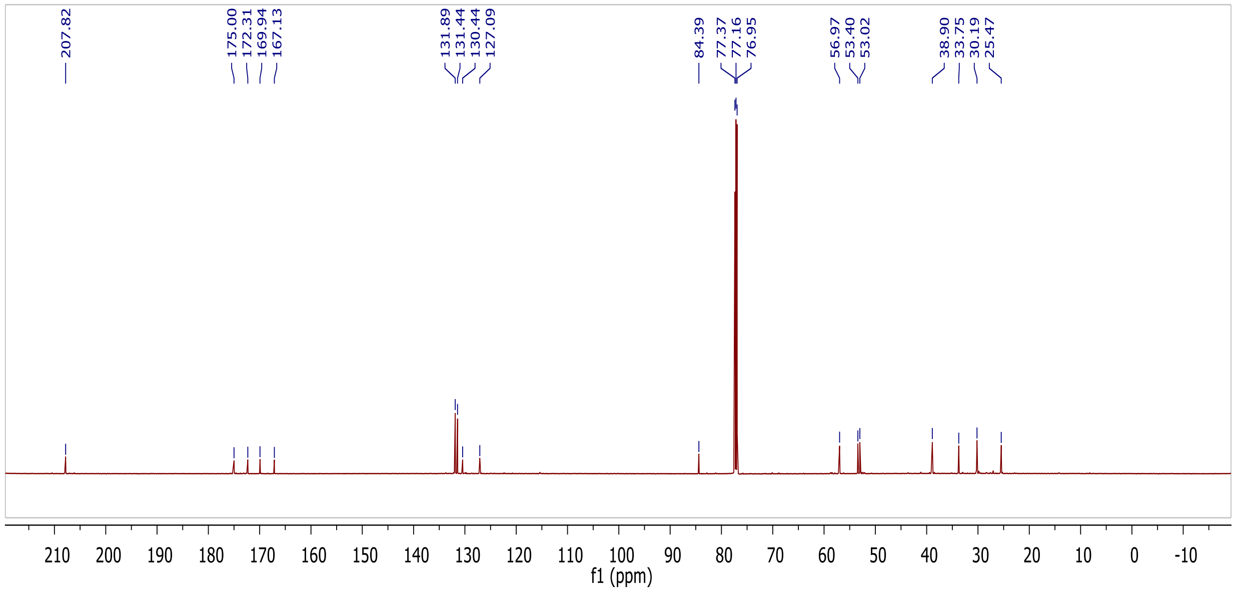

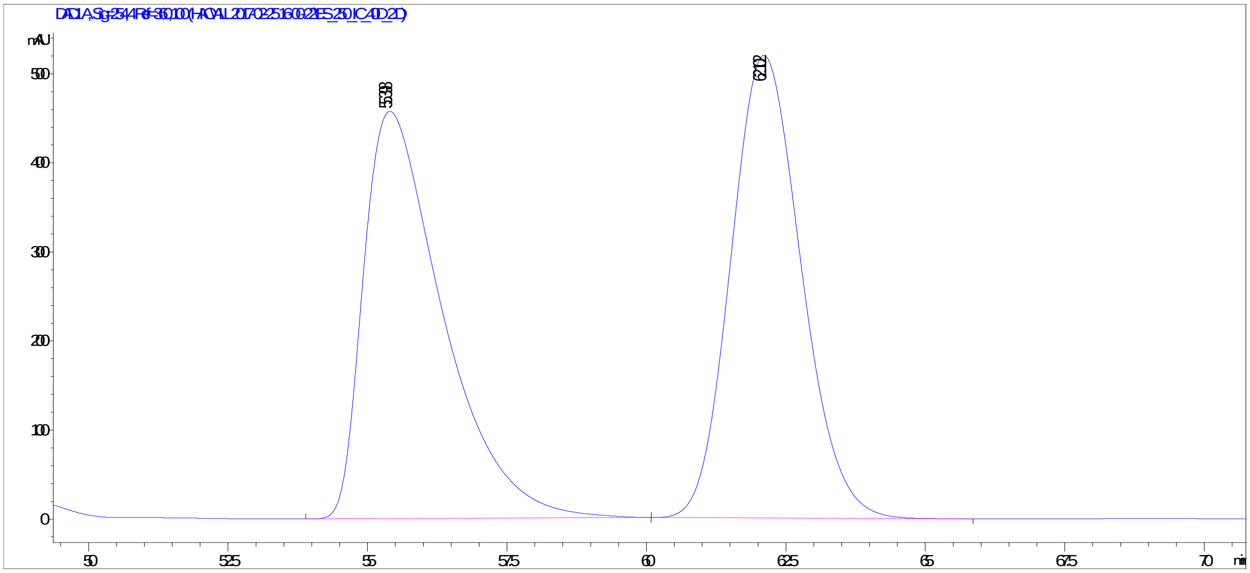

#	Time	Area	Height	Width	Area%	Symmetry
1	55.398	45261.4	457.4	1.4866	49.856	0.465
2	62.102	45523	519.5	1.3624	50.144	0.846

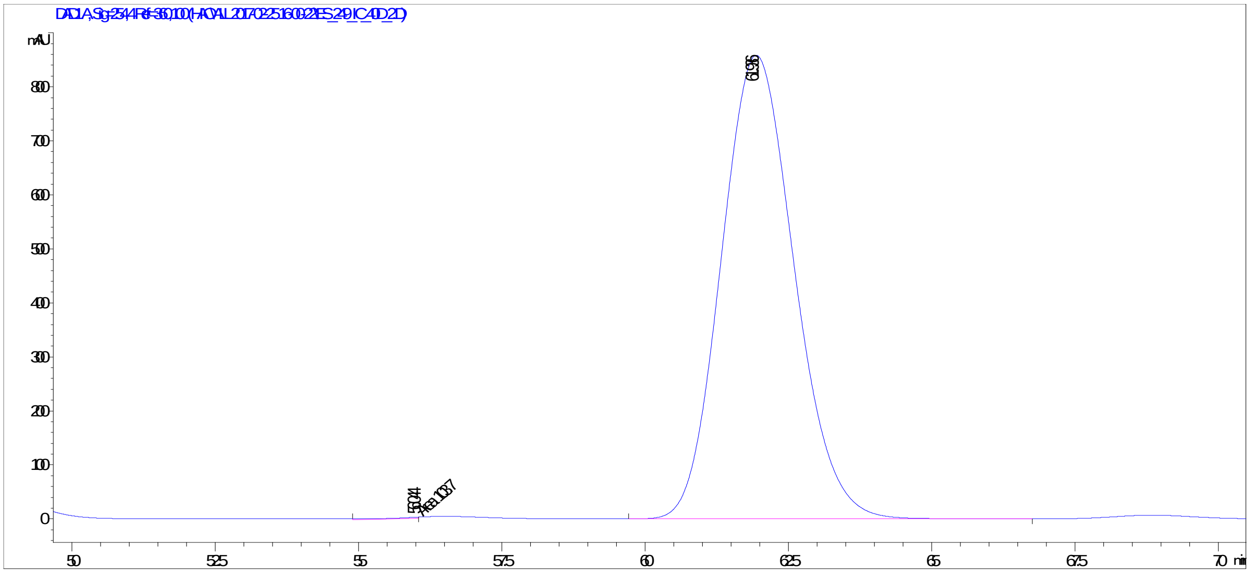

#	Time	Area	Height	Width	Area%	Symmetry
1	56.044	103.7	2.4	0.7283	0.137	0
2	61.936	75831.1	858.7	1.3923	99.863	0.876

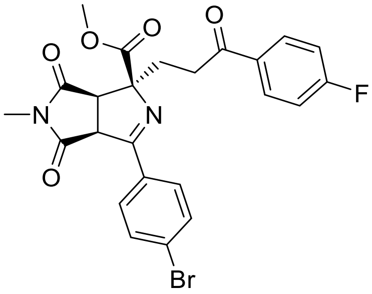

7d

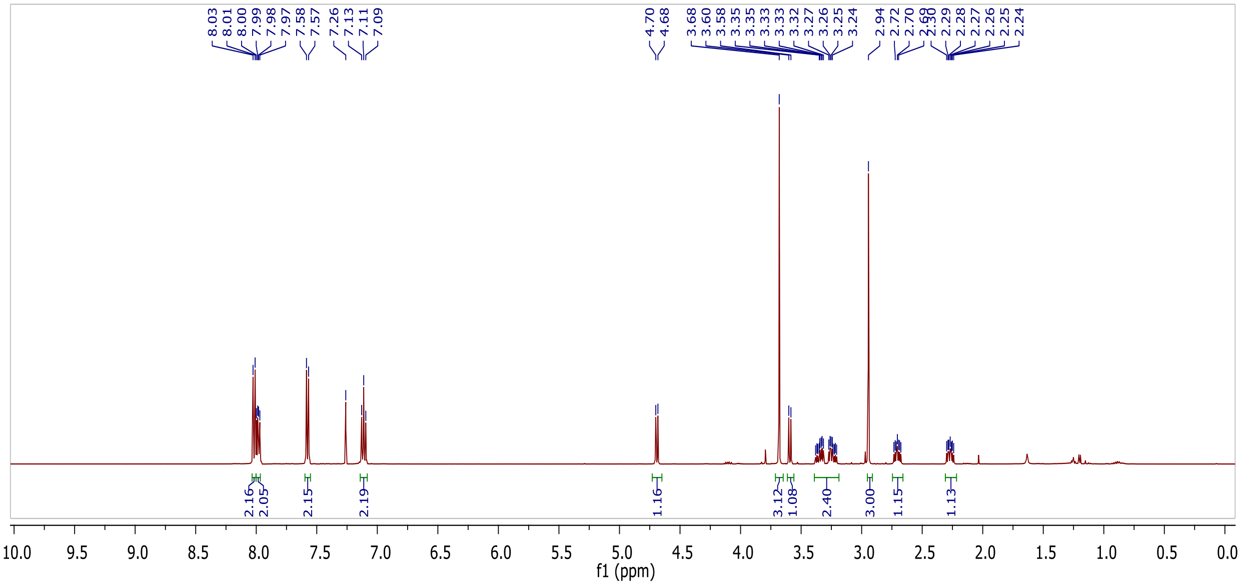
8.03
8.01
8.00
7.99
7.98
7.97
7.58
7.57
7.26
7.13
7.11
7.09
4.70
4.68
3.68
3.60
3.58
3.35
3.35
3.33
3.33
3.32
3.27
3.26
3.25
3.24
2.94
2.72
2.70
2.30
2.29
2.28
2.27
2.26
2.25
2.24
2.16
2.05
2.15
2.19
1.16
3.12
1.08
2.40
3.00
1.15
1.13
f1 (ppm)

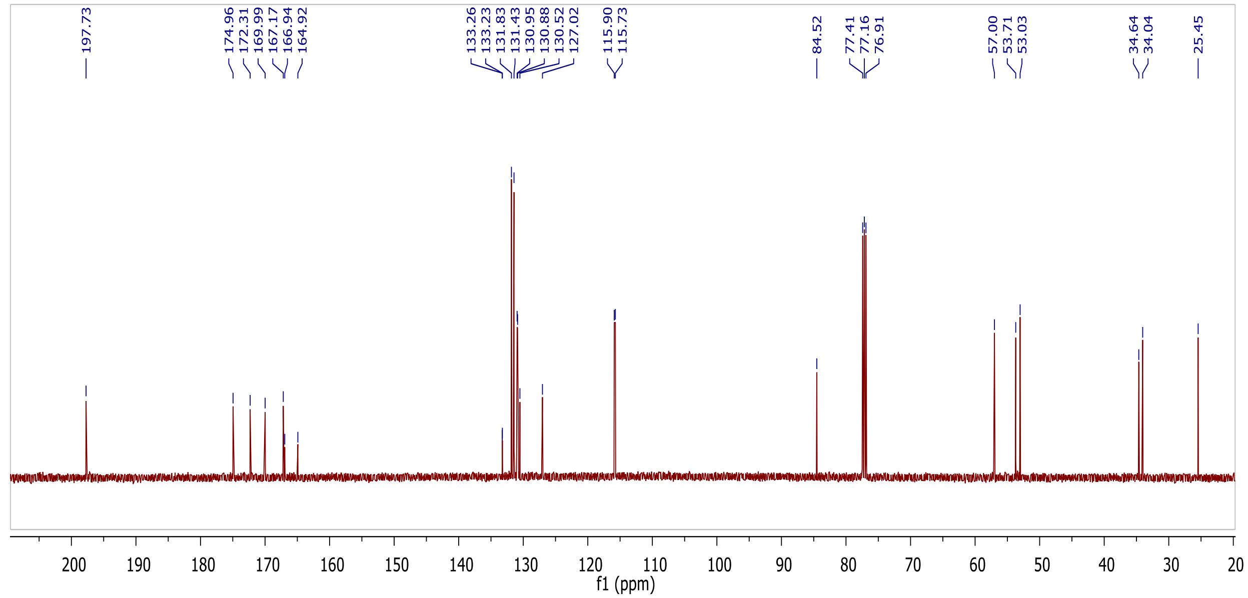
197.73
174.96
172.31
169.99
167.17
166.94
164.92
133.26
133.23
131.83
131.43
130.95
130.88
130.52
127.02
115.90
115.73
84.52
77.41
77.16
76.91
57.00
53.71
53.03
34.64
34.04
25.45
f1 (ppm)

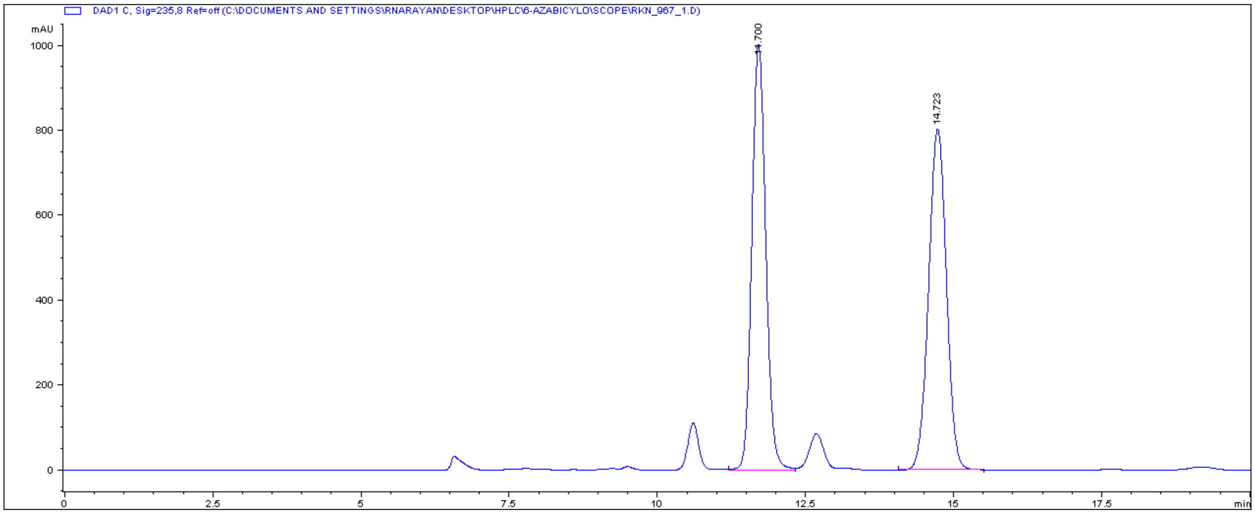

Signal 3: DAD1 C, Sig=235,8 Ref=off

Peak #	RetTime [min]	Type	Width [min]	Area [mAU*s]	Height [mAU]	Area %
1	11.700	BV	0.2523	1.63671e4	1003.65613	49.2370
2	14.723	BB	0.3287	1.68743e4	804.36115	50.7630

Totals : 3.32414e4 1808.01727

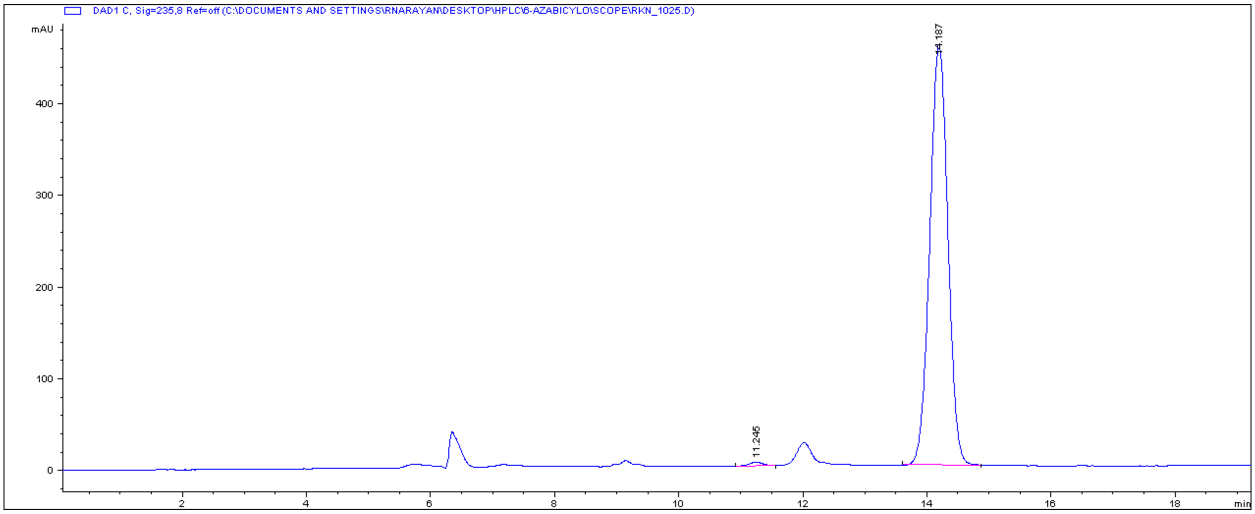

Signal 3: DAD1 C, Sig=235,8 Ref=off

Peak #	RetTime [min]	Type	Width [min]	Area [mAU*s]	Height [mAU]	Area %
1	11.245	BV	0.2228	65.61167	4.23292	0.7002
2	14.187	BB	0.3145	9304.34180	459.00684	99.2998

Totals : 9369.95347 463.23976

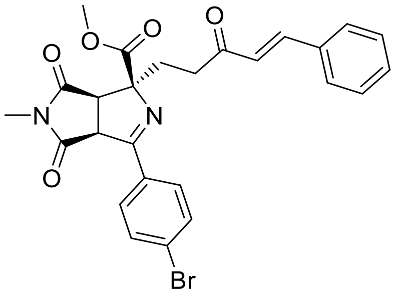

7e

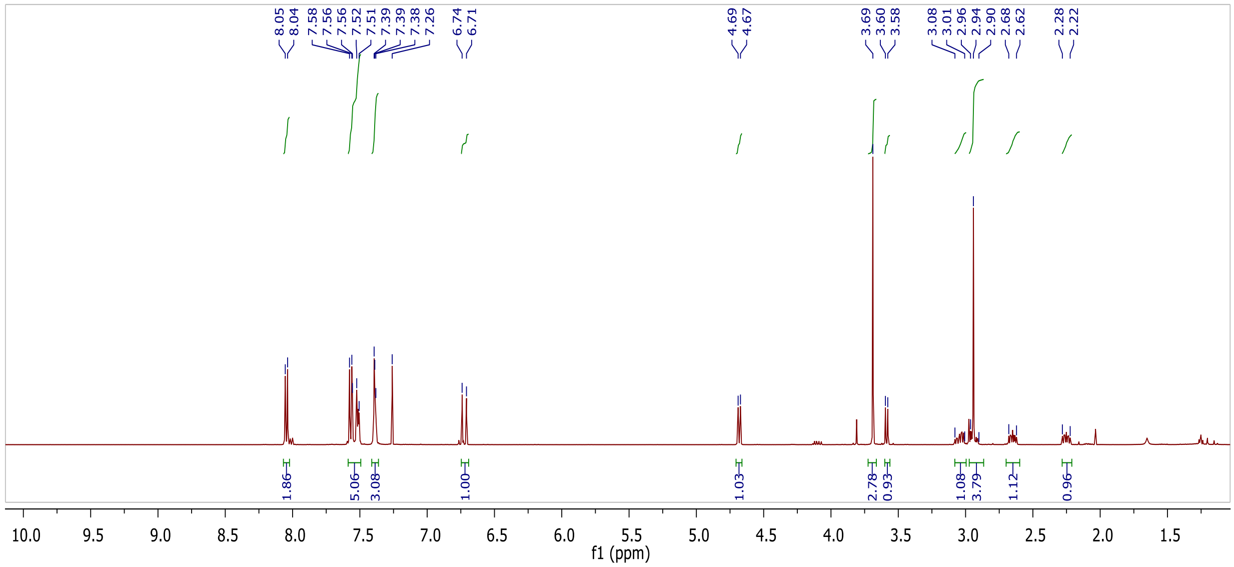
8.05
8.04
7.58
7.56
7.56
7.52
7.51
7.39
7.39
7.38
7.26
6.74
6.71
4.69
4.67
3.69
3.60
3.58
3.08
3.01
2.96
2.94
2.90
2.68
2.62
2.28
2.22
1.86
5.06
3.08
1.00
1.03
2.78
0.93
1.08
3.79
1.12
0.96
10.0 9.5 9.0 8.5 8.0 7.5 7.0 6.5 6.0 5.5 5.0 4.5 4.0 3.5 3.0 2.5 2.0 1.5
f1 (ppm)

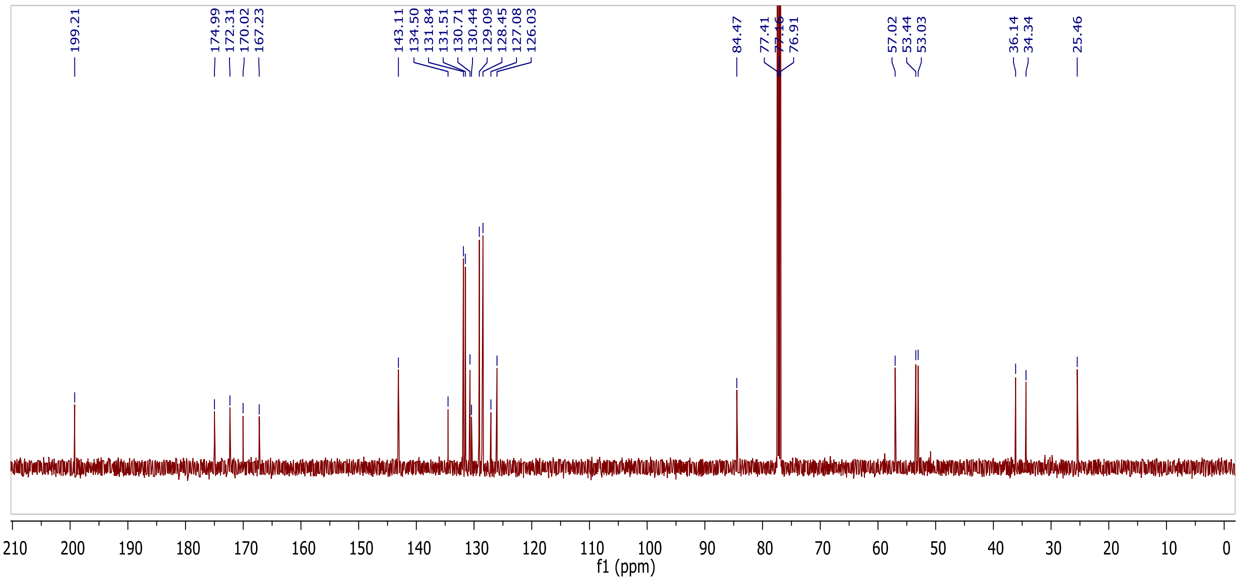
199.21
174.99
172.31
170.02
167.23
143.11
134.50
131.84
131.51
130.71
130.44
129.09
128.45
127.08
126.03
84.47
77.41
77.16
76.91
57.02
53.44
53.03
36.14
34.34
25.46
210 200 190 180 170 160 150 140 130 120 110 100 90 80 70 60 50 40 30 20 10 0
f1 (ppm)

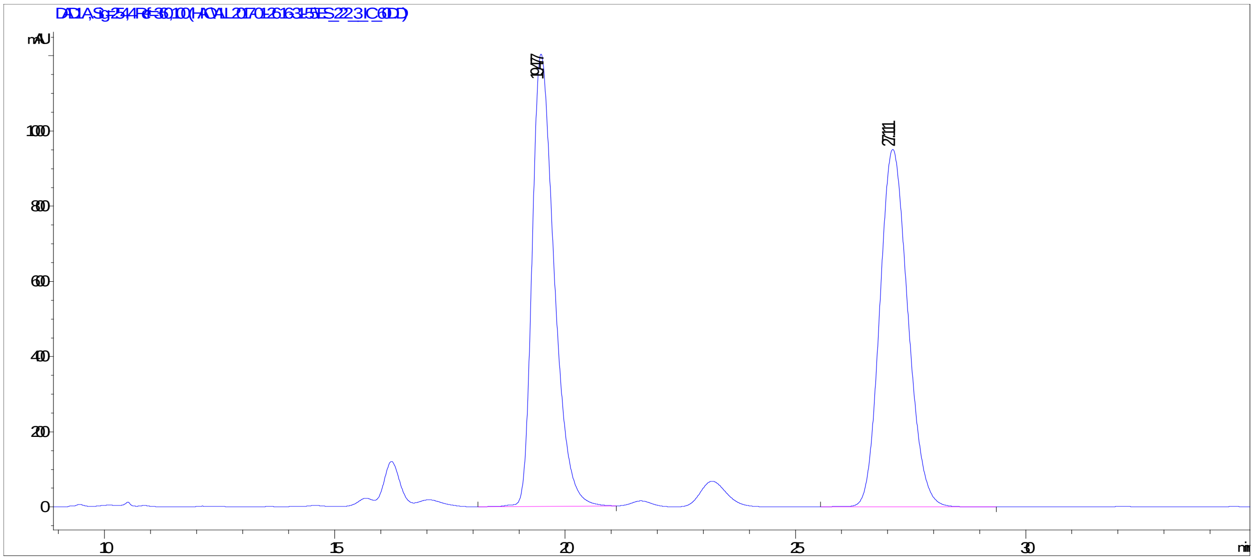

#	Time	Area	Height	Width	Area%	Symmetry
1	19.477	40013.3	1203.2	0.51	49.791	0.636
2	27.111	40349.7	951	0.663	50.209	0.823

Es-233

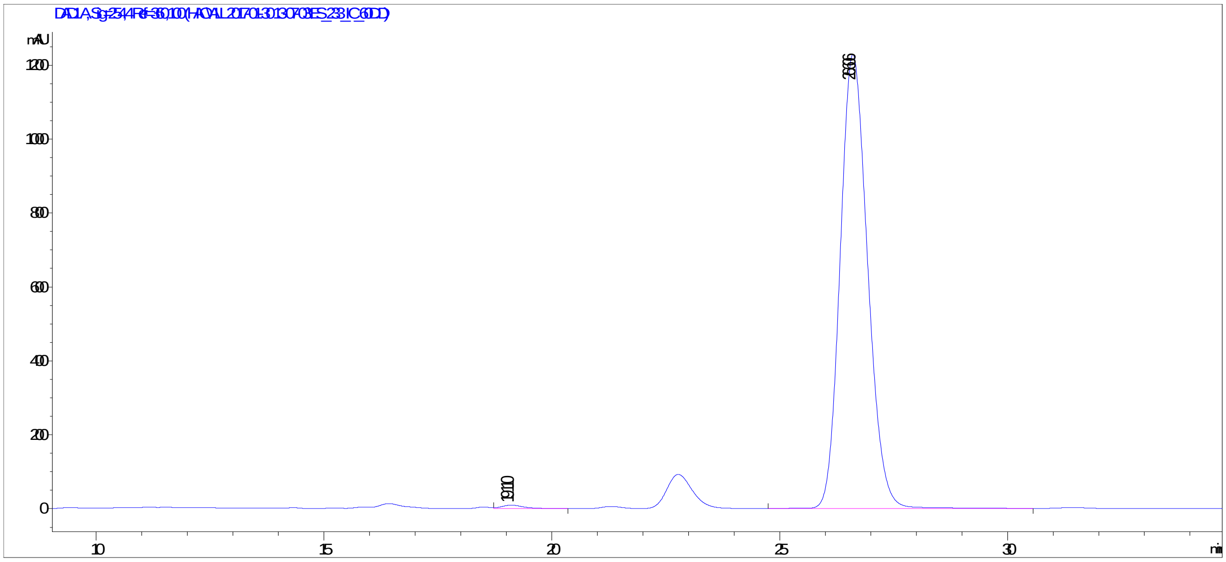

#	Time	Area	Height	Width	Area%	Symmetry
1	19.11	327.9	9.5	0.5163	0.634	0.701
2	26.606	51423.6	1229	0.6522	99.366	0.814

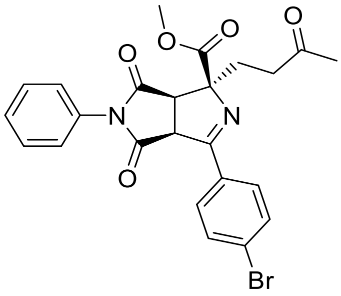
O
O
O
N
N
O
O
Br

7f

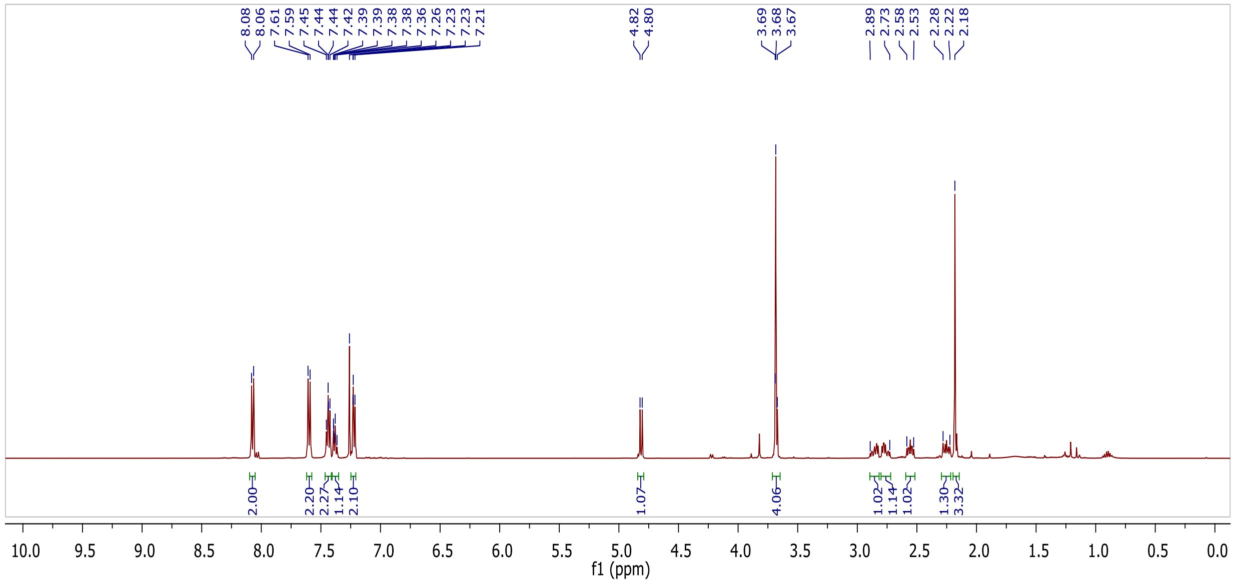
8.08
8.06
7.61
7.59
7.45
7.44
7.44
7.42
7.39
7.39
7.38
7.36
7.26
7.23
7.23
7.21
4.82
4.80
3.69
3.68
3.67
2.89
2.73
2.58
2.53
2.28
2.22
2.18
2.00
2.20
2.27
1.14
2.10
1.07
4.06
1.02
1.14
1.02
1.30
3.32
10.0 9.5 9.0 8.5 8.0 7.5 7.0 6.5 6.0 5.5 5.0 4.5 4.0 3.5 3.0 2.5 2.0 1.5 1.0 0.5 0.0
f1 (ppm)

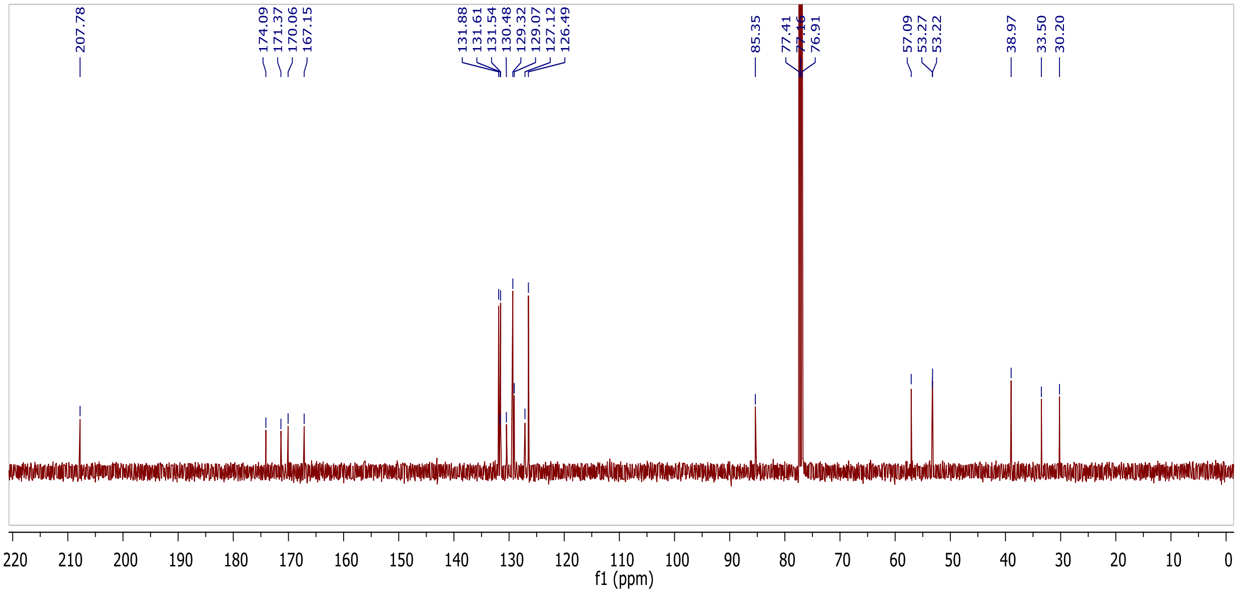
207.78
174.09
171.37
170.06
167.15
131.88
131.61
131.54
130.48
129.32
129.07
127.12
126.49
85.35
77.41
76.91
57.09
53.27
53.22
38.97
33.50
30.20
220 210 200 190 180 170 160 150 140 130 120 110 100 90 80 70 60 50 40 30 20 10 0
f1 (ppm)

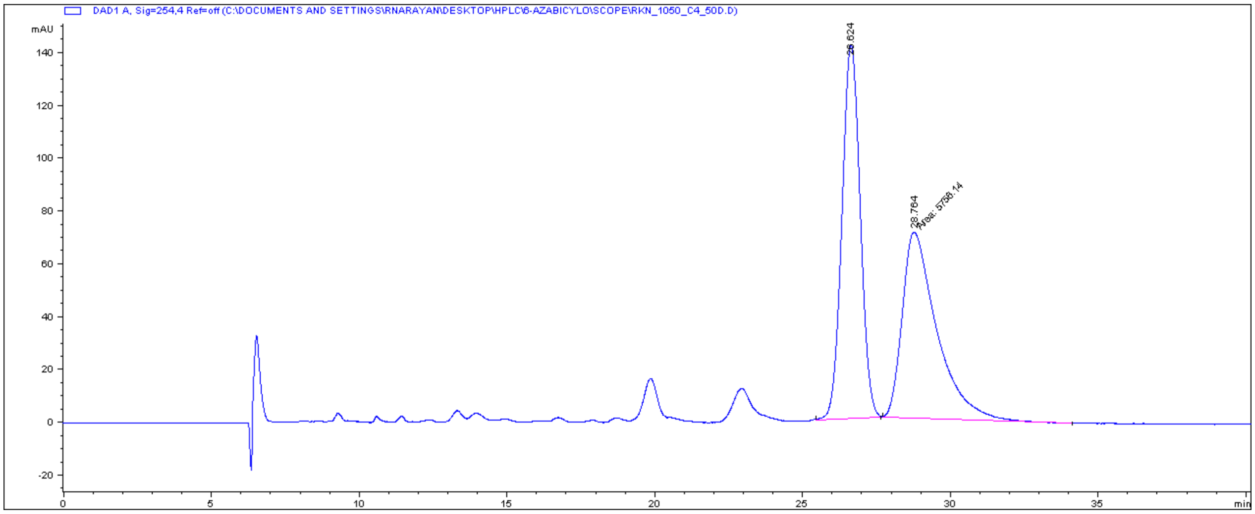

Signal 1: DAD1 A, Sig=254,4 Ref=off

Peak #	RetTime [min]	Type	Width [min]	Area [mAU*s]	Height [mAU]	Area %
1	26.624	BV	0.6493	6097.57275	141.91504	51.4402
2	28.764	MM	1.3608	5756.13721	70.49850	48.5598

Totals : 1.18537e4 212.41354

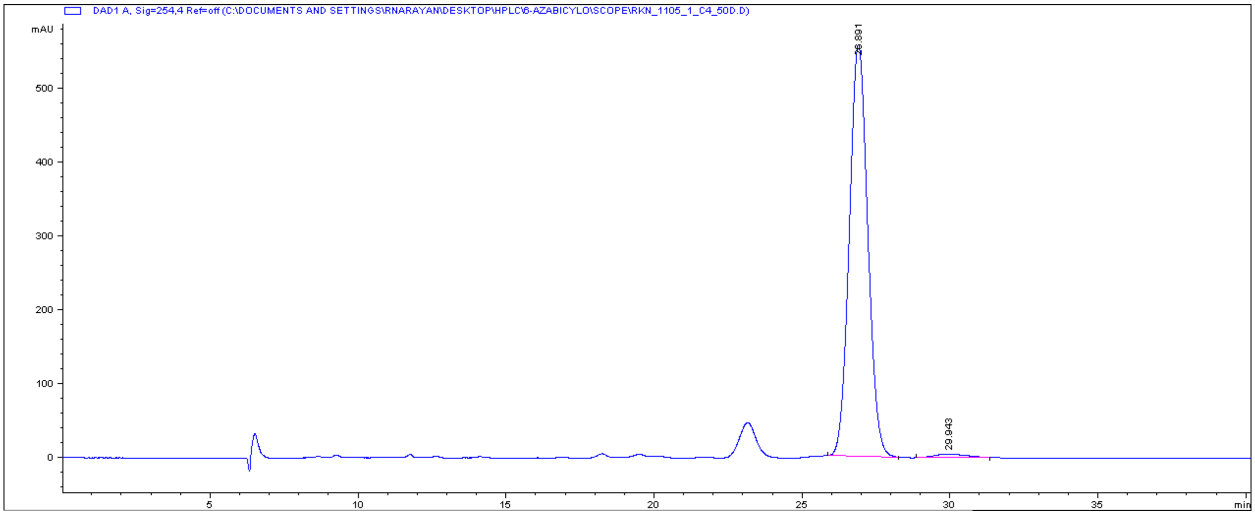

Signal 1: DAD1 A, Sig=254,4 Ref=off

Peak #	RetTime [min]	Type	Width [min]	Area [mAU*s]	Height [mAU]	Area %
1	26.891	BB	0.6761	2.42191e4	558.34576	98.4898
2	29.943	BB	0.8891	371.36539	4.92817	1.5102

Totals : 2.45905e4 563.27394

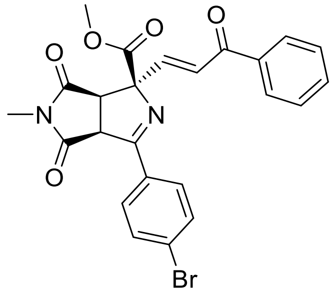

7g

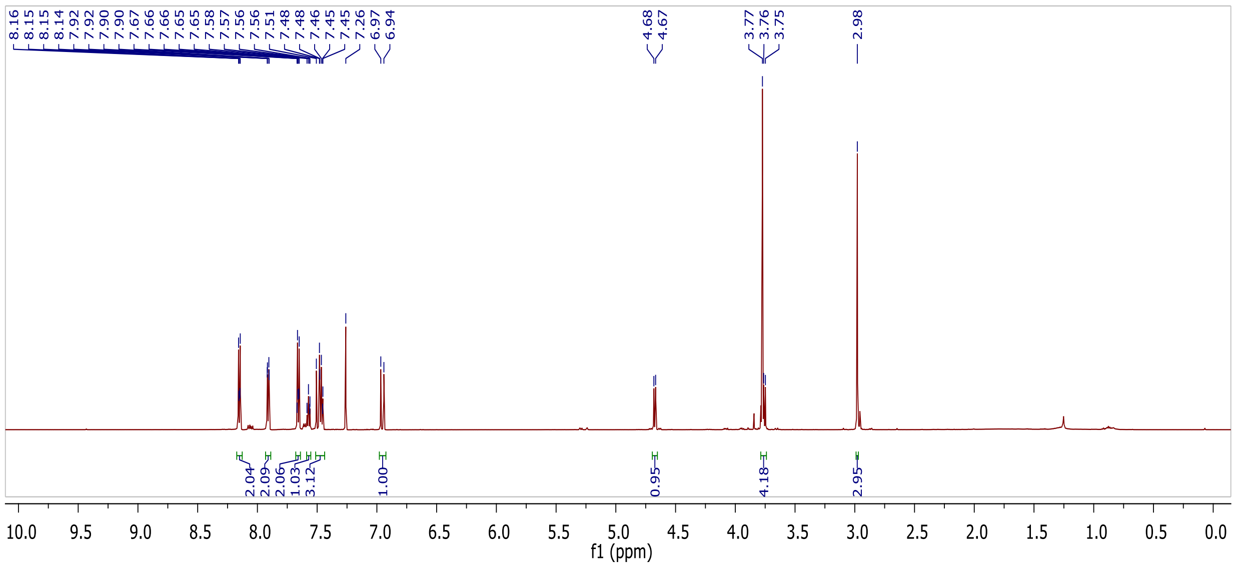

8.16
8.15
8.15
8.14
7.92
7.92
7.90
7.90
7.67
7.66
7.66
7.65
7.65
7.58
7.57
7.56
7.56
7.51
7.48
7.46
7.45
7.45
7.26
6.97
6.94
4.68
4.67
3.77
3.76
3.75
2.98
2.04
2.09
2.06
1.03
3.12
1.00
0.95
4.18
2.95
10.0 9.5 9.0 8.5 8.0 7.5 7.0 6.5 6.0 5.5 5.0 4.5 4.0 3.5 3.0 2.5 2.0 1.5 1.0 0.5 0.0
f1 (ppm)

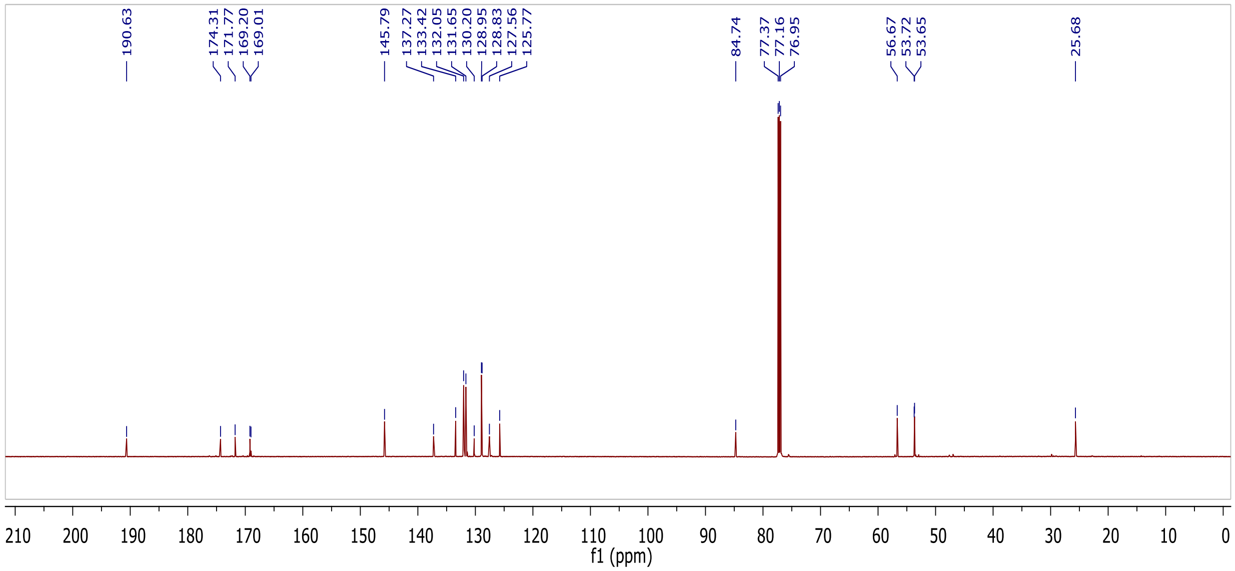

190.63
174.31
171.77
169.20
169.01
145.79
137.27
133.42
132.05
131.65
130.20
128.95
128.83
127.56
125.77
84.74
77.37
77.16
76.95
56.67
53.72
53.65
25.68
210 200 190 180 170 160 150 140 130 120 110 100 90 80 70 60 50 40 30 20 10 0
f1 (ppm)

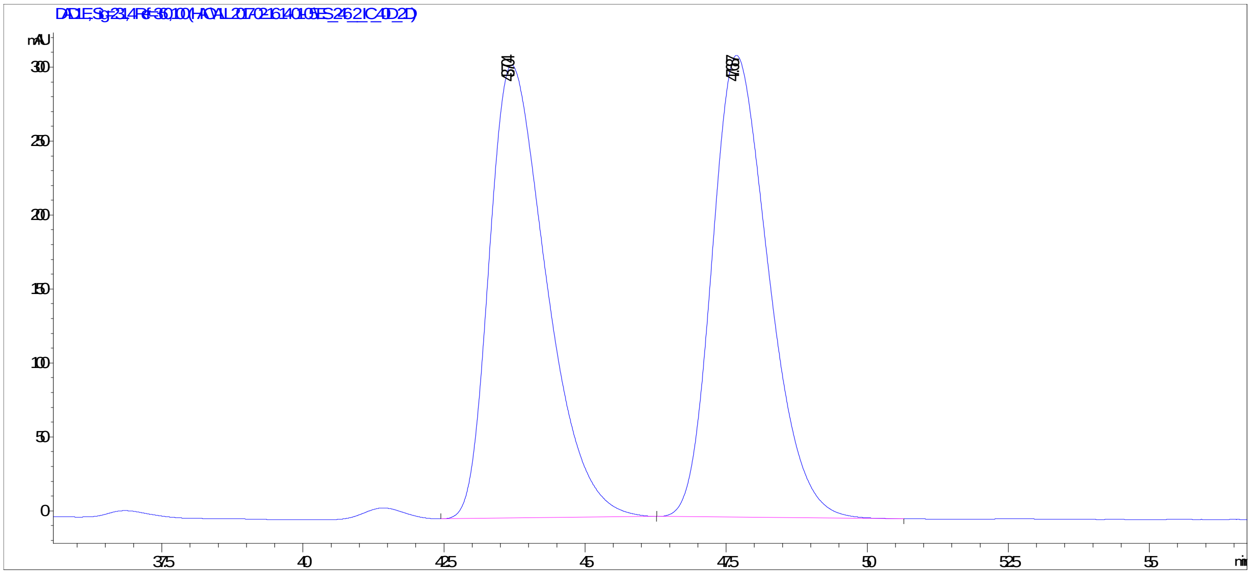

#	Time	Area	Height	Width	Area%	Symmetry
1	43.704	21576.8	306.3	1.0859	49.831	0.609
2	47.687	21723.6	312.3	1.0696	50.169	0.723

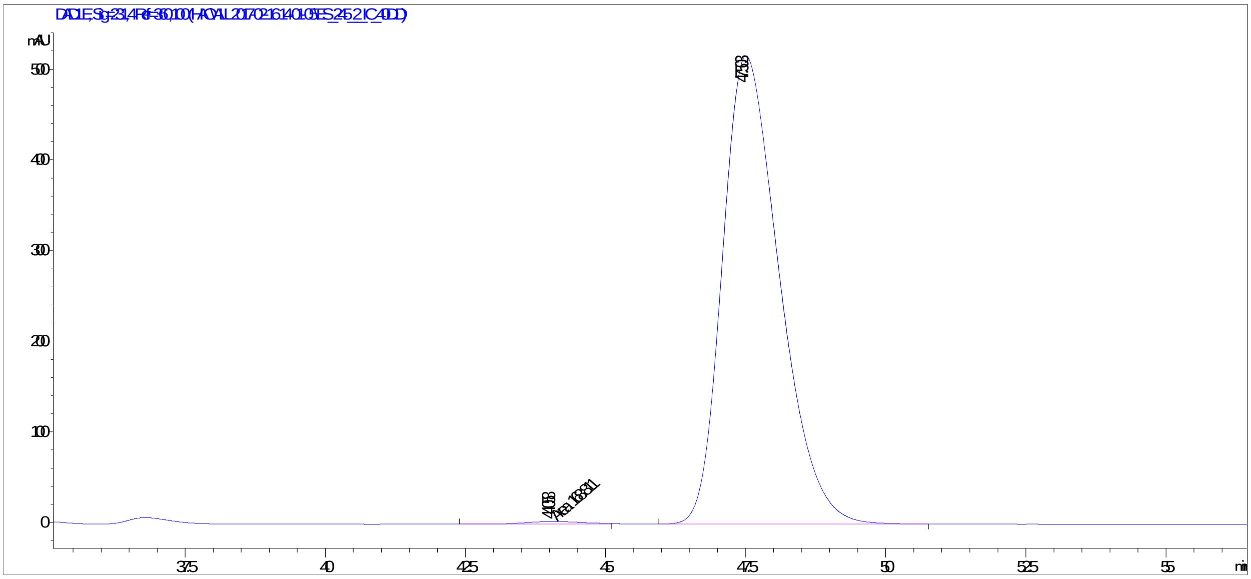

#	Time	Area	Height	Width	Area%	Symmetry
1	44.058	168.8	2.7	1.032	0.461	0.853
2	47.503	36424.5	516	1.0896	99.539	0.668

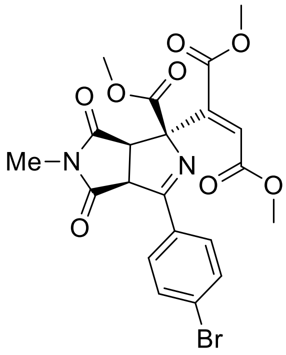

Me
N
O
O
O
O
N
O
O
O
O
Br

7h

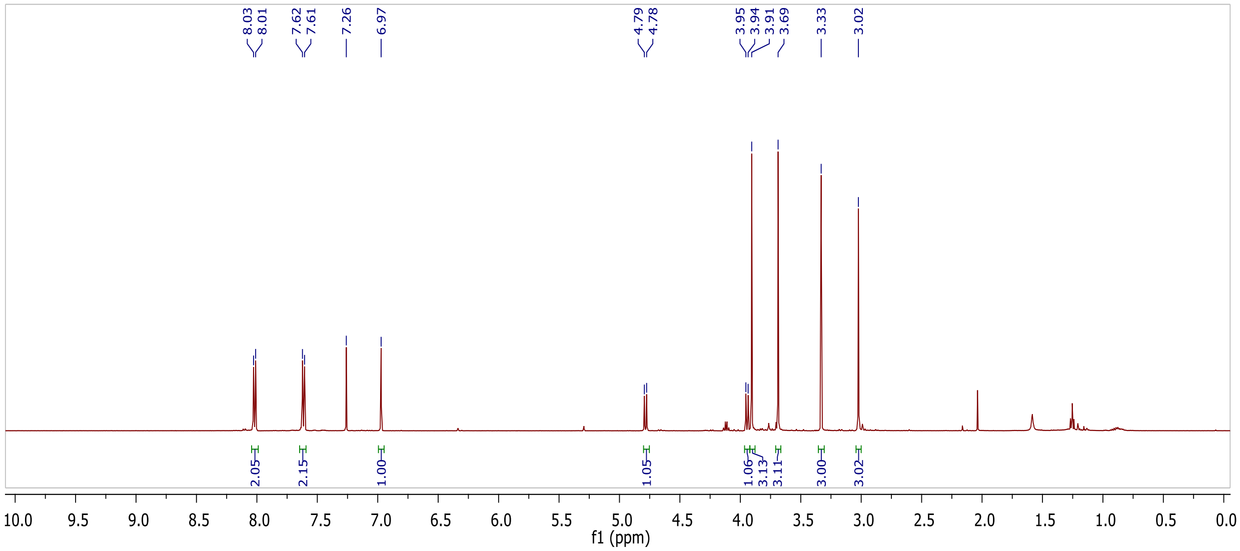

8.03
8.01
7.62
7.61
7.26
6.97
4.79
4.78
3.95
3.94
3.91
3.69
3.33
3.02
2.05
2.15
1.00
1.05
1.06
3.13
3.11
3.00
3.02
10.0
9.5
9.0
8.5
8.0
7.5
7.0
6.5
6.0
5.5
5.0
4.5
4.0
3.5
3.0
2.5
2.0
1.5
1.0
0.5
0.0
f1 (ppm)

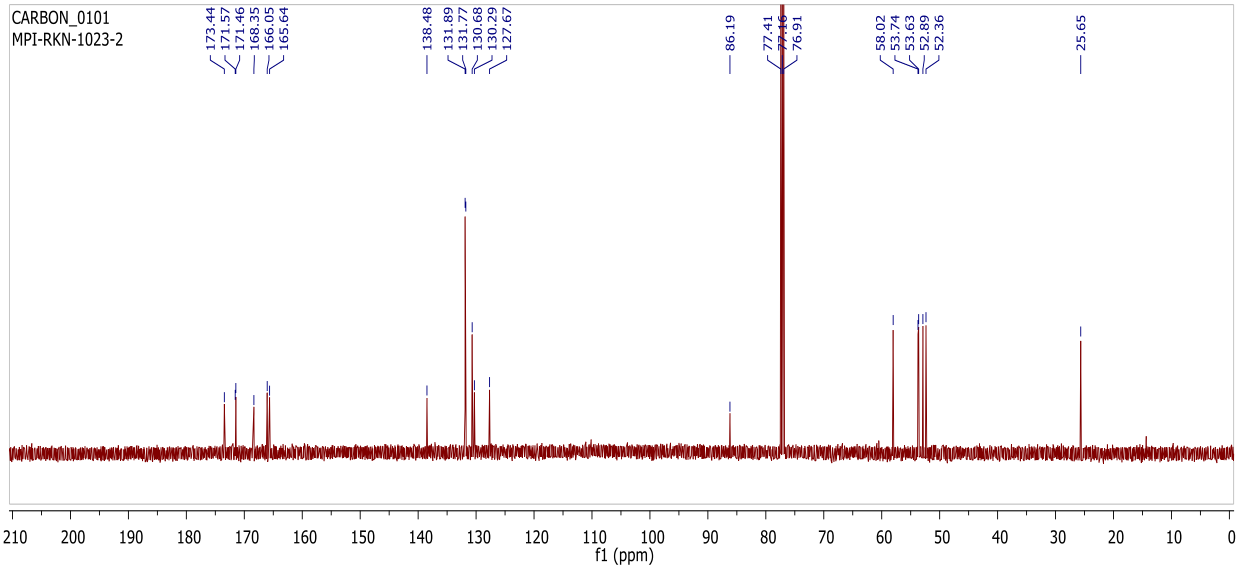

CARBON_0101
MPI-RKN-1023-2
173.44
171.57
171.46
168.35
166.05
165.64
138.48
131.89
131.77
130.68
130.29
127.67
86.19
77.41
77.16
76.91
58.02
53.74
53.63
52.89
52.36
25.65
210
200
190
180
170
160
150
140
130
120
110
100
90
80
70
60
50
40
30
20
10
0
f1 (ppm)

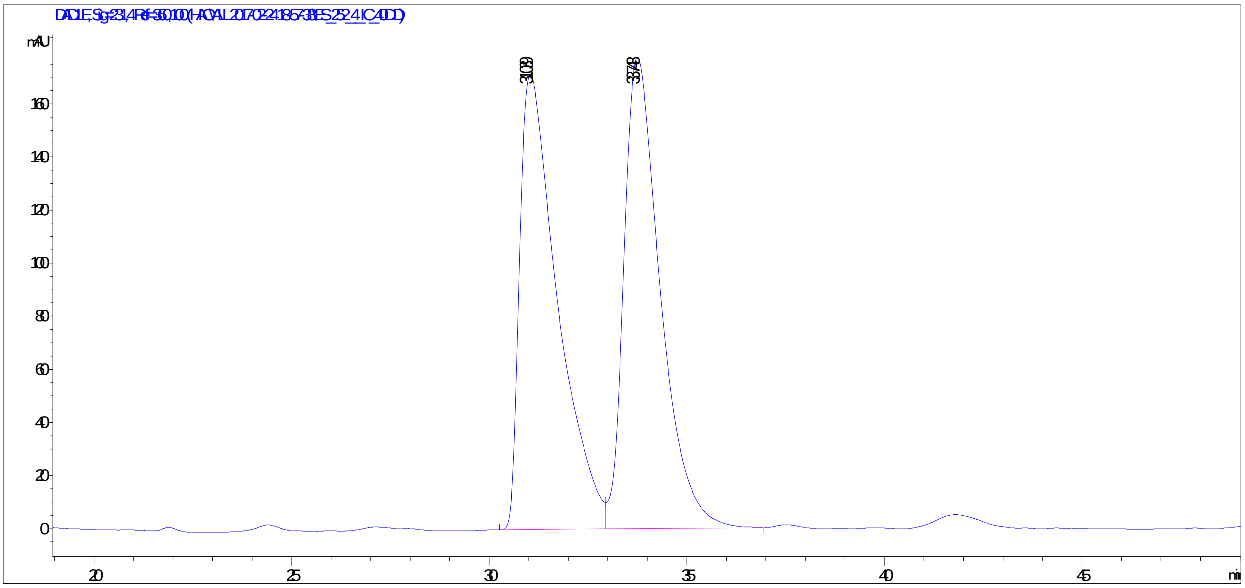

#	Time	Area	Height	Width	Area%	Symmetry
1	31.039	11412	171.6	0.9838	50.029	0.361
2	33.748	11398.8	177.7	0.9704	49.971	0.55

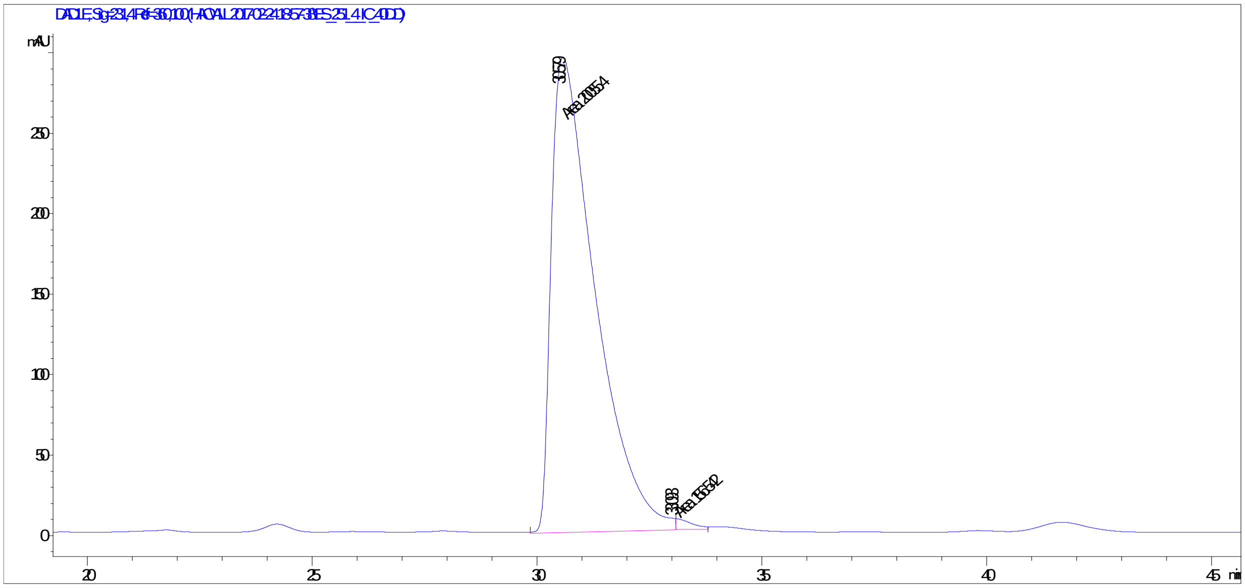

#	Time	Area	Height	Width	Area%	Symmetry
1	30.579	20055.4	295.5	1.1313	99.225	0.347
2	33.093	156.5	6.6	0.3958	0.775	0

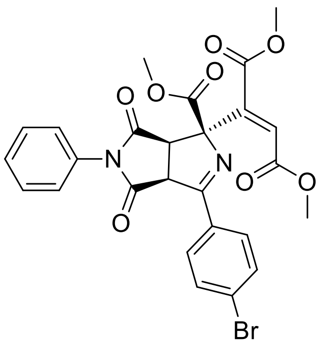

O
O
O
O
N
N
O
O
O
O
Br

7i

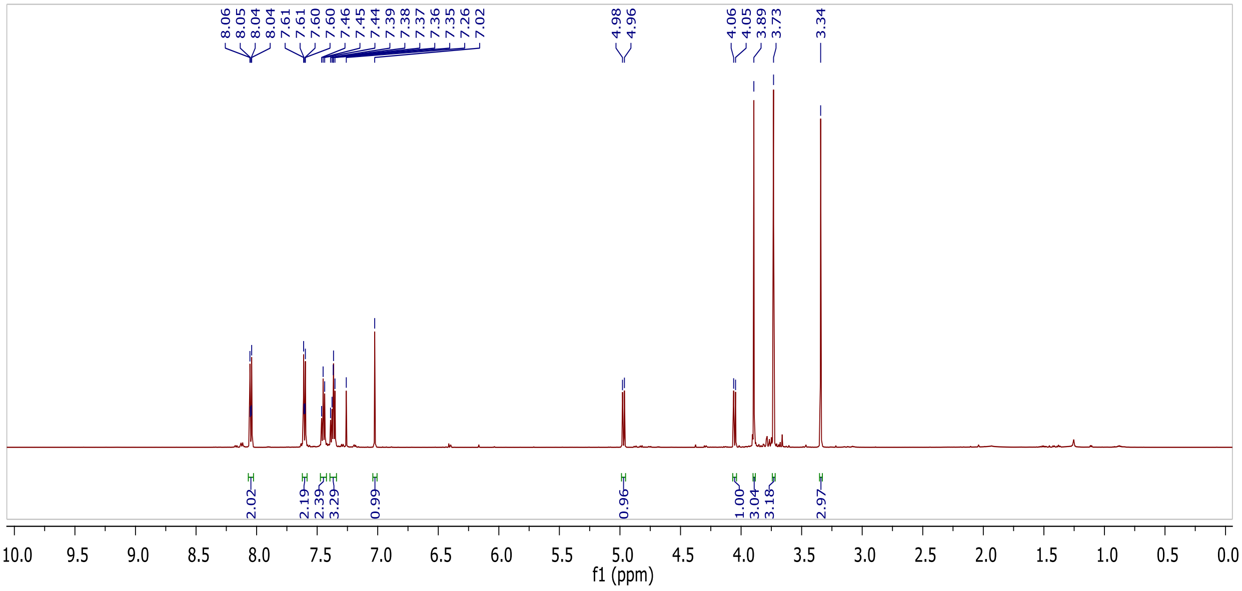

8.06
8.05
8.04
8.04
7.61
7.60
7.60
7.46
7.45
7.44
7.39
7.38
7.37
7.36
7.35
7.26
7.02
4.98
4.96
4.06
4.05
3.89
3.73
3.34
2.02
2.19
2.39
3.29
0.99
0.96
1.00
3.04
3.18
2.97
10.0 9.5 9.0 8.5 8.0 7.5 7.0 6.5 6.0 5.5 5.0 4.5 4.0 3.5 3.0 2.5 2.0 1.5 1.0 0.5 0.0
f1 (ppm)

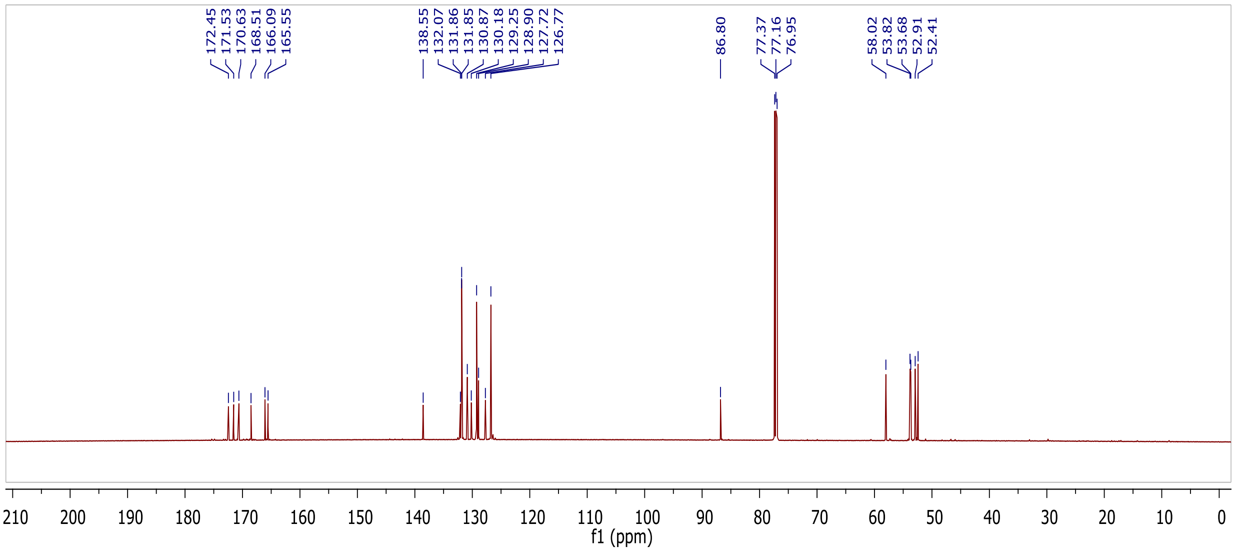

172.45
171.53
170.63
168.51
166.09
165.55
138.55
132.07
131.86
131.85
130.87
130.18
129.25
128.90
127.72
126.77
86.80
77.37
77.16
76.95
58.02
53.82
53.68
52.91
52.41
210 200 190 180 170 160 150 140 130 120 110 100 90 80 70 60 50 40 30 20 10 0
f1 (ppm)

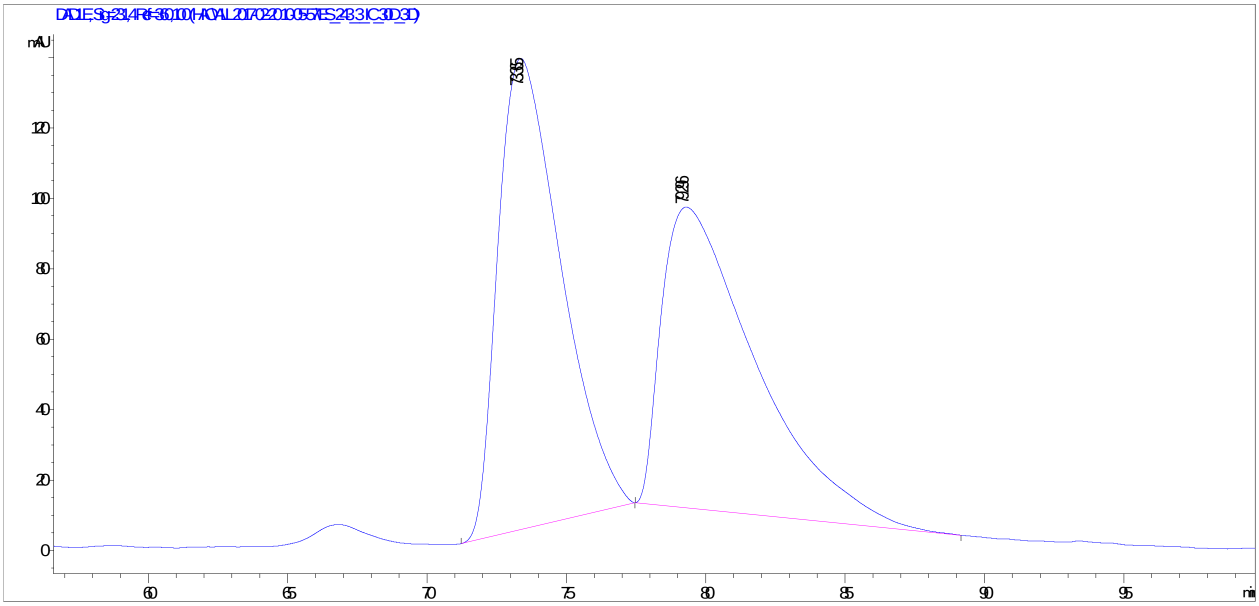

#	Time	Area	Height	Width	Area%	Symmetry
1	73.365	21044.5	133.8	2.3407	51.109	0.542
2	79.296	20131.3	85.4	3.1167	48.891	0.326

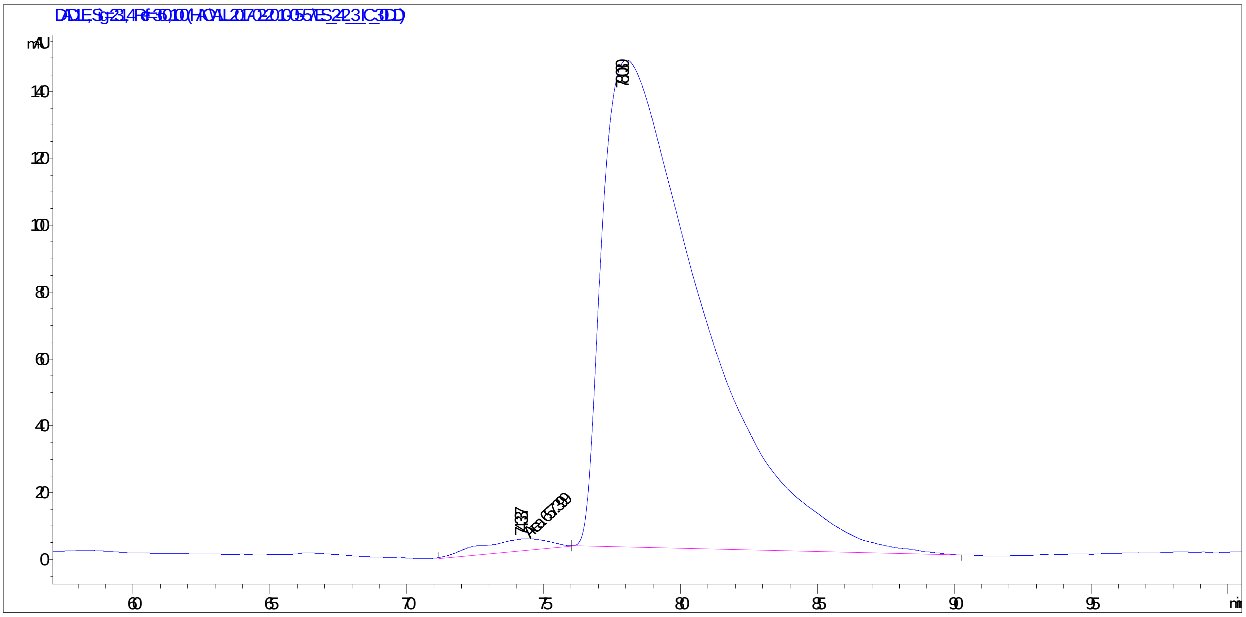

#	Time	Area	Height	Width	Area%	Symmetry
1	74.337	657.4	3.5	3.0948	1.743	2.302
2	78.03	37059.5	145.8	3.4767	98.257	0.298

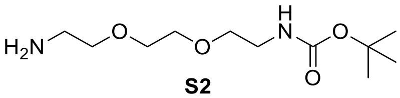

S2

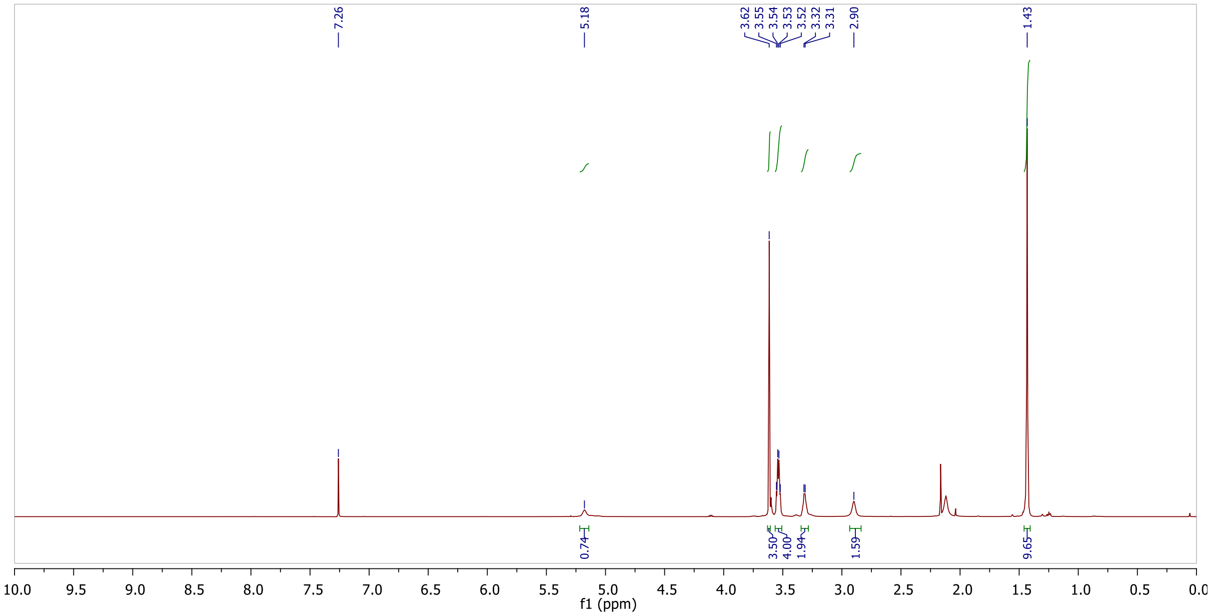

7.26
5.18
3.62
3.55
3.54
3.53
3.52
3.32
3.31
2.90
1.43
0.74
3.50
4.00
1.94
1.59
9.65
10.0 9.5 9.0 8.5 8.0 7.5 7.0 6.5 6.0 5.5 5.0 4.5 4.0 3.5 3.0 2.5 2.0 1.5 1.0 0.5 0.0
f1 (ppm)

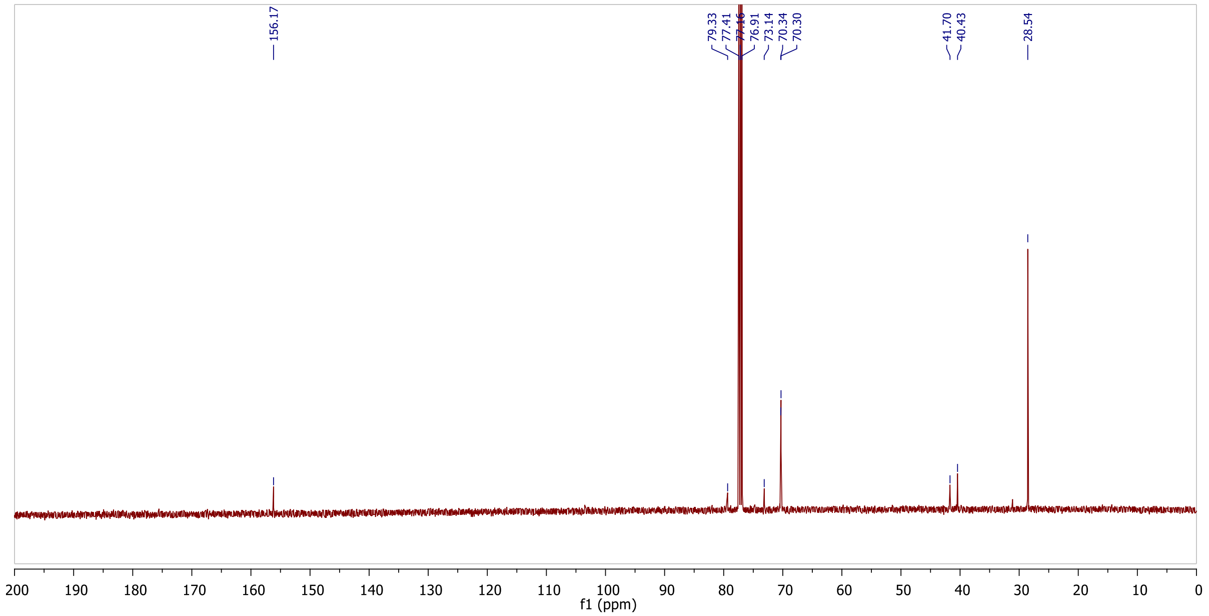

156.17
79.33
77.41
77.16
76.91
73.14
70.34
70.30
41.70
40.43
28.54
200 190 180 170 160 150 140 130 120 110 100 90 80 70 60 50 40 30 20 10 0
f1 (ppm)

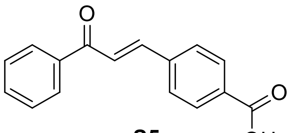
O
OH

S5a

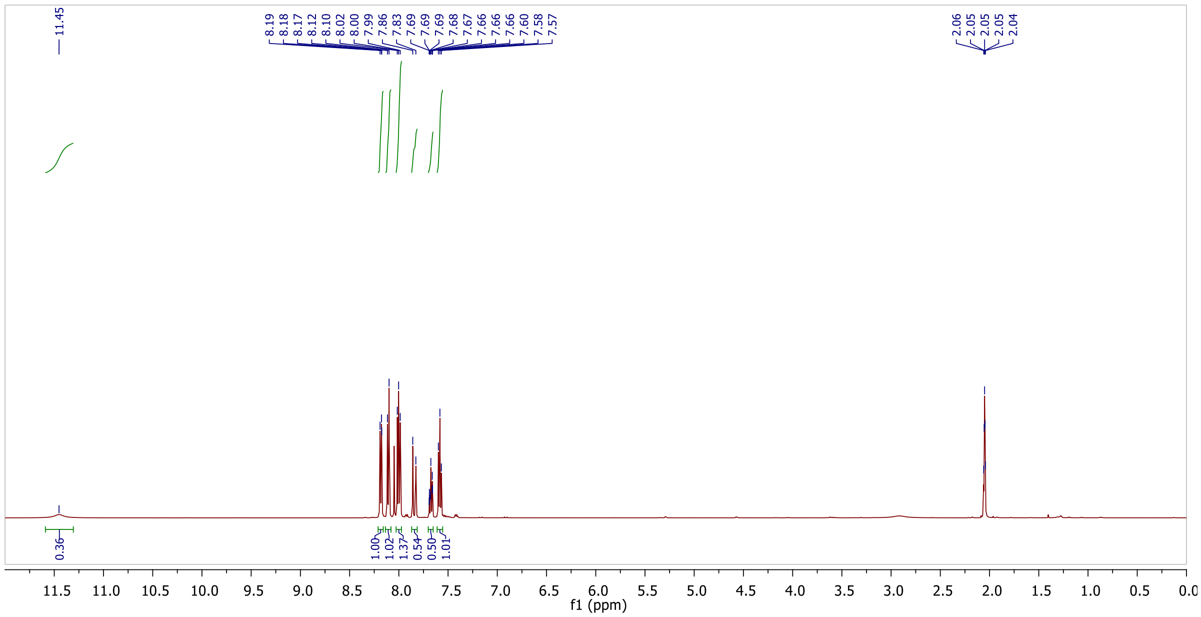
11.45
8.19 8.18 8.17 8.12 8.10 8.02 8.00 7.99 7.86 7.83 7.69 7.69 7.69 7.68 7.67 7.66 7.66 7.66 7.60 7.58 7.57
2.06 2.05 2.05 2.05 2.04
0.36
1.00 1.02 1.37 0.54 0.50 1.01
11.5 11.0 10.5 10.0 9.5 9.0 8.5 8.0 7.5 7.0 6.5 6.0 5.5 5.0 4.5 4.0 3.5 3.0 2.5 2.0 1.5 1.0 0.5 0.0
f1 (ppm)

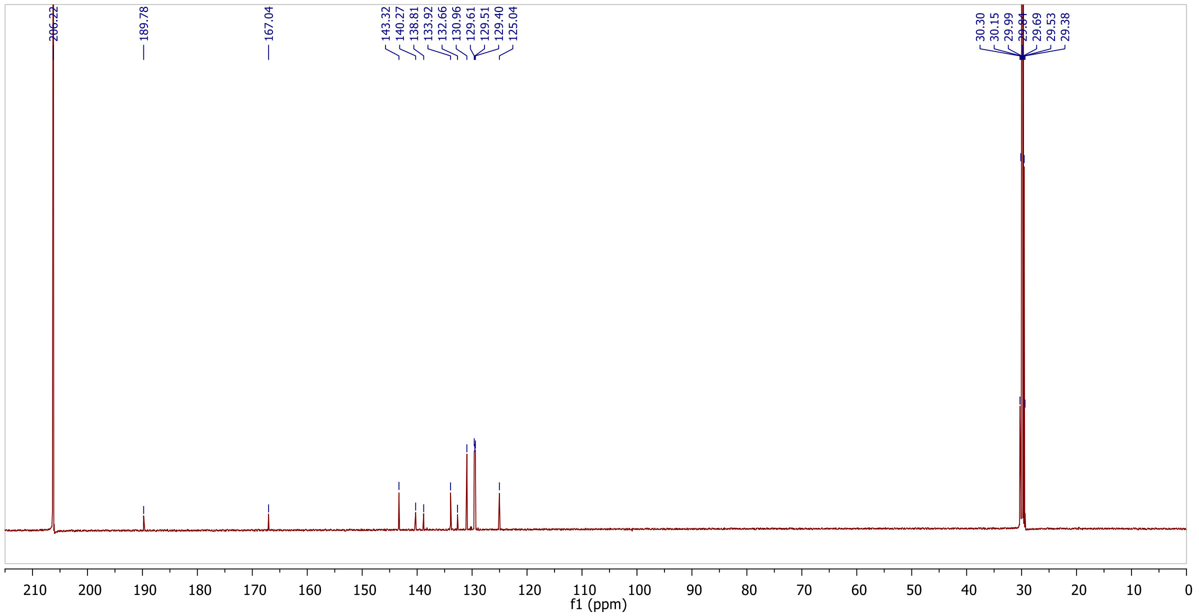
206.22
189.78
167.04
143.32 140.27 138.81 133.92 132.66 130.96 129.61 129.51 129.40 125.04
30.30 30.15 29.99 29.84 29.69 29.53 29.38
210 200 190 180 170 160 150 140 130 120 110 100 90 80 70 60 50 40 30 20 10 0
f1 (ppm)

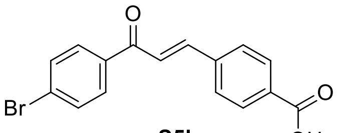
O
Br
O
OH

S5b

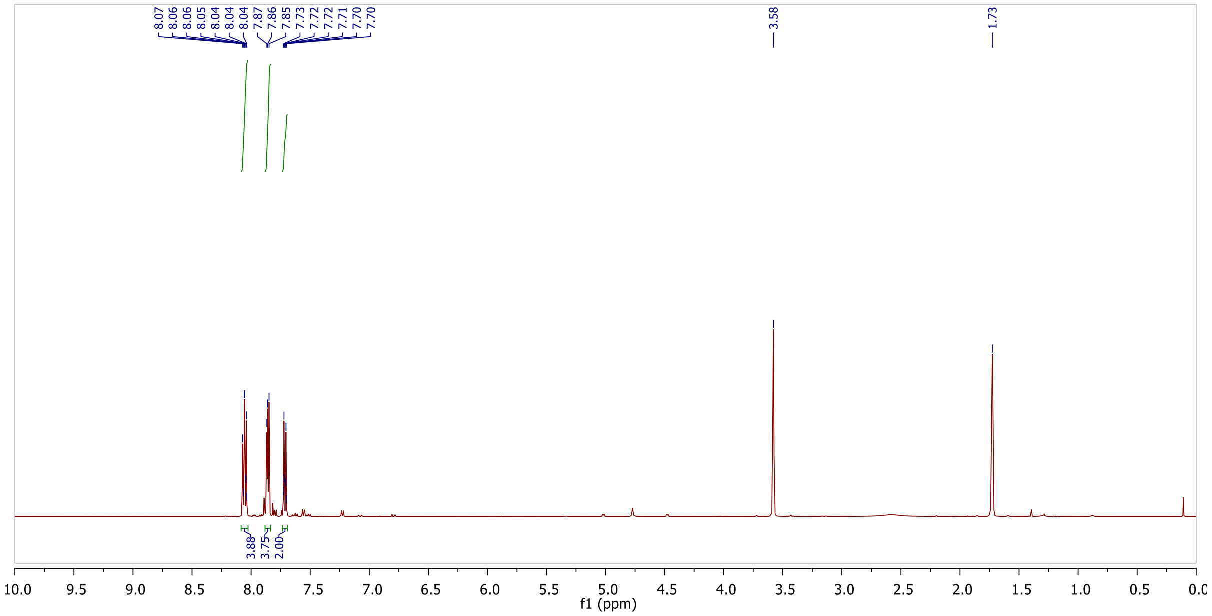
8.07
8.06
8.06
8.05
8.04
8.04
8.04
7.87
7.86
7.85
7.73
7.72
7.72
7.71
7.70
7.70
3.58
1.73
3.88
3.75
2.00
10.0
9.5
9.0
8.5
8.0
7.5
7.0
6.5
6.0
5.5
5.0
4.5
4.0
3.5
3.0
2.5
2.0
1.5
1.0
0.5
0.0
f1 (ppm)

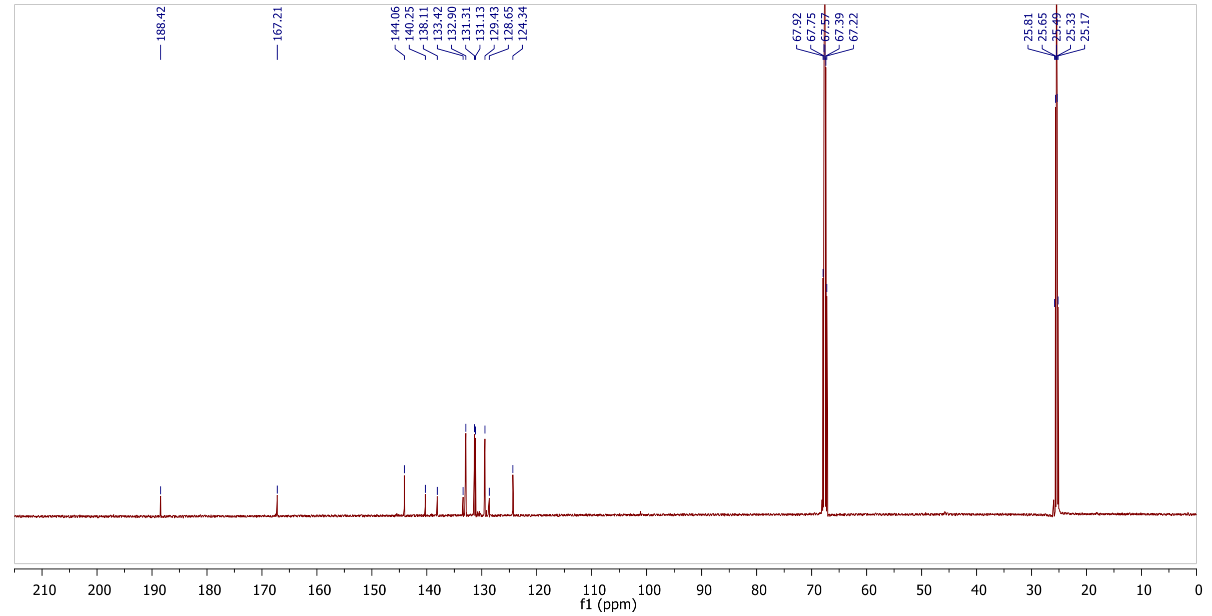
188.42
167.21
144.06
140.25
138.11
133.42
132.90
131.31
131.13
129.43
128.65
124.34
67.92
67.75
67.57
67.39
67.22
25.81
25.65
25.49
25.33
25.17
210
200
190
180
170
160
150
140
130
120
110
100
90
80
70
60
50
40
30
20
10
0
f1 (ppm)

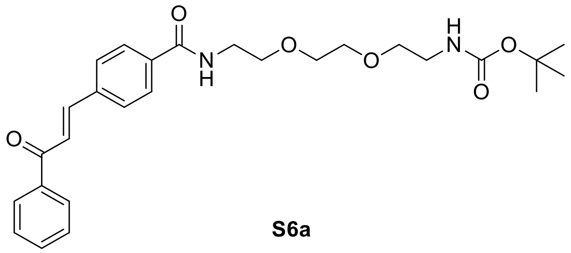
O
N
H
O
O
H
N
O
O
O

S6a

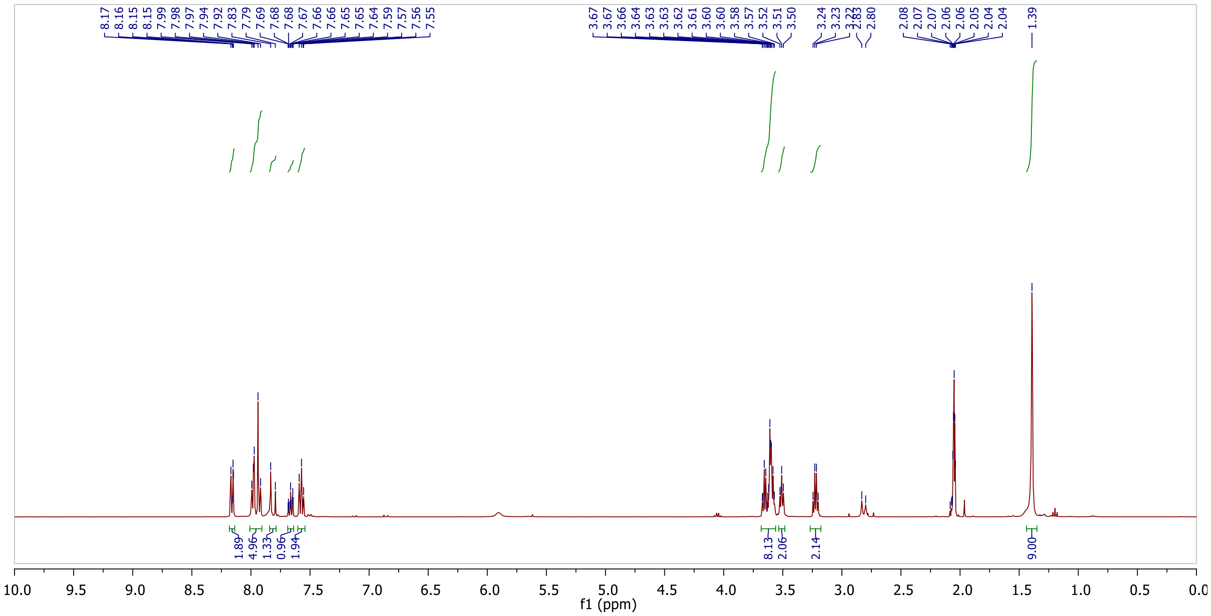
8.17
8.16
8.15
7.99
7.98
7.97
7.94
7.92
7.83
7.79
7.69
7.68
7.68
7.67
7.66
7.65
7.65
7.64
7.59
7.57
7.56
7.55
3.67
3.67
3.66
3.64
3.63
3.63
3.62
3.61
3.60
3.60
3.58
3.57
3.52
3.51
3.50
3.24
3.23
3.22
2.83
2.80
2.08
2.07
2.07
2.06
2.06
2.05
2.04
2.04
1.39
1.89
4.96
1.33
0.96
1.94
8.13
2.06
2.14
9.00
10.0 9.5 9.0 8.5 8.0 7.5 7.0 6.5 6.0 5.5 5.0 4.5 4.0 3.5 3.0 2.5 2.0 1.5 1.0 0.5 0.0
f1 (ppm)

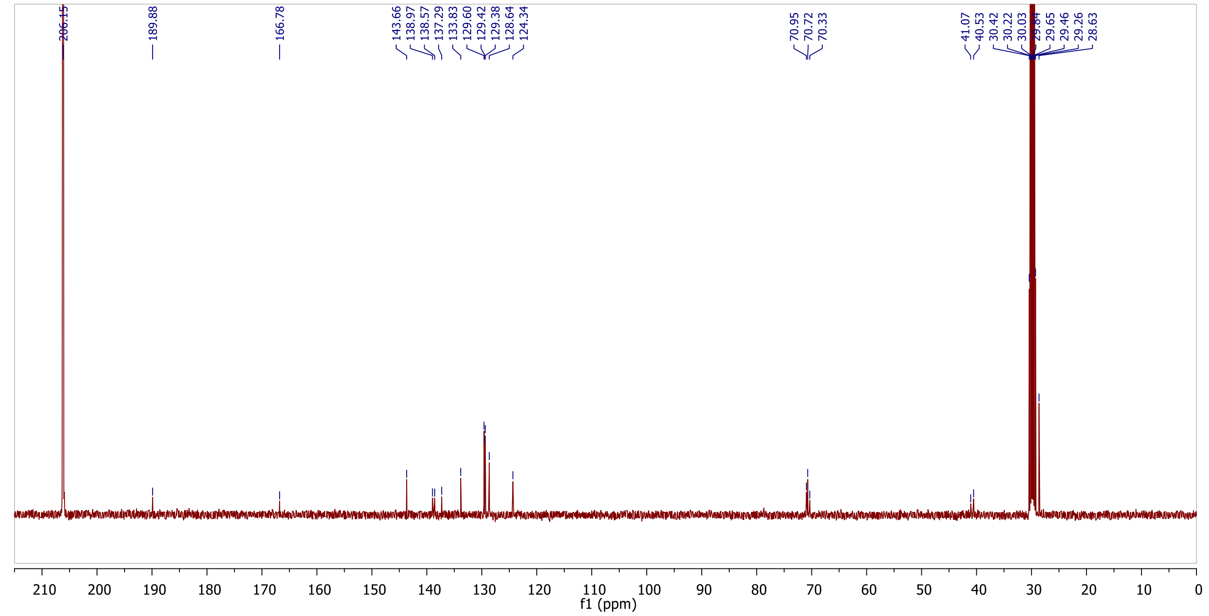
206.15
189.88
166.78
143.66
138.97
138.57
137.29
133.83
129.60
129.42
129.38
128.64
124.34
70.95
70.72
70.33
41.07
40.53
30.42
30.22
30.03
29.84
29.65
29.46
29.26
28.63
210 200 190 180 170 160 150 140 130 120 110 100 90 80 70 60 50 40 30 20 10 0
f1 (ppm)

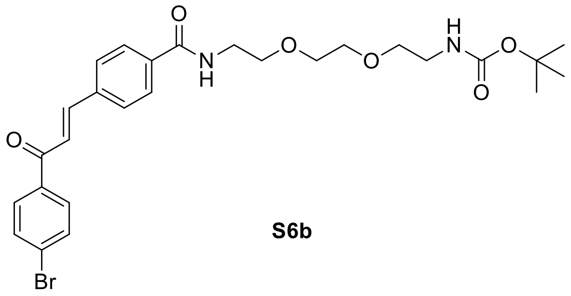

S6b

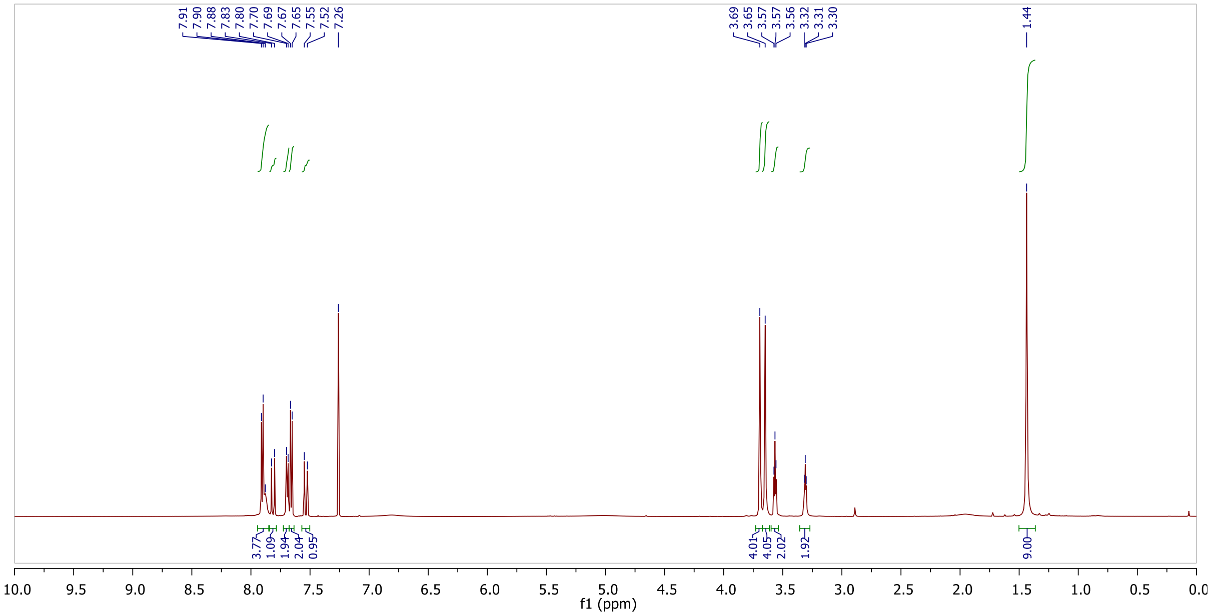
7.91
7.90
7.88
7.83
7.80
7.70
7.69
7.67
7.65
7.55
7.52
7.26
3.69
3.65
3.57
3.57
3.56
3.32
3.31
3.30
1.44
3.77
1.09
1.94
2.04
0.95
4.01
4.05
2.02
1.92
9.00
10.0
9.5
9.0
8.5
8.0
7.5
7.0
6.5
6.0
5.5
5.0
4.5
4.0
3.5
3.0
2.5
2.0
1.5
1.0
0.5
0.0
f1 (ppm)

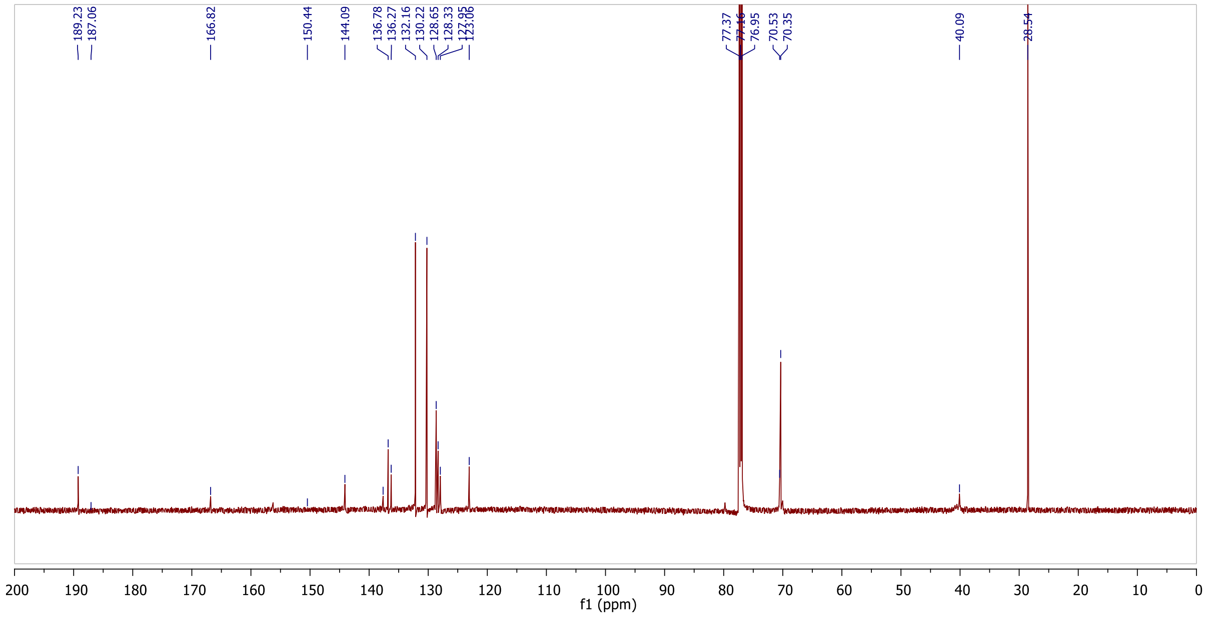
189.23
187.06
166.82
150.44
144.09
136.78
136.27
132.16
130.22
128.65
128.33
127.95
123.06
77.37
77.16
76.95
70.53
70.35
40.09
28.54
200
190
180
170
160
150
140
130
120
110
100
90
80
70
60
50
40
30
20
10
0
f1 (ppm)

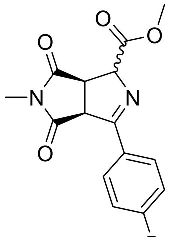

S9

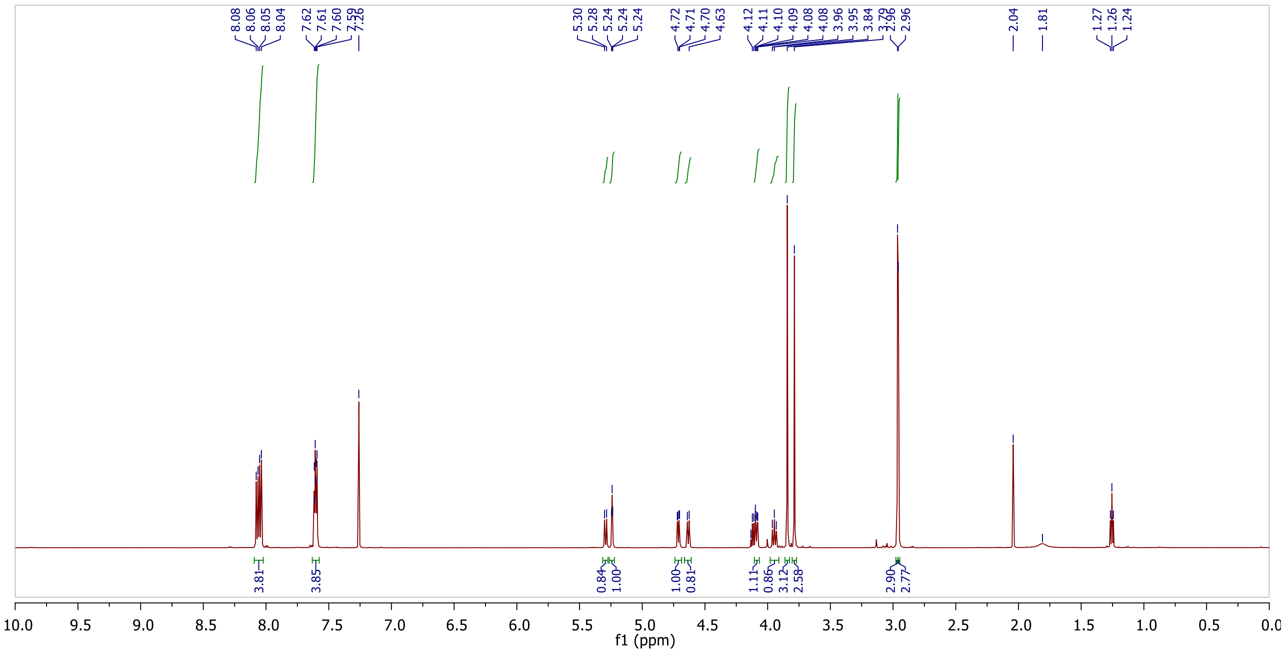
8.08
8.06
8.05
8.04
7.62
7.61
7.60
7.59
7.26
5.30
5.28
5.24
5.24
5.24
4.72
4.71
4.70
4.63
4.12
4.11
4.10
4.09
4.08
4.08
3.96
3.95
3.84
3.79
2.98
2.96
2.04
1.81
1.27
1.26
1.24
3.81
3.85
0.84
1.00
1.00
0.81
1.11
0.86
3.12
2.58
2.90
2.77
f1 (ppm)

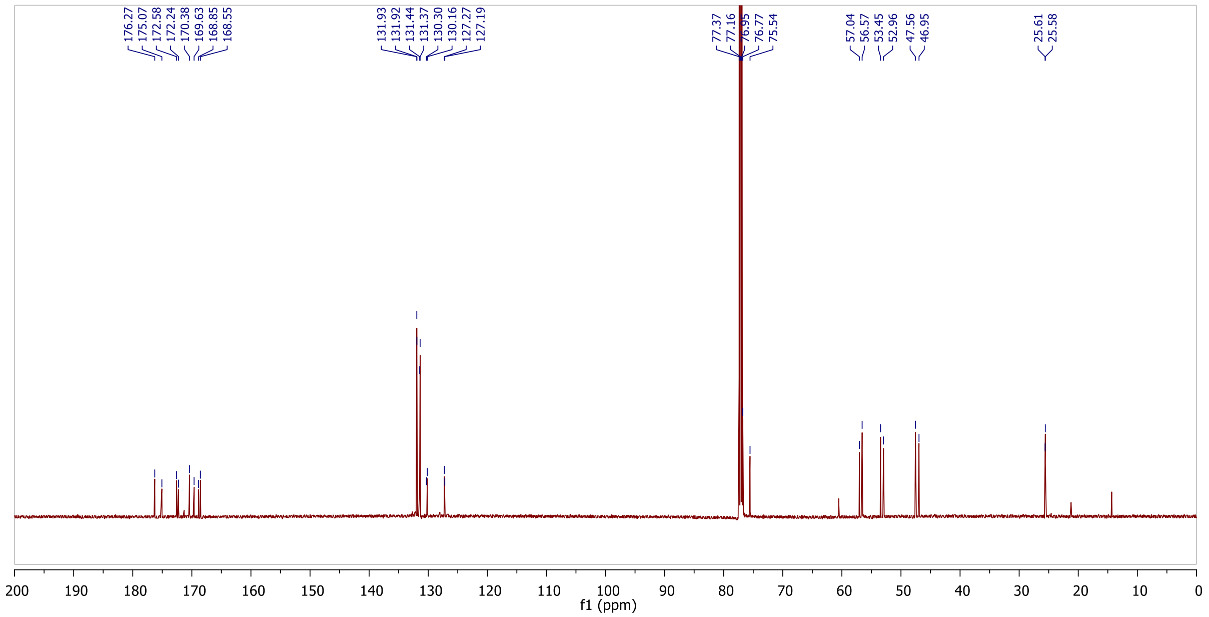
176.27
175.07
172.58
172.24
170.38
169.63
168.85
168.55
131.93
131.92
131.44
131.37
130.30
130.16
127.27
127.19
77.37
77.16
76.95
76.77
75.54
57.04
56.57
53.45
52.96
47.56
46.95
25.61
25.58
f1 (ppm)

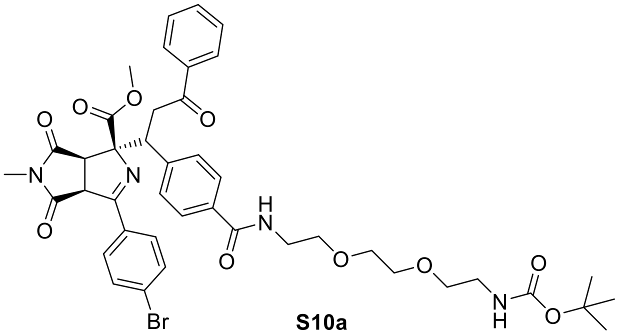

S10a

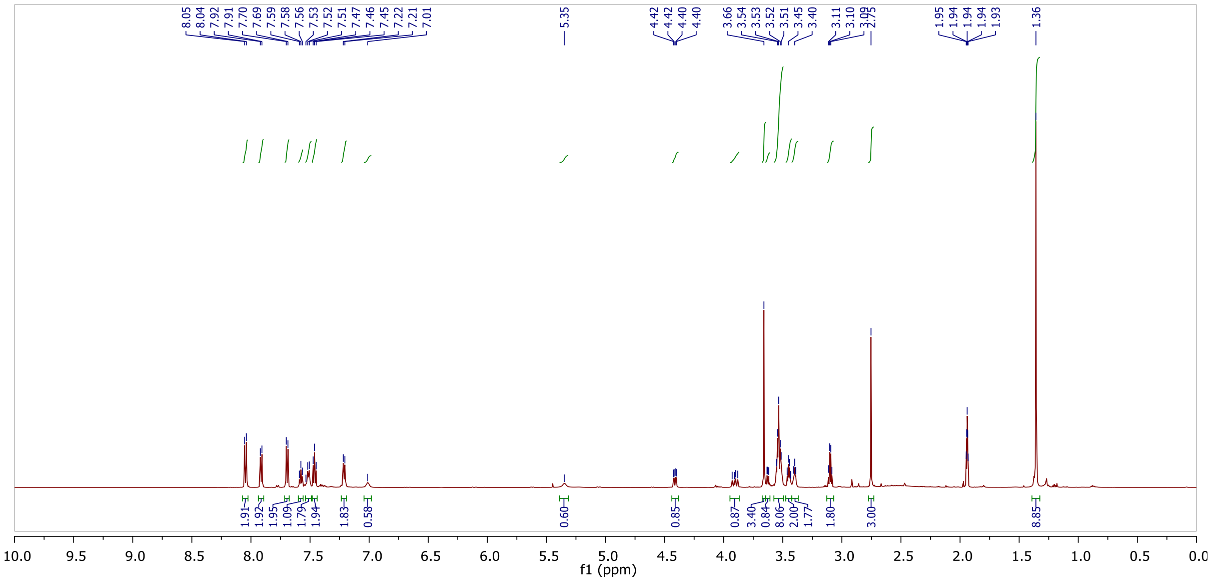

8.05
8.04
7.92
7.91
7.70
7.69
7.59
7.58
7.56
7.53
7.52
7.51
7.47
7.46
7.45
7.22
7.21
7.01
5.35
4.42
4.42
4.40
4.40
3.66
3.54
3.53
3.52
3.51
3.45
3.40
3.11
3.10
3.09
2.75
1.95
1.94
1.94
1.94
1.93
1.36
1.91
1.92
1.95
1.09
1.79
1.94
1.83
0.58
0.60
0.85
0.87
3.40
0.84
8.06
2.00
1.77
1.80
3.00
8.85
10.0
9.5
9.0
8.5
8.0
7.5
7.0
6.5
6.0
5.5
5.0
4.5
4.0
3.5
3.0
2.5
2.0
1.5
1.0
0.5
0.0
f1 (ppm)

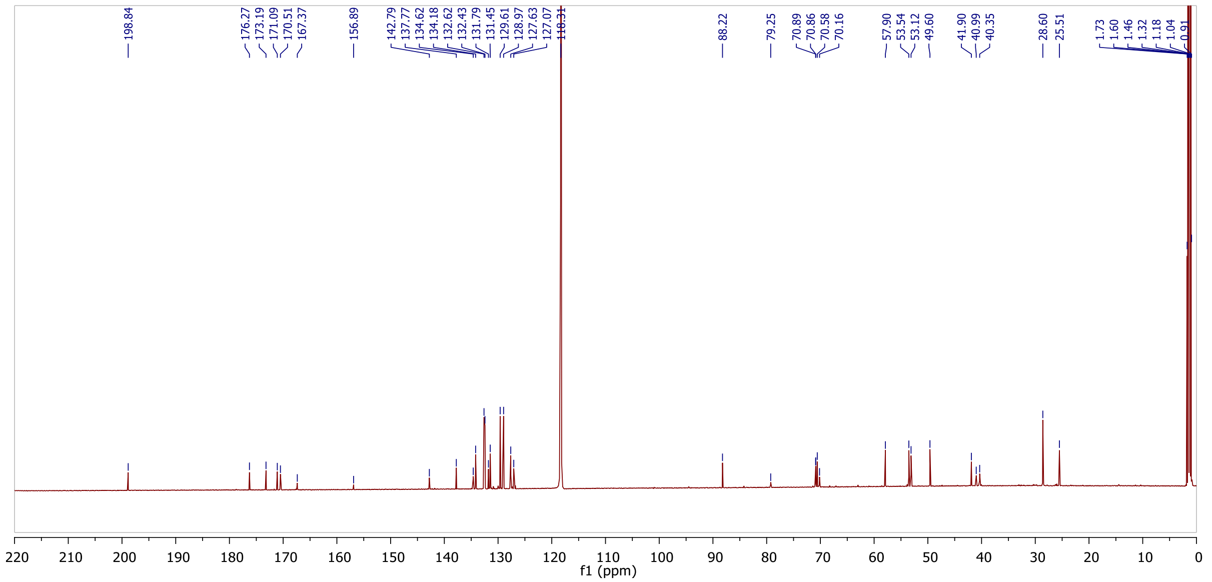

198.84
176.27
173.19
171.09
170.51
167.37
156.89
142.79
137.77
134.62
134.18
132.62
132.43
131.79
131.45
129.61
128.97
127.63
127.07
118.31
88.22
79.25
70.89
70.86
70.58
70.16
57.90
53.54
53.12
49.60
41.90
40.99
40.35
28.60
25.51
1.73
1.60
1.46
1.32
1.18
1.04
0.91
220
210
200
190
180
170
160
150
140
130
120
110
100
90
80
70
60
50
40
30
20
10
0
f1 (ppm)

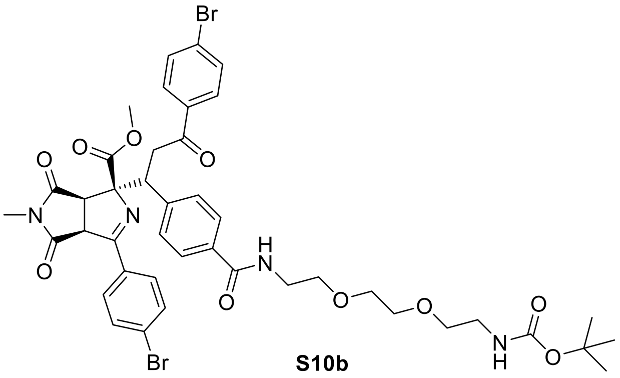

S10b

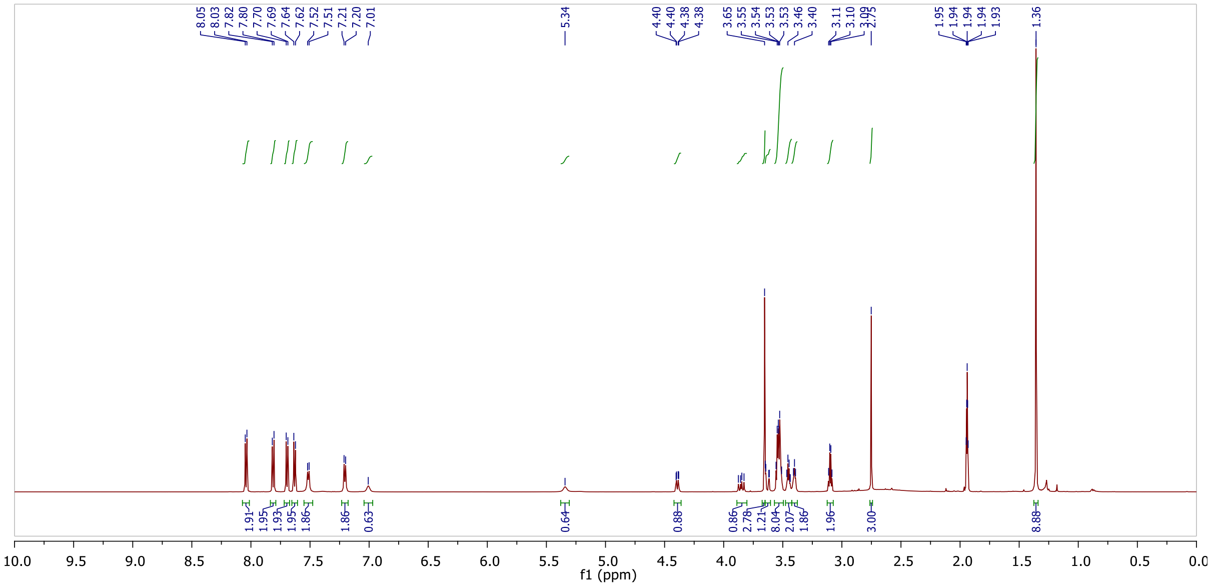

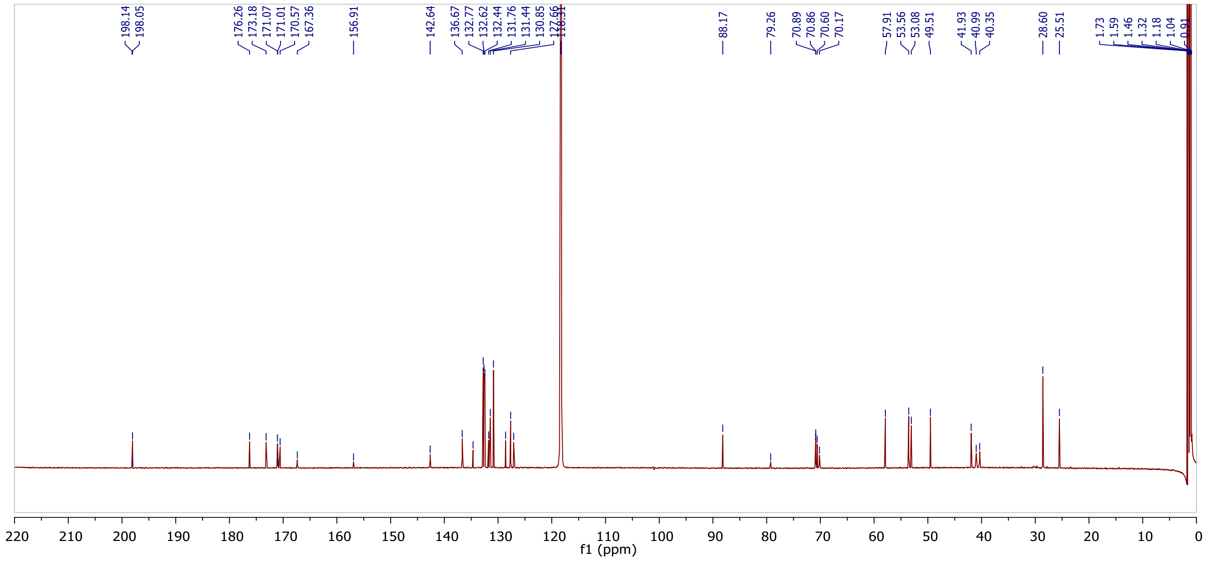

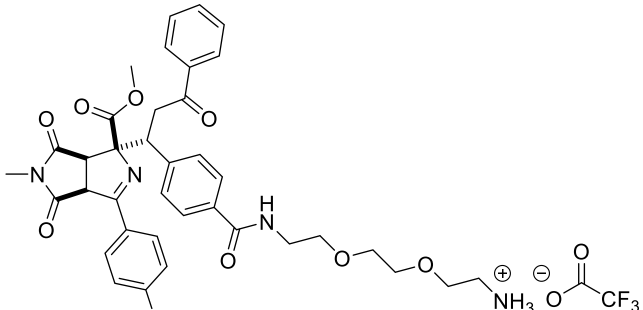

S11a

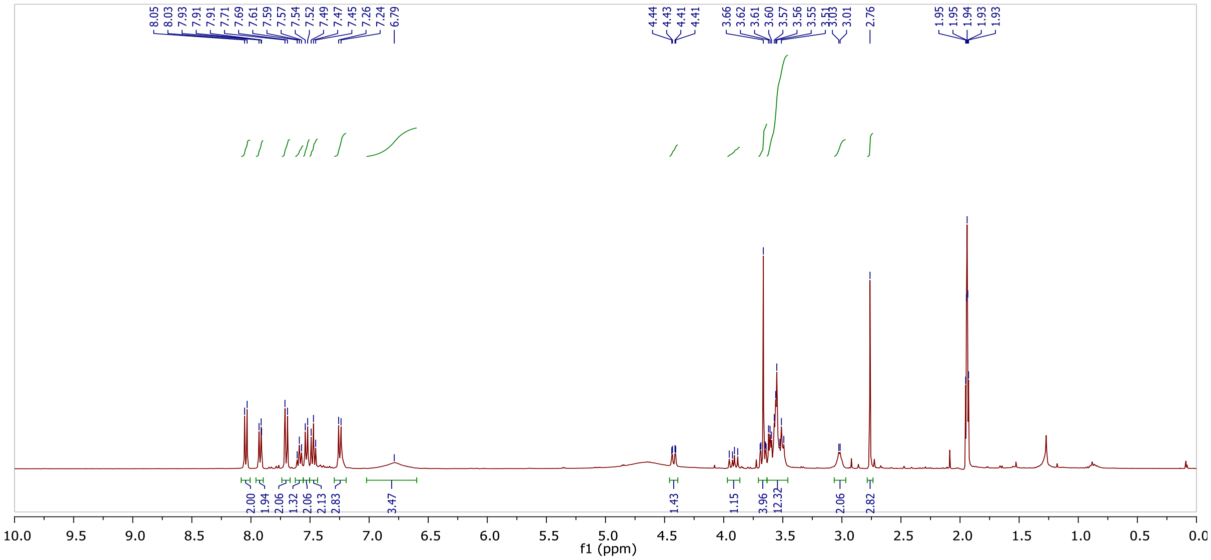

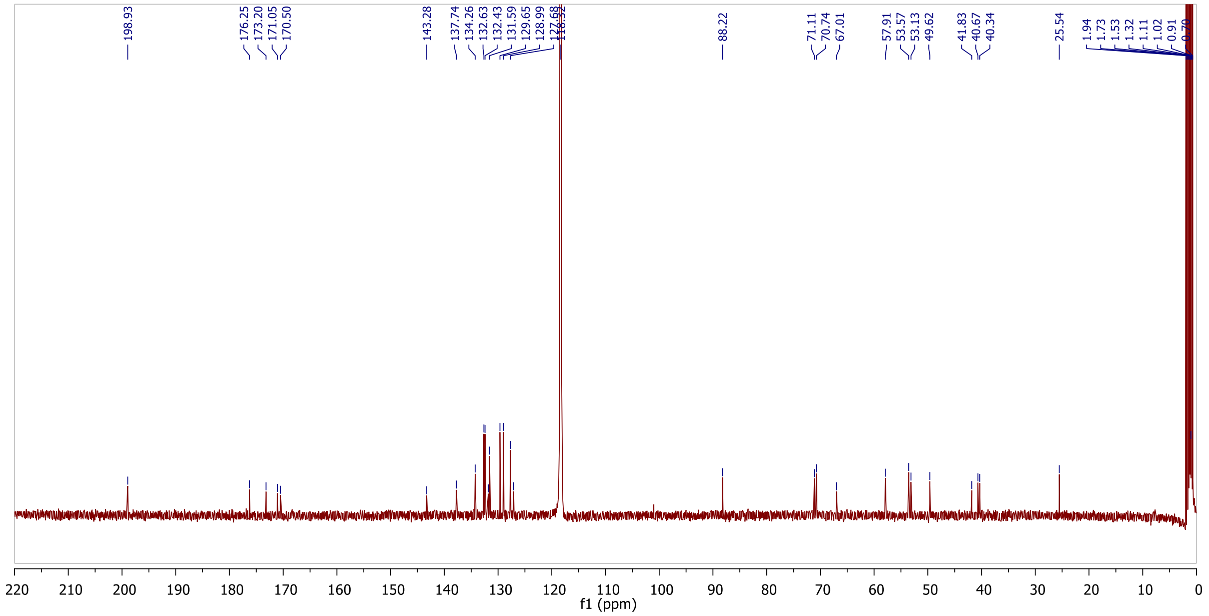

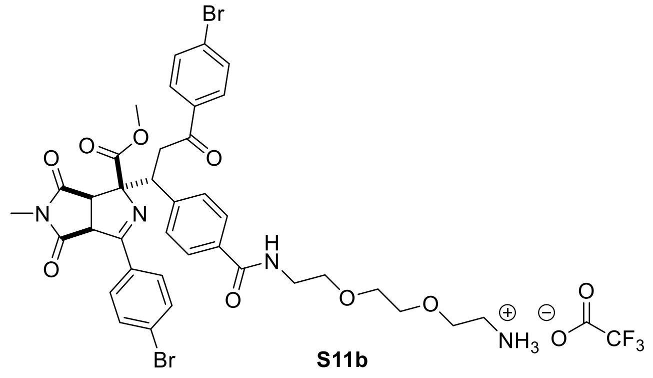
Br
O
O
O
N
N
O
H
N
O
O
O
NH3
O
O
CF3
Br

S11b

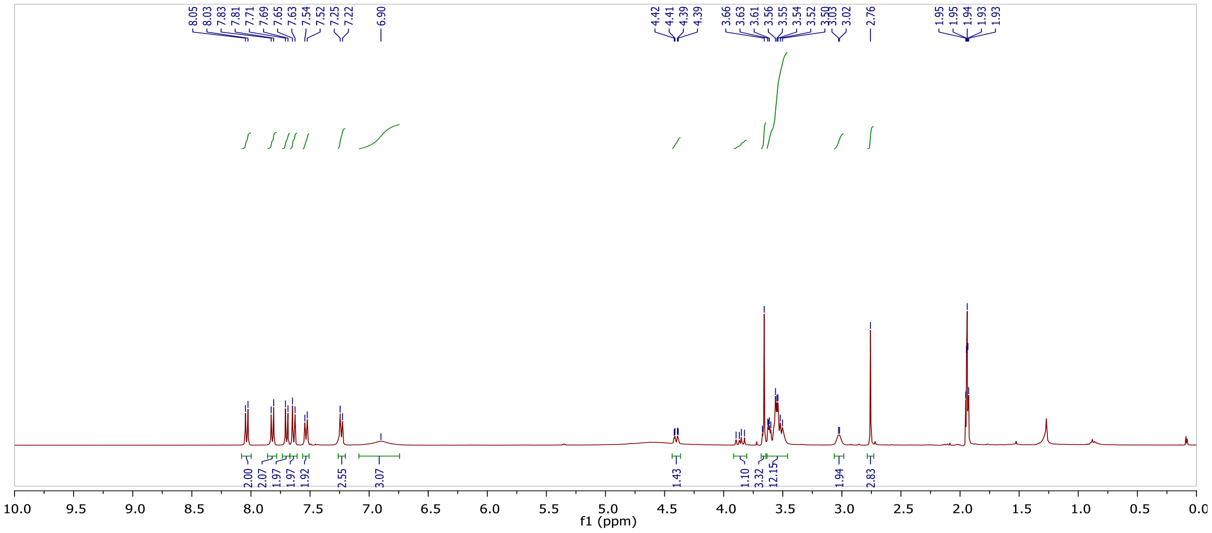
8.05 8.03 7.83 7.81 7.71 7.69 7.65 7.63 7.54 7.52 7.25 7.22
6.90
4.42 4.41 4.39 4.39
3.66 3.63 3.61 3.56 3.55 3.54 3.52 3.50 3.03 3.02
2.76
1.95 1.95 1.94 1.93 1.93
2.00 2.07 1.97 1.97 1.92 2.55 3.07
1.43
1.10 3.32 12.15
1.94 2.83
10.0 9.5 9.0 8.5 8.0 7.5 7.0 6.5 6.0 5.5 5.0 4.5 4.0 3.5 3.0 2.5 2.0 1.5 1.0 0.5 0.0
f1 (ppm)

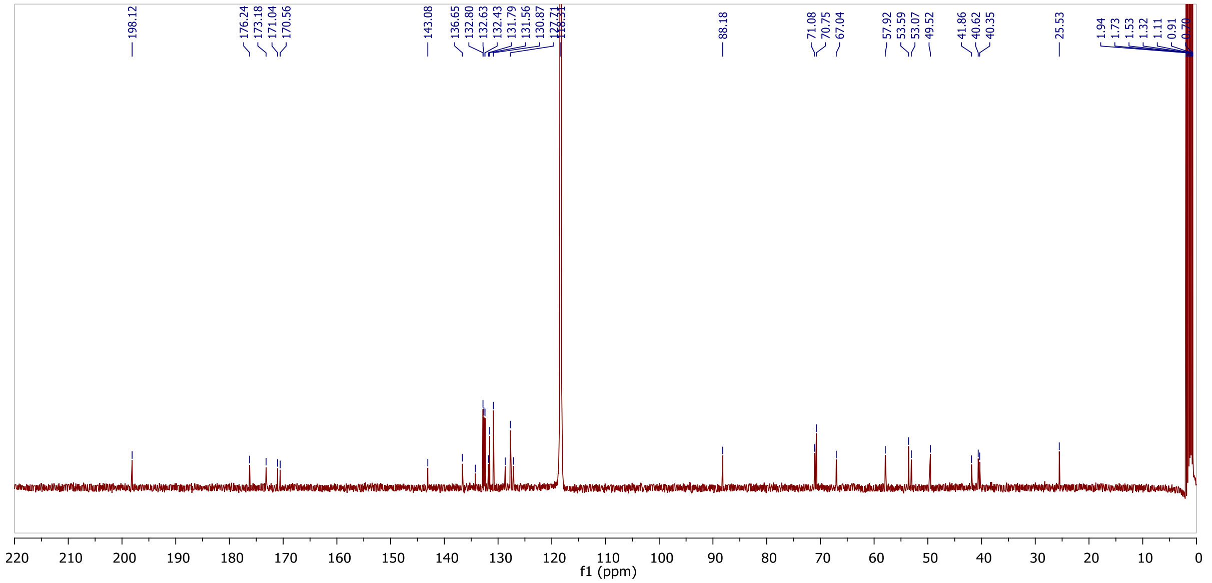
198.12
176.24 173.18 171.04 170.56
143.08
136.65 132.80 132.63 132.43 131.79 131.56 130.87 127.71 118.31
88.18
71.08 70.75 67.04
57.92 53.59 53.07 49.52
41.86 40.62 40.35
25.53
1.94 1.73 1.53 1.32 1.11 0.91 0.70
220 210 200 190 180 170 160 150 140 130 120 110 100 90 80 70 60 50 40 30 20 10 0
f1 (ppm)

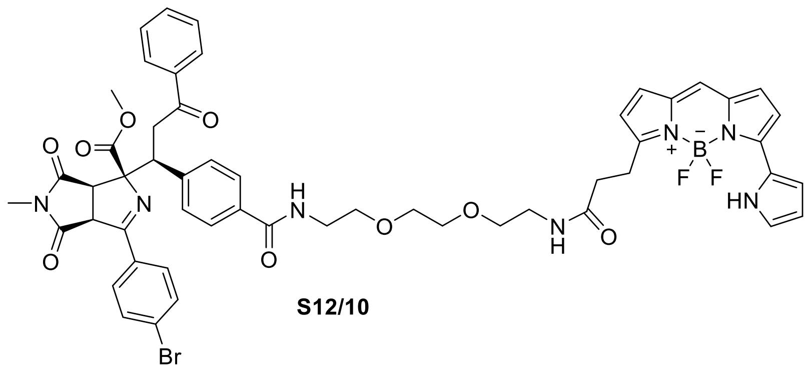

S12/10

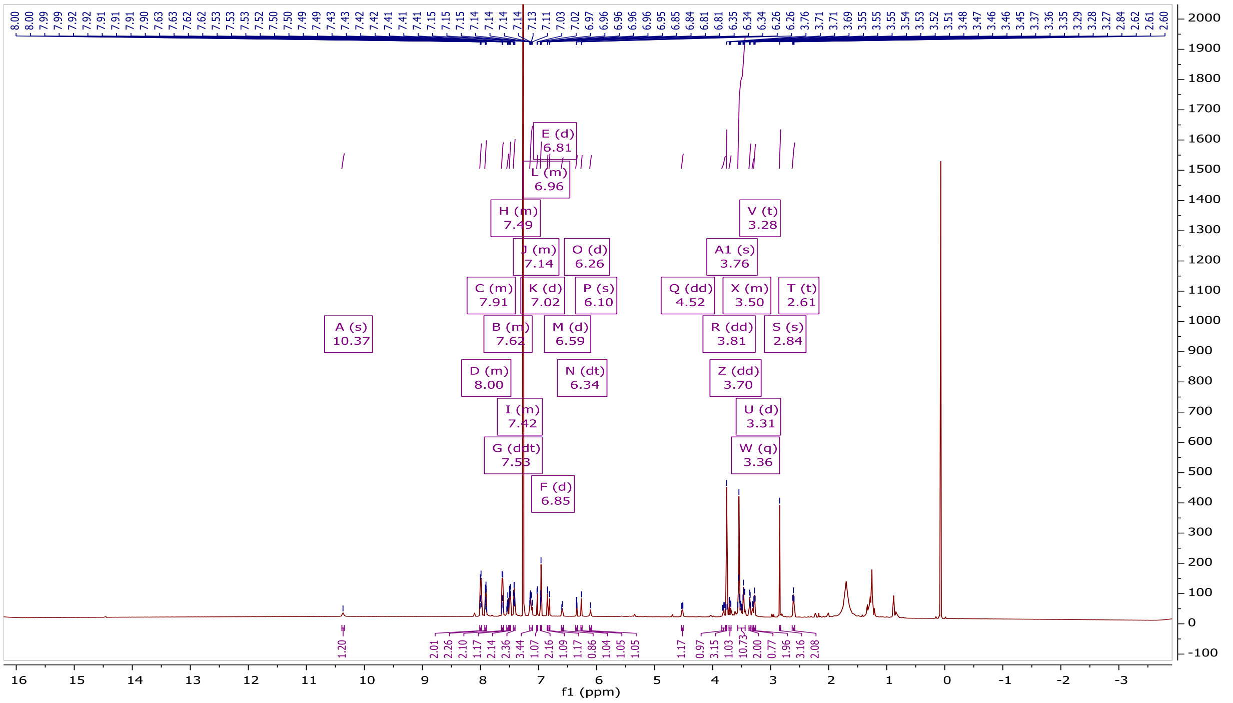
A (s) 10.37
B (m) 7.62
C (m) 7.91
D (m) 8.00
E (d) 6.81
F (d) 6.85
G (ddt) 7.53
H (m) 7.49
I (m) 7.42
J (m) 7.14
K (d) 7.02
L (m) 6.96
M (d) 6.59
N (dt) 6.34
O (d) 6.26
P (s) 6.10
Q (dd) 4.52
R (dd) 3.81
S (s) 2.84
T (t) 2.61
U (d) 3.31
V (t) 3.28
W (q) 3.36
X (m) 3.50
Z (dd) 3.70
A1 (s) 3.76
f1 (ppm)

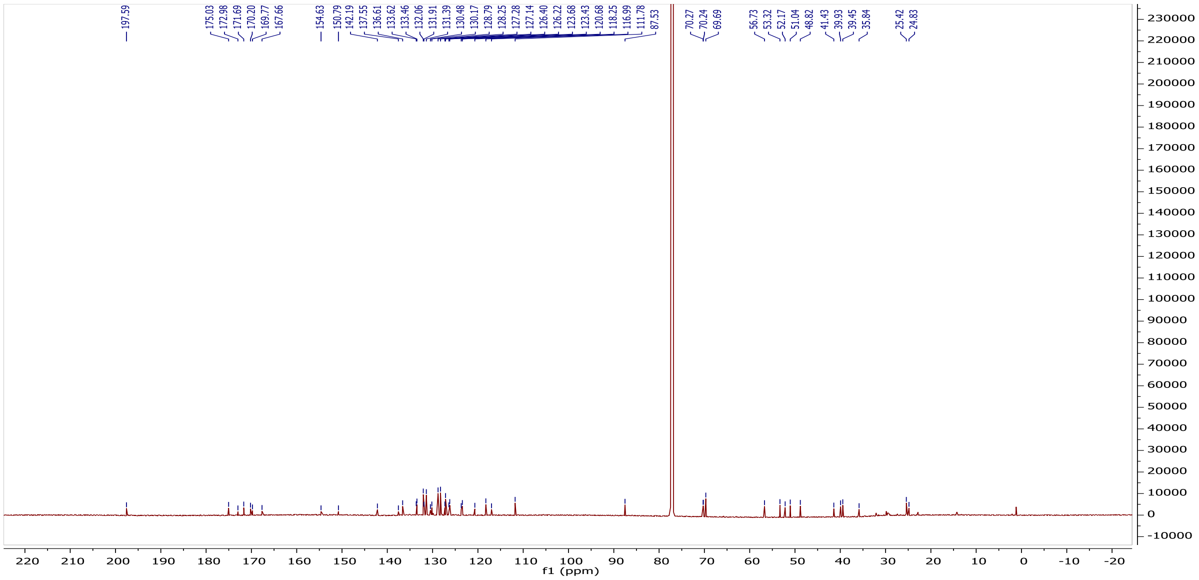

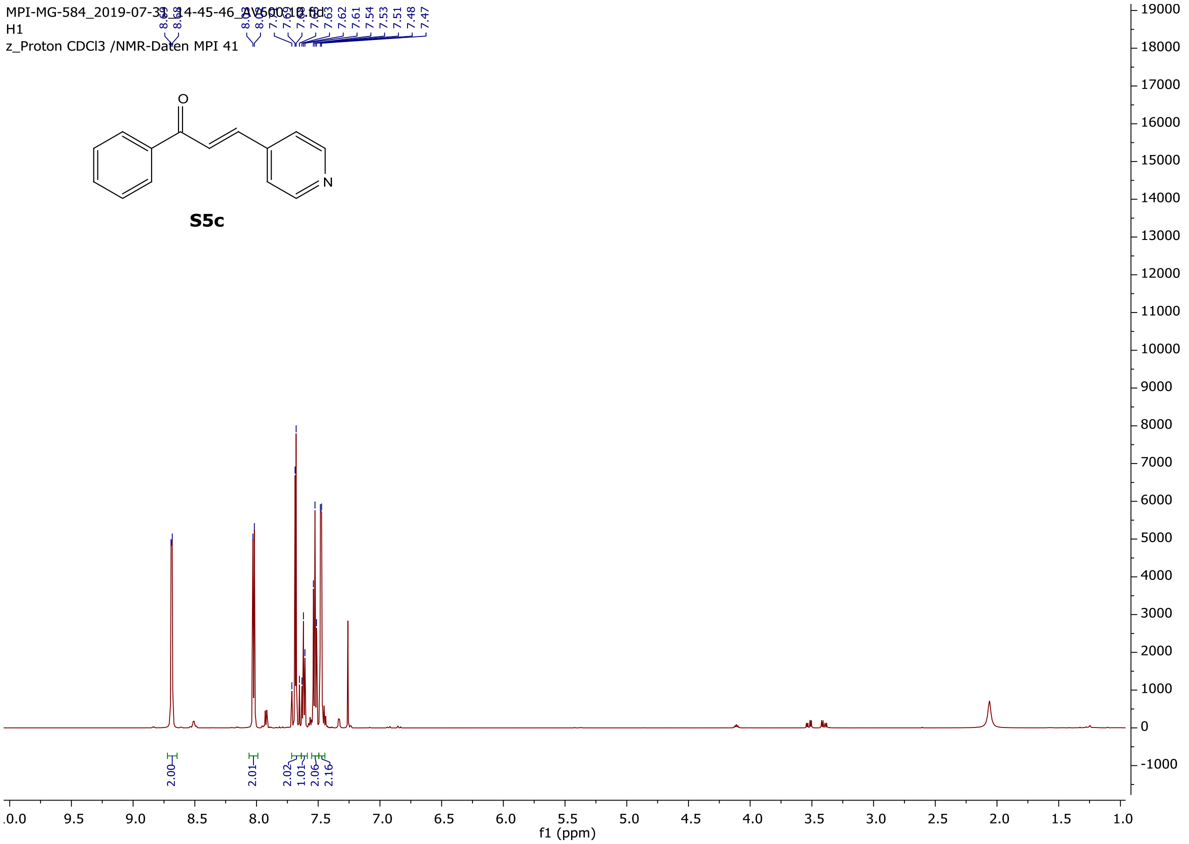
MPI-MG-584_2019-07-31_14-45-46_AV600.10.fid
H1
z_Proton CDCl3 /NMR-Daten MPI 41
7.63
7.62
7.61
7.54
7.53
7.51
7.48
7.47
S5c
2.00
2.01
2.02
1.01
2.06
2.16
f1 (ppm)

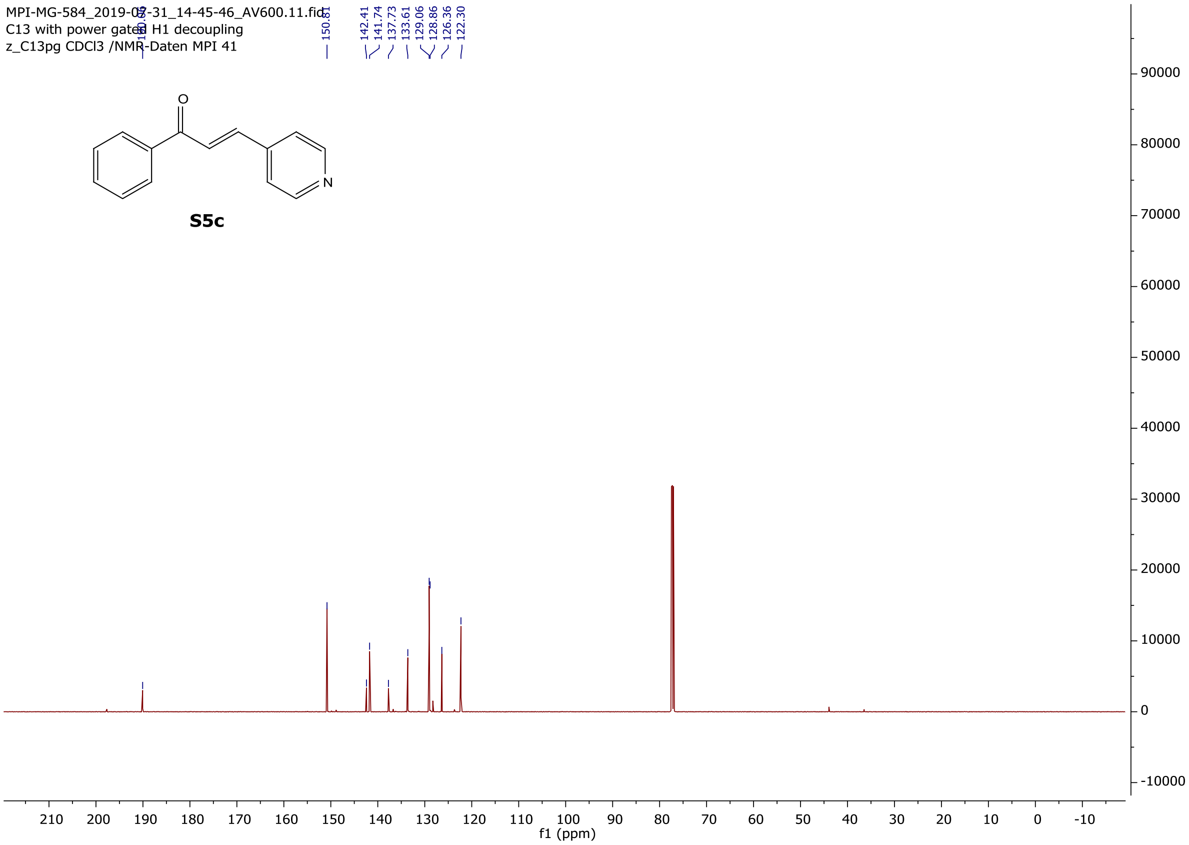
MPI-MG-584_2019-07-31_14-45-46_AV600.11.fid
C13 with power gated H1 decoupling
z_C13pg CDCl3 /NMR-Daten MPI 41
150.81
142.41
141.74
137.73
133.61
129.06
128.86
126.36
122.30
S5c
f1 (ppm)

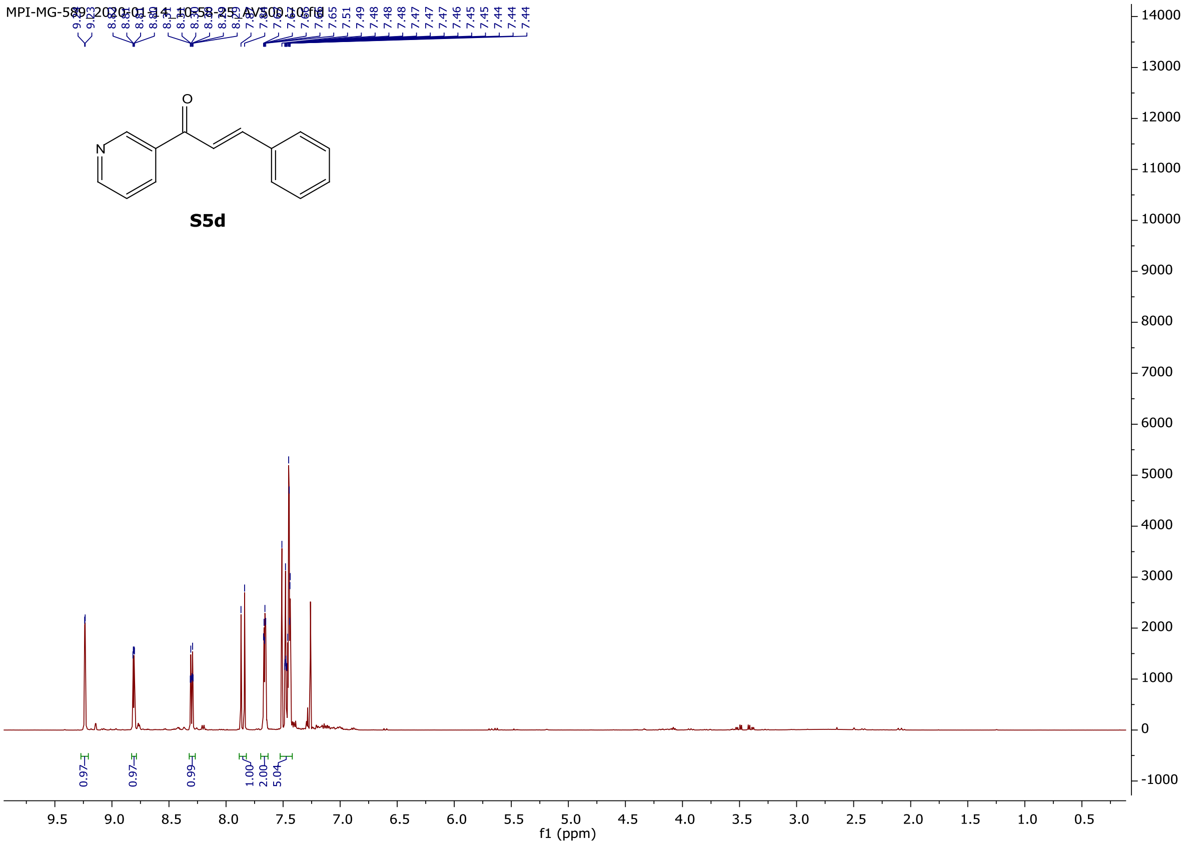

S5d
f1 (ppm)

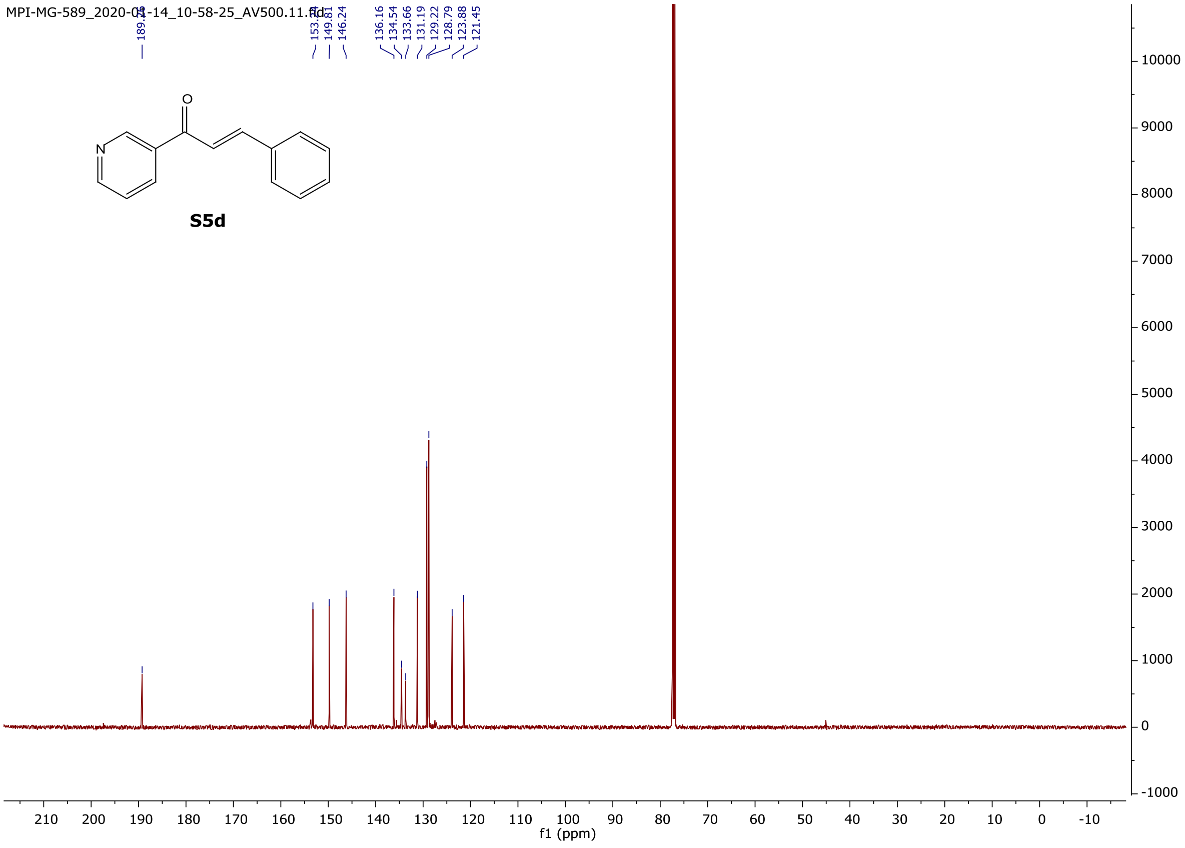

MPI-MG-589_2020-01-14_10-58-25_AV500.11.fid
153.16
149.81
146.24
136.16
134.54
133.66
131.19
129.22
128.79
123.88
121.45
S5d
f1 (ppm)

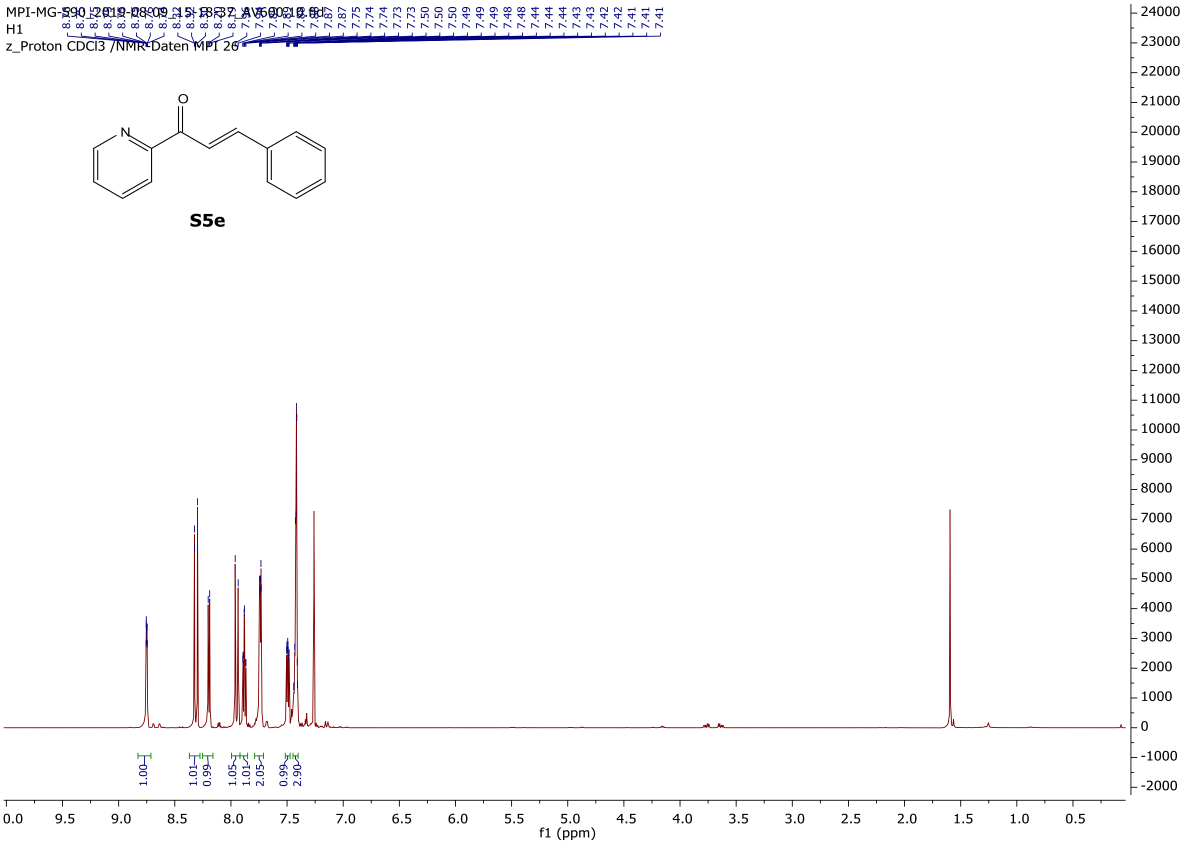
H1
z_Proton CDCl3 /NMR-Daten MPI 26
S5e
1.00
1.01
0.99
1.05
1.01
2.05
0.99
2.90
f1 (ppm)

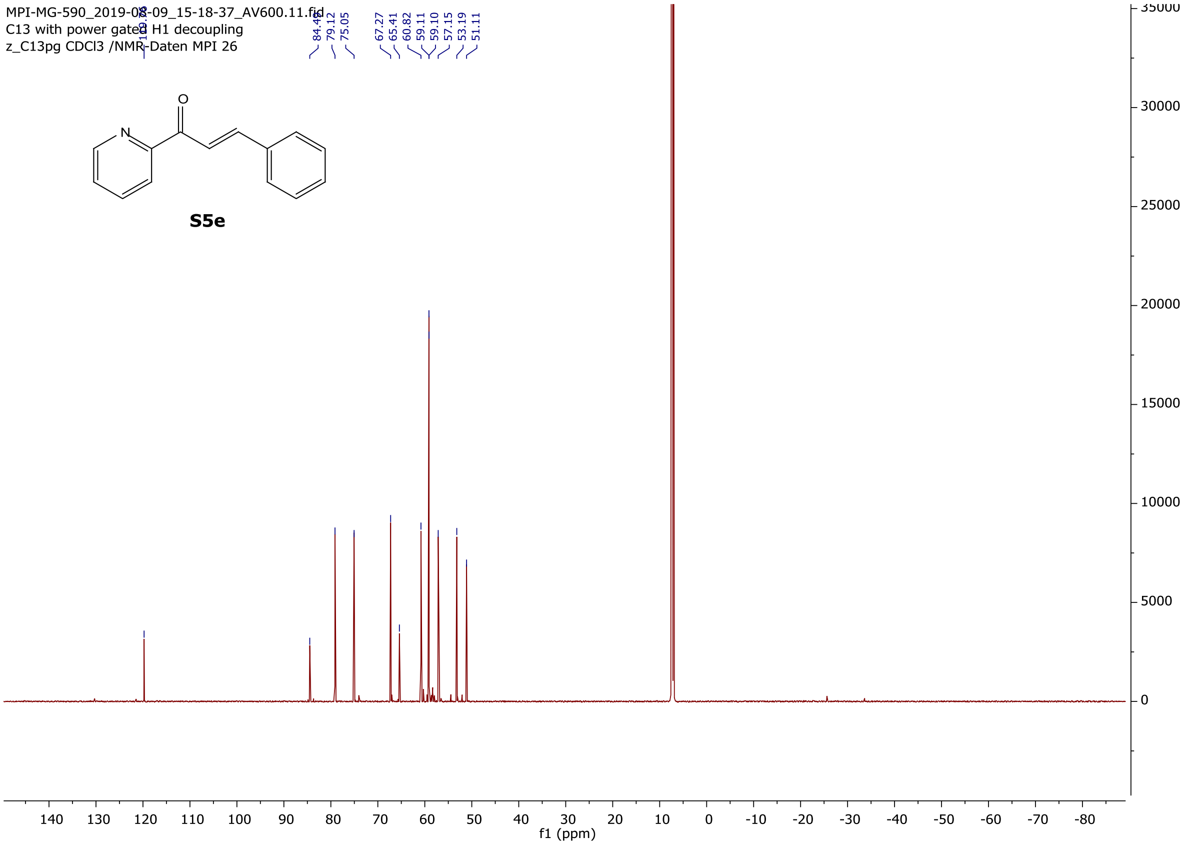
MPI-MG-590_2019-08-09_15-18-37_AV600.11.fid
C13 with power gated H1 decoupling
z_C13pg CDCl3 /NMR-Daten MPI 26
79.12
75.05
67.27
65.41
60.82
59.11
59.10
57.15
53.19
51.11
S5e
f1 (ppm)

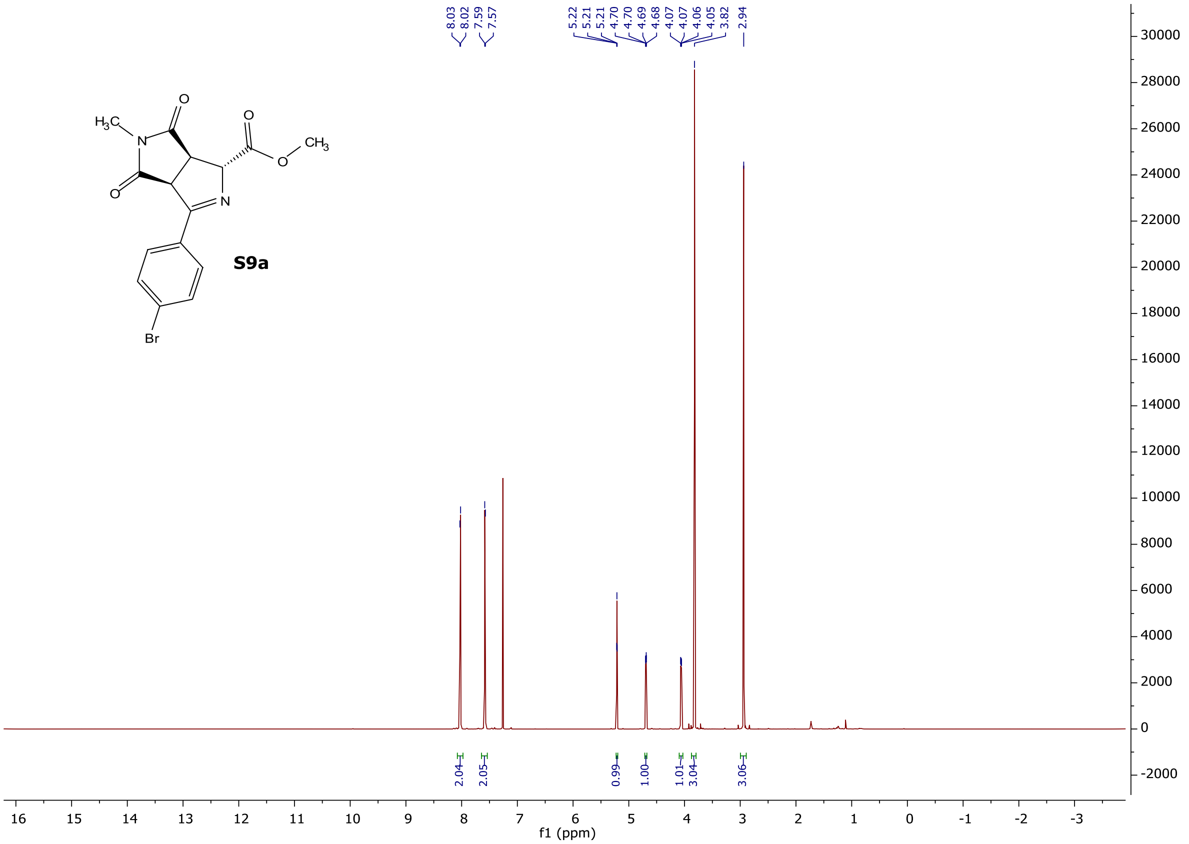

S9a
8.03
8.02
7.59
7.57
5.22
5.21
5.21
4.70
4.70
4.69
4.68
4.07
4.07
4.06
4.05
3.82
2.94
2.04
2.05
0.99
1.00
1.01
3.04
3.06
f1 (ppm)

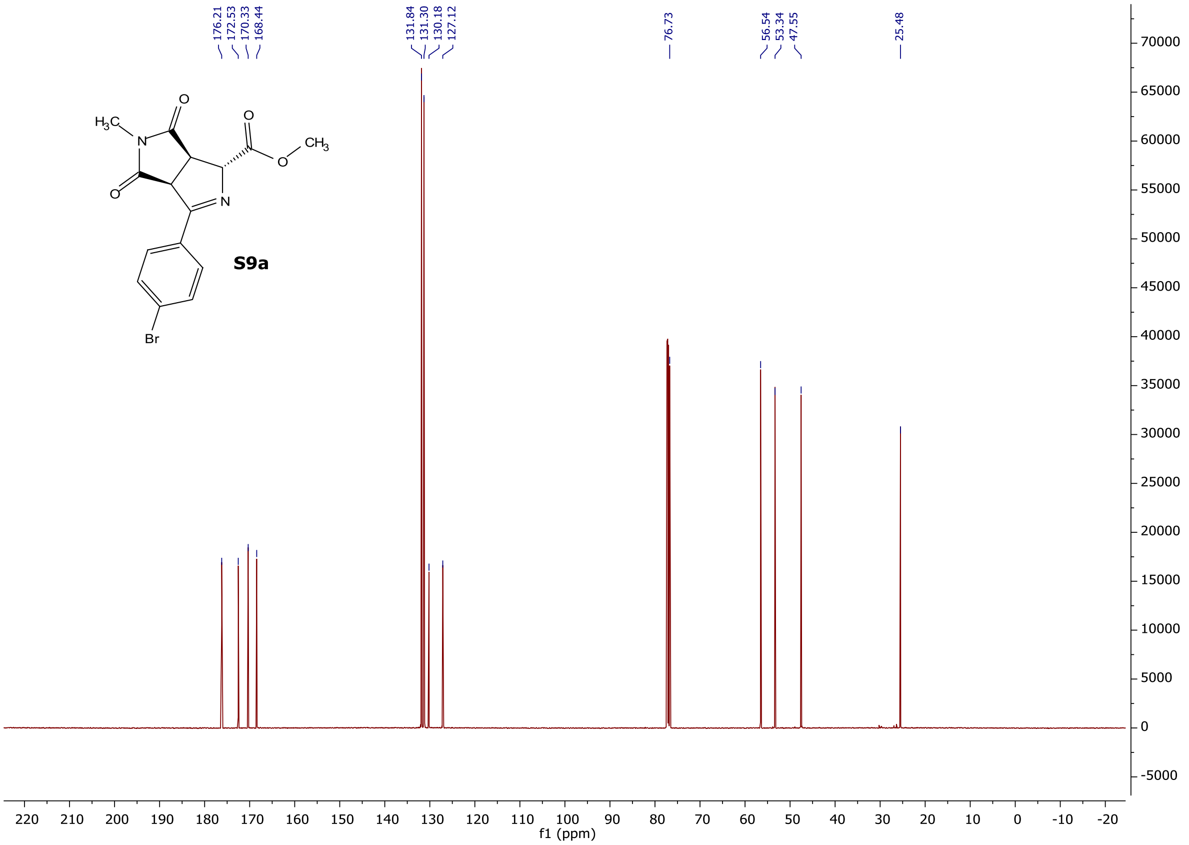

S9a
176.21
172.53
170.33
168.44
131.84
131.30
130.18
127.12
76.73
56.54
53.34
47.55
25.48
f1 (ppm)

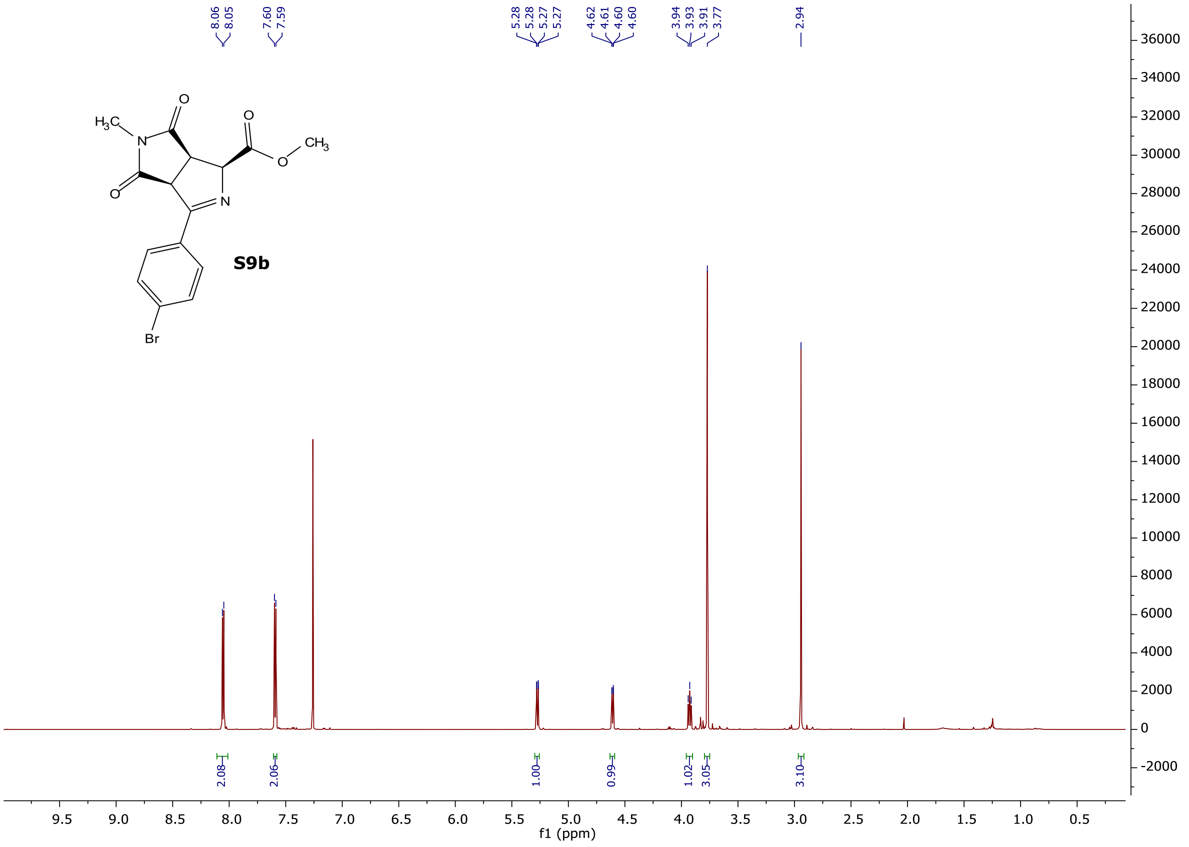

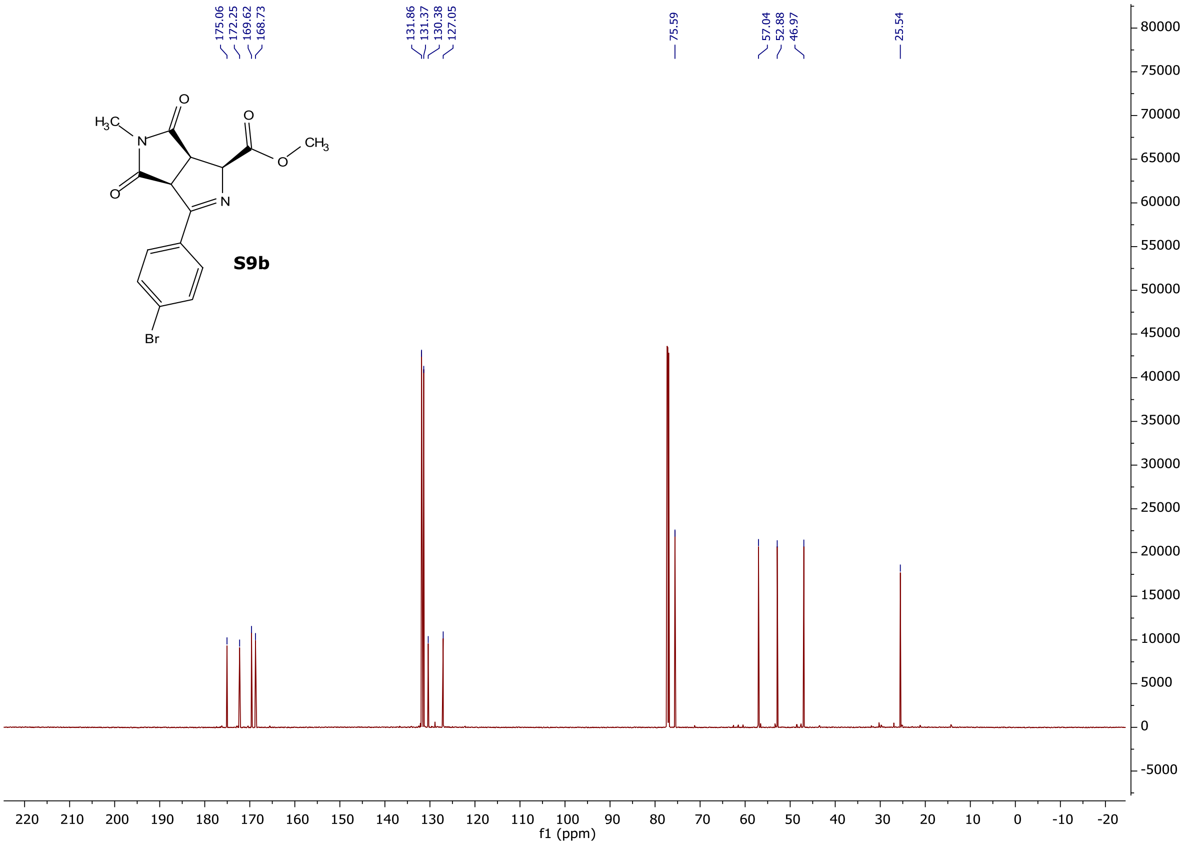

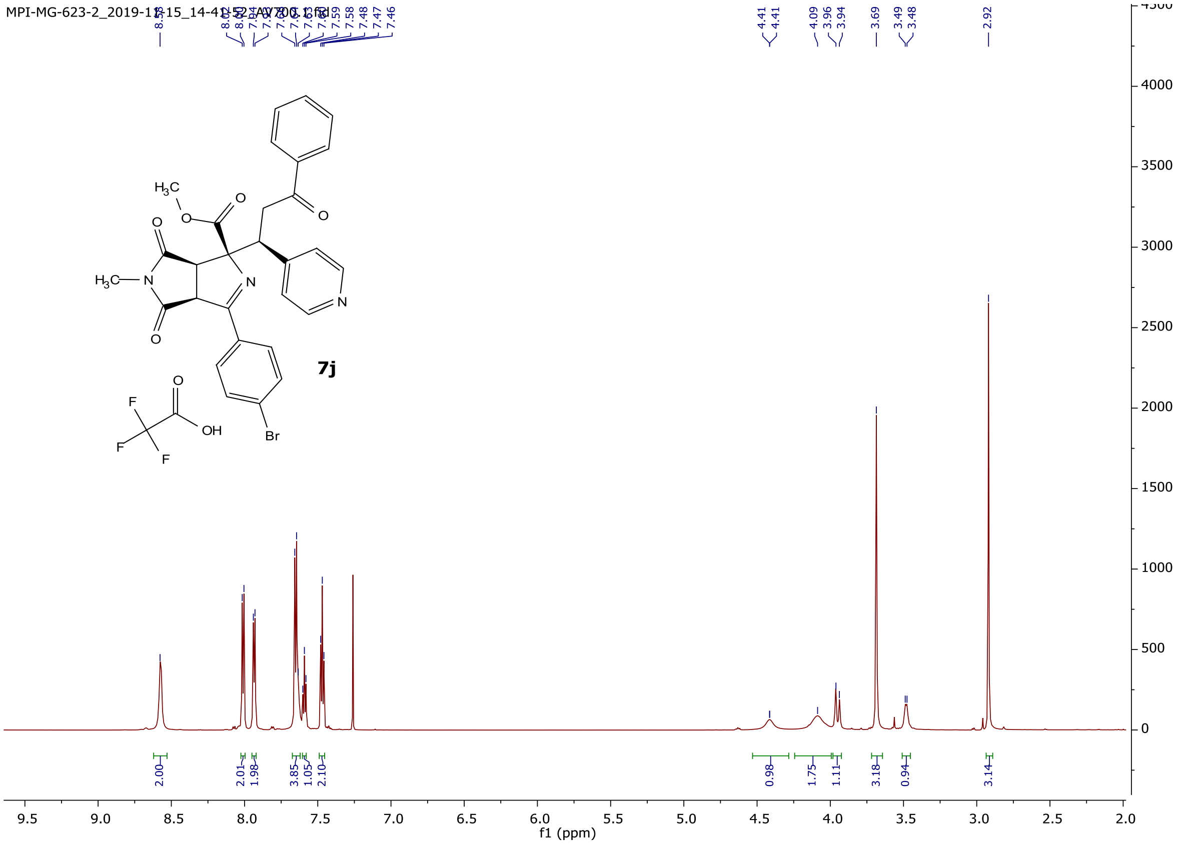
4.41
4.41
4.09
3.96
3.94
3.69
3.49
3.48
2.92
7.59
7.58
7.48
7.47
7.46
7j
Br
OH
2.00
2.01
1.98
3.85
1.05
2.10
0.98
1.75
1.11
3.18
0.94
3.14
f1 (ppm)

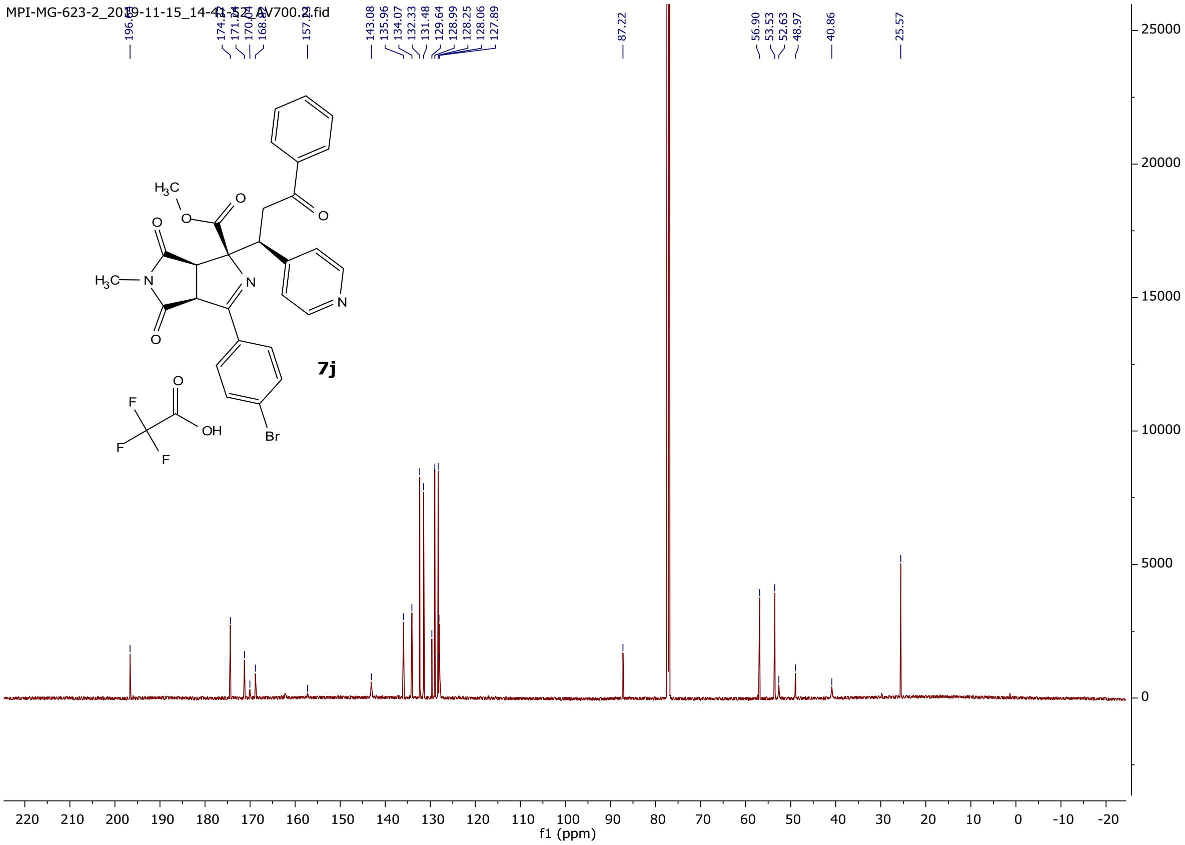
143.08
135.96
134.07
132.33
131.48
129.64
128.99
128.25
128.06
127.89
87.22
56.90
53.53
52.63
48.97
40.86
25.57
7j
Br
OH
f1 (ppm)

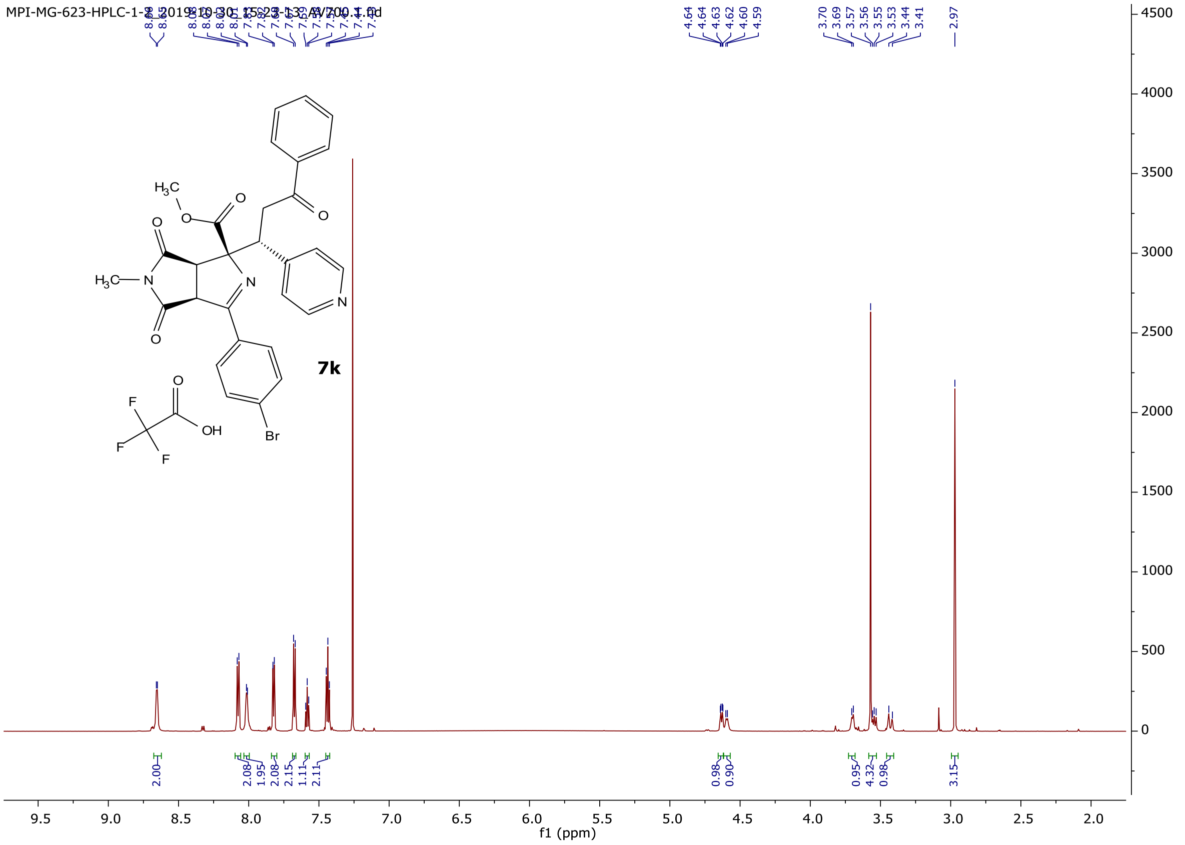
MPI-MG-623-HPLC-1-2_2019-10-30_15-23-13_AV700.1.fid
7k
f1 (ppm)

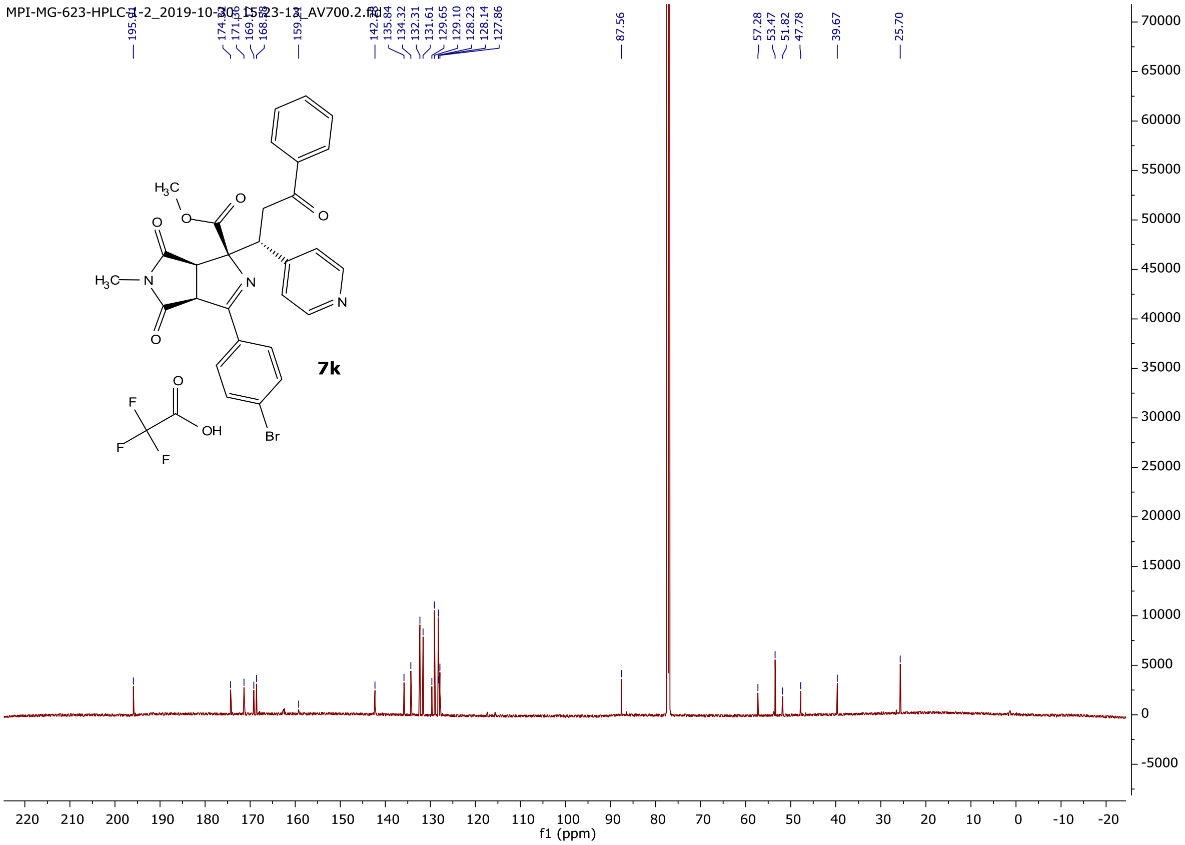
MPI-MG-623-HPLC-1-2_2019-10-30_15-23-13_AV700.2.fid
7k
f1 (ppm)

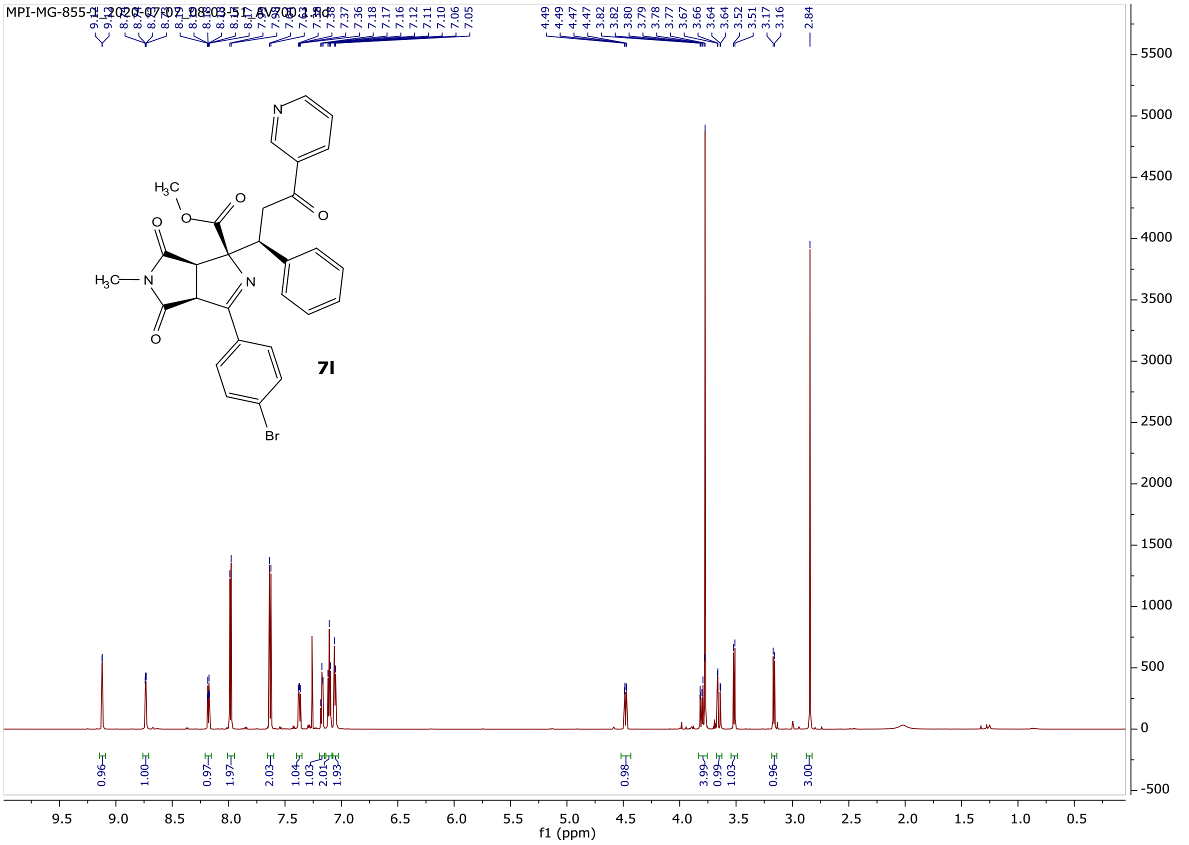
7.37
7.36
7.18
7.17
7.16
7.12
7.11
7.10
7.06
7.05
4.49
4.49
4.47
4.47
3.82
3.82
3.80
3.79
3.78
3.77
3.67
3.66
3.64
3.64
3.52
3.51
3.17
3.16
2.84
0.96
1.00
0.97
1.97
2.03
1.04
1.03
2.01
1.93
0.98
3.99
0.99
1.03
0.96
3.00
7l
f1 (ppm)

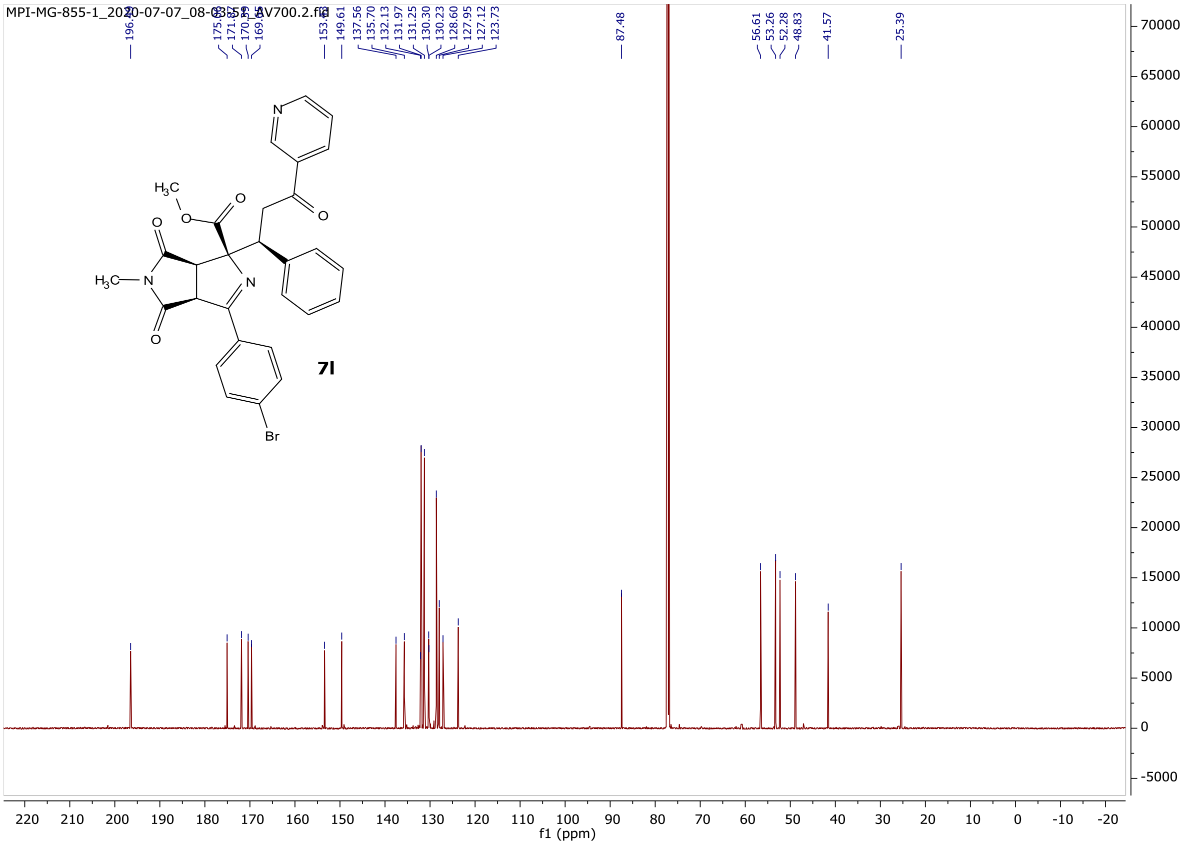
149.61
137.56
135.70
132.13
131.97
131.25
130.30
130.23
128.60
127.95
127.12
123.73
87.48
56.61
53.26
52.28
48.83
41.57
25.39
7l
f1 (ppm)

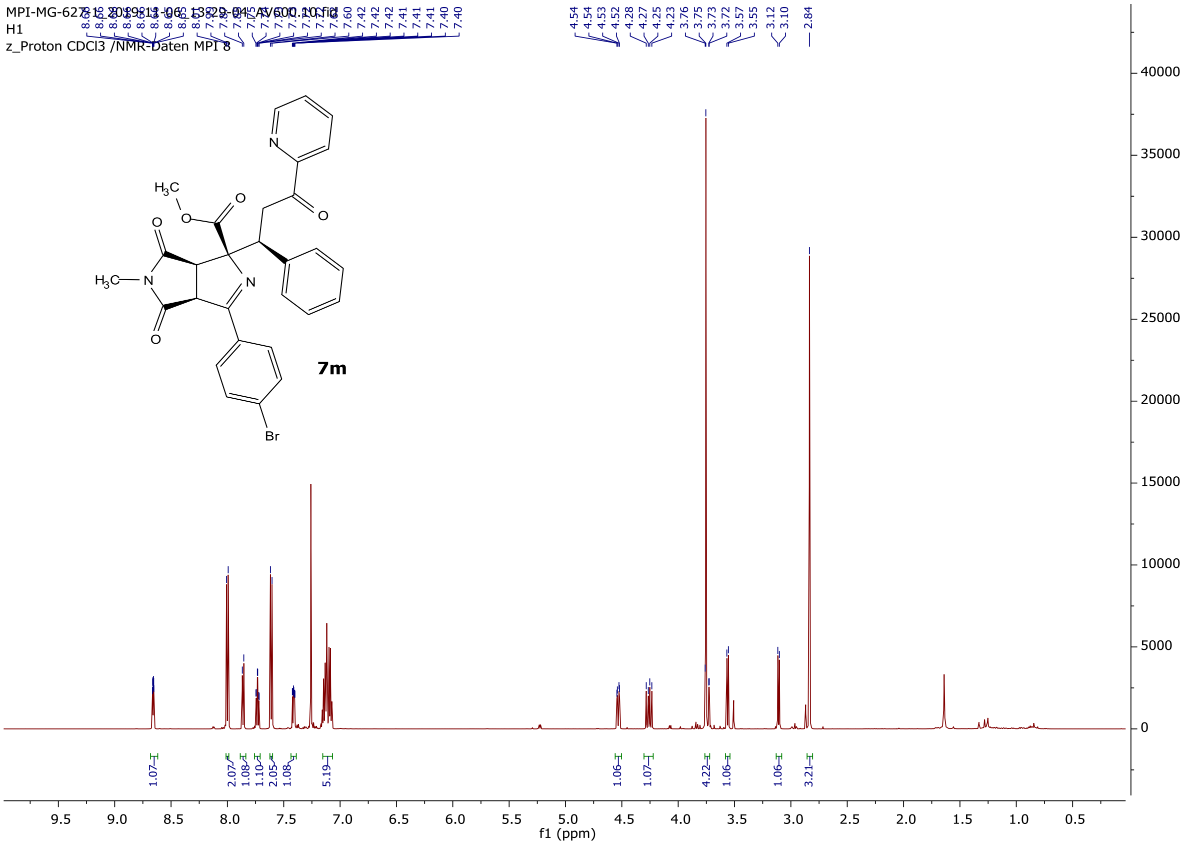
H1
z_Proton CDCl3 /NMR-Daten MPI 8
7.60
7.42
7.42
7.42
7.41
7.41
7.40
7.40
4.54
4.54
4.53
4.52
4.28
4.27
4.25
4.23
3.76
3.75
3.73
3.72
3.57
3.55
3.12
3.10
2.84
H_3C
O
O
O
N
H_3C
N
N
O
O
7m
Br
1.07
2.07
1.08
1.10
2.05
1.08
5.19
1.06
1.07
4.22
1.06
1.06
3.21
f1 (ppm)

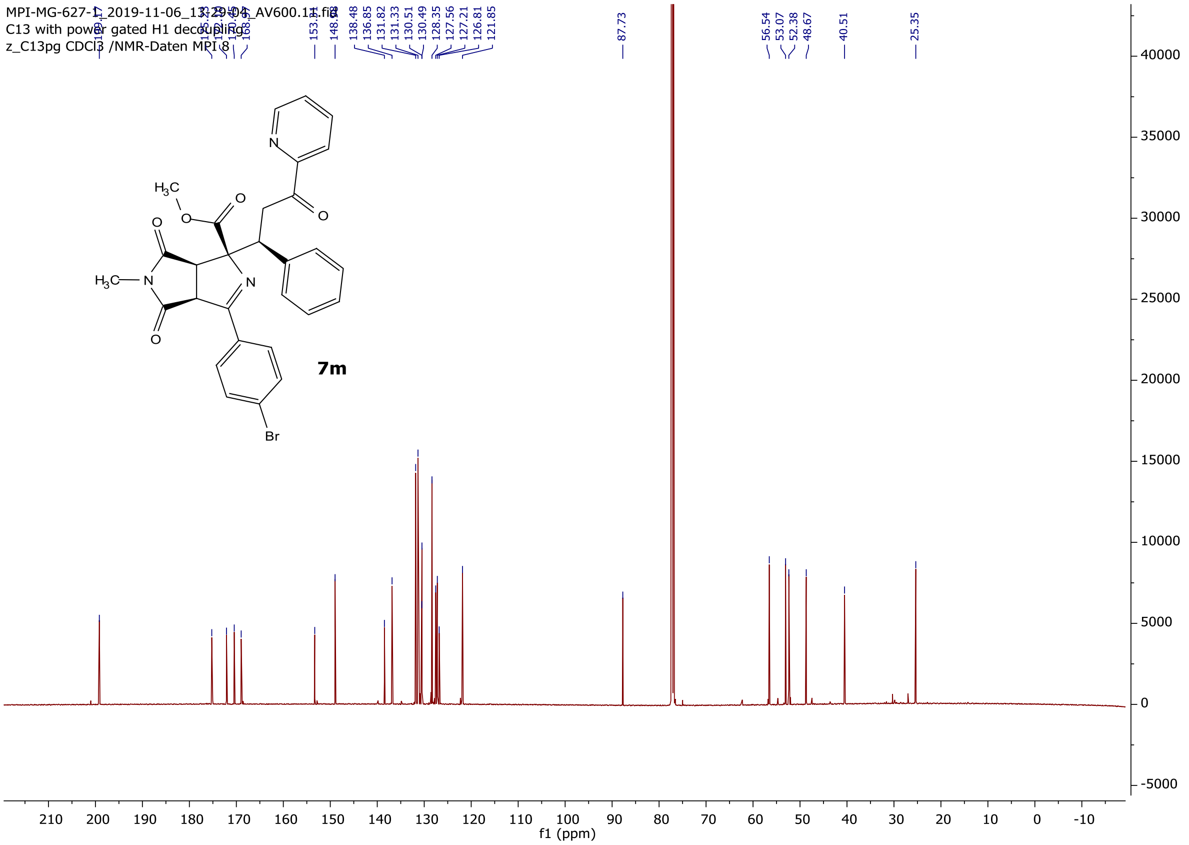
C13 with power gated H1 decoupling
z_C13pg CDCl3 /NMR-Daten MPI 8
138.48
136.85
131.82
131.33
130.51
130.49
128.35
127.56
127.21
126.81
121.85
87.73
56.54
53.07
52.38
48.67
40.51
25.35
H_3C
O
O
O
N
H_3C
N
N
O
O
7m
Br
f1 (ppm)

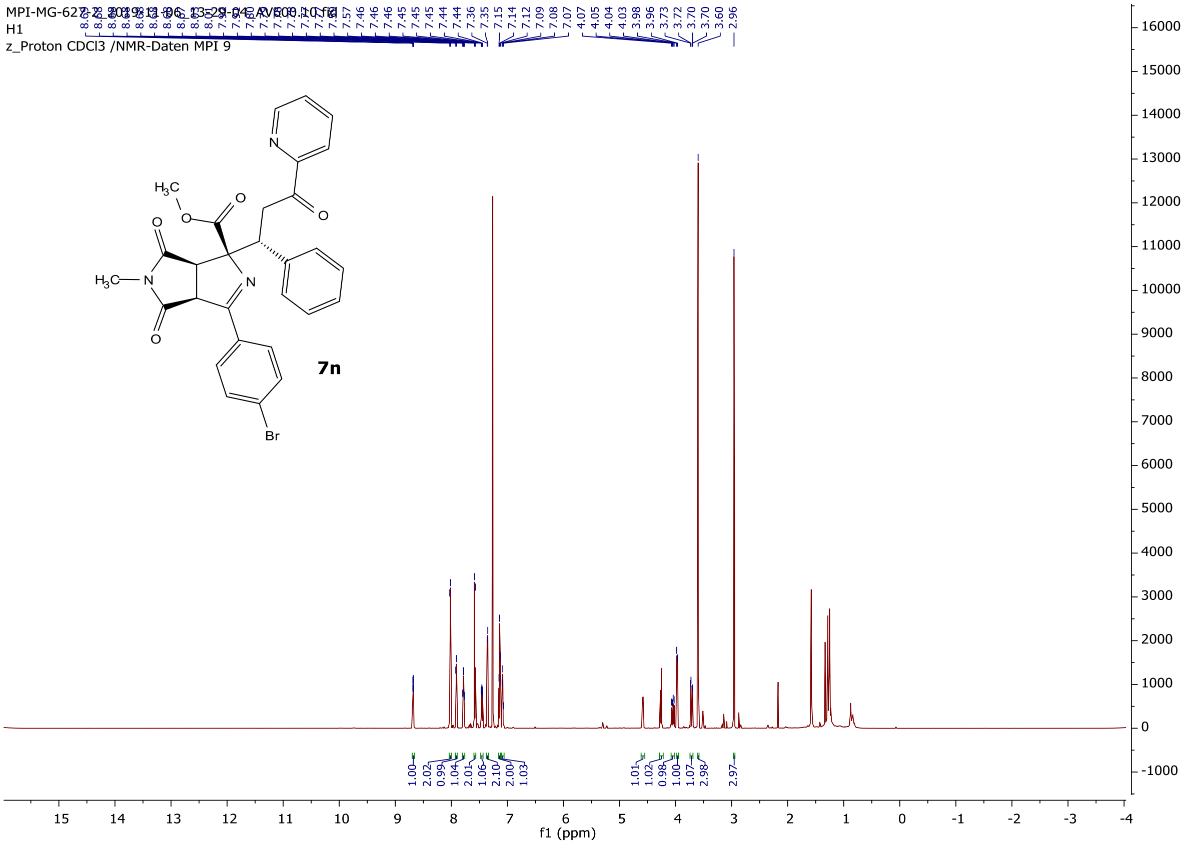
MPI-MG-627-2_2019-11-06_13-29-04_AV600.10.fid
H1
z_Proton CDCl3 /NMR-Daten MPI 9
7n
f1 (ppm)

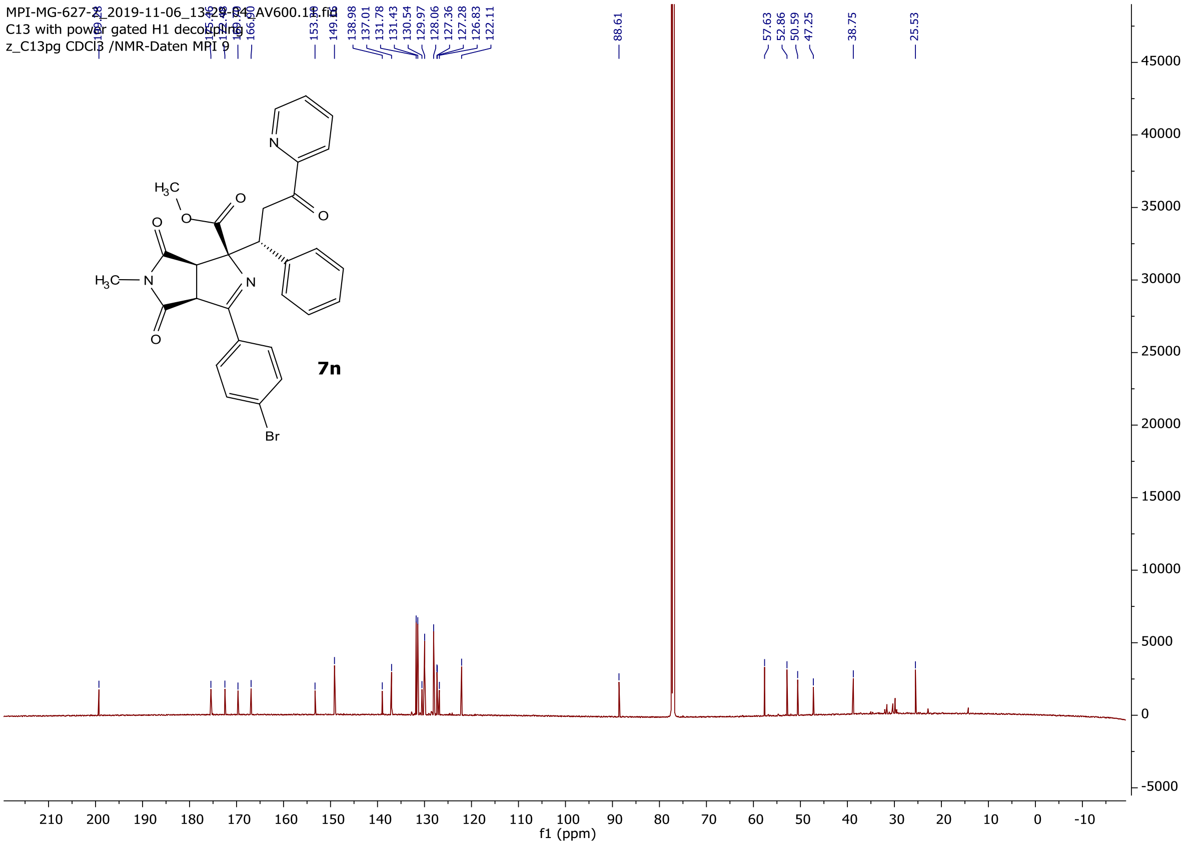
MPI-MG-627-2_2019-11-06_13-29-04_AV600.11.fid
C13 with power gated H1 decoupling
z_C13pg CDCl3 /NMR-Daten MPI 9
7n
f1 (ppm)

HPLC-MS trace of 7l:

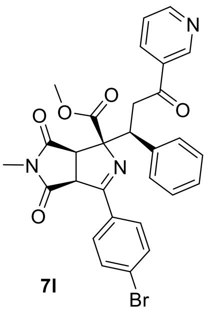

7l

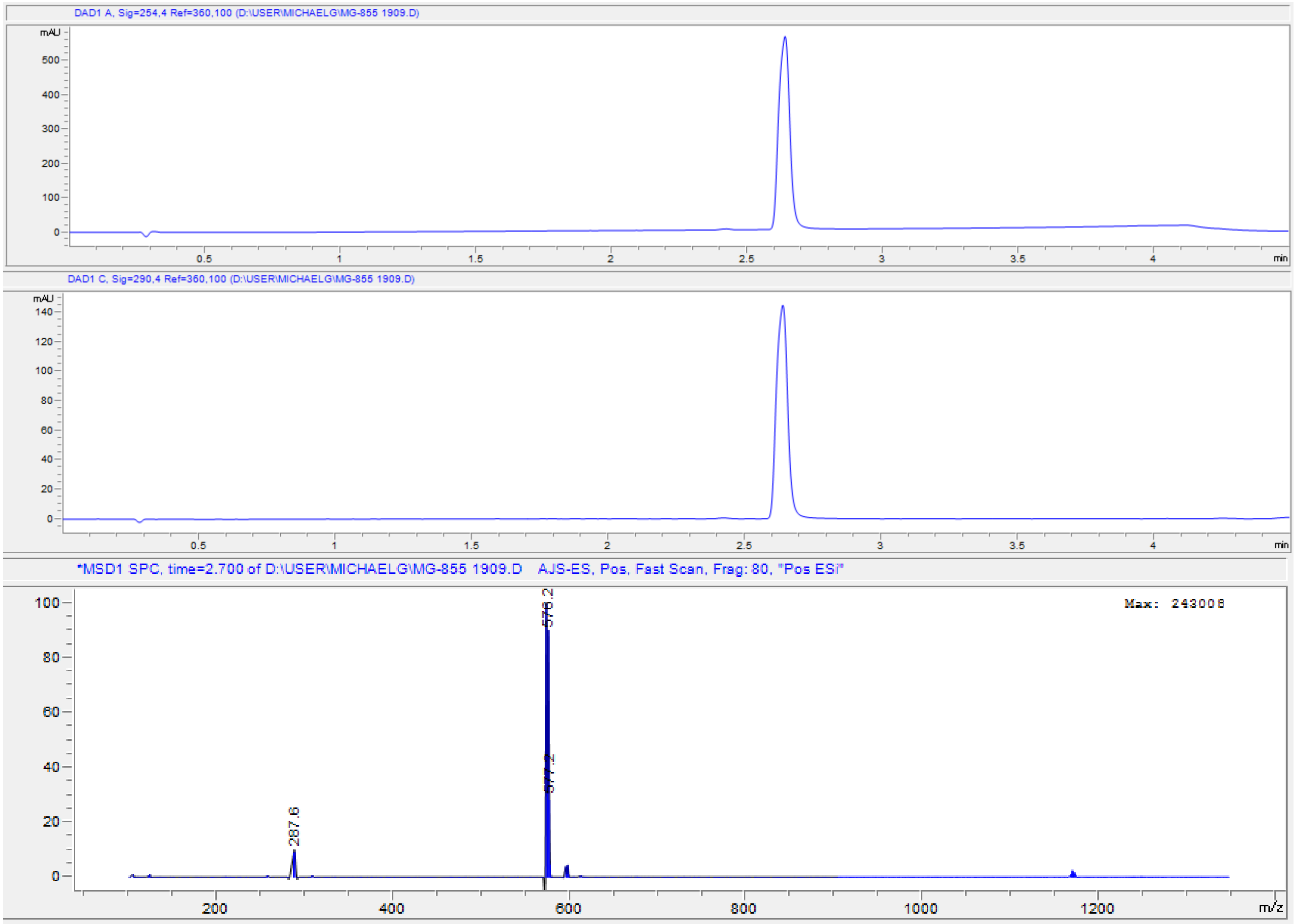

[1] C. A. Lipinski, F. Lombardo, B. W. Dominy, P. J. Feeney, *Adv Drug Deliver Rev* **1997**, *23*, 3.
[2] F. Lovering, J. Bikker, C. Humblet, *J Med Chem* **2009**, *52*, 6752.
[3] J. R. Silvius, F. l'Heureux, *Biochemistry* **1994**, *33*, 3014.
[4] J. Taipale, J. K. Chen, M. K. Cooper, B. Wang, R. K. Mann, L. Milenkovic, M. P. Scott, P. A. Beachy, *Nature* **2000**, *406*, 1005.
[5] L. Kremer, C. Schultz-Fademrecht, M. Baumann, P. Habenberger, A. Choidas, B. Klebl, S. Kordes, H. R. Scholer, J. Sterneckert, S. Ziegler, G. Schneider, H. Waldmann, *Angew Chem Int Edit* **2017**, *56*, 13021.
[6] M. W. Pfaffl, *Nucleic Acids Res* **2001**, *29*, e45.
[7] J. Cox, M. Mann, *Nature biotechnology* **2008**, *26*, 1367.
[8] S. Tyanova, J. Cox, *Methods in molecular biology (Clifton, N.J.)* **2018**, *1711*, 133.
[9] S.-C. Zhang, L. Gremer, H. Heise, P. Janning, A. Shymanets, I. C. Cirstea, E. Krause, B. Nürnberg, M. R. Ahmadian, *PLOS ONE* **2014**, *9*, e102425.
[10] E. Boulter, R. Garcia-Mata, C. Guilluy, A. Dubash, G. Rossi, P. J. Brennwald, K. Burridge, *Nature cell biology* **2010**, *12*, 477.
[11] J. Cox, M. Y. Hein, C. A. Luber, I. Paron, N. Nagaraj, M. Mann, *Mol Cell Proteomics* **2014**, *13*, 2513.
[12] S. Tyanova, T. Temu, P. Sinitcyn, A. Carlson, M. Y. Hein, T. Geiger, M. Mann, J. Cox, *Nat Methods* **2016**, *13*, 731.
[13] S. M. Hacker, K. M. Backus, M. R. Lazear, S. Forli, B. E. Correia, B. F. Cravatt, *Nat Chem* **2017**, *9*, 1181.
[14] a) M. P. Jacobson, R. A. Friesner, Z. Xiang, B. Honig, *J Mol Biol* **2002**, *320*, 597; b) M. P. Jacobson, D. L. Pincus, C. S. Rapp, T. J. Day, B. Honig, D. E. Shaw, R. A. Friesner, *Proteins* **2004**, *55*, 351.
[15] M. H. Olsson, C. R. Sondergaard, M. Rostkowski, J. H. Jensen, *J Chem Theory Comput* **2011**, *7*, 525.
[16] W. L. Jorgensen, J. Tirado-Rives, *J Am Chem Soc* **1988**, *110*, 1657.
[17] a) J. C. Shelley, A. Cholleti, L. L. Frye, J. R. Greenwood, M. R. Timlin, M. Uchimaya, *J Comput Aided Mol Des* **2007**, *21*, 681; b) J. R. Greenwood, D. Calkins, A. P. Sullivan, J. C. Shelley, *J Comput Aided Mol Des* **2010**, *24*, 591.
[18] A. D. Bochevarov, E. Harder, T. F. Hughes, J. R. Greenwood, D. A. Braden, D. M. Philipp, D. Rinaldo, M. D. Halls, J. Zhang, R. A. Friesner, *Int J Quantum Chem* **2013**, *113*, 2110.
[19] a) Y. C. Martin, *J Comput Aided Mol Des* **2009**, *23*, 693; b) P. Pospisil, P. Ballmer, L. Scapozza, G. Folkers, *J Recept Sig Transd* **2003**, *23*, 361.
[20] a) W. Sherman, T. Day, M. P. Jacobson, R. A. Friesner, R. Farid, *J Med Chem* **2006**, *49*, 534; b) W. Sherman, H. S. Beard, R. Farid, *Chem Biol Drug Des* **2006**, *67*, 83.
[21] a) R. A. Friesner, J. L. Banks, R. B. Murphy, T. A. Halgren, J. J. Klicic, D. T. Mainz, M. P. Repasky, E. H. Knoll, M. Shelley, J. K. Perry, D. E. Shaw, P. Francis, P. S. Shenkin, *J Med Chem* **2004**, *47*, 1739; b) T. A. Halgren, R. B. Murphy, R. A. Friesner, H. S. Beard, L. L. Frye, W. T. Pollard, J. L. Banks, *J Med Chem* **2004**, *47*, 1750; c) R. A. Friesner, R. B. Murphy, M. P. Repasky, L. L. Frye, J. R. Greenwood, T. A. Halgren, P. C. Sanschagrin, D. T. Mainz, *J Med Chem* **2006**, *49*, 6177.
[22] N. H. Keep, M. Barnes, I. Barsukov, R. Badii, L. Y. Lian, A. W. Segal, P. C. Moody, G. C. Roberts, *Structure (London, England : 1993)* **1997**, *5*, 623.
[23] K. J. Bowers, D. E. Chow, H. Xu, R. O. Dror, M. P. Eastwood, B. A. Gregersen, J. L. Klepeis, I. Kolossvary, M. A. Moraes, F. D. Sacerdoti, J. K. Salmon, Y. Shan, D. E. Shaw, *SC '06: Proceedings of the 2006 ACM/IEEE Conference on Supercomputing* **2006**, 43.
[24] H. J. C. Berendsen, J. R. Grigera, T. P. Straatsma, *J Phys Chem* **1987**, *91*, 6269.
